# Chiral phosphine-mediated intramolecular [3 + 2] annulation: enhanced enantioselectivity by achiral Brønsted acid†
†Electronic supplementary information (ESI) available. See DOI: 10.1039/c7sc00952f



**DOI:** 10.1039/c7sc00952f

**Published:** 2017-05-17

**Authors:** Weijun Yao, Zhaoyuan Yu, Shan Wen, Huanzhen Ni, Nisar Ullah, Yu Lan, Yixin Lu

**Affiliations:** a Department of Chemistry , National University of Singapore , 3 Science Drive 3 , Singapore 117543 . Email: chmlyx@nus.edu.sg; b Chemistry Department , King Fahd University of Petroleum and Materials , Dhahran 31261 , Saudi Arabia . Email: nullah@kfupm.edu.sa; c School of Chemistry and Chemical Engineering , Chongqing University , Chongqing 400030 , P. R. China . Email: lanyu@cqu.edu.cn; d National University of Singapore (Suzhou) Research Institute , 377 Lin Quan Street, Suzhou Industrial Park , Suzhou , Jiangsu 215123 , P. R. China

## Abstract

Enantioselective intramolecular [3 + 2] annulation of chalcones bearing an allene moiety has been successfully developed. The reaction was effectively promoted by amino acid-derived phosphines, in combination with achiral Brønsted acids. Dihydrocoumarin architectures were constructed in high yields and with excellent enantiomeric excesses. Theoretical studies *via* DFT calculations revealed that the hydrogen bonding network induced by achiral Brønsted acids/chiral phosphines could more efficiently distinguish between two enantioselective pathways, thus leading to enhanced enantioselectivity.

## Abstract 2

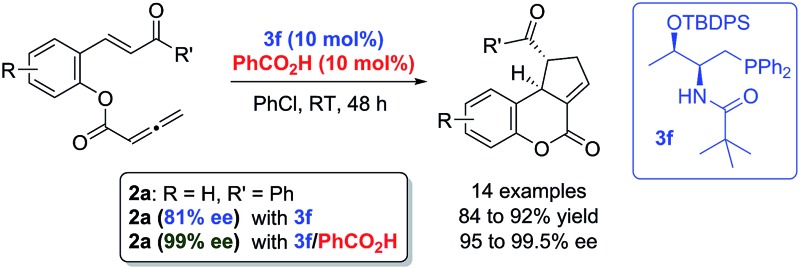
Enantioselective intramolecular [3 + 2] annulation of chalcone–allenes catalyzed by amino acid-derived phosphines and achiral Brønsted acids has been developed.

## 

In the past decade, asymmetric phosphine catalysis has emerged as an efficient approach for the construction of functionalized chiral carbocyclic structures.^1^ Allenes are the most commonly investigated substrates in phosphine catalysis, due to their high and versatile reactivities, as well as their ready synthetic accessibility.^2^ Since Lu’s pioneering report on phosphine-catalyzed [3 + 2] cyclization of allenoates with activated alkenes in 1995,^3^ a wide variety of asymmetric intermolecular annulation processes between allenes and activated alkenes have been developed, such as [3 + 2],^4^ [4 + 2],^5^ and [4 + 1]^6^ annulations, among others.^7^ However, phosphine-catalyzed intramolecular annulations are very rare. In 2003, Krische disclosed the first racemic version of intramolecular [3 + 2] annulation between enone and 2-alkynoate moieties, leading to the total synthesis of (±)-hirsutene.^8^ Subsequently, Kwon reported phosphine-promoted intramolecular [3 + 2] cyclizations of 2-styrenyl allenoates to form functionalized coumarins.^9^ Very recently, Fu developed an enantioselective intramolecular [3 + 2] cycloaddition of allenes and alkenes to create fused chiral ring scaffolds.^10^ In the past few years, our group has developed amino acid-based bifunctional phosphine catalysts, and demonstrated their applications in a wide range of enantioselective intermolecular annulation processes.^11^ Attracted by the great potential of phosphine-catalyzed intramolecular processes for quick access to challenging chiral skeletons, we became interested in such valuable transformations.

Intramolecular reactions proceed more readily than their intermolecular counterparts due to the intrinsic entropy difference. In phosphine catalysis, however, while phosphine-mediated asymmetric intermolecular cyclizations are very common, there is only one reported enantioselective intramolecular annulation to date.^10^ The paucity of this important reaction type may be due to the crowdedness of the advanced intermediates formed upon phosphine activation. This results in an inherent challenge to distinguish different transition states in a rather crowded and constrained environment. Herein, we document a highly enantioselective intramolecular [3 + 2] annulation of chalcones and allenes, promoted by a catalytic system combining chiral phosphines and achiral Brønsted acids, for highly diastereoselective and enantioselective construction of dihydrocoumarin architectures. We believe that introducing an additive molecule to interact with reaction partners synergistically may represent a novel and general approach to the discovery of asymmetric intramolecular processes in phosphine catalysis.

Substrate **1a**, which contains both chalcone and allene moieties, was chosen for initial investigation (Table 1). Achiral Ph_2_PMe effectively promoted the desired [3 + 2] annulation and led to the formation of the racemic tricyclic coumarin^12^ **2a** in 85% yield (entry 1). A series of l-valine-derived bifunctional phosphines were examined, and the amide functionality was found to be superior relative to sulfonamide and thiourea. Pivalamide **3d** was the best catalyst (entries 2–5). The threonine core^13^ again proved to be advantageous; l-threonine-based **3f** increased the ee value to 81% (entries 6 and 7). Dipeptide phosphines further enhanced the enantioselectivity when **4b** furnished **2a**, with an ee value of 87% (entry 9). Recently, Fu observed the beneficial effects of adding a proton donor in asymmetric γ-addition reactions.^14^ To further improve the enantioselectivity of the annulation reaction, we decided to introduce an achiral Brønsted acid additive as an extra controlling element for asymmetric induction. We hypothesized that the cooperative interplay of the phosphine catalyst, the substrate, and the acidic additive can add in a structural dimension to potentially make the transition states less constrained for such an intramolecular process. To our delight, the addition of benzoic acid (10 mol%) led to a substantial improvement in the enantioselectivity for the reactions catalyzed by mono-amino acid-derived phosphines, despite prolonged reaction times (entries 10–15). Notably, the addition of benzoic acid did not affect the enantioselectivity of this reaction when **4b** was employed as the catalyst (entry 16). To provide a more comprehensive picture, more Brønsted acid additives were investigated (entries 17–24). The beneficial effects of the Brønsted acid additives could be correlated to their acidities. Less acidic ethanol (p*K*_a_ 15.7) had little effect (entry 17) and phenol (p*K*_a_ 9.95) marginally increased the ee value (entry 18). *p*-Nitrophenol (p*K*_a_ 7.10), acetic acid (p*K*_a_ 4.76), and 3-(trifluoromethyl)benzoic acid (p*K*_a_ 3.77), which are similar to benzoic acid (p*K*_a_ 4.20), provided more enantioenriched products (entries 19–21). More acidic diphenylphosphoric acid (p*K*_a_ 1.90) led to slightly inferior results (entry 22). Too acidic trifluoroacetic acid (p*K*_a_ –0.25) or methanesulfonic acid (p*K*_a_ –2.6) inhibited the reaction, and no products were observed (entries 23 and 24). Essentially, the Brønsted acid additive needed to possess sufficient acidity to induce a better enantioselectivity, while a too acidic additive was found to be detrimental to the reaction. A catalytic system consisting of **3f** and benzoic acid was then selected, and subsequent solvent screening followed. It was revealed that chlorobenzene was the solvent of choice (entries 25–31). In the presence of the phosphine **3f**, with benzoic acid as an additive, the [3 + 2] annulation of **1a** in chlorobenzene proceeded smoothly to afford the dihydrocoumarin **2a** in 90% yield and with an ee value of 99%.

**Table 1:** Screening of catalysts & additives for enantioselective intramolecular [3 + 2] annulation^*a*^

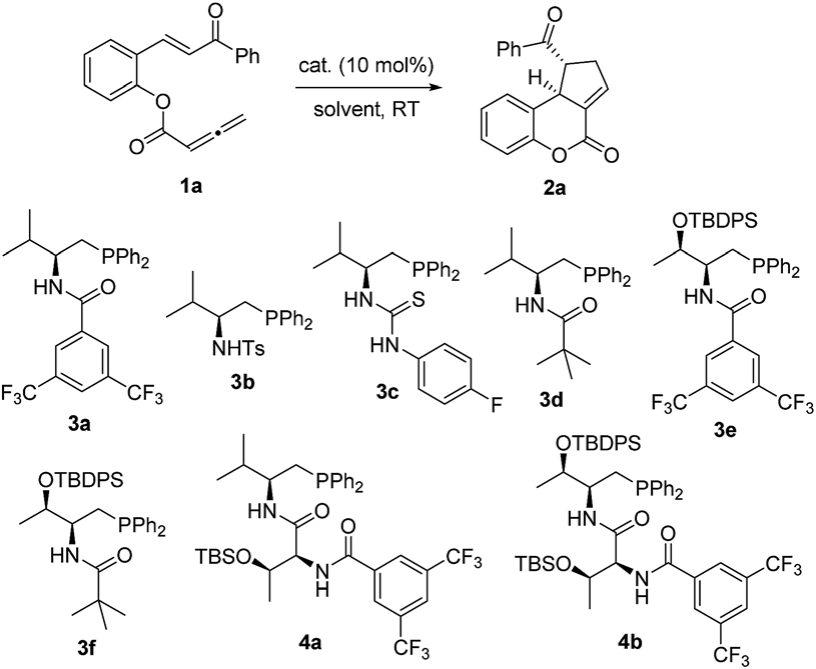					
Entry	Catalyst	Additive (p*K*_a_ in H_2_O)	Solvent	Yield^*b*^ [%]	ee^*c*^ [%]
1	MePPh_2_	—	Toluene	85	—
2	**3a**	—	Toluene	87	43
3	**3b**	—	Toluene	73	12
4	**3c**	—	Toluene	47	43
5	**3d**	—	Toluene	90	65
6	**3e**	—	Toluene	88	77
7	**3f**	—	Toluene	90	81
8	**4a**	—	Toluene	90	58
9	**4b**	—	Toluene	90	87
10	**3a**	PhCO_2_H (4.20)	Toluene	85	66
11	**3b**	PhCO_2_H (4.20)	Toluene	75	28
12	**3c**	PhCO_2_H (4.20)	Toluene	45	73
13	**3d**	PhCO_2_H (4.20)	Toluene	87	84
14	**3e**	PhCO_2_H (4.20)	Toluene	87	94
15	**3f**	PhCO_2_H (4.20)	Toluene	90	98
16	**4b**	PhCO_2_H (4.20)	Toluene	91	86
17	**3f**	EtOH (15.7)	Toluene	91	82
18	**3f**	PhOH (9.95)	Toluene	88	84
19	**3f**	4-NO_2_-PhOH (7.10)	Toluene	90	98
20	**3f**	CH_3_CO_2_H (4.76)	Toluene	88	98
21	**3f**	3-CF_3_PhCO_2_H (3.77)	Toluene	75	95
22	**3f**	(PhO)_2_P(O)OH (1.90)	Toluene	77	92
23	**3f**	TFA (–0.25)	Toluene	—	—
24	**3f**	CH_3_SO_3_H (–2.6)	Toluene	—	—
25	**3f**	PhCO_2_H (4.20)	EtOAc	72	93
26	**3f**	PhCO_2_H (4.20)	CH_2_Cl_2_	38	97
27	**3f**	PhCO_2_H (4.20)	CHCl_3_	41	95
28	**3f**	PhCO_2_H (4.20)	Ether	21	91
29	**3f**	PhCO_2_H (4.20)	THF	48	88
30	**3f**	PhCO_2_H (4.20)	Xylene	83	92
31	**3f**	PhCO_2_H (4.20)	PhCl	90	99

^*a*^Reactions were performed with **1a** (0.15 mmol) and the catalyst (0.015 mmol) in toluene (1.5 mL) at room temperature for 24 h; when an additive (0.015 mmol) was added, the reaction time was 48 h.

^*b*^Isolated yield for the major regioisomer.

^*c*^Determined by HPLC analysis on a chiral stationary phase.

With the optimized reaction conditions in hand, we next investigated the scope of the reaction (Table 2). Both aromatic moieties in chalcone structures could be varied, regardless of the electronic nature and substitution patterns of the aryl structures, and annulation products were obtained in high yields and with near perfect enantioselectivities (entries 1–14). In all of the examples examined, only one diastereomer was detected. The employment of the methyl-substituted enone **1o** or γ-methyl substituted allenoate **1p** did not lead to the desired product, presumably due to the low reactivity of such substrates. The absolute configurations of the annulation products were assigned on the basis of X-ray structural analysis of crystals of **2a**. The annulation product could be manipulated to form more complex ring structures. For instance, the [3 + 2] annulation product **2n** underwent intramolecular Mizoroki–Heck coupling to yield a unique coumarin derivative **5** in 76% yield (eqn (1)).1
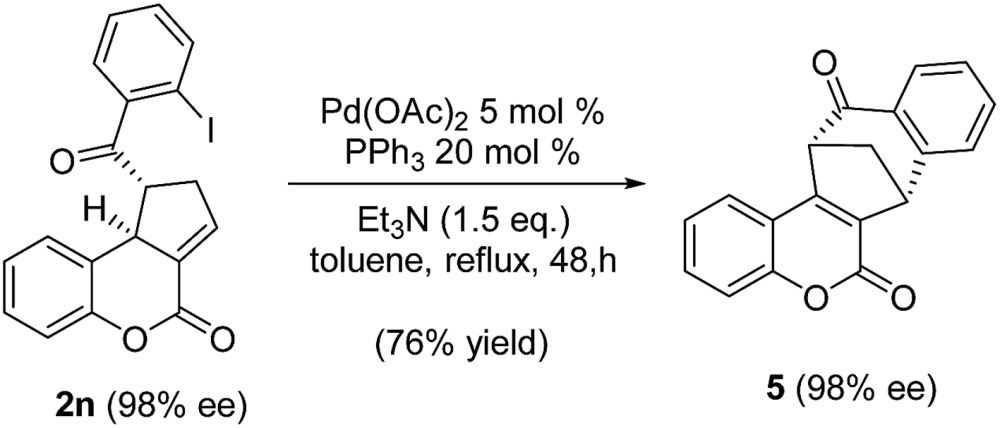



**Table 2:** The reaction scope^*a*^

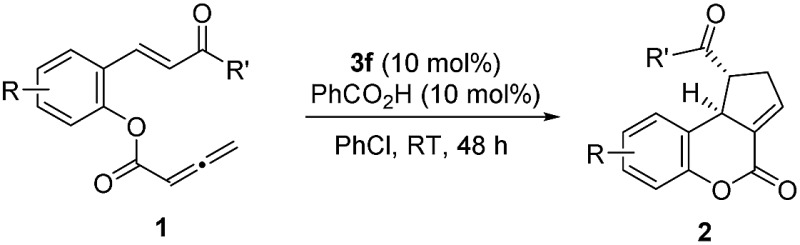				
Entry	Structure	**1** (R or R′)	Yield^*b*^ [%]	ee^*c*^ [%]
1		**1a** (R = H)	90	99
2		**1b** (R = 4-Me)	86	98
3		**1c** (R = 5-OMe)	87	99
4	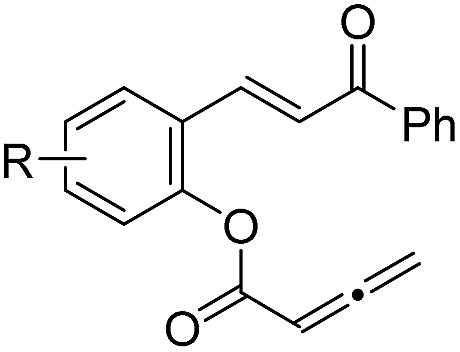	**1d** (R = 4-NO_2_)	84	95
5		**1e** (R = 2-Br)	90	98
6		**1f** (R = 5-Br)	92	98
7		**1g** (R = 4-Br)	89	98
8		**1h** (R = 4-Cl)	91	98
9		**1i** (R = 2,4-Cl)	89	98
10		**1j** (R′ = 4-MePh)	86	98
11	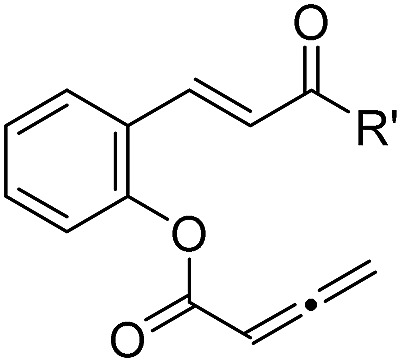	**1k** (R′ = 2-thiophenyl)	87	98
12		**1l** (R′ = 4-F-Ph)	86	99
13		**1m** (R′ = 3-Br-Ph)	87	99.5
14		**1n** (R′ = 2-I-Ph)	85	98
15	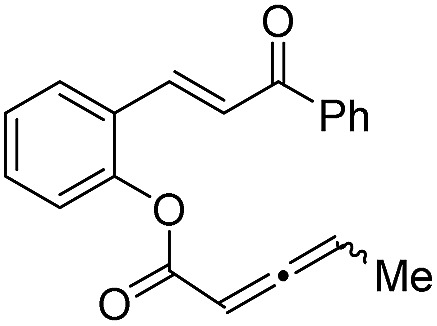	**1o** (R′ = Me)	—	—
16		**1p**	—	—

^*a*^Reactions were performed with **1** (0.15 mmol) and **3f** (0.015 mmol) and benzoic acid (0.015 mmol) in chlorobenzene (1.5 mL) at room temperature for 48 h.

^*b*^Isolated yield.

^*c*^Determined by HPLC analysis on a chiral stationary phase.

To further understand the role of benzoic acid in this cyclization process, we investigated the reaction with benzoic acid (10 mol%) as an additive in the presence of 3 equivalents of D_2_O (eqn (2)). The reaction proceeded smoothly to afford the desired product in 86% yield and with an ee value of 98%. ^1^H NMR showed that there was no deuterium incorporation in the product. This result suggests that benzoic acid is involved in the reaction through hydrogen bonding interactions, rather than facilitating the proton transfer process.2
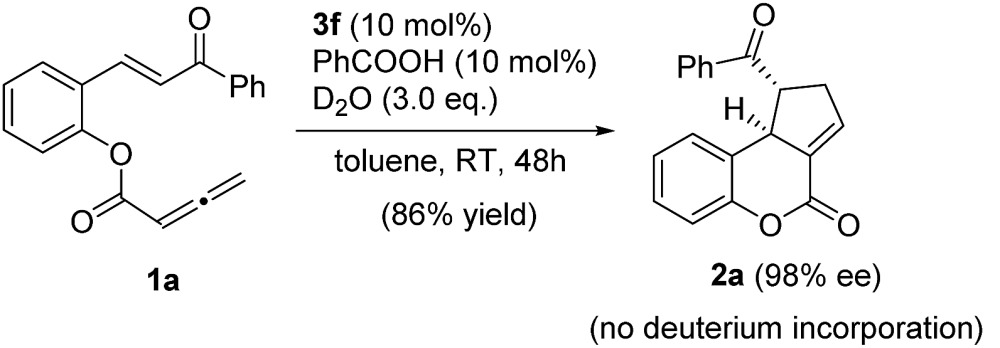



The mechanism of **3f**-catalyzed intramolecular [3 + 2] cyclization of chalcone allenoate is proposed in Scheme 1. An initial nucleophilic attack by **3f** on the allenoate **1a** forms the zwitterionic intermediate **7-int**. Without acid as an additive, **7-int** undergoes intramolecular Michael addition *via* the transition state **8-ts** to afford the intermediate **9-int**. An intramolecular Michael addition of **9-int** occurs *via* the transition state **10-ts** to give the intermediate **11-int**, followed by proton shift and elimination to yield the product **2a** and to release the catalyst **3f**. In this case, the ee value of the product is 81%. In the presence of an acidic additive, such as benzoic acid, a hydrogen bond network is formed between the zwitterionic intermediate **7-int** and benzoic acid to give complex **15-int**, and the subsequent intramolecular Michael addition takes place *via* the transition state **16-ts** to form the complex **17-int**. The enantioselectivity was improved to 98% in this pathway.

**Scheme 1:**
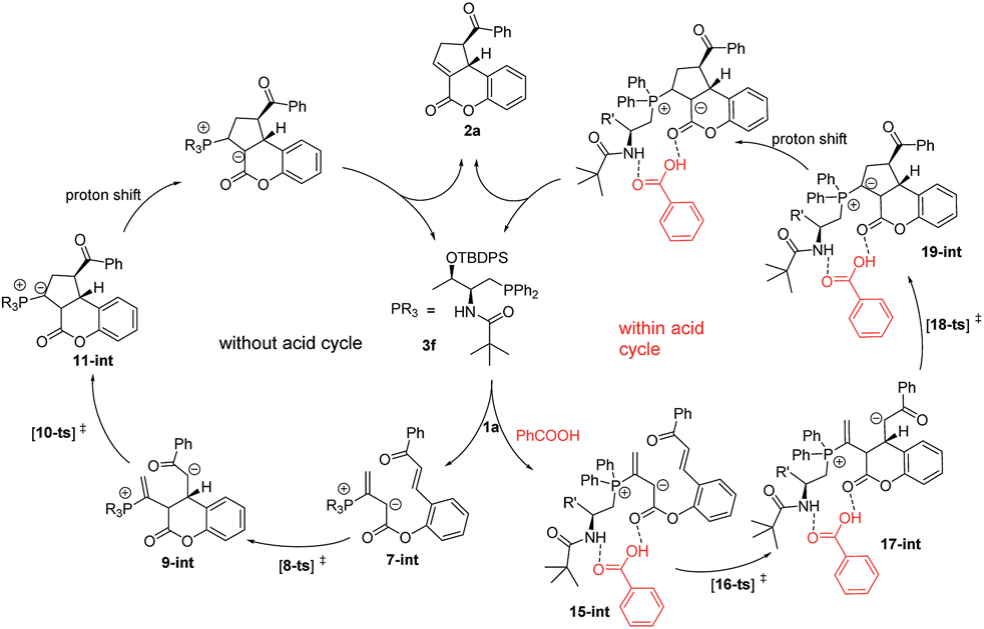
A proposed mechanism for the **3f**-catalyzed intramolecular [3 + 2] cyclization.

In order to rationalize the origin of the enantioselectivity^15^ and the effects of the acid additive in the annulation, density functional theory (DFT) calculations were carried out with GAUSSIAN 09 programs.^16^ The computed Gibbs free energy profiles of the intramolecular [3 + 2] cyclization of **1a** catalyzed by **3f** are shown in Fig. 1(a), and optimized structures of selected transition states are shown in Fig. 1(b). This multi-step cyclization process starts from the nucleophilic attack by **3f** on the allenoate **1a** *via* the transition state **6-ts**, which has an 18.6 kcal mol^–1^ energy barrier to form the zwitterionic intermediate **7-int**. The key enantio-differentiated cyclization then occurs *via* two possible pathways. The first pathway is the *Si*-face attack that occurs through **8-ts-*Si***, with a 2.5 kcal mol^–1^ barrier, generating the intermediate **9-int-*Si***. The subsequent ring closure occurs *via* the transition state **10-ts-*Si*** to form the intermediate **11-int-*Si***. The relative free energy of **10-ts-*Si*** is 11.3 kcal mol^–1^ lower than that of **8-ts-*Si***. The alternative *Re*-face attack proceeds *via* the transition state **8-ts-*Re*** with a barrier of 1.5 kcal mol^–1^, which is 1.0 kcal mol^–1^ lower than that of **8-ts-*Si***. The above calculations suggest that the enantioselectivity is determined by the cyclization step and predict an ee value of 69%, based on the energy difference between transition states **8-ts-*Re*** and **8-ts-*Si***. This is in good agreement with the experimental result, where the product **2a-*Re*** was formed preferentially. The analysis of the two transition states in Fig. 1(b) reveals the origin of the enantioselectivity; in the geometry of **8-ts-*Si***, the C···C distance of 3.86 Å suggests repulsion between the phenyl group of the reactant and the *tert*-butyl moiety of the phosphine catalyst, resulting in a higher transition state barrier.

**Fig. 1:**
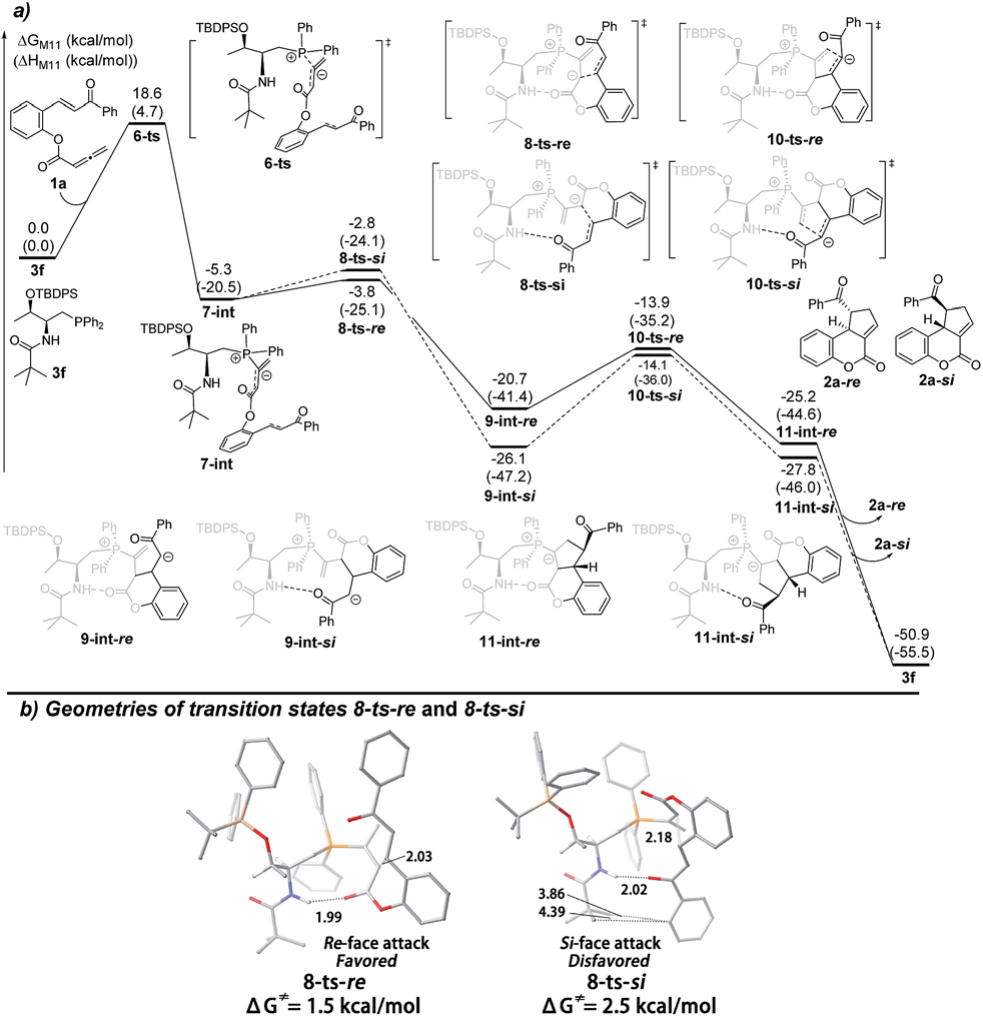
(a) The energy surface of intramolecular [3 + 2] cyclization catalyzed by the phosphine catalyst **3f**, and (b) the geometries of the transition states **8-ts-*Re*** and **8-ts-*Re***.

We next set out to understand the enhancement of enantioselectivity with the addition of benzoic acid. As the proton transfer process takes place after the key C–C bond formation, we were thus focusing on the involvement of benzoic acid in hydrogen bonding interactions to understand the observed enhancement of enantioselectivity. As shown in Fig. 2(a), a hydrogen-bonding complex **13-int** is formed from the active catalyst **3f** and benzoic acid **12** with a free energy increase of 7.4 kcal mol^–1^. The nucleophilic addition of phosphine takes place *via* the transition state **14-ts** with an overall barrier of 22.7 kcal mol^–1^, and generates the intermediate **15-int**. Similarly, from the intermediate **15-int**, the intramolecular Michael addition can then occur *via* two possible pathways: the *Si*-face attack pathway leads to the formation of **2a-*Si*** and the *Re*-face attack pathway gives the product **2a-*Re***. In both pathways, the benzoic acid forms two hydrogen bonds with the amide moiety of the catalyst and the ester group. In the presence of benzoic acid and two induced hydrogen bonding interactions, the two possible transition states, *i.e.* the *Si*-face attack pathway *via* **16-ts-*Si*** and the *Re*-face attack pathway *via* **16-ts-*Re***, are better differentiated. The calculated Gibbs free energy difference of 3.0 kcal mol^–1^ predicts an enantiomeric excess of 99%, fully consistent with the experimental results. The geometries of the transition states for the *Re*-face and *Si*-face attack involving benzoic acid are illustrated in Fig. 2(b). In the transition state **16-ts-*Si***, when the hydrogen bonds are formed the phosphorus bearing two phenyl groups is rotated and gets closer to the *tert*-butyl moiety of the phosphine catalyst. The C···C distance of 3.93 Å indicates that steric repulsion occurs, resulting in a higher transition state barrier.

**Fig. 2:**
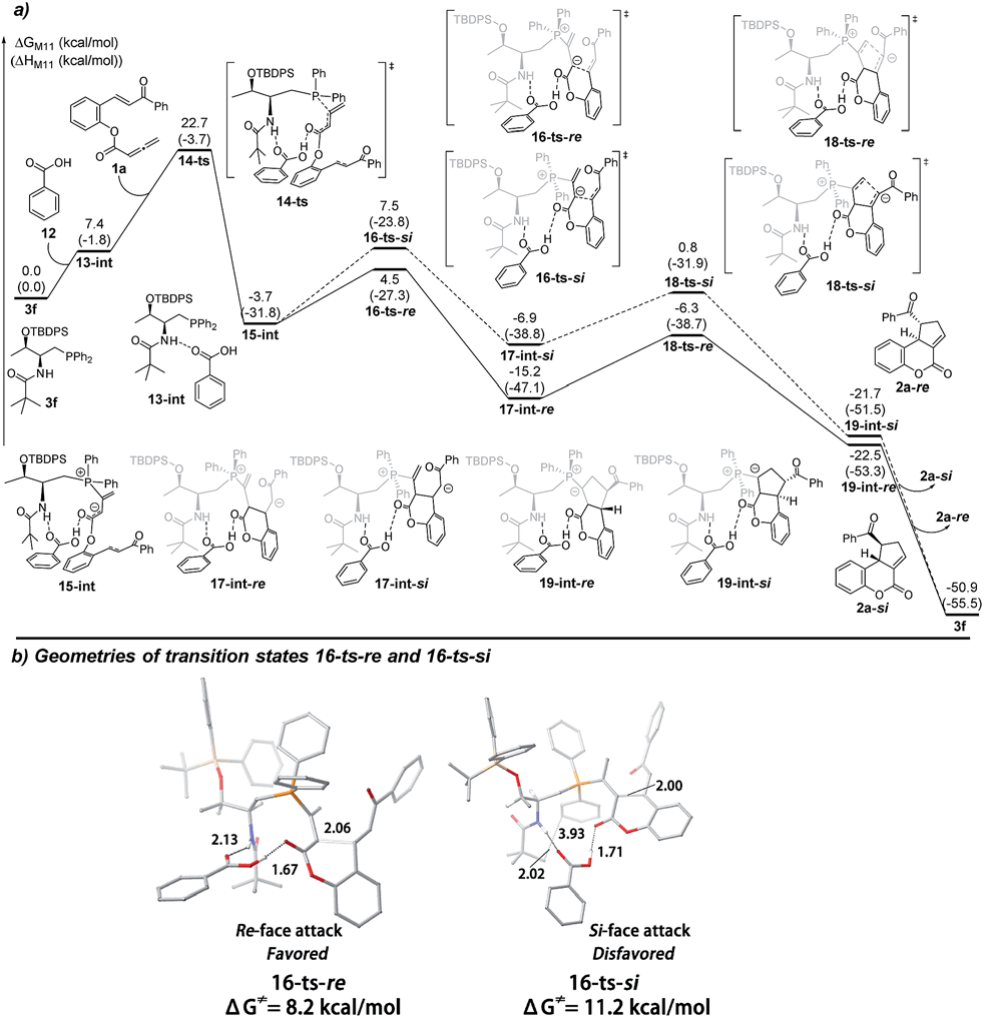
(a) The energy surface of intramolecular [3 + 2] cyclization cooperatively catalyzed by a phosphine catalyst with benzoic acid, and (b) the geometries of the transition states **16-ts-*Re*** and **16-ts-*Re*** .

## Conclusions

In summary, we have developed an enantioselective intramolecular [3 + 2] annulation of chalcones with allenes by employing a catalytic system combining chiral bifunctional phosphines and achiral Brønsted acids. Highly functionalized dihydrocoumarin scaffolds were obtained in high yields and with excellent enantioselectivities. Our DFT calculations revealed that the key hydrogen bonding network introduced by the achiral Brønsted acid additives was crucial for the observed enantioselectivity. The method described in this report may represent a general approach for the discovery of more phosphine-catalyzed enantioselective intramolecular processes. We are currently investigating in this direction, and our discoveries will be reported in due course.

## References

[cit1] Lu X., Zhang C., Xu Z. (2001). Acc. Chem. Res..

[cit2] Cowen B. J., Miller S. J. (2009). Chem. Soc. Rev..

[cit3] Zhang C., Lu X. (1995). J. Org. Chem..

[cit4] Zhu G., Chen Z., Jiang Q., Xiao D., Cao P., Zhang X. (1997). J. Am. Chem. Soc..

[cit5] Tran Y. S., Kwon O. (2007). J. Am. Chem. Soc..

[cit6] Zhang Q., Yang L., Tong X. (2010). J. Am. Chem. Soc..

[cit7] Takizawa S., Kishi K., Yoshida Y., Mader S., Arteaga F. A., Lee S., Hoshino M., Rueping M., Fujita M., Sasai H. (2015). Angew. Chem., Int. Ed..

[cit8] Wang J.-C., Ng S.-S., Krische M. J. (2003). J. Am. Chem. Soc..

[cit9] Henry C. E., Kwon O. (2007). Org. Lett..

[cit10] Lee S. Y., Fujiwara Y., Nishiguchi A., Kalek M., Fu G. C. (2015). J. Am. Chem. Soc..

[cit11] Han X., Chan W.-L., Yao W., Wang Y., Lu Y. (2016). Angew. Chem., Int. Ed..

[cit12] Kozak W., Dásko M., Masłyk M., Pieczykolan J. S., Gielniewski B., Rachona J., Demkowicz S. (2014). RSC Adv..

[cit13] Cheng L., Han X., Huang H., Wong M. W., Lu Y. (2007). Chem. Commun..

[cit14] Kalek M., Fu G. C. (2015). J. Am. Chem. Soc..

[cit15] Xia Y., Liang Y., Chen Y., Wang M., Jiao L., Huang F., Liu S., Li Y., Yu Z.-X. (2007). J. Am. Chem. Soc..

[cit16] See the ESI for the details of DFT calculations

