# Supplementary material for: Chiral phosphine-mediated intramolecular [3 + 2] annulation: enhanced enantioselectivity by achiral Brønsted acid
Source: Chem Sci. 2017 May 17;8(7):5196–200. doi: 10.1039/c7sc00952f (PMC5618691; doi:10.1039/c7sc00952f)
Supplement: Supplementary file 1 [file SC-008-C7SC00952F-s001.pdf]

Electronic Supplementary Material (ESI) for Chemical Science.
This journal is © The Royal Society of Chemistry 2017

Chiral Phosphine-mediated Intramolecular [3+2] Annulation: Enhanced Enantioselectivity by Achiral Brønsted Acid

Weijun Yao,[†] Zhaoyuan Yu,[†,‡] Shan Wen,[†] Huanzhen Ni,[†] Nisar Ullah,*[,||] Yu Lan,*[,‡] and Yixin Lu*[,†,§]

[†]Department of Chemistry, National University of Singapore, 3 Science Drive 3, Singapore 117543
[‡]School of Chemistry and Chemical Engineering, Chongqing University, Chongqing 400030, P. R. China
[||]Chemistry Department, King Fahd University of Petroleum and Materials, Dhahran 31261, Saudi Arabia
§National University of Singapore (Suzhou) Research Institute, 377 Lin Quan Street, Suzhou Industrial Park, Suzhou, Jiangsu, PR China, 215123

Emails: chmlyx@nus.edu.sg; lanyu@cqu.edu.cn; nullah@kfupm.edu.sa

Supporting Information

A. General information S2
B. Representative procedure for the synthesis of allenoates **1** S2
C. Analytical data of allenoates **1** S3
D. Representative procedure of the intramolecular [3+2] cyclization S7
E. Analytical data and HPLC chromatogram of products **2** S8
F. Intramolecular Heck reaction of **2n** S22
G. X-Ray crystallographic analysis and determination of the absolute configurations of the products S23
H. DFT studies S26
I. References S91
J. NMR spectra of the products S92

A. General information

Unless otherwise specified, all reactions were carried out under a nitrogen atmosphere, with dry, freshly distilled solvents in anhydrous conditions. THF, diethyl ether and toluene were distilled from sodium; while CH_2Cl_2 and CH_3CN were distilled from CaH_2 and ethyl acetate (EtOAc) and $CHCl_3$ were used without further purification. All chemicals were used without further purification as commercially available unless otherwise noted. Thin-layer chromatography (TLC) was performed on silica gel plates (60F-254) using UV-light (254 and 365 nm). Flash chromatography was conducted on silica gel (300–400 mesh). ^1H and ^{13}C NMR spectra were recorded on a Bruker AMX500 (500 MHz) spectrometer. Chemical shifts were reported in parts per million (ppm). All high resolution mass spectra were obtained on a Finnigan/MAT 95XL-T spectrometer. The racemic sample was prepared by $MePPh_2$. The catalysts **3** and **4** were prepared by following our previously reported procedures. Optical rotations were measured using a Jasco DIP-1000 polarimeter. Enantiomeric excesses were determined by HPLC analysis on a chiral stationary phase.

B. Representative procedure for the synthesis of allenoates 1

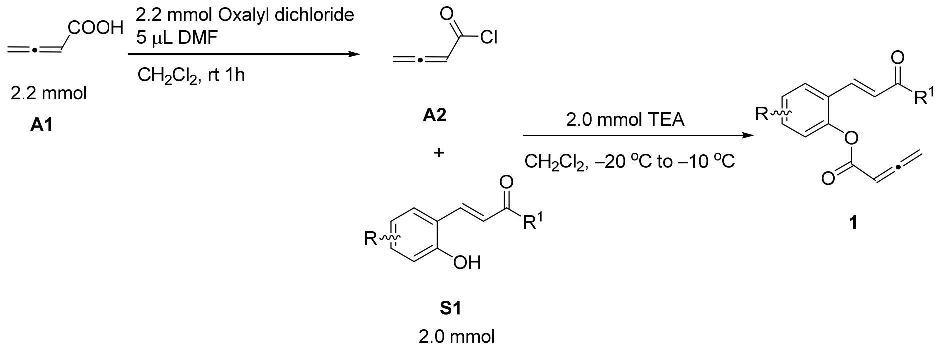

Allenoates **1** were synthesized by reacting in-situ prepared allenic acid chloride **A2** with the corresponding chalcone **S1**,[1] which were easily derived from the corresponding salicylaldehyde. To a stirred solution of allenic acid **A1**[2] (2.2 mmol) in anhydrous CH_2Cl_2 (10 mL) under nitrogen at room temperature was added oxalyl chloride (2.2 mmol, 189 µL), followed by DMF (5 µL). The resulting mixture was stirred further for 1 h and was used directly in the next step. To a flame dried round bottle flask with a magnetic stirring bar under N_2 were added chalcone **S1** (2.0 mmol) and anhydrous CH_2Cl_2 (10 mL), followed by Et_3N (2.0 mmol, 278 µL). The resulting mixture was cooled to -20 °C, and the freshly prepared **A2** solution was added dropwise over 15 min under nitrogen. The reaction mixture was kept at -20 °C for 1 h and then at -10 °C for another 2 h. The reaction was then quenched with iced water, and extracted with CH_2Cl_2 (2× 30 mL). The combined organic extracts were washed by brine (50 mL), dried over Na_2SO_4, filtered and concentrated. The residue was purified directly by flash column chromatography to afford allenoates **1**.

C. Analytical data of allenoates 1

(*E*)-2-(3-Oxo-3-phenylprop-1-en-1-yl)phenyl buta-2,3-dienoate **1a**

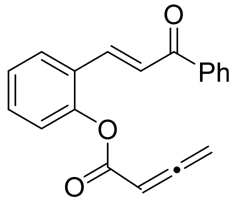

71% yield, a white solid. ^{1}H NMR (500 MHz, $CDCl_3$) δ 7.99 (dd, *J* = 5.2 Hz, 3.3 Hz, 2H), 7.90 (d, *J* = 15.9 Hz, 1H), 7.77 (dd, *J* = 7.8 Hz, 1.4 Hz, 1H), 7.62 – 7.57 (m, 1H), 7.53 – 7.48 (m, 3H), 7.44 (td, *J* = 8.1 Hz, 1.6 Hz, 1H), 7.31 (t, *J* = 7.6 Hz, 1H), 7.23 (dd, *J* = 8.1 Hz, 1.0 Hz, 1H), 5.87 (t, *J* = 6.5 Hz, 1H), 5.36 (d, *J* = 6.5 Hz, 2H); ^{13}C NMR (125 MHz, $CDCl_3$) δ 217.02, 190.57, 163.72, 149.79, 138.28, 138.11, 132.85, 131.26, 128.64, 128.58, 127.98, 127.74, 126.38, 124.34, 123.25, 87.37, 80.09; HRMS (ESI) m/z calcd for $C_{19}H_{14}NaO_3$ $[M+Na]^+$ = 313.0835, found = 313.0827.

(*E*)-4-Methyl-2-(3-oxo-3-phenylprop-1-en-1-yl)phenyl buta-2,3-dienoate **1b**

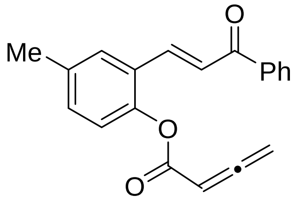

79% yield, a yellow oil.^{1}H NMR (500 MHz, $CDCl_3$) δ 7.91 (d, *J* = 8.2 Hz, 2H), 7.89 (d, *J* = 16.0 Hz, 1H), 7.76 (dd, *J* = 7.8 Hz, 1.2 Hz, 1H), 7.53 (d, *J* = 15.8 Hz, 1H), 7.46 – 7.40 (m, 1H), 7.30 (dd, *J* = 7.5 Hz, 4.4 Hz, 3H), 7.23 (d, *J* = 8.1 Hz, 1H), 5.87 (t, *J* = 6.5 Hz, 1H), 5.36 (d, *J* = 6.5 Hz, 2H), 2.44 (s, 3H); ^{13}C NMR (125 MHz, $CDCl_3$) δ 217.02, 189.93, 163.71, 149.76, 143.72, 137.81, 135.55, 131.10, 129.33, 128.71, 128.00, 127.88, 126.33, 124.36, 123.22, 87.39, 80.05, 21.67; HRMS (ESI) m/z calcd for $C_{20}H_{16}NaO_3$ $[M+Na]^+$ = 327.0992, found = 327.0988.

(*E*)-5-Methoxy-2-(3-oxo-3-phenylprop-1-en-1-yl)phenyl buta-2,3-dienoate **1c**

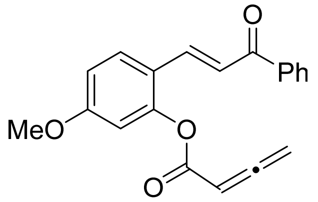

80% yield, a pale yellow solid. ^{1}H NMR (500 MHz, $CDCl_3$) δ 7.98 (d, *J* = 8.2 Hz, 2H), 7.85 (d, *J* = 15.8 Hz, 1H), 7.70 (dd, *J* = 8.8 Hz, 2.0 Hz, 1H), 7.60 – 7.54 (m, 1H), 7.52 – 7.47 (m, 2H), 7.43 (d, *J* = 15.8 Hz, 1H), 6.85 (dt, *J* = 8.8 Hz, 2.8 Hz, 1H), 6.77 (d, *J* = 2.0 Hz, 1H), 5.92 – 5.75 (m, 1H), 5.47 – 5.22 (m, 2H), 3.84 (d, *J* = 5.2 Hz, 3H); ^{13}C NMR (125 MHz, $CDCl_3$) δ 217.05, 190.63, 163.58, 162.17, 151.16, 138.43, 138.28, 132.60, 129.01, 128.56, 128.47, 121.83, 120.24, 113.06, 108.44, 87.36, 80.13, 55.68; HRMS (ESI) m/z calcd for $C_{20}H_{16}NaO_4$ $[M+Na]^+$ = 343.0941, found = 343.0933.

(*E*)-4-Nitro-2-(3-oxo-3-phenylprop-1-en-1-yl)phenyl buta-2,3-dienoate **1d**

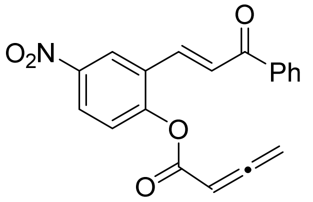

55% yield, a pale yellow solid. ^{1}H NMR (500 MHz, $CDCl_3$) δ 8.65 (d, *J* = 2.4 Hz, 1H), 8.33 – 8.21 (m, 1H), 8.06 – 7.99 (m, 2H), 7.91 (dd, *J* = 15.8 Hz, 2.7 Hz, 1H), 7.67 (d, *J* = 15.8 Hz, 1H), 7.62 (d, *J* = 7.3 Hz, 1H), 7.54 (t, *J* = 7.6 Hz, 2H), 7.48 (dd, *J* = 9.0 Hz, 2.5 Hz, 1H), 5.88 (t, *J* = 6.4 Hz, 1H), 5.44 (d, *J* = 6.4 Hz, 2H); ^{13}C NMR (125 MHz, $CDCl_3$) δ 217.52, 189.47, 162.71, 153.89,

145.60, 137.52, 135.51, 133.38, 129.17, 128.83, 128.64, 126.26, 125.62, 124.18, 122.94, 86.95, 80.55; HRMS (ESI) m/z calcd for $C_{19}H_{13}NNaO_5$ $[M+Na]^+$ = 358.0686, found = 358.0677.

(*E*)-2-Bromo-6-(3-oxo-3-phenylprop-1-en-1-yl)phenyl buta-2,3-dienoate **1e**

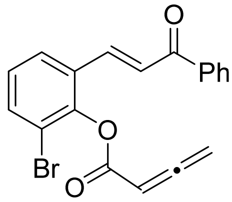

83% yield, a white solid. ^{1}H NMR (500 MHz, $CDCl_3$) δ 7.99 (d, *J* = 8.5 Hz, 2H), 7.79 (d, *J* = 15.8 Hz, 1H), 7.70 (d, *J* = 7.9 Hz, 1H), 7.66 (dd, *J* = 8.0 Hz, 1.3 Hz, 1H), 7.60 (t, *J* = 7.4 Hz, 1H), 7.54 – 7.48 (m, 3H), 7.20 (t, *J* = 7.9 Hz, 1H), 5.91 (t, *J* = 6.5 Hz, 1H), 5.40 (d, *J* = 6.4 Hz, 2H); ^{13}C NMR (125 MHz, $CDCl_3$) δ 217.40, 190.09, 162.64, 147.49, 137.85, 137.66, 134.81, 133.02, 130.57, 128.68, 128.59, 127.53, 126.99, 125.46, 118.12, 86.83, 80.25; HRMS (ESI) m/z calcd for $C_{19}H_{13}BrNaO_3$ $[M+Na]^+$ = 390.9940, found = 390.9937.

(*E*)-5-Bromo-2-(3-oxo-3-phenylprop-1-en-1-yl)phenyl buta-2,3-dienoate **1f**

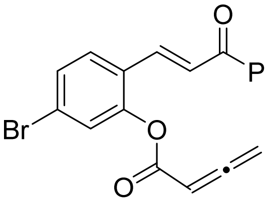

78% yield, a white solid. ^{1}H NMR (500 MHz, $CDCl_3$) δ 7.98 (d, *J* = 8.1 Hz, 1H), 7.81 (d, *J* = 15.8 Hz, 1H), 7.64 – 7.57 (m, 1H), 7.52 (d, *J* = 15.7 Hz, 1H), 7.50 (t, *J* = 7.5 Hz, 1H). 7.46 – 7.41 (m, 1H), 5.85 (t, *J* = 6.5 Hz, 1H), 5.37 (d, *J* = 6.4 Hz, 1H); ^{13}C NMR (125 MHz, $CDCl_3$) δ 217.21, 190.22, 163.25, 149.97, 137.95, 137.15, 132.99, 129.67, 128.81, 128.68, 128.55, 126.89, 126.63, 124.55, 124.38, 87.09, 80.25; HRMS (ESI) m/z calcd for $C_{19}H_{13}BrNaO_3$ $[M+Na]^+$ = 390.9940, found = 390.9931.

(*E*)-4-Bromo-2-(3-oxo-3-phenylprop-1-en-1-yl)phenyl buta-2,3-dienoate **1g**

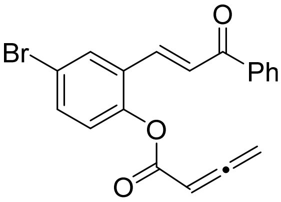

76% yield, a white solid. ^{1}H NMR (500 MHz, $CDCl_3$) δ 8.00 (d, *J* = 7.4 Hz, 2H), 7.88 (d, *J* = 2.3 Hz, 1H), 7.81 (d, *J* = 15.8 Hz, 1H), 7.60 (t, *J* = 7.4 Hz, 1H), 7.56 – 7.49 (m, 4H), 7.13 (d, *J* = 8.7 Hz, 1H), 5.85 (t, *J* = 6.5 Hz, 1H), 5.38 (d, *J* = 6.4 Hz, 1H); ^{13}C NMR (125 MHz, $CDCl_3$) δ 217.16, 189.93, 163.39, 148.69, 137.81, 136.56, 133.86, 133.09, 130.43, 129.78, 128.72, 128.59, 125.15, 124.89, 119.50, 87.16, 80.23; HRMS (ESI) m/z calcd for $C_{19}H_{13}BrNaO_3$ $[M+Na]^+$ = 390.9940, found = 390.9935.

(*E*)-4-Chloro-2-(3-oxo-3-phenylprop-1-en-1-yl)phenyl buta-2,3-dienoate **1h**

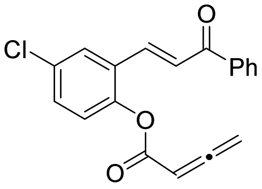

70% yield, a white solid. ^{1}H NMR (500 MHz, $CDCl_3$) δ 8.00 (d, *J* = 7.3 Hz, 2H), 7.82 (d, *J* = 15.8 Hz, 1H), 7.73 (d, *J* = 2.4 Hz, 1H), 7.65 – 7.56 (m, 1H), 7.56 – 7.46 (m, 3H), 7.39 (dd, *J* = 8.7 Hz, 2.4 Hz, 1H), 7.19 (d, *J* = 8.7 Hz, 1H), 5.85 (t, *J* = 6.5 Hz, 1H), 5.37 (d, *J* = 6.5 Hz, 2H); ^{13}C NMR (125 MHz, $CDCl_3$) δ 217.16, 189.94, 163.48, 148.16, 137.81, 136.66, 133.09, 131.86, 130.93,

129.35, 128.72, 128.59, 127.43, 125.13, 124.57, 87.16, 80.22; HRMS (ESI) m/z calcd for $C_{19}H_{13}ClNaO_3$ $[M+Na]^+$ = 347.0445, found = 347.0437.

(*E*)-2,4-Dichloro-6-(3-oxo-3-phenylprop-1-en-1-yl)phenyl buta-2,3-dienoate **1i**

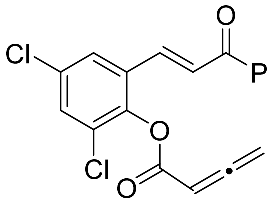

78% yield, a white solid. ^1H NMR (500 MHz, $CDCl_3$) δ 8.00 (d, *J* = 7.5 Hz, 2H), 7.73 (d, *J* = 15.8 Hz, 1H), 7.66 – 7.58 (m, 2H), 7.55 – 7.46 (m, 4H), 5.89 (t, *J* = 6.4 Hz, 1H), 5.41 (d, *J* = 6.5 Hz, 2H); ^{13}C NMR (125 MHz, $CDCl_3$) δ 217.50, 189.53, 162.45, 144.99, 137.57, 136.00, 133.27, 132.28, 131.50, 131.15, 129.58, 128.76, 128.61, 126.20, 125.96, 86.49, 80.38; HRMS (ESI) m/z calcd for $C_{19}H_{12}Cl_2NaO_3$ $[M+Na]^+$ = 381.0056, found = 381.0051.

(*E*)-2-(3-Oxo-3-(p-tolyl)prop-1-en-1-yl)phenyl buta-2,3-dienoate **1j**

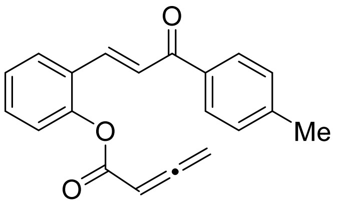

82% yield, a white solid. ^1H NMR (500 MHz, $CDCl_3$) δ 7.64 – 7.60 (m, 1H), 7.59 (d, *J* = 16.6 Hz, 1H), 7.42 – 7.36 (m, 1H), 7.25 (t, *J* = 7.4 Hz, 1H), 7.19 (dd, *J* = 8.2 Hz, 0.5 Hz, 1H), 6.71 (d, *J* = 16.3 Hz, 1H), 5.85 (t, *J* = 6.5 Hz, 1H), 5.38 (d, *J* = 6.5 Hz, 1H), 2.33 (s, 3H); ^{13}C NMR (125 MHz, $CDCl_3$) δ 217.00, 198.02, 163.59, 149.52, 136.60, 131.26, 128.97, 127.74, 127.19, 126.41, 123.12, 87.29, 80.08, 27.58; HRMS (ESI) m/z calcd for $C_{20}H_{16}NaO_3$ $[M+Na]^+$ = 327.0992, found = 327.0988.

(*E*)-2-(3-Oxo-3-(thiophen-2-yl)prop-1-en-1-yl)phenyl buta-2,3-dienoate **1k**

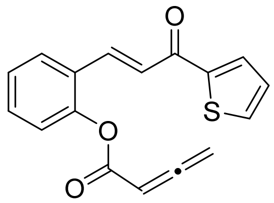

68% yield, a pale yellow solid. ^1H NMR (500 MHz, $CDCl_3$) δ 8.13 (d, *J* = 2.7 Hz, 1H), 7.90 (d, *J* = 15.8 Hz, 1H), 7.75 (d, *J* = 7.8 Hz, 1H), 7.65 (dd, *J* = 5.0 Hz, 0.9 Hz, 1H), 7.45 – 7.41 (m, 1H), 7.39 (d, *J* = 16.1 Hz, 1H), 7.36 (dd, *J* = 3.2 Hz, 1.9 Hz, 1H), 7.29 (t, *J* = 7.6 Hz, 1H), 7.22 (d, *J* = 8.1 Hz, 1H), 5.87 (t, *J* = 6.5 Hz, 1H), 5.39 (d, *J* = 6.5 Hz, 2H); ^{13}C NMR (125 MHz, $CDCl_3$) δ 217.05, 183.91, 163.73, 149.78, 142.96, 137.48, 132.18, 131.24, 127.88, 127.70, 127.46, 126.57, 126.37, 124.96, 123.23, 87.38, 80.14; HRMS (ESI) m/z calcd for $C_{17}H_{12}NaO_3S$ $[M+Na]^+$ = 319.0399, found = 319.0398.

(*E*)-2-(3-(4-Fluorophenyl)-3-oxoprop-1-en-1-yl)phenyl buta-2,3-dienoate **1l**

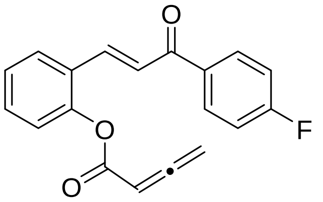

73% yield, a pale yellow solid. ^1H NMR (500 MHz, $CDCl_3$) δ 8.07 – 7.99 (m, 2H), 7.89 (d, *J* = 15.8 Hz, 1H), 7.76 (dd, *J* = 7.8 Hz, 1.4 Hz, 1H), 7.50 (d, *J* = 15.8 Hz, 1H), 7.47 – 7.41 (m, 1H), 7.31 (t, *J* = 7.6 Hz, 1H), 7.23 (d, *J* = 8.1 Hz, 1H), 7.17 (t, *J* = 8.6 Hz, 2H), 5.87 (t, *J* = 6.5 Hz, 1H), 5.37 (d, *J* = 6.5 Hz, 2H); ^{13}C NMR (125 MHz, $CDCl_3$) δ 217.03, 188.71, 165.64 (d, *J* = 254.6 Hz),

163.75, 149.82, 138.35, 134.39 (d, *J* = 2.9 Hz), 131.41, 131.18 (d, *J* = 9.3 Hz), 127.91, 127.61, 126.42, 123.71, 123.26, 115.77 (d, *J* = 21.8 Hz), 87.33, 80.14. HRMS (ESI) m/z calcd for $C_{19}H_{13}FNaO_3$ $[M+Na]^+$ = 331.0741, found = 331.0737.

(*E*)-2-(3-(3-Bromophenyl)-3-oxoprop-1-en-1-yl)phenyl buta-2,3-dienoate **1m**

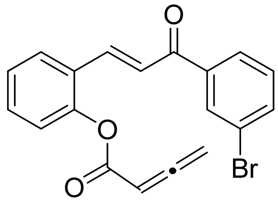

77% yield, a white solid. ^{1}H NMR (500 MHz, $CDCl_3$) δ 8.10 (t, *J* = 1.7 Hz, 1H), 7.89 (d, *J* = 15.7 Hz, 1H), 7.90 – 7.88 (m, 1H), 7.74 (dd, *J* = 7.8 Hz, 1.4 Hz, 1H), 7.68 (ddd, *J* = 7.9 Hz, 1.9 Hz, 1.0 Hz, 1H), 7.45 (t, *J* = 8.5 Hz, 1H), 7.42 (dd, *J* = 7.9 Hz, 1.2 Hz, 1H), 7.35 (t, *J* = 7.9 Hz, 1H), 7.31 – 7.26 (m, 1H), 7.21 (dd, *J* = 8.2 Hz, 1.1 Hz, 1H), 5.87 (t, *J* = 6.5 Hz, 1H), 5.37 (d, *J* = 6.5 Hz, 2H); ^{13}C NMR (125 MHz, $CDCl_3$) δ 217.05, 188.92, 163.70, 149.88, 139.85, 139.12, 135.70, 131.59, 131.53, 130.28, 128.23, 127.43, 127.08, 126.45, 123.52, 123.31, 122.97, 87.36, 80.20; HRMS (ESI) m/z calcd for $C_{19}H_{13}BrNaO_3$ $[M+Na]^+$ = 390.9940, found = 390.9936.

(*E*)-2-(3-(2-Iodophenyl)-3-oxoprop-1-en-1-yl)phenyl buta-2,3-dienoate **1n**

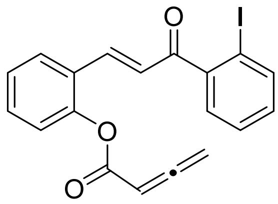

72% yield, a white solid. ^{1}H NMR (500 MHz, $CDCl_3$) δ 7.91 (dd, *J* = 7.9 Hz, 0.8 Hz, 1H), 7.68 (dd, *J* = 7.8 Hz, 1.5 Hz, 1H), 7.51 (d, *J* = 16.3 Hz, 1H), 7.44 – 7.39 (m, 2H), 7.36 (dd, *J* = 7.6 Hz, 1.7 Hz, 1H), 7.27 (t, *J* = 7.5 Hz, 1H), 7.19 (dd, *J* = 8.2 Hz, 1.0 Hz, 1H), 7.14(td, *J* = 7.6 Hz, 1.7Hz, 1H), 7.08 (d, *J* = 16.3 Hz, 1H), 5.75 (t, *J* = 6.5 Hz, 1H), 5.24 (d, *J* = 6.5 Hz, 2H); ^{13}C NMR (125 MHz, $CDCl_3$) δ 216.89, 195.83, 163.51, 149.69, 144.56, 140.47, 140.01, 131.72, 131.34, 128.66, 128.04, 127.97, 127.45, 127.17, 126.49, 123.24, 92.24, 87.18, 80.05. HRMS (ESI) m/z calcd for $C_{19}H_{13}INaO_3$ $[M+Na]^+$ = 438.9802, found = 438.9797.

(*E*)-2-(3-oxobut-1-en-1-yl)phenyl buta-2,3-dienoate **1o**

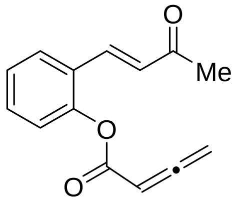

81% yield, a pale yellow oil. ^{1}H NMR (400 MHz, $CDCl_3$) δ 7.65 – 7.53 (m, 2H), 7.37 (t, *J* = 7.7 Hz, 1H), 7.21 (t, *J* = 7.5 Hz, 1H), 7.17 (d, *J* = 8.1 Hz, 1H), 6.70 (d, *J* = 16.4 Hz, 1H), 5.84 (t, *J* = 6.4 Hz, 1H), 5.37 (d, *J* = 6.5 Hz, 2H), 2.31 (s, 3H); ^{13}C NMR (100 MHz, $CDCl_3$) δ 216.9, 198.1, 163.6, 149.5, 136.6, 131.2, 128. 9, 127.7, 127.1, 126.4, 123.1, 87.2, 80.1, 27. 6. HRMS (ESI) m/z calcd for $C_{14}H_{12}NaO_3$ $[M+Na]^+$ = 251.0679, found = 251.0685.

(*E*)-2-(3-oxo-3-phenylprop-1-en-1-yl)phenyl penta-2,3-dienoate **1p**

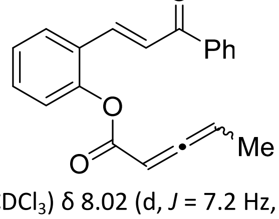

77% yield, a pale yellow oil. ^{1}H NMR (500 MHz, $CDCl_3$) δ 8.02 (d, *J* = 7.2 Hz, 2H), 7.93 (d, *J* = 15.9 Hz, 1H), 7.78 (dd, *J* = 7.8, 1.1 Hz, 1H), 7.62 – 7.58 (m, 1H), 7.55 (d, *J* = 15.9 Hz, 1H), 7.51 (t, *J* = 7.7 Hz, 2H), 7.45 (td, *J* = 8.2, 1.4 Hz, 1H), 7.31 (t, *J* = 7.6 Hz, 1H), 7.26 (d, *J* = 8.1 Hz, 1H), 5.81 (dq, *J* = 6.2, 3.1 Hz, 1H), 5.74 (dt, *J* = 7.2, 4.1 Hz, 1H), 1.86 (dd, *J* = 7.4, 3.2 Hz, 3H); ^{13}C

NMR (125 MHz, $CDCl_3$) δ 214.4, 190.5, 164.2, 150.0, 138.3, 138.1, 132.9, 131.2, 128.6, 128.6, 128.0, 127.7, 126.3, 124.2, 123.3, 91.2, 86.9, 12.6; HRMS (ESI) m/z calcd for $C_{20}H_{16}NaO_3$ $[M+Na]^+$ = 327.0992, found = 327.1001.

D. Representative procedure of the intramolecular [3+2] cyclization

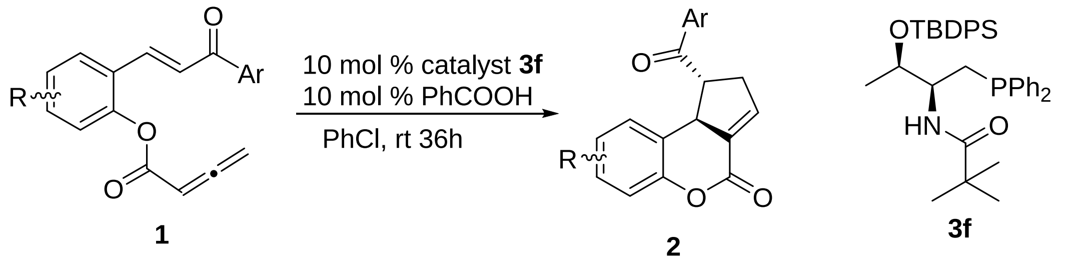

To a flame dried round bottle flask with a magnetic stirring bar at room temperature under nitrogen were added allenoate **1** (0.15 mmol), benzoic acid (0.015 mmol, 1.8 mg) and chlorobenzene (3 mL), followed by the addition of **3f** (0.015 mmol, 8.9 mg). The resulting mixture was stirred for 36 h and was directly purified by column chromatography on silica gel to afford annulation adduct **2**.

E. Analytical data and HPLC chromatogram of products 2

(1*R*,9b*R*)-1-Benzoyl-2,9b-dihydrocyclopenta[c]chromen-4(1*H*)-one **2a**

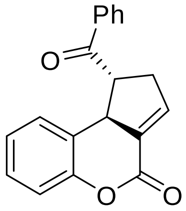

90% yield, a white solid $[\alpha]^{25}_{D}$ = –195 (c 1.0, $CHCl_3$). ^{1}H NMR (500 MHz, $CDCl_3$) δ 8.05 (d, *J* = 7.8 Hz, 2H), 7.67 (t, *J* = 7.4 Hz, 1H), 7.56 (t, *J* = 7.7 Hz, 2H), 7.23 (t, *J* = 7.7 Hz, 1H), 7.09 (d, *J* = 8.1 Hz, 1H), 7.03 (t, *J* = 7.5 Hz, 1H), 6.89 (dd, *J* = 5.4 Hz, 2.9 Hz, 1H), 6.85 (d, *J* = 7.6 Hz, 1H), 5.03 (dd, *J* = 8.2 Hz, 3.4 Hz, 1H), 4.42 (dd, *J* = 18.9 Hz, 9.6 Hz, 1H), 3.17 (ddt, *J* = 18.1 Hz, 9.6 Hz, 3.0 Hz, 1H), 2.76 (dddd, *J* = 18.0 Hz, 10.1 Hz, 4.1 Hz, 2.1 Hz, 1H). ^{13}C NMR (125 MHz, $CDCl_3$) δ 199.20, 160.21, 150.85, 141.52, 135.87, 133.94, 131.41, 129.09, 128.70, 128.39, 126.04, 125.97, 124.93, 117.27, 55.59, 44.00, 39.04. HRMS (ESI) m/z calcd for $C_{19}H_{14}NaO_3$ $[M+Na]^+$ = 313.0835, found = 313.0827. The ee value was 99%, t_R (major) = 16.25 min, t_R (minor) = 21.45 min (Chiralpak ID, λ = 254 nm, 45% *i*-PrOH/hexane, flow rate = 1.0 mL/min).

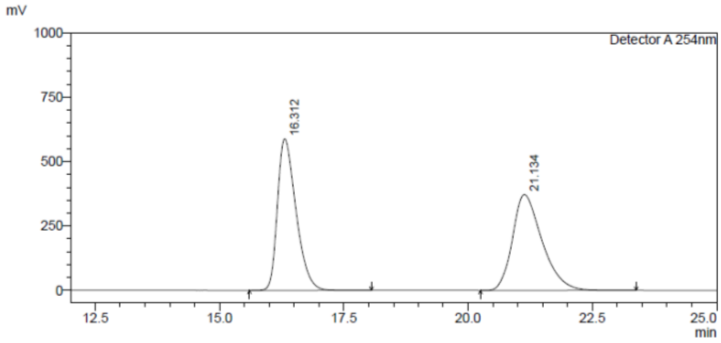

<Peak Table>

Detector A 254nm

Peak#	Ret. Time	Area	Height	Conc.	Unit	Mark	Name
1	16.312	15278256	588549	49.838			
2	21.134	15377393	371941	50.162			
Total		30655649	960490				

Racemic **2a**

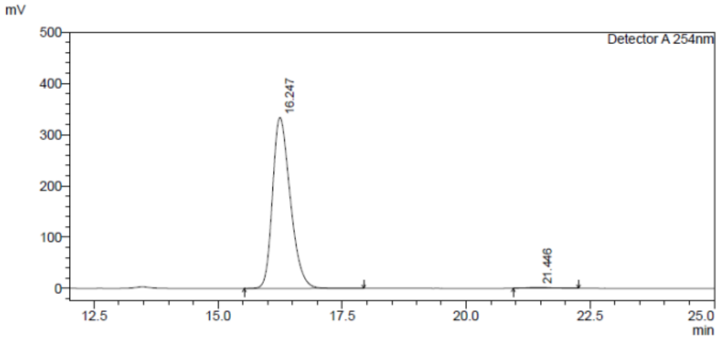

<Peak Table>

Detector A 254nm

Peak#	Ret. Time	Area	Height	Conc.	Unit	Mark	Name
1	16.247	8300984	333618	99.302			
2	21.446	58348	1742	0.698		M	
Total		8359332	335360				

Enantiomerically enriched **2a**

(1*R*,9b*R*)-1-Benzoyl-8-methyl-2,9b-dihydrocyclopenta[c]chromen-4(1*H*)-one **2b**

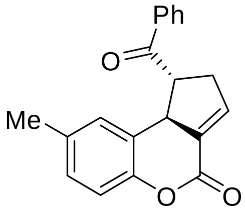

86% yield, a pale yellow foam, $[\alpha]^{25}_{D}$ = –145 (c 0.5, $CHCl_3$). ^{1}H NMR (500 MHz, $CDCl_3$) δ 8.08 (dd, *J* = 5.1 Hz, 3.4 Hz, 2H), 7.74 – 7.67 (m, 1H), 7.59 (t, *J* = 7.7 Hz, 2H), 7.07 – 6.96 (m, 2H), 6.88 (dd, *J* = 5.4 Hz, 3.1 Hz, 1H), 6.66 (s, 1H), 5.01 (dd, *J* = 8.1 Hz, 3.5 Hz, 1H), 4.42 (dd, *J* = 18.8 Hz, 9.8 Hz, 1H), 3.19 (ddt, *J* = 18.1 Hz, 9.7 Hz, 3.0 Hz, 1H), 2.77 (dddd, *J* = 18.1 Hz, 10.1 Hz, 4.1 Hz, 2.1 Hz, 1H), 2.22 (s, 3H); ^{13}C NMR (125 MHz, $CDCl_3$) δ 199.2, 160.4, 148.8, 141.2, 135.9, 134.6, 133. 9, 131.6, 129.1, 128.9, 128.7, 126.2, 125.7, 117.0, 55.6, 43.8, 39.0, 20.8; HRMS (ESI) m/z calcd for $C_{20}H_{16}NaO_3$ $[M+Na]^+$ = 327.0992, found = 327.0986. The ee value was 98%, t_R (major) = 15.12 min, t_R (minor) = 19.12 min (Chiralpak ID, λ = 254 nm, 45% *i*-PrOH/hexane, flow rate = 1.0 mL/min).

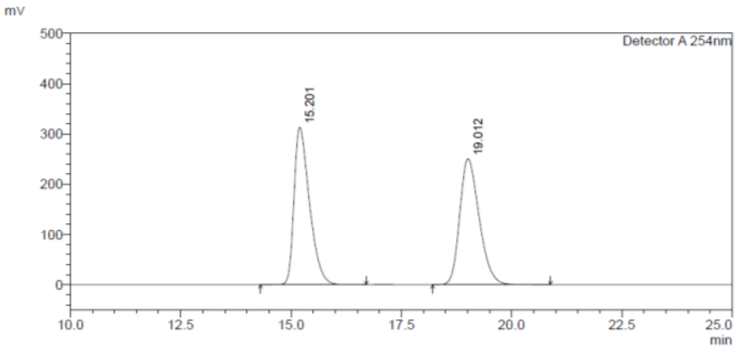

<Peak Table>

Detector A 254nm

Peak#	Ret. Time	Area	Height	Conc.	Unit	Mark	Name
1	15.201	7640951	313443	49.828			
2	19.012	7693656	250701	50.172			
Total		15334607	564144				

Racemic **2b**

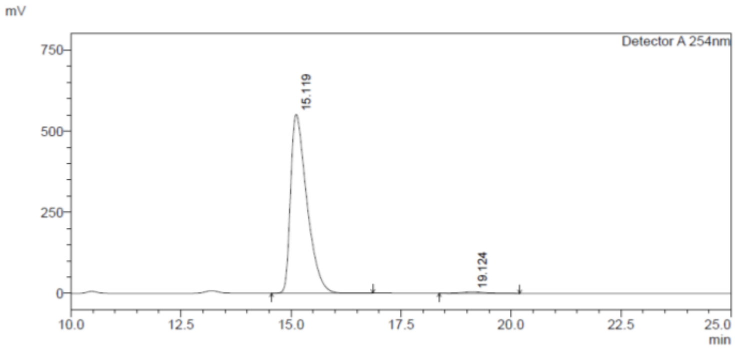

<Peak Table>

Detector A 254nm

Peak#	Ret. Time	Area	Height	Conc.	Unit	Mark	Name
1	15.119	14238243	551179	98.966			
2	19.124	148726	4193	1.034			
Total		14386969	555371				

Enantiomerically enriched **2b**

(1*R*,9b*R*)-1-Benzoyl-7-methoxy-2,9b-dihydrocyclopenta[c]chromen-4(1*H*)-one **2c**

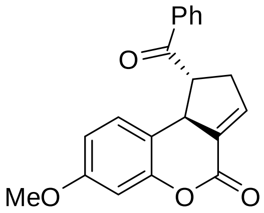

87% yield, a pale yellow foam, $[\alpha]^{25}_{D}$ = –117 (c 0.4, $CHCl_3$). ^{1}H NMR (500 MHz, $CDCl_3$) δ 8.03 (d, *J* = 8.3 Hz, 2H), 7.71 – 7.61 (m, 1H), 7.55 (t, *J* = 7.7 Hz, 2H), 6.87 (dd, *J* = 5.2 Hz, 2.3 Hz, 1H), 6.74 (d, *J* = 8.5 Hz, 1H), 6.63 (d, *J* = 2.4 Hz, 1H), 6.61 – 6.55 (m, 1H), 4.92 (dd, *J* = 8.2 Hz, 3.5 Hz, 1H), 4.35 (q, *J* = 9.5 Hz, 1H), 3.75 (s, 3H), 3.14 (ddt, *J* = 18.1 Hz, 9.6 Hz, 2.8 Hz, 1H), 2.85 – 2.57 (m, 1H); ^{13}C NMR (125 MHz, $CDCl_3$) δ 199.36, 160.18, 159.68, 151.54, 141.55, 135.94, 133.89, 131.57, 129.06, 128.67, 126.55, 117.92, 111.06, 102.73, 55.98, 55.54, 43.45, 39.00; HRMS (ESI) m/z calcd for $C_{20}H_{16}NaO_4$ $[M+Na]^+$ = 343.0941, found = 343.0933. The ee value was 98%, t_R (major) = 24.39 min, t_R (minor) = 30.95 min (Chiralpak ID, λ = 254 nm, 45% *i*-PrOH/hexane, flow rate = 1.0 mL/min).

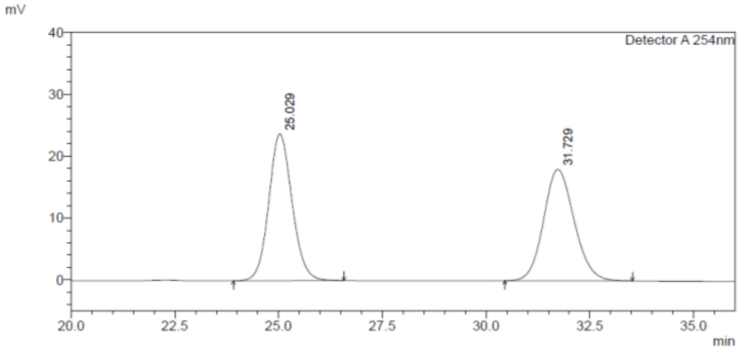

<Peak Table>

Detector A 254nm

Peak#	Ret. Time	Area	Height	Conc.	Unit	Mark	Name
1	25.029	928346	23745	50.042			
2	31.729	926773	18024	49.958			
Total		1855119	41769				

Racemic **2c**

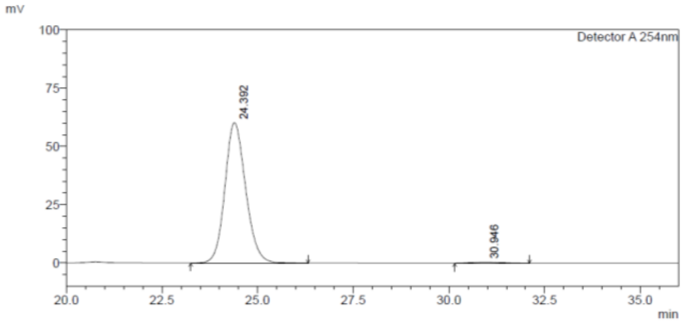

<Peak Table>

Detector A 254nm

Peak#	Ret. Time	Area	Height	Conc.	Unit	Mark	Name
1	24.392	2278569	60316	98.925			
2	30.946	24772	505	1.075			
Total		2303341	60821				

Enantiomerically enriched **2c**

(1*R*,9b*R*)-1-Benzoyl-8-nitro-2,9b-dihydrocyclopenta[c]chromen-4(1*H*)-one **2d**

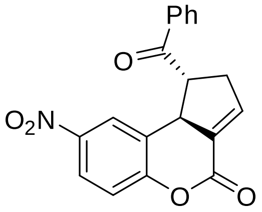

84% yield, a yellow solid, $[\alpha]^{25}_{D}$ = –167 (c 1.0, $CHCl_3$). 1H NMR (500 MHz, CDCl3) δ 8.18 – 8.10 (m, 1H), 8.08 – 8.00 (m, 2H), 7.78 (dd, J = 2.5 Hz, 1.4 Hz, 1H), 7.69 (t, J = 7.4 Hz, 1H), 7.58 (t, J = 7.8 Hz, 2H), 7.23 (d, J = 9.0 Hz, 1H), 6.99 (dd, J = 5.4 Hz, 3.1 Hz, 1H), 5.10 (dd, J = 7.6 Hz, 3.2 Hz, 1H), 4.44 (dd, J = 18.6 Hz, 9.7 Hz, 1H), 3.26 (ddt, J = 18.5 Hz, 9.8 Hz, 3.0 Hz, 1H), 2.82 (dddd, J = 18.5 Hz, 9.9 Hz, 4.2 Hz, 2.1 Hz, 1H); 13C NMR (125 MHz, CDCl3) δ 198.41, 158.33, 155.26, 144.51, 143.57, 135.47, 134.27, 129.32, 129.21, 128.80, 127.47, 124.34, 122.02, 118.23, 55.42, 43.80, 39.09; HRMS (ESI) m/z calcd for $C_{19}H_{13}NNaO_5$ $[M+Na]^+$ = 358.0686, found = 358.0677. The ee value was 95%, t_R (major) = 24.37 min, t_R (minor) = 21.89 min (Chiralpak ID, λ = 254 nm, 45% *i*-PrOH/hexane, flow rate = 1.0 mL/min).

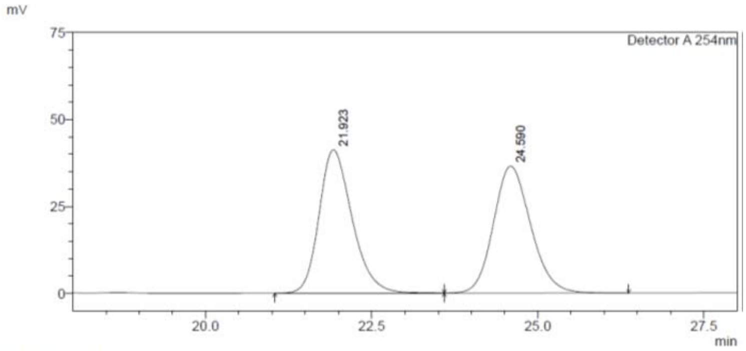

<Peak Table>

Detector A 254nm

Peak#	Ret. Time	Area	Height	Conc.	Unit	Mark	Name
1	21.923	1429212	41225	50.045			
2	24.590	1426668	36511	49.955		V	
Total		2855879	77735				

Racemic **2d**

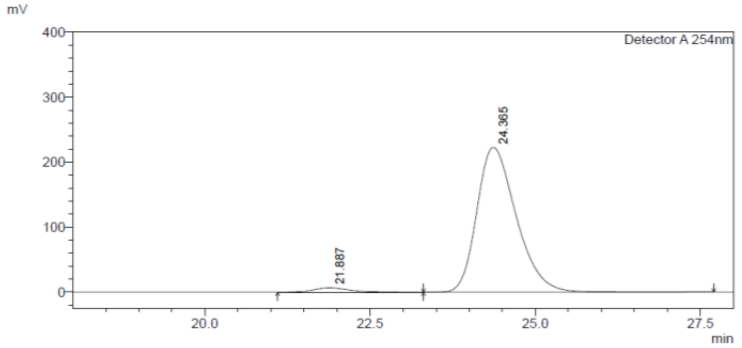

<Peak Table>

Detector A 254nm

Peak#	Ret. Time	Area	Height	Conc.	Unit	Mark	Name
1	21.887	243895	6676	2.572		S	
2	24.365	9240224	222767	97.428		S	
Total		9484118	229443				

Enantiomerically enriched **2d**

(1*R*,9b*R*)-1-Benzoyl-6-bromo-2,9b-dihydrocyclopenta[c]chromen-4(1*H*)-one **2e**

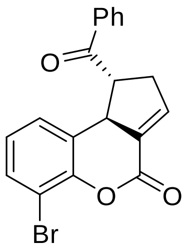

90% yield, a pale yellow solid, $[\alpha]^{25}_{D}$ = –203 (c 0.5, $CHCl_3$) . ^{1}H NMR (500 MHz, $CDCl_3$) δ8.04 (d, *J* = 8.3 Hz, 2H), 7.67 (t, *J* = 7.3 Hz, 1H), 7.56 (t, *J* = 7.7 Hz, 2H), 7.49 – 7.41 (m, 1H), 6.89 (dd, *J* = 12.7 Hz, 4.9 Hz, 2H), 6.78 (d, *J* = 7.6 Hz, 1H), 5.05 (d, *J* = 3.3 Hz, 1H), 4.40 (q, *J* = 9.6 Hz, 1H), 3.20 (ddt, *J* = 18.2 Hz, 9.7 Hz, 2.9 Hz, 1H), 2.77 (dddd, *J* = 18.2 Hz, 10.1 Hz, 4.1 Hz, 2.2 Hz, 1H); ^{13}C NMR (125 MHz, $CDCl_3$) δ 198.86, 158.83, 147.81, 142.27, 135.70, 134.05, 132.35, 130.71, 129.13, 128.71, 127.89, 125.57, 125.01, 111.19, 55.51, 44.30, 39.21; HRMS (ESI) m/z calcd for $C_{19}H_{13}BrNaO_3$ $[M+Na]^+$ = 390.9940, found = 390.9934. The ee value was 98%, t_R (major) = 18.11 min, t_R (minor) = 22.78 min (Chiralpak ID, λ = 254 nm, 45% *i*-PrOH/hexane, flow rate = 1.0 mL/min).

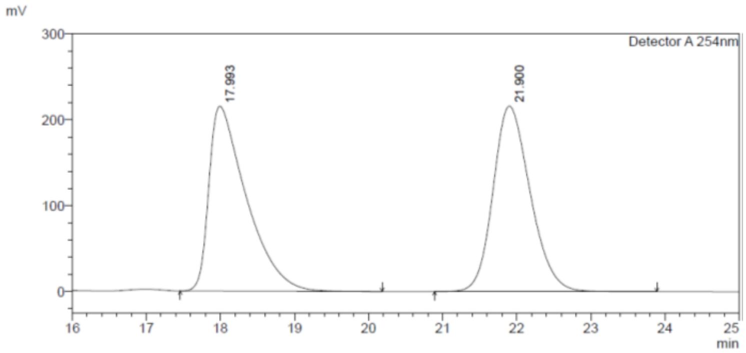

<Peak Table>

Detector A 254nm

Peak#	Ret. Time	Area	Height	Conc.	Unit	Mark	Name
1	17.993	7580170	215414	50.167			
2	21.900	7529763	216034	49.833			
Total		15109933	431448				

Racemic **2e**

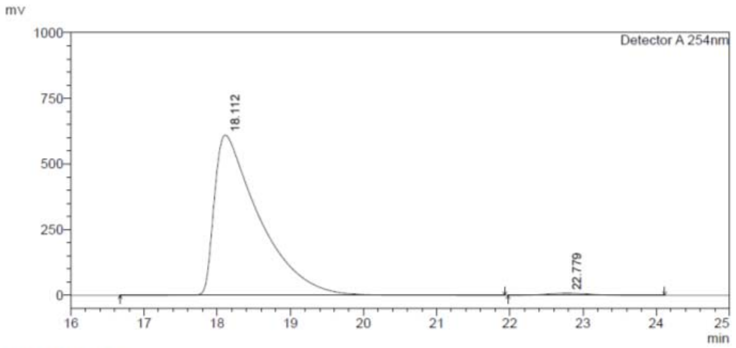

<Peak Table>

Detector A 254nm

Peak#	Ret. Time	Area	Height	Conc.	Unit	Mark	Name
1	18.112	25924611	610217	98.951			
2	22.779	274712	7382	1.049			
Total		26199323	617600				

Enantiomerically enriched **2e**

(1*R*,9b*R*)-1-Benzoyl-7-bromo-2,9b-dihydrocyclopenta[c]chromen-4(1*H*)-one **2f**

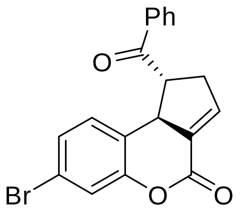

92% yield, a white solid, $[\alpha]^{25}_{D}$ = –155 (c 0.5, $CHCl_3$) . ^{1}H NMR (500 MHz, $CDCl_3$) δ 8.11 – 7.96 (m, 2H), 7.67 (t, *J* = 7.4 Hz, 1H), 7.56 (t, *J* = 7.7 Hz, 2H), 7.25 (d, *J* = 1.9 Hz, 1H), 7.16 (dd, *J* = 8.2 Hz, 1.8 Hz, 1H), 7.00 – 6.85 (m, 1H), 6.73 (dd, *J* = 8.2 Hz, 1.2 Hz, 1H), 5.01 – 4.90 (m, 1H), 4.36 (dd, *J* = 18.9 Hz, 9.7 Hz, 1H), 3.18 (ddt, *J* = 18.1 Hz, 9.6 Hz, 2.9 Hz, 1H), 2.76 (dddd, *J* = 18.1 Hz, 10.2 Hz, 4.1 Hz, 2.1 Hz, 1H); ^{13}C NMR (125 MHz, $CDCl_3$) δ 198.89, 159.35, 151.30, 142.24, 135.72, 134.06, 130.63, 129.13, 128.68, 127.91, 127.25, 125.11, 121.12, 120.52, 55.68, 43.67, 38.99; HRMS (ESI) m/z calcd for $C_{19}H_{13}BrNaO_3$ $[M+Na]^+$ = 390.9940, found = 390.9936. The ee value was 98%, t_R (major) = 19.34 min, t_R (minor) = 14.26 min (Chiralpak IC, λ = 254 nm, 45% *i*-PrOH/hexane, flow rate = 1.0 mL/min).

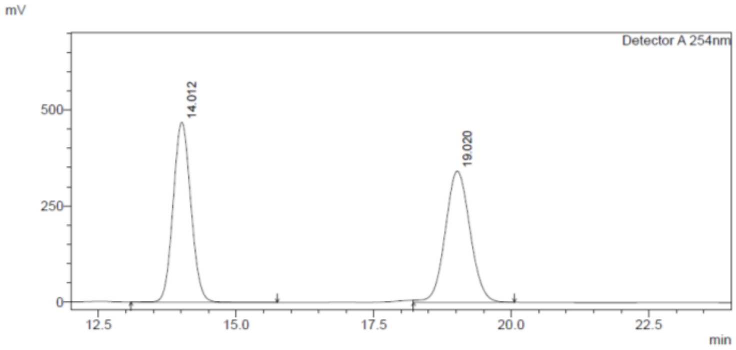

<Peak Table>

Detector A 254nm

Peak#	Ret. Time	Area	Height	Conc.	Unit	Mark	Name
1	14.012	10485029	468717	49.407			
2	19.020	10736839	341623	50.593		M	
Total		21221868	810341				

Racemic **2f**

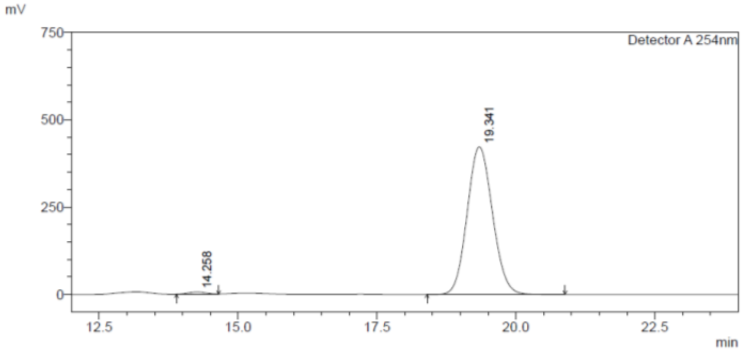

<Peak Table>

Detector A 254nm

Peak#	Ret. Time	Area	Height	Conc.	Unit	Mark	Name
1	14.258	145946	6434	1.076		M	
2	19.341	13412308	422832	98.924			
Total		13558254	429266				

Enantiomerically enriched **2f**

(1*R*,9b*R*)-1-Benzoyl-8-bromo-2,9b-dihydrocyclopenta[c]chromen-4(1*H*)-one **2g**

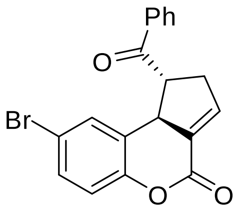

89% yield, a pale yellow oil, $[\alpha]^{25}_{D}$ = –125 (c 0.5, $CHCl_3$) . ^{1}H NMR (500 MHz, $CDCl_3$) δ 8.08 – 7.94 (m, 2H), 7.71 – 7.64 (m, 1H), 7.56 (dd, *J* = 10.7 Hz, 4.8 Hz, 2H), 7.34 (ddd, *J* = 8.7 Hz, 2.3 Hz, 0.9 Hz, 1H), 6.99 – 6.94 (m, 2H), 6.90 (dd, *J* = 5.3 Hz, 3.1 Hz, 1H), 5.19 – 4.89 (m, 1H), 4.37 (dd, *J* = 18.7 Hz, 9.8 Hz, 1H), 3.20 (ddt, *J* = 18.3 Hz, 9.7 Hz, 3.0 Hz, 1H), 2.75 (dddd, *J* = 18.3 Hz, 10.0 Hz, 4.1 Hz, 2.1 Hz, 1H); ^{13}C NMR (126 MHz, $CDCl_3$) δ 198.70, 159.49, 149.97, 142.18, 135.64, 134.06, 131.43, 130.48, 129.13, 128.84, 128.77, 128.19, 119.01, 117.52, 55.52, 43.64, 39.02; HRMS (ESI) m/z calcd for $C_{19}H_{13}BrNaO_3$ $[M+Na]^+$ = 390.9940, found = 390.9934. The ee value was 98%, t_R (major) = 14.23 min, t_R (minor) = 16.11 min (Chiralpak ID, λ = 254 nm, 45% *i*-PrOH/hexane, flow rate = 1.0 mL/min).

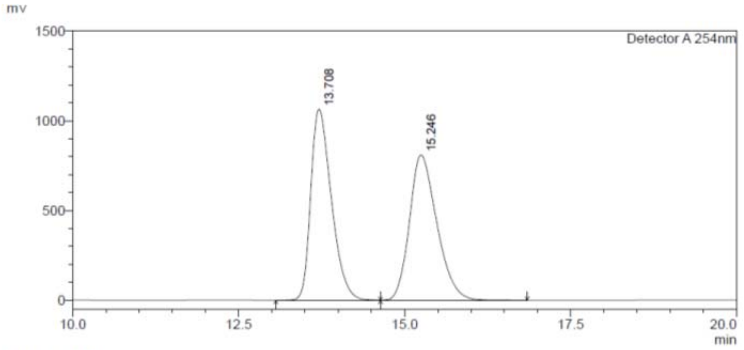

<Peak Table>

Detector A 254nm

Peak#	Ret. Time	Area	Height	Conc.	Unit	Mark	Name
1	13.708	23004336	1065407	49.706			
2	15.246	23276326	809524	50.294		V	
Total		46280663	1874932				

Racemic **2g**

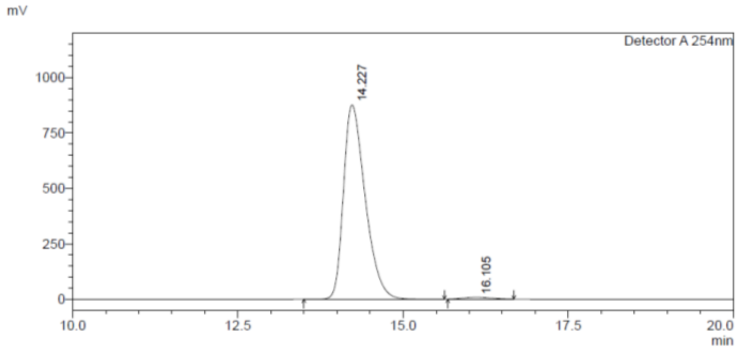

<Peak Table>

Detector A 254nm

Peak#	Ret. Time	Area	Height	Conc.	Unit	Mark	Name
1	14.227	20011754	876420	98.911		M	
2	16.105	220312	7831	1.089		M	
Total		20232065	884251				

Enantiomerically enriched **2g**

(1*R*,9b*R*)-1-Benzoyl-8-chloro-2,9b-dihydrocyclopenta[c]chromen-4(1*H*)-one **2h**

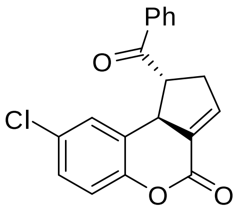

91% yield, a pale yellow solid, $[\alpha]^{25}_{D}$ = –156 (c 1.0, $CHCl_3$) . ^{1}H NMR (500 MHz, $CDCl_3$) δ 8.04 (d, *J* = 7.4 Hz, 2H), 7.68 (t, *J* = 7.4 Hz, 1H), 7.57 (t, *J* = 7.7 Hz, 2H), 7.20 (dd, *J* = 8.7 Hz, 1.5 Hz, 1H), 7.03 (d, *J* = 8.7 Hz, 1H), 6.90 (d, *J* = 2.2 Hz, 1H), 6.83 (s, 1H), 5.02 (d, *J* = 4.4 Hz, 1H), 4.38 (q, *J* = 9.6 Hz, 1H), 3.20 (ddt, *J* = 18.2 Hz, 9.7 Hz, 2.9 Hz, 1H), 2.76 (dddd, *J* = 18.1 Hz, 10.0 Hz, 4.0 Hz, 2.0 Hz, 1H); ^{13}C NMR (125 MHz, $CDCl_3$) δ 198.7, 159.5, 149.4, 142.1, 135.6, 134.1, 130.5, 130.0, 129.1, 128.8, 128.4, 127.8, 125.9, 118.6, 55.6, 43.7, 39.0; HRMS (ESI) m/z calcd for $C_{19}H_{13}ClNaO_3$ $[M+Na]^+$ = 347.0445, found = 347.0437. The ee value was 98%, t_R (major) = 15.08 min, t_R (minor) = 12.14 min (Chiralpak IC, λ = 254 nm, 45% *i*-PrOH/hexane, flow rate = 1.0 mL/min).

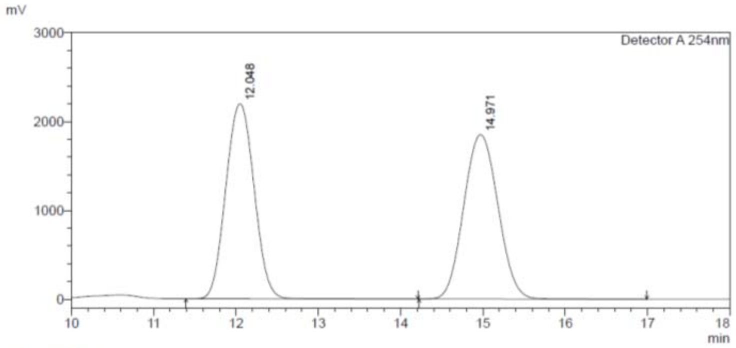

<Peak Table>

Detector A 254nm

Peak#	Ret. Time	Area	Height	Conc.	Unit	Mark	Name
1	12.048	52534430	2195977	49.540			
2	14.971	53508994	1849526	50.460			
Total		106043424	4045503				

Racemic **2h**

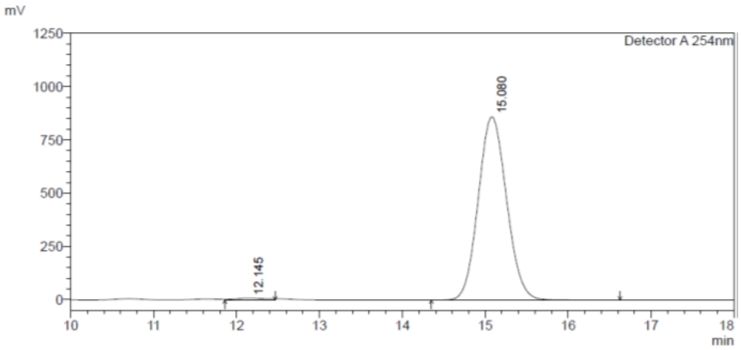

<Peak Table>

Detector A 254nm

Peak#	Ret. Time	Area	Height	Conc.	Unit	Mark	Name
1	12.145	230785	9065	1.118		M	
2	15.080	20411181	858997	98.882			
Total		20641966	868061				

Enantiomerically enriched **2h**

(1*R*,9b*R*)-1-Benzoyl-6,8-dichloro-2,9b-dihydrocyclopenta[c]chromen-4(1*H*)-one **2i**

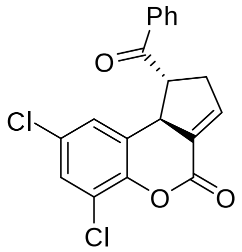

89% yield, a pale yellow solid, $[\alpha]^{25}_{D}$ = –189 (c 1.0, $CHCl_3$) . ^{1}H NMR (300 MHz, $CDCl_3$) δ 8.03 (d, *J* = 8.1 Hz, 2H), 7.68 (t, *J* = 7.3 Hz, 1H), 7.56 (t, *J* = 7.7 Hz, 2H), 7.33 – 7.27 (m, 1H), 7.01 – 6.86 (m, 1H), 6.80 – 6.67 (m, 1H), 5.03 (d, *J* = 5.3 Hz, 1H), 4.37 (q, *J* = 9.5 Hz, 1H), 3.24 (ddt, *J* = 18.4 Hz, 9.7 Hz, 2.8 Hz, 1H), 2.76 (dddd, *J* = 14.1 Hz, 9.9 Hz, 3.7 Hz, 2.1 Hz, 1H); ^{13}C NMR (75 MHz, $CDCl_3$) δ 198.34, 158.11, 145.53, 142.90, 135.38, 134.09, 129.72, 129.66, 129.07, 128.95, 128.87, 128.68, 124.25, 123.11, 55.40, 43.88, 39.04; HRMS (ESI) m/z calcd for $C_{19}H_{12}Cl_2NaO_3$ $[M+Na]^+$ = 381.0056, found = 381.0048. The ee value was 98%, t_R (major) = 13.24 min, t_R (minor) = 15.06 min (Chiralpak ID, λ = 254 nm, 45% *i*-PrOH/hexane, flow rate = 1.0 mL/min).

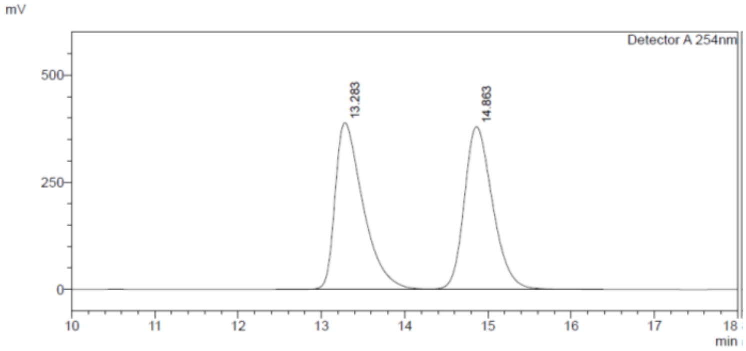

<Peak Table>

Detector A 254nm

Peak#	Ret. Time	Area	Height	Conc.	Unit	Mark	Name
1	13.283	9055396	389063	50.238		M	
2	14.863	8969639	379619	49.762		V M	
Total		18025035	768682				

Racemic **2i**

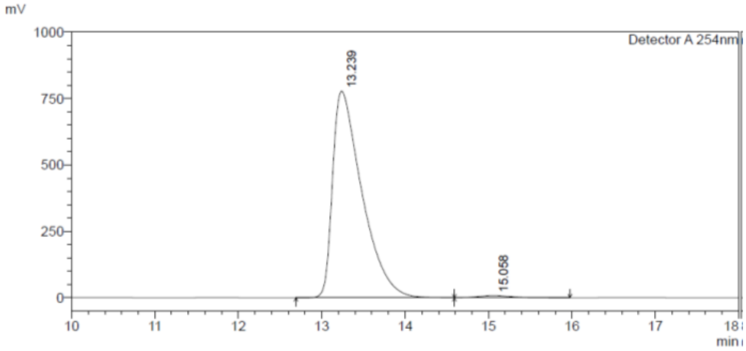

<Peak Table>

Detector A 254nm

Peak#	Ret. Time	Area	Height	Conc.	Unit	Mark	Name
1	13.239	19042779	778011	98.891			
2	15.058	213639	8157	1.109		V	
Total		19256417	786168				

Enantiomerically enriched **2i**

(1*R*,9b*R*)-1-(4-Methylbenzoyl)-2,9b-dihydrocyclopenta[c]chromen-4(1*H*)-one **2j**

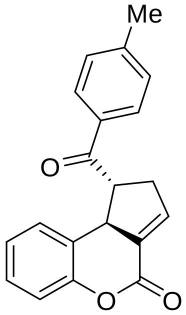

86% yield, a pale yellow solid, $[\alpha]^{25}_{D}$ = –170 (c 0.4, $CHCl_3$) . ^{1}H NMR (500 MHz, $CDCl_3$) δ 7.94 (d, *J* = 8.0 Hz, 2H), 7.34 (d, *J* = 8.0 Hz, 2H), 7.21 (t, *J* = 7.7 Hz, 1H), 7.06 (d, *J* = 8.1 Hz, 1H), 7.01 (t, *J* = 7.5 Hz, 1H), 6.88 – 6.81 (m, 2H), 5.14 – 4.86 (m, 1H), 4.38 (q, *J* = 9.5 Hz, 1H), 3.14 (ddt, *J* = 15.1 Hz, 9.1 Hz, 2.8 Hz, 1H), 2.73 (dddd, *J* = 14.4 Hz, 10.1 Hz, 3.9 Hz, 1.8 Hz, 1H), 2.45 (s, 3H); ^{13}C NMR (125 MHz, $CDCl_3$) δ 198.80, 160.23, 150.84, 144.99, 141.65, 133.39, 131.37, 129.77, 128.84, 128.32, 126.14, 126.01, 124.90, 117.20, 55.47, 44.01, 39.13, 21.74; HRMS (ESI) m/z calcd for $C_{20}H_{16}NaO_3$ $[M+Na]^+$ = 327.0992, found = 327.0987. The ee value was 98%, t_R (major) = 15.76 min, t_R (minor) = 22.32 min (Chiralpak ID, λ = 254 nm, 45% *i*-PrOH/hexane, flow rate = 1.0 mL/min).

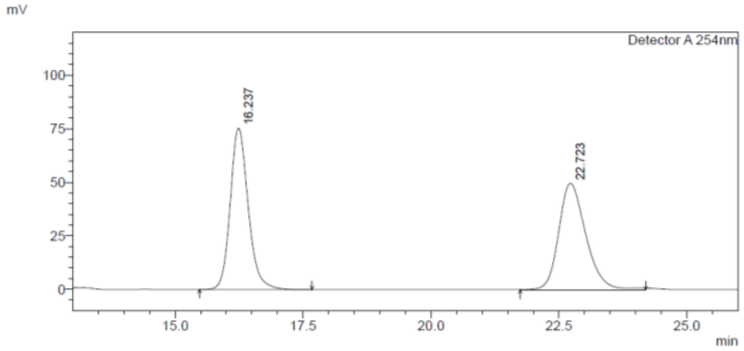

<Peak Table>

Detector A 254nm

Peak#	Ret. Time	Area	Height	Conc.	Unit	Mark	Name
1	16.237	1844829	75425	50.012			
2	22.723	1843953	49774	49.988		M	
Total		3688782	125198				

Racemic **2j**

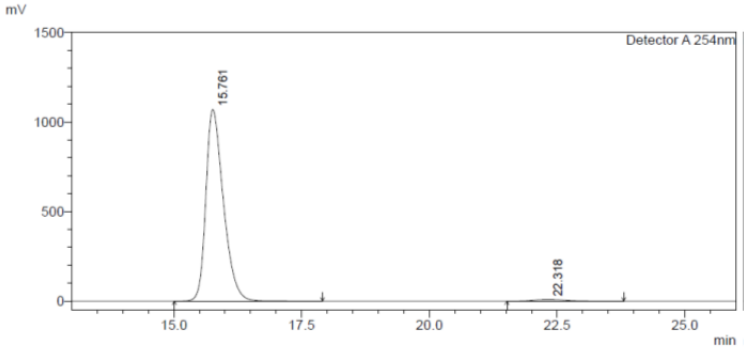

<Peak Table>

Detector A 254nm

Peak#	Ret. Time	Area	Height	Conc.	Unit	Mark	Name
1	15.761	25480967	1070010	98.788			
2	22.318	312686	8372	1.212			
Total		25793654	1078382				

Enantiomerically enriched **2j**

(1*R*,9b*R*)-1-(Thiophene-2-carbonyl)-2,9b-dihydrocyclopenta[c]chromen-4(1*H*)-one **2k**

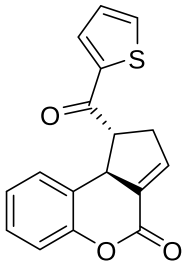

87% yield, a pale yellow solid, $[\alpha]^{25}_{D}$ = –125 (c 1.0, $CHCl_3$). ^{1}H NMR (500 MHz, $CDCl_3$) δ 8.17 (dd, *J* = 2.8 Hz, 1.0 Hz, 1H), 7.66 (dd, *J* = 5.2 Hz, 1.0 Hz, 1H), 7.43 (dd, *J* = 5.1 Hz, 2.9 Hz, 1H), 7.23 (dd, *J* = 8.1 Hz, 7.5 Hz, 1H), 7.08 (d, *J* = 8.1 Hz, 1H), 7.04 (t, *J* = 7.6 Hz, 1H), 6.89 (dd, *J* = 8.7 Hz, 5.5 Hz, 2H), 4.97 (dd, *J* = 8.2 Hz, 3.6 Hz, 1H), 4.22 (dd, *J* = 18.9 Hz, 9.6 Hz, 1H), 3.15 (ddt, *J* = 18.1 Hz, 9.6 Hz, 3.0 Hz, 1H), 2.82 (dddd, *J* = 18.1 Hz, 10.1 Hz, 4.1 Hz, 2.1 Hz, 1H); ^{13}C NMR (125 MHz, $CDCl_3$) δ 193.34, 160.14, 150.83, 141.70, 141.21, 133.18, 131.33, 128.41, 127.27, 127.21, 125.97, 124.93, 117.28, 57.01, 43.98, 39.15; HRMS (ESI) m/z calcd for $C_{17}H_{12}NaO_3S$ $[M+Na]^+$ = 319.0399, found = 319.0392. The ee value was 98%, t_R (major) = 16.07 min, t_R (minor) = 22.47 min (Chiralpak ID, λ = 254 nm, 45% *i*-PrOH/hexane, flow rate = 1.0 mL/min).

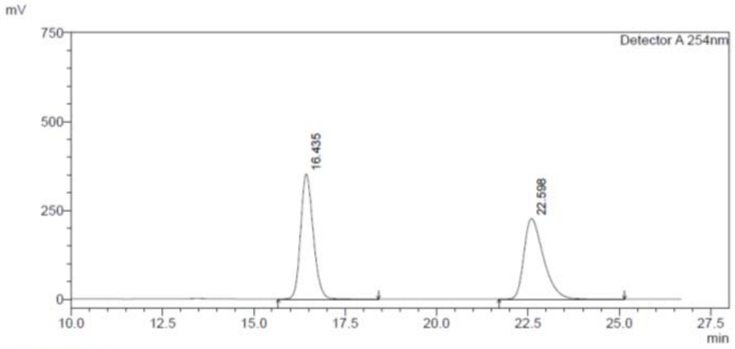

<Peak Table>

Detector A 254nm

Peak#	Ret. Time	Area	Height	Conc.	Unit	Mark	Name
1	16.435	8416752	351912	49.985			
2	22.598	8421959	226438	50.015			
Total		16838711	578350				

Racemic **2k**

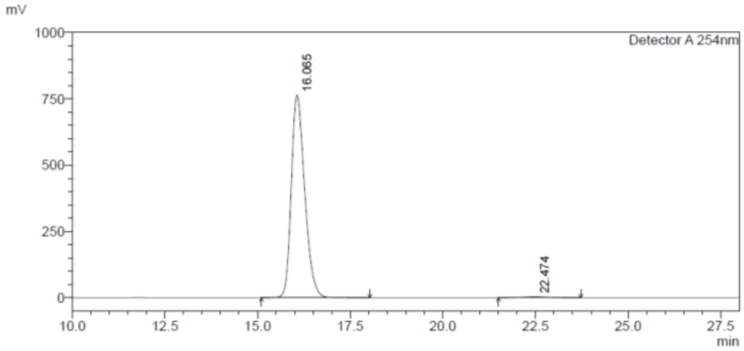

<Peak Table>

Detector A 254nm

Peak#	Ret. Time	Area	Height	Conc.	Unit	Mark	Name
1	16.065	19253446	764362	99.184			
2	22.474	158456	3483	0.816			
Total		19411903	767845				

Enantiomerically enriched **2k**

(1*R*,9b*R*)-1-(4-Fluorobenzoyl)-2,9b-dihydrocyclopenta[c]chromen-4(1*H*)-one **2l**

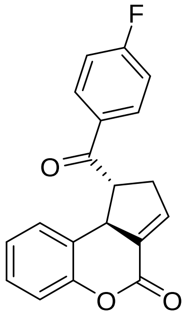

86% yield, a pale yellow solid, $[\alpha]^{25}_{D}$ = –181 (c 1.0, $CHCl_3$). ^{1}H NMR (500 MHz, $CDCl_3$) δ 8.14 – 8.02 (m, 2H), 7.25 – 7.20 (m, 3H), 7.09 (d, *J* = 8.1 Hz, 1H), 7.04 (t, *J* = 7.5 Hz, 1H), 6.88 (dd, *J* = 5.2 Hz, 3.0 Hz, 1H), 6.85 – 6.81 (m, 1H), 5.02 (dd, *J* = 7.9 Hz, 3.3 Hz, 1H), 4.36 (dd, *J* = 18.8 Hz, 9.7 Hz, 1H), 3.15 (ddt, *J* = 18.0 Hz, 9.6 Hz, 3.0 Hz, 1H), 2.76 (dddd, *J* = 17.9 Hz, 10.2 Hz, 4.1 Hz, 2.1 Hz, 1H); ^{13}C NMR (125MHz, $CDCl_3$) δ 197.59, 166.24 (d, *J* = 256.5 Hz), 160.10, 150.85, 141.34, 132.30 (d, *J* = 3.0 Hz), 131.39 (d, *J* = 9.5 Hz), 128.45, 125.91, 125.85, 124.94, 117.33, 116.29 (d, *J* = 22.0 Hz), 55.52, 44.07, 39.00; HRMS (ESI) m/z calcd for $C_{19}H_{13}FNaO_3$ $[M+Na]^+$ = 331.0741, found = 331.0732. The ee value was 99%, t_R (major) = 13.49 min, t_R (minor) = 18.90 min (Chiralpak ID, λ = 254 nm, 45% *i*-PrOH/hexane, flow rate = 1.0 mL/min).

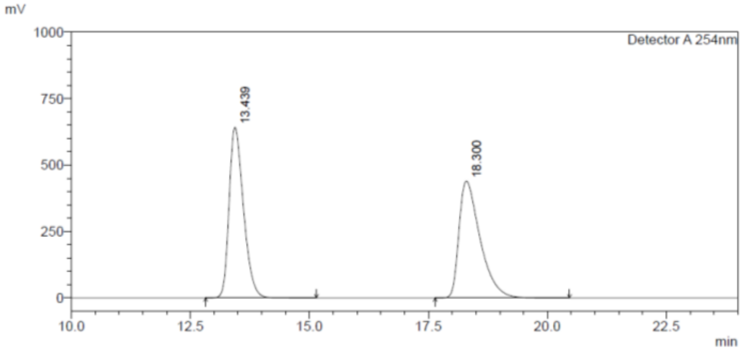

<Peak Table>
Detector A 254nm

Peak#	Ret. Time	Area	Height	Conc.	Unit	Mark	Name
1	13.439	13487617	642139	49.856			
2	18.300	13565478	439991	50.144			
Total		27053095	1082131				

Racemic **2l**

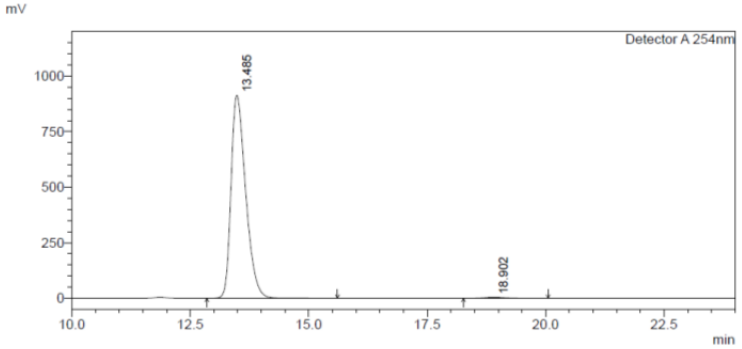

<Peak Table>
Detector A 254nm

Peak#	Ret. Time	Area	Height	Conc.	Unit	Mark	Name
1	13.485	19617880	914248	99.448		S	
2	18.902	108851	3516	0.552			
Total		19726731	917764				

Enantiomerically enriched **2l**

(1*R*,9b*R*)-1-(3-Bromobenzoyl)-2,9b-dihydrocyclopenta[c]chromen-4(1*H*)-one **2m**

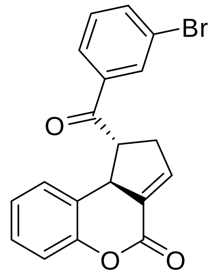

87% yield, a white solid, $[\alpha]^{25}_{D}$ = –173 (c 1.0, $CHCl_3$). ^{1}H NMR (500 MHz, $CDCl_3$) δ 8.17 (t, *J* = 1.8 Hz, 1H), 7.99 – 7.92 (m, 1H), 7.78 (ddd, *J* = 8.0 Hz, 1.9 Hz, 0.9 Hz, 1H), 7.44 (t, *J* = 7.9 Hz, 1H), 7.26 – 7.20 (m, 1H), 7.09 (dd, *J* = 8.2 Hz, 0.9 Hz, 1H), 7.04 (td, *J* = 7.5 Hz, 1.1 Hz, 1H), 6.88 (dd, *J* = 5.4 Hz, 3.2 Hz, 1H), 6.82 (dt, *J* = 7.7 Hz, 1.3 Hz, 1H), 5.01 (dd, *J* = 8.1 Hz, 3.6 Hz, 1H), 4.34 (dd, *J* = 18.8 Hz, 9.8 Hz, 1H), 3.16 (ddt, *J* = 18.1 Hz, 9.6 Hz, 3.0 Hz, 1H), 2.75 (dddd, *J* = 18.0 Hz, 10.1 Hz, 4.1 Hz, 2.1 Hz, 1H); ^{13}C NMR (125 MHz, $CDCl_3$) δ 197.93, 160.04, 150.84, 141.27, 137.60, 136.78, 131.73, 131.39, 130.65, 128.51, 127.14, 125.84, 125.76, 124.97, 123.51, 117.35, 55.62, 44.00, 38.89. HRMS (ESI) m/z calcd for $C_{19}H_{13}BrNaO_3$ $[M+Na]^+$ = 390.9940, found = 390.9937. The ee value was 99.5%, t_R (major) = 16.61 min, t_R (minor) = 25.84 min (Chiralpak ID, λ = 254 nm, 45% *i*-PrOH/hexane, flow rate = 1.0 mL/min).

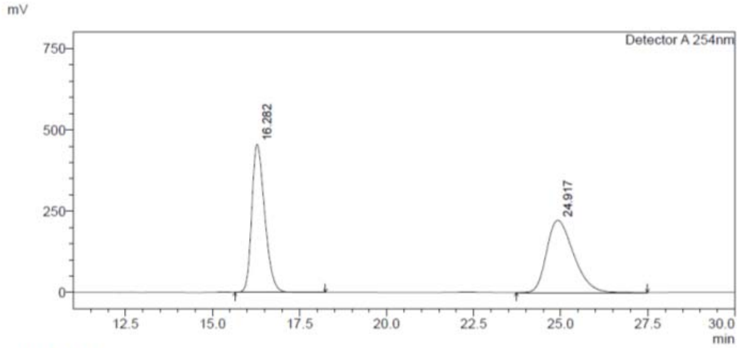

<Peak Table>

Detector A 254nm

Peak#	Ret. Time	Area	Height	Conc.	Unit	Mark	Name
1	16.282	11774521	455303	49.679			
2	24.917	11926577	221956	50.321		S	
Total		23701098	677259				

Racemic **2m**

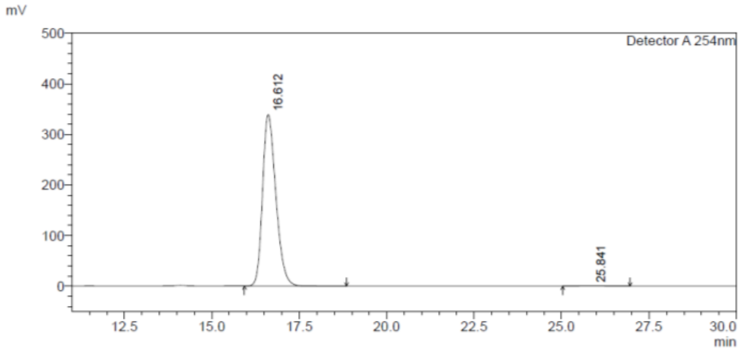

<Peak Table>

Detector A 254nm

Peak#	Ret. Time	Area	Height	Conc.	Unit	Mark	Name
1	16.612	9002656	339172	99.738			
2	25.841	23654	467	0.262			
Total		9026310	339639				

Enantiomerically enriched **2m**

(1*R*,9b*R*)-1-(2-Iodobenzoyl)-2,9b-dihydrocyclopenta[c]chromen-4(1*H*)-one **2n**

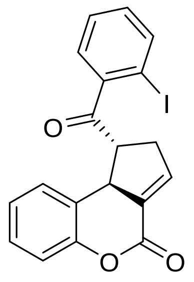

85% yield, a pale yellow solid, $[\alpha]^{25}_{D}$ = –70 (c 1.0, $CHCl_3$). ^{1}H NMR (500 MHz, $CDCl_3$) δ 8.01 (d, *J* = 8.0 Hz, 1H), 7.52 – 7.44 (m, 2H), 7.29 – 7.19 (m, 2H), 7.12 – 7.07 (m, 2H), 7.05 (dd, *J* = 7.9 Hz, 1.3 Hz, 1H), 6.90 – 6.84 (m, 1H), 4.83 (dd, *J* = 7.7 Hz, 3.1 Hz, 1H), 4.36 (q, *J* = 9.7 Hz, 1H), 3.01 – 2.85 (m, 2H); ^{13}C NMR (125 MHz, $CDCl_3$) δ 203.21, 160.16, 150.88, 143.40, 141.90, 141.20, 132.36, 130.96, 128.52, 128.34, 128.32, 126.29, 125.85, 124.99, 117.31, 91.68, 58.07, 44.31, 37.33; HRMS (ESI) m/z calcd for $C_{19}H_{13}INaO_3$ $[M+Na]^+$ = 438.9802, found = 438.9811. The ee value was 98%, t_R (major) = 18.36 min, t_R (minor) = 26.19 min (Chiralpak ID, λ = 254 nm, 45% *i*-PrOH/hexane, flow rate = 1.0 mL/min).

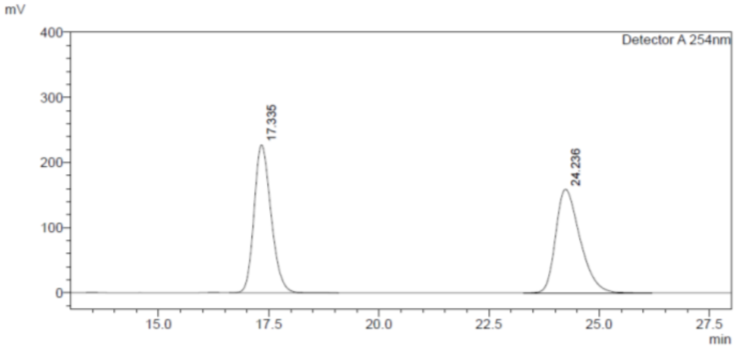

<Peak Table>

Detector A 254nm

Peak#	Ret. Time	Area	Height	Conc.	Unit	Mark	Name
1	17.335	6110134	227252	49.933			
2	24.236	6126482	159191	50.067			
Total		12236616	386443				

Racemic **2n**

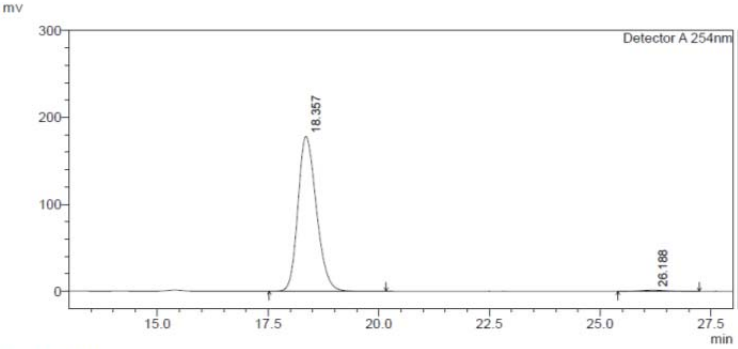

<Peak Table>

Detector A 254nm

Peak#	Ret. Time	Area	Height	Conc.	Unit	Mark	Name
1	18.357	5158130	178237	99.041			
2	26.188	49968	1224	0.959			
Total		5208098	179461				

Enantiomerically enriched **2n**

F Intramolecular Heck reaction of 2n

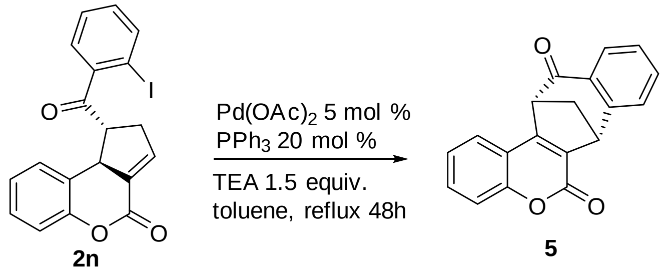

2n 98% ee   **5** 76% yield, 98% ee

To the solution of **2n** (0.1 mmol, 41.6 mg) in toluene (2 mL) under the argon was added $Pd(OAc)_2$ (5 mol %, 1.1 mg), PPh_3 (20 mol%, 5.2 mg) and triethylamine (0.15 mmol, 21 μL). The mixture was heated to reflux for 48 h, and was then cooled to room temperature and directly purified by column chromatography on silica gel to afford product **5** (21.9 mg, 76% yield) as a white solid, $[\alpha]^{25}_{D} = +229$ (c 0.5, $CHCl_3$); ^{1}H NMR (500 MHz, $CDCl_3$) δ 7.90 (d, *J* = 7.8 Hz, 1H), 7.72 (d, *J* = 7.8 Hz, 1H), 7.51 (t, *J* = 7.9 Hz, 1H), 7.44 (p, *J* = 7.5 Hz, 2H), 7.36 – 7.29 (m, 3H), 4.34 (dd, *J* = 15.4 Hz, 4.4 Hz, 2H), 3.08 (dt, J = 11.1 Hz, 4.5 Hz, 1H), 3.00 (d, *J* = 11.1 Hz, 1H); ^{13}C NMR (125 MHz, $CDCl_3$) δ 192.46, 158.76, 154.81, 154.53, 146.02, 137.03, 133.98, 132.01, 128.76, 128.24, 128.22, 126.16, 125.42, 124.65, 117.47, 117.09, 56.47, 47.92, 45.05. HRMS (ESI) m/z calcd for $C_{19}H_{12}NaO_3$ $[M+Na]^+$ = 311.0679, found = 311.0675. The ee value was 98%, t_R (major) = 19.68 min, t_R (minor) = 12.27 min (Chiralpak ID, λ = 254 nm, 45% *i*-PrOH/hexane, flow rate = 1.0 mL/min).

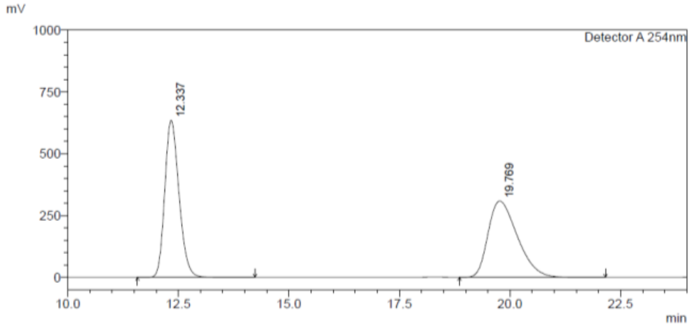

<Peak Table>
Detector A 254nm

Peak#	Ret. Time	Area	Height	Conc.	Unit	Mark	Name
1	12.337	14224798	635276	49.971			
2	19.769	14241215	308801	50.029			
Total		28466014	944077				

Racemic **5**

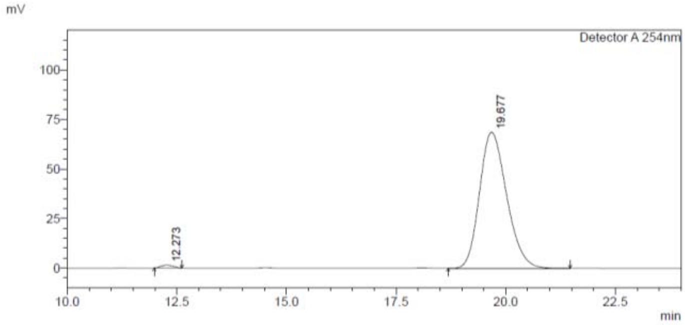

<Peak Table>
Detector A 254nm

Peak#	Ret. Time	Area	Height	Conc.	Unit	Mark	Name
1	12.273	28422	1480	0.952		M	
2	19.677	2957268	68736	99.048			
Total		2985691	70216				

Enantiomerically enriched **5**

G X-Ray crystallographic analysis and determination of the absolute configurations of the products

X-Ray Crystallographic Analysis of **2a**

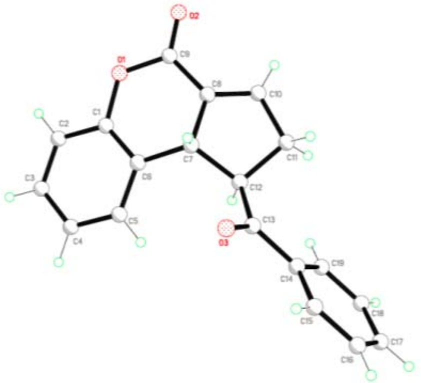

Figure S1. X-ray structure of **2a**

Table 1. Crystal data and structure refinement for **2a**.

Identification code	F626	
Empirical formula	C19 H14 O3	
Formula weight	290.30	
Temperature	100(2) K	
Wavelength	1.54178 Å	
Crystal system	Orthorhombic	
Space group	$P2_12_12_1$	
Unit cell dimensions	a = 7.8666(2) Å	α= 90°.
	b = 10.3517(2) Å	β= 90°.
	c = 17.8836(4) Å	γ = 90°.
Volume	1456.31(6) Å^3	
Z	4	
Density (calculated)	1.324 Mg/m^3	
Absorption coefficient	0.722 mm^{-1}	
F(000)	608	
Crystal size	0.322 x 0.291 x 0.271 mm^3	
Theta range for data collection	4.936 to 70.039°.	
Index ranges	-9<=h<=9, -12<=k<=12, -21<=l<=21	
Reflections collected	25564	
Independent reflections	2762 [R(int) = 0.0353]	
Completeness to theta = 67.679°	100.0 %	
Absorption correction	Semi-empirical from equivalents	
Max. and min. transmission	0.7536 and 0.6717	
Refinement method	Full-matrix least-squares on F^2	
Data / restraints / parameters	2762 / 0 / 200	
Goodness-of-fit on F^2	1.065	

Final R indices [I>2sigma(I)]	R1 = 0.0306, wR2 = 0.0767
R indices (all data)	R1 = 0.0331, wR2 = 0.0789
Absolute structure parameter	-0.03(6)
Extinction coefficient	0.0079(7)
Largest diff. peak and hole	0.153 and -0.146 e.Å^{-3}

X-Ray Crystallographic Analysis of **5**

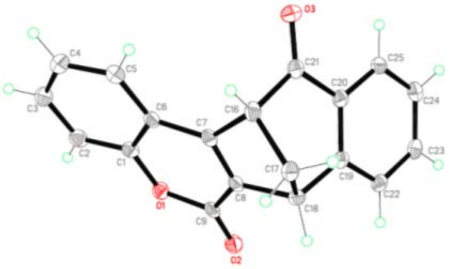

Figure S2. X-ray structure of **5**

Table 2. Crystal data and structure refinement for **5**.

Identification code	G180	
Empirical formula	C19 H12 O3	
Formula weight	288.29	
Temperature	100(2) K	
Wavelength	1.54178 Å	
Crystal system	Orthorhombic	
Space group	$P2_12_12_1$	
Unit cell dimensions	a = 6.4062(2) Å	α= 90°.
	b = 11.3591(3) Å	β= 90°.
	c = 18.7239(4) Å	γ = 90°.
Volume	1362.51(6) Å^3	
Z	4	
Density (calculated)	1.405 Mg/m^3	
Absorption coefficient	0.772 mm^{-1}	
F(000)	600	
Crystal size	0.169 x 0.082 x 0.044 mm^3	
Theta range for data collection	4.553 to 74.415°.	
Index ranges	-7<=h<=7, -13<=k<=14, -23<=l<=23	
Reflections collected	24033	
Independent reflections	2761 [R(int) = 0.0326]	
Completeness to theta = 67.679°	100.0 %	
Absorption correction	Semi-empirical from equivalents	
Max. and min. transmission	0.7528 and 0.6718	
Refinement method	Full-matrix least-squares on F^2	

Data / restraints / parameters	2761 / 0 / 199
Goodness-of-fit on F^2	1.060
Final R indices [I>2sigma(I)]	R1 = 0.0279, wR2 = 0.0681
R indices (all data)	R1 = 0.0297, wR2 = 0.0692
Absolute structure parameter	0.09(5)
Extinction coefficient	n/a
Largest diff. peak and hole	0.133 and -0.173 e.Å^{-3}

H. DFT studies

a). Computational methods.

Density functional theory (DFT) methods, as implemented in the Gaussian 09[1] program, have been employed to study the origin of enantioselectivity and effects of benzoic acid in the phosphine catalyzed [3+2] cyclization. All the stationary points were optimized at the B3LYP[2]/6-31G(d)[3] level of theory. The vibrational frequencies were computed at the same level of theory to determine whether the optimized structure was at an energy minimum or a transition state and to evaluate the corrections of enthalpy and Gibbs free energy. Solvent effects were computed by the CPCM[4] solvation model at the M11[5]/6-311+G(d)[6] levels of theory using the gas optimized structure, and the M11 calculated Gibbs free energies in toluene are discussed in the text.

b). B3LYP and M11 absolute calculation energies, enthalpies, and free energies

Geometry	$E_{(elec\text{-}B3LYP)}$[1]	$H_{(corr\text{-}B3LYP)}$[2]	$G_{(corr\text{-}B3LYP)}$[3]	$E_{(solv\text{-}M11)}$[4]	IF[5]
**3f**	-2273.746004	0.794275	0.668854	-2273.187437	-
**1a**	-958.031620	0.296948	0.225227	-957.757564	-
**2a-*re***	-958.102046	0.300557	0.236133	-957.849648	-
**2a-*si***	-958.102046	0.300557	0.236133	-957.849648	-
**6-ts**	-3231.765455	1.091917	0.916998	-3230.938259	-226.3
**7-int**	-3231.800352	1.094307	0.921376	-3230.980749	-
**8-ts-*re***	-3231.788865	1.094291	0.931154	-3230.988145	-320.6
**8-ts-*si***	-3231.787464	1.094057	0.930903	-3230.986242	-245.6
**9-int-*re***	-3231.795023	1.095621	0.931382	-3231.015350	-
**9-int-*si***	-3231.800564	1.095954	0.932421	-3231.024889	-
**10-ts-*re***	-3231.789787	1.094870	0.931789	-3231.004794	-265.1
**10-ts-*si***	-3231.789046	1.095140	0.932968	-3231.006327	-321.5
**11-int-*re***	-3231.812698	1.097421	0.931236	-3231.022241	-
**11-int-*si***	-3231.816526	1.097280	0.929187	-3231.024353	-
**12**	-420.8140895	0.124021	0.084024	-420.7252534	-
**13-int**	-2694.571449	0.920306	0.769591	-2693.917645	-
**14-ts**	-3652.602632	1.218219	1.023153	-3651.679128	-219.6
**15-int**	-3652.639764	1.220642	1.028174	-3651.726280	-
**16-ts-*re***	-3652.621503	1.220305	1.033837	-3651.718768	-307.8
**16-ts-*si***	-3652.614695	1.219974	1.032745	-3651.712903	-325.5

**17-int-*re***	-3652.630232	1.222306	1.036023	-3651.752375	-
**17-int-*si***	-3652.621137	1.222075	1.035720	-3651.738925	-
**18-ts-*re***	-3652.622974	1.221467	1.035879	-3651.738115	-293.8
**18-ts-*si***	-3652.615454	1.221371	1.036296	-3651.727205	-268.7
**19-int-*re***	-3652.649377	1.223828	1.035821	-3651.763840	-
**19-int-*si***	-3652.647929	1.223331	1.033616	-3651.760346	-

[1]The electronic energy calculated by B3LYP in gas phase. [2]The thermal correction to enthalpy calculated by B3LYP in gas phase. [3]The thermal correction to Gibbs free energy calculated by B3LYP in gas phase. [4]The electronic energy calculated by M11 in toluene. [5]The B3LYP calculated imaginary frequencies for the transition states.

c). B3LYP geometries for all the optimized compounds and transition states

3f

P	0.11347600	0.38816800	1.68384600
C	0.96781000	0.41651300	0.00226900
H	2.03315400	0.57493200	0.19533000
H	0.62730000	1.27110800	-0.59484200
C	0.84041300	-0.89688000	-0.77615500
H	1.12723300	-1.72250700	-0.11976500
C	1.78574600	-0.96922900	-2.01061100
H	1.53998200	-1.90409400	-2.52411600
C	1.61937600	0.18954400	-2.99290500
H	1.93700800	1.13585800	-2.54290600
H	0.57543200	0.28323700	-3.31333200
H	2.23477100	0.01939600	-3.88167700
C	0.90582400	1.88060600	2.45388300
C	0.18950300	2.98762000	2.93366800
C	2.29579300	1.84429400	2.67042300
C	0.84479600	4.03628500	3.58462600
H	-0.88617100	3.03875800	2.79891800
C	2.95063200	2.89540700	3.31060500
H	2.87568600	0.98379800	2.34362100
C	2.22671200	3.99877400	3.76947100
H	0.26956400	4.88572200	3.94467000
H	4.02661800	2.84840500	3.45723000
H	2.73533400	4.81677700	4.27258800
C	-1.59066400	0.99906400	1.26814900
C	-2.67576400	0.32150500	1.84719900
C	-1.86240600	2.07934300	0.40922700
C	-3.99234700	0.70664600	1.57831500
H	-2.48279800	-0.51509200	2.51407900
C	-3.17618800	2.46296300	0.13380000
H	-1.04344800	2.63977000	-0.03468500

C	-4.24463900	1.77652700	0.71811000
H	-4.81769800	0.17047700	2.03922300
H	-3.36645200	3.30125800	-0.53157200
H	-5.26705300	2.07681500	0.50481600
O	3.13742900	-0.99193700	-1.56442000
Si	4.25861500	-2.22833200	-1.35940900
C	3.96610600	-3.10662300	0.29511800
C	2.77210000	-3.81908500	0.54112000
C	4.90776600	-3.04509400	1.34034500
C	2.54072300	-4.44620100	1.76679400
H	1.99603500	-3.87590500	-0.21738000
C	4.68245000	-3.67757000	2.56555200
H	5.82997200	-2.48790000	1.20428400
C	3.49831500	-4.38291400	2.78108100
H	1.60874100	-4.98197900	1.92774000
H	5.43010900	-3.61264500	3.35246000
H	3.31893900	-4.87320700	3.73474500
C	5.88674900	-1.26116200	-1.31473500
C	5.89164400	0.11580500	-1.60784500
C	7.12699700	-1.86058600	-1.01503300
C	7.07457200	0.85848700	-1.60534000
H	4.95154800	0.60843900	-1.83560100
C	8.31286300	-1.12339200	-1.00921100
H	7.17649100	-2.92001400	-0.77676000
C	8.28963000	0.24064900	-1.30616700
H	7.04648100	1.92072000	-1.83641000
H	9.25365900	-1.61430700	-0.77259400
H	9.21162000	0.81670600	-1.30322100
C	4.24696800	-3.46247400	-2.84730300
C	3.05394300	-4.44529100	-2.81558900
H	3.07657400	-5.07877400	-3.71483000
H	2.07606200	-3.95283900	-2.78819700
H	3.10629000	-5.11171600	-1.94822200
C	5.54272200	-4.30744900	-2.82081100
H	5.52015700	-5.04272700	-3.63814900
H	5.64943800	-4.86897300	-1.88383100
H	6.43951300	-3.69419300	-2.95604400
C	4.21593600	-2.66210700	-4.16846000
H	5.03196800	-1.93164600	-4.22451400
H	3.27231900	-2.12135400	-4.30035600
H	4.32290200	-3.34386600	-5.02479000
N	-0.54410800	-1.14639100	-1.18368900
H	-1.18310100	-0.36676400	-1.23053800
C	-0.99654800	-2.40652700	-1.44546100
O	-0.25261400	-3.38682000	-1.37165400
C	-2.48727100	-2.58394300	-1.81961700
C	-3.23845700	-1.26380100	-2.06828200

H	-2.77957500	-0.67965100	-2.87573500
H	-4.26903600	-1.48037400	-2.37207500
H	-3.29817600	-0.63909800	-1.16920400
C	-2.53694300	-3.45328600	-3.09349100
H	-1.96821300	-4.37474200	-2.94825600
H	-3.57488600	-3.70946600	-3.33626300
H	-2.11074400	-2.92161300	-3.95314200
C	-3.14724000	-3.34582600	-0.64798600
H	-4.19134800	-3.57938600	-0.88853700
H	-2.61493100	-4.28102300	-0.45245300
H	-3.13643300	-2.74537900	0.26944900

1a

C	-1.96219000	1.11295200	0.33497000
C	-0.65024500	1.43095600	-0.00608200
C	-0.12291700	2.72531500	0.17346200
C	-1.00857600	3.70014700	0.68237200
C	-2.31241300	3.38855700	1.05711700
C	-2.79543400	2.09230600	0.88115000
H	-2.32866800	0.10093500	0.18974100
H	0.00198400	0.66263100	-0.41305500
H	-2.93536200	4.17218300	1.47676700
H	-3.81449700	1.85109000	1.16951000
C	1.27453600	2.95543700	-0.19229900
H	1.74743400	2.13348100	-0.72935400
C	2.06746300	4.01748700	0.06001400
H	1.71232600	4.86639100	0.62556700
C	3.48088100	4.00627700	-0.40526600
O	3.96592800	3.01142500	-0.94224500
C	4.31464800	5.23563000	-0.19725000
C	3.77823000	6.48507800	0.15737400
C	5.70138500	5.11990100	-0.39430700
C	4.61672600	7.58963600	0.31565300
H	2.70960700	6.62008000	0.29085100
C	6.53588400	6.21974700	-0.22529400
H	6.09767000	4.15099300	-0.67972100
C	5.99412300	7.45922700	0.13131200
H	4.19014500	8.55251200	0.58260600
H	7.60765100	6.11558200	-0.37214000
H	6.64427700	8.32044400	0.26200000
O	-0.55032500	4.98913100	0.91728100
C	-0.67356400	6.03716700	0.03742900
O	-0.14414600	7.08159600	0.33964200
C	-1.43608800	5.90606300	-1.23224300
H	-1.56995500	6.87602200	-1.70805000
C	-1.86911900	4.83254300	-1.85728700
C	-2.31805800	3.83651600	-2.56884000

H	-1.68468600	3.33179300	-3.29516400
H	-3.32716700	3.45336800	-2.43541900

2a-*re*

C	-12.82507100	11.59500700	4.05925900
C	-11.43736300	11.50647900	4.17413100
C	-10.60748000	11.62681900	3.05436600
C	-11.21350800	11.84787500	1.80910900
C	-12.59876800	11.94115800	1.67790600
C	-13.40485800	11.81378800	2.80756700
H	-13.44869000	11.49874600	4.94324700
H	-10.98702200	11.35305700	5.15116800
H	-13.01967600	12.11000400	0.69208000
H	-14.48414900	11.88571800	2.70777600
C	-9.09871400	11.58229700	3.11078900
H	-8.73226100	12.58240100	3.39070200
C	-8.43518000	10.55386500	4.06503300
H	-9.10136800	9.68920900	4.16019900
C	-8.10629000	11.11307400	5.44977400
O	-8.05059100	12.32189900	5.63146900
C	-7.81025200	10.16373700	6.57166000
C	-7.89124800	8.76784600	6.44175100
C	-7.43391100	10.71170600	7.81018800
C	-7.60063800	7.93945900	7.52599800
H	-8.18254400	8.31667600	5.49866900
C	-7.14476800	9.88522600	8.89102400
H	-7.37619900	11.79173500	7.89607800
C	-7.22761100	8.49592200	8.75082200
H	-7.66732100	6.86087600	7.41452300
H	-6.85472600	10.31979600	9.84363800
H	-7.00239600	7.84936700	9.59471500
O	-10.49087300	12.01280500	0.63033900
C	-9.17574300	11.61922800	0.50666000
O	-8.66395900	11.63393200	-0.58668800
C	-8.53527200	11.19264100	1.76285900
C	-7.45962300	10.40316500	1.86382900
C	-7.14191800	10.09176400	3.30403600
H	-6.91148000	9.03351100	3.47176200
H	-6.25816000	10.65806600	3.63513900
H	-6.88026300	10.03582500	1.02283800

2a-*si*

C	-12.82507100	11.59500700	-4.05925900
C	-11.43736300	11.50647900	-4.17413100
C	-10.60748000	11.62681900	-3.05436600
C	-11.21350800	11.84787500	-1.80910900
C	-12.59876800	11.94115800	-1.67790600

C	-13.40485800	11.81378800	-2.80756700
H	-13.44869000	11.49874600	-4.94324700
H	-10.98702200	11.35305700	-5.15116800
H	-13.01967600	12.11000400	-0.69208000
H	-14.48414900	11.88571800	-2.70777600
C	-9.09871400	11.58229700	-3.11078900
H	-8.73226100	12.58240100	-3.39070200
C	-8.43518000	10.55386500	-4.06503300
H	-9.10136800	9.68920900	-4.16019900
C	-8.10629000	11.11307400	-5.44977400
O	-8.05059100	12.32189900	-5.63146900
C	-7.81025200	10.16373700	-6.57166000
C	-7.89124800	8.76784600	-6.44175100
C	-7.43391100	10.71170600	-7.81018800
C	-7.60063800	7.93945900	-7.52599800
H	-8.18254400	8.31667600	-5.49866900
C	-7.14476800	9.88522600	-8.89102400
H	-7.37619900	11.79173500	-7.89607800
C	-7.22761100	8.49592200	-8.75082200
H	-7.66732100	6.86087600	-7.41452300
H	-6.85472600	10.31979600	-9.84363800
H	-7.00239600	7.84936700	-9.59471500
O	-10.49087300	12.01280500	-0.63033900
C	-9.17574300	11.61922800	-0.50666000
O	-8.66395900	11.63393200	0.58668800
C	-8.53527200	11.19264100	-1.76285900
C	-7.45962300	10.40316500	-1.86382900
C	-7.14191800	10.09176400	-3.30403600
H	-6.91148000	9.03351100	-3.47176200
H	-6.25816000	10.65806600	-3.63513900
H	-6.88026300	10.03582500	-1.02283800

6-ts

C	-9.48859800	15.22649200	-4.83964400
C	-9.54577000	15.54056400	-3.48686000
C	-9.85745200	14.57236800	-2.51156200
C	-10.11188100	13.25950300	-2.96640300
C	-10.06205300	12.93887900	-4.32179500
C	-9.75165100	13.91982000	-5.26138700
H	-9.25255200	15.99928400	-5.56541500
H	-9.36645400	16.56387700	-3.17183300
H	-10.27850000	11.91891800	-4.62195500
H	-9.71921700	13.66596400	-6.31718600
C	-9.94622400	14.89198000	-1.08910100
H	-10.41026400	14.14992500	-0.44616100
C	-9.51256400	16.01553700	-0.48040400
H	-9.03369900	16.80947700	-1.04254900

C	-9.67917400	16.18199600	0.98100200
O	-10.22800800	15.31730800	1.66693900
C	-9.17759300	17.43846800	1.63546400
C	-8.26536200	18.31899300	1.03267200
C	-9.63855500	17.72377400	2.93174300
C	-7.82752300	19.45772700	1.71001800
H	-7.86872400	18.11148500	0.04426300
C	-9.21173400	18.86592500	3.60236600
H	-10.33587700	17.03031100	3.39007300
C	-8.30344900	19.73693300	2.99225800
H	-7.11324800	20.12554600	1.23611100
H	-9.58402900	19.07936900	4.60076800
H	-7.96671600	20.62804900	3.51544300
O	-10.50980700	12.25253600	-2.10048200
C	-9.61313700	11.76683400	-1.14682200
O	-8.46172600	12.17928300	-1.08627500
C	-10.22927900	10.73007200	-0.36737800
H	-11.15383300	10.30799600	-0.75220200
C	-9.68241300	10.16624300	0.74318000
C	-9.94179700	9.61805500	1.92277300
H	-10.81482700	9.95498700	2.47921800
H	-9.30609100	8.87799800	2.39655300
P	-7.18603300	10.15131500	0.71356400
C	-5.86855200	10.33489200	-0.61741200
H	-4.88757400	10.42718800	-0.14282300
H	-6.11222700	11.30084900	-1.07151200
C	-5.77854600	9.23462000	-1.67574900
H	-5.53557500	8.28359700	-1.19886600
C	-4.63558200	9.48678900	-2.70362700
H	-4.66779800	8.63486900	-3.39285900
C	-4.78402900	10.77793000	-3.50735500
H	-4.67416000	11.65622200	-2.86351200
H	-5.75998400	10.82542500	-4.00255900
H	-4.00930800	10.82964900	-4.27888300
C	-6.69863500	8.67858800	1.71478900
C	-6.44669000	8.75146000	3.09628500
C	-6.70406900	7.40810600	1.10478500
C	-6.17369600	7.59782000	3.83488900
H	-6.45589400	9.71233000	3.59883800
C	-6.41238300	6.26134700	1.84192700
H	-6.94104100	7.29827600	0.05098000
C	-6.14524300	6.35143800	3.20984700
H	-5.97925200	7.67918900	4.90125800
H	-6.39504200	5.29728100	1.34225000
H	-5.92353900	5.45578900	3.78395500
C	-6.75340900	11.59849200	1.77537100
C	-7.71645200	12.57453900	2.06958600

C	-5.44817400	11.76823000	2.27295300
C	-7.38844300	13.68782500	2.84712000
H	-8.72508600	12.48062800	1.68698200
C	-5.12119700	12.87916900	3.05022600
H	-4.68292200	11.02494200	2.06635300
C	-6.09248500	13.84101300	3.33963800
H	-8.15372600	14.42974300	3.05359600
H	-4.10838200	12.99170200	3.42857900
H	-5.83749000	14.70742100	3.94455500
O	-3.39341100	9.52463100	-2.00873100
Si	-2.07317300	8.49479000	-1.89300000
C	-2.57941200	6.82537800	-1.15368500
C	-3.64952500	6.08759300	-1.70111000
C	-1.91646200	6.26946000	-0.04210300
C	-4.02915200	4.85175200	-1.17400000
H	-4.23152900	6.47860300	-2.52930800
C	-2.28451700	5.02850000	0.48198100
H	-1.10865400	6.81564700	0.43599000
C	-3.33997500	4.31307000	-0.08618600
H	-4.86801000	4.32016600	-1.61516600
H	-1.75101700	4.62530400	1.33938500
H	-3.62784400	3.34699900	0.32161500
C	-0.93803300	9.44830900	-0.71636200
C	-1.31873300	10.72573700	-0.26247900
C	0.30479000	8.95200200	-0.27412500
C	-0.50354200	11.46926900	0.59346500
H	-2.26593300	11.14181900	-0.59138200
C	1.12415400	9.69033500	0.58265400
H	0.64707600	7.97150000	-0.59496600
C	0.72102800	10.95310700	1.02000700
H	-0.82499400	12.45359900	0.92549800
H	2.07669700	9.27876900	0.90713200
H	1.35735500	11.53004600	1.68636300
C	-1.22673100	8.26180300	-3.61190600
C	-2.19338000	7.63169600	-4.63890600
H	-1.67408400	7.47988200	-5.59663300
H	-3.05871400	8.27263300	-4.84256400
H	-2.56414200	6.65383800	-4.31145800
C	-0.00117200	7.33023300	-3.47381600
H	0.45654100	7.16699700	-4.46054100
H	-0.27267900	6.34708400	-3.07133000
H	0.77229100	7.76200300	-2.82905100
C	-0.75371400	9.63277900	-4.14251800
H	-0.03544000	10.10578300	-3.46337300
H	-1.58969600	10.32882200	-4.27780600
H	-0.26075700	9.51292900	-5.11841500
N	-7.05417800	9.04878700	-2.36883000

H	-7.65923000	9.85165100	-2.46951200
C	-7.42685800	7.83709600	-2.87643500
O	-6.71651100	6.83848000	-2.73823700
C	-8.78507500	7.72825200	-3.60804700
C	-9.43831300	9.08280900	-3.93004700
H	-8.78097900	9.71863000	-4.53602700
H	-10.35618100	8.91816400	-4.50642400
H	-9.72610300	9.63121200	-3.02649500
C	-8.53456700	6.95404000	-4.91914500
H	-8.03084200	6.00683000	-4.71360000
H	-9.48639700	6.75006900	-5.42343700
H	-7.90513500	7.53183900	-5.60709900
C	-9.71812000	6.90491200	-2.69084300
H	-10.67639600	6.72318700	-3.19187900
H	-9.26238700	5.94005700	-2.44980700
H	-9.92061900	7.43566400	-1.75320300

7-int

C	-8.89286800	15.93053500	-4.67182200
C	-9.11746500	16.08783600	-3.31007200
C	-9.44737600	14.99621700	-2.48098000
C	-9.54140700	13.72164500	-3.08753800
C	-9.31870300	13.56272300	-4.45666200
C	-8.99747500	14.66069200	-5.25020900
H	-8.64608000	16.79305900	-5.28437900
H	-9.05061100	17.08017800	-2.87439200
H	-9.40896100	12.56693000	-4.87916300
H	-8.82924900	14.52789200	-6.31556200
C	-9.71828400	15.14423900	-1.05568800
H	-10.10242800	14.26854900	-0.54226000
C	-9.55414200	16.25588400	-0.30384600
H	-9.18073000	17.17650300	-0.73817500
C	-9.88371500	16.24515600	1.13339400
O	-10.36690200	15.25225100	1.68528000
C	-9.62905300	17.48536800	1.94775600
C	-8.78024500	18.52480400	1.53573800
C	-10.25991300	17.58389000	3.19907800
C	-8.57117200	19.63570300	2.35423300
H	-8.25608800	18.46431000	0.58752300
C	-10.06163900	18.69739700	4.01028300
H	-10.90589700	16.76947900	3.51027200
C	-9.21508000	19.72780800	3.58938900
H	-7.90387600	20.42850800	2.02683300
H	-10.56503300	18.76471400	4.97115900
H	-9.05686700	20.59709800	4.22233900
O	-9.93877800	12.61074200	-2.38282900
C	-9.04239100	12.03086400	-1.44319000

O	-7.94193800	12.56266700	-1.24388700
C	-9.60605600	10.87431300	-0.88310600
H	-10.63968300	10.66086600	-1.13744800
C	-9.05997900	10.10317000	0.20084900
C	-9.77739600	9.23061900	0.96634200
H	-10.84983700	9.14321100	0.81134300
H	-9.34224300	8.60238200	1.73084400
P	-7.28279500	10.21488400	0.62301900
C	-6.16744500	10.23574400	-0.84440100
H	-5.15656800	10.38486000	-0.45337200
H	-6.46310900	11.14897200	-1.37024700
C	-6.12863100	9.01491900	-1.77198200
H	-5.81927500	8.12775300	-1.21258100
C	-5.04402600	9.20286200	-2.87621700
H	-5.06661200	8.28168700	-3.46670200
C	-5.28539600	10.39366700	-3.80089400
H	-5.18719000	11.34257800	-3.26440700
H	-6.28562500	10.34480500	-4.24327600
H	-4.55109300	10.38967800	-4.61275400
C	-6.82239000	8.78443700	1.66719600
C	-6.35221000	8.96470400	2.97684700
C	-6.92184700	7.48033400	1.14731400
C	-5.97570400	7.86290800	3.74717000
H	-6.28141500	9.96031900	3.39950900
C	-6.53564600	6.38618000	1.91894300
H	-7.31836000	7.31075400	0.15246100
C	-6.05986900	6.57523800	3.21843600
H	-5.61663900	8.01652400	4.76084500
H	-6.60080600	5.38767500	1.49849200
H	-5.75894200	5.71973500	3.81673600
C	-6.92994100	11.72026300	1.57777000
C	-7.98316100	12.53090600	2.02083800
C	-5.60362600	12.06582600	1.88811700
C	-7.71366500	13.67201100	2.77729200
H	-9.00817200	12.29122400	1.75996200
C	-5.34182800	13.20610200	2.64619100
H	-4.77341600	11.45142300	1.55049300
C	-6.39606100	14.00771800	3.09164500
H	-8.53966500	14.30209800	3.08965800
H	-4.31466800	13.46892300	2.88332400
H	-6.18855300	14.89935900	3.67713000
O	-3.77828200	9.36205400	-2.23825700
Si	-2.43092000	8.37866600	-2.05291700
C	-2.87719100	6.78004200	-1.13259500
C	-3.91499500	5.94182300	-1.59202800
C	-2.19133100	6.37728600	0.02994900
C	-4.23655200	4.75317300	-0.93299400

H	-4.51563600	6.21627000	-2.45346400
C	-2.50379600	5.18557000	0.68773600
H	-1.40713300	7.00625700	0.44021500
C	-3.52427100	4.36563300	0.20361000
H	-5.04639700	4.13599400	-1.31331600
H	-1.95162400	4.90083500	1.58020500
H	-3.76599900	3.43487600	0.71143000
C	-1.29687000	9.47321600	-1.00260200
C	-1.68441400	10.79224500	-0.69637800
C	-0.04429800	9.04311900	-0.52028900
C	-0.86852500	11.63648400	0.06004600
H	-2.63552600	11.16273200	-1.06656900
C	0.77571100	9.88203300	0.23773000
H	0.30602800	8.03605600	-0.73128300
C	0.36438800	11.18293600	0.53175900
H	-1.19438000	12.65122500	0.27608400
H	1.73563100	9.51897300	0.59644600
H	1.00156100	11.83845500	1.11999100
C	-1.61384100	7.99426500	-3.75654100
C	-2.57768500	7.22758100	-4.68862900
H	-2.07339900	6.99818400	-5.63877700
H	-3.47161700	7.81201200	-4.93405300
H	-2.90228400	6.27524800	-4.25502200
C	-0.35205700	7.12617200	-3.55013800
H	0.09548200	6.88045400	-4.52420700
H	-0.58005200	6.17941000	-3.04602700
H	0.41474300	7.64825300	-2.96711100
C	-1.20458600	9.31783000	-4.43960100
H	-0.49023500	9.88634100	-3.83348400
H	-2.06944700	9.96471100	-4.62702300
H	-0.72856500	9.11188600	-5.40940300
N	-7.43843900	8.74419500	-2.36243300
H	-8.16143600	9.45549200	-2.28138200
C	-7.73917500	7.52232700	-2.89709000
O	-6.91249300	6.60713100	-2.90456700
C	-9.17085100	7.32015300	-3.44887500
C	-9.76752100	8.60687000	-4.05469400
H	-9.12626000	9.00767000	-4.84893000
H	-10.74356700	8.38083400	-4.49982600
H	-9.92976700	9.39608100	-3.31219800
C	-9.11263000	6.22739600	-4.53236100
H	-8.64851100	5.31795900	-4.14471700
H	-10.12543800	5.99172100	-4.87915700
H	-8.52614100	6.55847800	-5.39721100
C	-10.04904700	6.83211800	-2.27177900
H	-11.07209600	6.64574900	-2.61992500
H	-9.65546500	5.89638800	-1.85877100

H	-10.09302200	7.57156400	-1.46444100

8-ts-*re*

C	-12.01216400	15.39752000	-1.38493700
C	-10.69841200	15.01940400	-1.09788800
C	-10.32795700	13.67265600	-0.99221600
C	-11.33692600	12.71332700	-1.19634800
C	-12.65071800	13.07155300	-1.48321700
C	-12.99009100	14.42216200	-1.57845000
H	-12.26548300	16.45107800	-1.46171100
H	-9.92966500	15.77461200	-0.96861600
H	-13.38542100	12.28702400	-1.63415800
H	-14.01433400	14.70499700	-1.80458200
C	-8.92960900	13.24565200	-0.68056100
H	-8.44809100	12.65607100	-1.46163700
C	-8.03265100	14.10034100	-0.00809100
H	-8.41280900	14.91924900	0.59249500
C	-6.63395500	13.85785900	-0.09689400
O	-6.15329700	12.89254300	-0.75388300
C	-5.67204000	14.80483500	0.57944800
C	-6.02937800	15.65333100	1.63924300
C	-4.34311900	14.82658400	0.12854000
C	-5.08841600	16.50105300	2.22437700
H	-7.04229000	15.63671300	2.02969100
C	-3.40250000	15.67783000	0.70596200
H	-4.07304500	14.16127400	-0.68500500
C	-3.77196300	16.52037400	1.75758000
H	-5.38249300	17.14467800	3.04986300
H	-2.37977700	15.68545600	0.33636300
H	-3.04035200	17.18341000	2.21238500
O	-11.06785000	11.34674300	-1.19431300
C	-10.02758900	10.82004100	-0.47008100
O	-9.65994900	9.68773400	-0.77896900
C	-9.41449500	11.68026300	0.51397900
H	-10.11708700	12.28069900	1.08600200
C	-8.25172400	11.26987900	1.29496500
C	-7.85329700	12.02567700	2.34939000
P	-7.16814200	9.86878900	0.83496900
C	-6.73017500	9.88663100	-0.96314000
H	-5.82352700	10.48876500	-1.02371000
H	-7.51058900	10.45251900	-1.46791500
C	-6.48122200	8.54979400	-1.67435900
H	-5.87170400	7.88647000	-1.05497200
C	-5.64282800	8.78341600	-2.96892200
H	-5.55626600	7.80161300	-3.44276000
C	-6.27601900	9.76800100	-3.94931800
H	-6.29411300	10.78075800	-3.53319500

H	-7.29950900	9.46579000	-4.19606800
H	-5.69487100	9.79772000	-4.87640000
C	-7.91440400	8.30247700	1.40834400
C	-7.19120500	7.09997000	1.33142000
C	-9.19673700	8.29976300	1.97550700
C	-7.75346900	5.91448400	1.80494000
H	-6.18728000	7.07457600	0.92045900
C	-9.75103700	7.11173400	2.45161700
H	-9.76013900	9.22320900	2.04290700
C	-9.03293500	5.91755100	2.36475500
H	-7.18596300	4.99132000	1.73503500
H	-10.74527200	7.12163800	2.88904000
H	-9.46659000	4.99303200	2.73608500
C	-5.59318700	10.04278100	1.74969500
C	-5.31857900	9.27367700	2.89214200
C	-4.66196200	11.00716300	1.32222000
C	-4.11825500	9.44826100	3.58171000
H	-6.03206100	8.53778200	3.24500300
C	-3.46454400	11.17154900	2.01803600
H	-4.88551800	11.65760900	0.47919500
C	-3.18786300	10.39122300	3.14241800
H	-3.91379700	8.84472700	4.46157300
H	-2.75394400	11.91827200	1.67690100
H	-2.25293300	10.52377300	3.68040900
O	-4.35891400	9.27662700	-2.59949100
Si	-2.85012200	8.57268800	-2.39163800
C	-2.86995000	7.34899500	-0.93894200
C	-3.69171700	6.20117600	-0.95883600
C	-2.09162500	7.56369500	0.21493900
C	-3.71564900	5.30569200	0.11361400
H	-4.35585300	6.00576100	-1.79511000
C	-2.10908400	6.66822100	1.28704400
H	-1.46939300	8.45032000	0.28800300
C	-2.91702700	5.53166500	1.23719900
H	-4.35925200	4.43073200	0.06344100
H	-1.49416600	6.86266000	2.16241300
H	-2.92907000	4.83098900	2.06869600
C	-1.77553600	10.07584600	-1.98125800
C	-2.31115100	11.37409600	-2.08732400
C	-0.42244300	9.96449100	-1.60172100
C	-1.53193100	12.50518500	-1.83101400
H	-3.35129400	11.50011500	-2.37148200
C	0.35902800	11.09158400	-1.33850100
H	0.03708900	8.98428100	-1.50236300
C	-0.19458700	12.36778100	-1.45481300
H	-1.97088100	13.49476100	-1.93209800
H	1.39934600	10.97211500	-1.04585100

H	0.41262400	13.24752200	-1.25643200
C	-2.23168900	7.74447200	-4.02192400
C	-3.05689400	6.49938300	-4.41633100
H	-2.66946300	6.08301600	-5.35793400
H	-4.11669900	6.72503100	-4.57843800
H	-2.99427700	5.70791800	-3.66215000
C	-0.76015800	7.30149300	-3.85282500
H	-0.41666800	6.79507500	-4.76655900
H	-0.63511400	6.59537000	-3.02229200
H	-0.09142600	8.15182800	-3.68285100
C	-2.31179900	8.78006900	-5.16590100
H	-1.73053500	9.68204800	-4.94286300
H	-3.34431500	9.09051600	-5.36308500
H	-1.91317400	8.34847200	-6.09560900
N	-7.73377700	7.86514600	-1.98404900
H	-8.59823100	8.37616100	-1.84094700
C	-7.75389900	6.53809500	-2.29228800
O	-6.71681500	5.86921500	-2.34168700
C	-9.11410800	5.88378800	-2.63197200
C	-10.33063500	6.80184900	-2.42667700
H	-10.29559900	7.68831200	-3.07028900
H	-11.24617600	6.25460000	-2.67985700
H	-10.42421000	7.14306200	-1.39058000
C	-9.03094800	5.45478400	-4.11496300
H	-8.16676500	4.80477100	-4.27852600
H	-9.93953100	4.91251700	-4.40271400
H	-8.93540700	6.32550100	-4.77520400
C	-9.25442000	4.62835300	-1.74550800
H	-10.14986000	4.06321200	-2.03066400
H	-8.37949100	3.98251700	-1.85715200
H	-9.34786100	4.90073500	-0.68780300
H	-8.47093700	12.85425800	2.68204400
H	-6.94813800	11.83768700	2.91217700

8-ts-*si*

C	-8.16788500	16.96330700	-0.84339300
C	-8.52711300	15.65286900	-1.16413400
C	-7.75329300	14.55534100	-0.75979400
C	-6.58726500	14.82842800	-0.01576700
C	-6.21949400	16.13119300	0.31287400
C	-7.01025400	17.20282600	-0.10362400
H	-8.78808100	17.79015200	-1.17754200
H	-9.41424000	15.46546400	-1.76084600
H	-5.31244200	16.28352600	0.88908800
H	-6.71873100	18.21789700	0.15137500
C	-8.13862400	13.15688700	-1.09616000
H	-7.39137100	12.58219900	-1.63972400

C	-9.46730300	12.80268700	-1.34915400
H	-10.26455500	13.45940500	-1.01831700
C	-9.81480100	11.60297600	-2.03078300
O	-8.96836400	10.75348900	-2.43688900
C	-11.27047200	11.36518400	-2.33810500
C	-12.30120200	11.75755300	-1.46899600
C	-11.61399800	10.71834200	-3.53529400
C	-13.63635400	11.51068100	-1.78929100
H	-12.05453500	12.23269800	-0.52412500
C	-12.94860600	10.48433000	-3.86390500
H	-10.81926300	10.41670400	-4.20941700
C	-13.96549000	10.87769300	-2.99025200
H	-14.42064400	11.80966100	-1.09844100
H	-13.19642600	9.99558400	-4.80278900
H	-15.00612800	10.69045000	-3.24196700
O	-5.70638400	13.83533000	0.37959500
C	-6.12527800	12.54306300	0.65945800
O	-5.25075700	11.68473600	0.66750700
C	-7.54002100	12.35093600	0.83476200
H	-8.07112300	13.16596500	1.31518900
C	-8.16183400	11.05836800	1.03366300
C	-9.45971400	10.96283700	1.42690000
H	-10.00856900	11.86379200	1.68662200
H	-9.98847700	10.02481800	1.52771600
P	-7.28064600	9.49190300	0.66444200
C	-6.41717400	9.59325500	-0.96031500
H	-5.43668900	9.99079800	-0.70604900
H	-6.96070000	10.37077600	-1.50637600
C	-6.26622400	8.34888600	-1.85560900
H	-6.20958500	7.42214800	-1.27496700
C	-4.92319400	8.43827700	-2.64203100
H	-4.94313300	7.60966800	-3.35555000
C	-4.75090900	9.75708400	-3.39904100
H	-4.64965200	10.61041700	-2.72295200
H	-5.61475800	9.92606100	-4.05127700
H	-3.85736900	9.73380400	-4.02874100
C	-8.49436700	8.12846200	0.66132900
C	-8.36168100	7.05451100	1.55547000
C	-9.57517000	8.15373700	-0.23815200
C	-9.30177500	6.02309500	1.55592800
H	-7.53169400	7.02097500	2.25226500
C	-10.51655200	7.12529700	-0.21992500
H	-9.68139300	8.97237800	-0.94258600
C	-10.38194900	6.06024600	0.67413200
H	-9.18873800	5.19482400	2.24952300
H	-11.35480100	7.15877800	-0.90907900
H	-11.11765700	5.26061700	0.68104300

C	-6.11490300	9.14561000	2.01488600
C	-6.45390300	9.53814600	3.31868800
C	-4.91255300	8.46635900	1.77610000
C	-5.59169300	9.25166700	4.37611100
H	-7.37929600	10.07564100	3.50270200
C	-4.05345900	8.18708500	2.84048100
H	-4.62913100	8.16897900	0.77126700
C	-4.39184500	8.57801500	4.13730600
H	-5.85400000	9.56358700	5.38323600
H	-3.11968000	7.66928400	2.64786500
H	-3.71743900	8.36256300	4.96169900
O	-3.88850800	8.19583100	-1.68469000
Si	-2.21665800	8.09981800	-1.84385100
C	-1.70042000	7.19995800	-0.26161200
C	-2.42636200	6.08590800	0.20434800
C	-0.54721700	7.57045800	0.45395500
C	-2.01209300	5.36540100	1.32680900
H	-3.33359800	5.78279800	-0.31214100
C	-0.12819100	6.85561500	1.57925200
H	0.02974700	8.43467400	0.13327500
C	-0.85806800	5.74788400	2.01585900
H	-2.58932800	4.50793100	1.66405300
H	0.76635100	7.16420800	2.11464500
H	-0.53125100	5.18741600	2.88827200
C	-1.53531300	9.86565200	-1.81402700
C	-2.20816900	10.80512000	-1.00455200
C	-0.40873400	10.31831500	-2.52565800
C	-1.78866200	12.13278700	-0.91968100
H	-3.08125300	10.50997900	-0.43051200
C	0.02265400	11.64476700	-2.43908200
H	0.14488500	9.63667900	-3.16508900
C	-0.66896200	12.55609700	-1.63916400
H	-2.35034700	12.82667600	-0.30007700
H	0.89525000	11.96626700	-3.00257000
H	-0.33818000	13.59002600	-1.57923800
C	-1.67425000	7.03050100	-3.36013900
C	-2.47226800	5.70440900	-3.35024200
H	-2.16978400	5.08385200	-4.20630200
H	-3.55470000	5.85727300	-3.42345500
H	-2.27753200	5.12192000	-2.44296700
C	-0.17217200	6.67616500	-3.23711600
H	0.11911000	6.01233100	-4.06395800
H	0.05008500	6.15347600	-2.30057700
H	0.47745000	7.55715100	-3.28798800
C	-1.90764400	7.73775600	-4.71332600
H	-1.40997400	8.71246100	-4.76952700
H	-2.97260400	7.89201300	-4.91748200

H	-1.51204000	7.11804400	-5.53122200
N	-7.38890400	8.23635400	-2.78298500
H	-8.00500000	9.03840300	-2.89403800
C	-7.56976800	7.09387300	-3.51468300
O	-6.85156100	6.10810700	-3.34557300
C	-8.69753900	7.11431600	-4.57670000
C	-8.51606600	8.32442000	-5.52064700
H	-7.53470400	8.30191800	-6.00922700
H	-9.28013500	8.29841900	-6.30715100
H	-8.61245100	9.28077600	-4.99572500
C	-8.61638700	5.81184900	-5.38984900
H	-8.72264800	4.93645400	-4.74348400
H	-9.41438100	5.79381300	-6.14174100
H	-7.65363000	5.72376900	-5.90249700
C	-10.07217100	7.19952500	-3.87972100
H	-10.87252000	7.15599600	-4.62878000
H	-10.21224900	6.36217300	-3.18704800
H	-10.19071900	8.13342600	-3.32223200

9-int-*re*

C	-11.74003200	15.48869600	-1.70184100
C	-10.49860600	15.06063700	-1.22514300
C	-10.22287300	13.70201200	-1.03866800
C	-11.23972800	12.79702700	-1.36722000
C	-12.48434800	13.19533500	-1.84255000
C	-12.73510600	14.55811700	-2.00686500
H	-11.92771500	16.55033100	-1.83553100
H	-9.71771400	15.77942200	-0.99935800
H	-13.22709300	12.44199200	-2.08503700
H	-13.70217600	14.88673200	-2.37663200
C	-8.90497100	13.16042600	-0.52238100
H	-8.32250500	12.74566200	-1.35851400
C	-8.01640400	14.11126100	0.17760800
H	-8.43400400	14.95672500	0.71188300
C	-6.64745600	13.88585600	0.08450200
O	-6.16338000	12.88029100	-0.55263600
C	-5.67474300	14.84431300	0.72499100
C	-6.01611400	15.71325500	1.77409300
C	-4.35175500	14.86177100	0.25715400
C	-5.07044200	16.57672100	2.32746100
H	-7.02432900	15.69919900	2.17814600
C	-3.40452200	15.72665700	0.80465500
H	-4.09181300	14.18008600	-0.54614000
C	-3.75961800	16.59050600	1.84346800
H	-5.35532000	17.23600800	3.14427500
H	-2.38651800	15.72717000	0.42107100
H	-3.02296900	17.26319700	2.27577400

O	-11.01816900	11.41391200	-1.30562400
C	-10.01630800	10.90356000	-0.55279600
O	-9.72099700	9.73055600	-0.70891600
C	-9.33165800	11.86328400	0.37475400
H	-10.09973000	12.26097900	1.05088100
C	-8.22128500	11.31296800	1.23278300
C	-7.92998900	11.98163200	2.36721300
P	-7.11918700	9.93602100	0.77920200
C	-6.70775700	9.97386700	-1.02290800
H	-5.81918200	10.60332400	-1.08518000
H	-7.50241100	10.53952500	-1.50754000
C	-6.45247800	8.65584900	-1.76359300
H	-5.81463900	7.99277600	-1.17339100
C	-5.65459200	8.92622300	-3.07703900
H	-5.55422000	7.95182000	-3.56371200
C	-6.34055600	9.90170700	-4.03087900
H	-6.37578500	10.90947900	-3.60391600
H	-7.36078900	9.57290900	-4.25662900
H	-5.78333800	9.95705600	-4.97125500
C	-7.85714800	8.35401000	1.33677700
C	-7.14823100	7.14894600	1.19498000
C	-9.10593000	8.34180500	1.97363900
C	-7.69278500	5.95470200	1.66732100
H	-6.16493400	7.12738500	0.73730200
C	-9.64440300	7.14557900	2.44797100
H	-9.65667300	9.26655000	2.10096700
C	-8.94164500	5.94957500	2.29266400
H	-7.13461600	5.03113900	1.54602700
H	-10.61339900	7.15106600	2.93894000
H	-9.36160500	5.01834200	2.66286100
C	-5.55699200	10.08866100	1.72009600
C	-5.28945900	9.26887500	2.83002600
C	-4.62653100	11.07330000	1.34083000
C	-4.09532500	9.41749900	3.53587000
H	-6.00103700	8.51474400	3.14572700
C	-3.43490600	11.20846400	2.05300000
H	-4.86378600	11.76299800	0.53067600
C	-3.16441500	10.38124500	3.14468700
H	-3.89621500	8.77753400	4.39091900
H	-2.72543700	11.97339000	1.75188200
H	-2.23461500	10.49334600	3.69627400
O	-4.37717800	9.45259000	-2.73678400
Si	-2.84438000	8.78789200	-2.57184600
C	-2.81020500	7.52494400	-1.15401900
C	-3.59804200	6.35422600	-1.19408200
C	-2.02490800	7.73352300	-0.00371500
C	-3.58280800	5.43124100	-0.14521300

H	-4.26594100	6.16074700	-2.02767500
C	-2.00263800	6.81054800	1.04469500
H	-1.42883200	8.63654300	0.08495400
C	-2.77726000	5.65207500	0.97437300
H	-4.20127800	4.53934300	-0.21029300
H	-1.38349600	7.00091600	1.91791400
H	-2.75858500	4.93028200	1.78748000
C	-1.80303800	10.30620100	-2.13854100
C	-2.37277200	11.59282700	-2.19759900
C	-0.44040300	10.21784100	-1.78791900
C	-1.61645000	12.73556900	-1.92508800
H	-3.42159000	11.70121900	-2.45571900
C	0.31818600	11.35653000	-1.50852600
H	0.04447500	9.24685700	-1.72385600
C	-0.26890200	12.62108400	-1.57907600
H	-2.08216500	13.71582400	-1.98780100
H	1.36657300	11.25524900	-1.23864600
H	0.32008600	13.51003900	-1.36738900
C	-2.23499000	8.02386600	-4.23688000
C	-3.05386000	6.78650800	-4.66640100
H	-2.66970200	6.40270100	-5.62315100
H	-4.11563100	7.01181500	-4.81614400
H	-2.98255200	5.97113900	-3.93884300
C	-0.75745000	7.59017400	-4.10020200
H	-0.41735400	7.12565600	-5.03719100
H	-0.61655200	6.85223900	-3.30055300
H	-0.09640100	8.44034700	-3.90163000
C	-2.33845300	9.09909400	-5.34195000
H	-1.75512600	9.99403900	-5.09739900
H	-3.37502700	9.41415700	-5.50907800
H	-1.95606200	8.70187600	-6.29348300
N	-7.70143900	7.95181000	-2.05173200
H	-8.57336300	8.43991500	-1.88584500
C	-7.70884200	6.62965000	-2.38639700
O	-6.66345200	5.97875000	-2.46757800
C	-9.06539300	5.96215300	-2.71524900
C	-10.29150000	6.86022000	-2.47965100
H	-10.27713800	7.75670400	-3.11061600
H	-11.20274500	6.30501400	-2.73066800
H	-10.37812900	7.18218700	-1.43666700
C	-8.99811000	5.55673600	-4.20578900
H	-8.12852400	4.92007400	-4.39118400
H	-9.90398300	5.00755400	-4.48872100
H	-8.92245000	6.43819300	-4.85424800
C	-9.17607800	4.69203500	-1.84592900
H	-10.06732200	4.11853900	-2.12724800
H	-8.29380300	4.06069600	-1.98003400

H	-9.25824800	4.94746500	-0.78314400
H	-8.56224900	12.79879000	2.69877500
H	-7.07462300	11.74780500	2.99097500

9-int-*si*

C	-8.27707500	16.92795500	-1.64833100
C	-8.68302200	15.59243800	-1.69202700
C	-7.94251000	14.58780500	-1.05962400
C	-6.77207200	14.97914000	-0.39888100
C	-6.34775700	16.30297100	-0.33728500
C	-7.11209000	17.28533000	-0.96660600
H	-8.87165700	17.68765600	-2.14775600
H	-9.58167400	15.30804100	-2.22947700
H	-5.42825700	16.54045900	0.18812300
H	-6.79329500	18.32304200	-0.92698700
C	-8.32807500	13.11756600	-1.06502400
H	-7.71722500	12.59886000	-1.81825300
C	-9.75020000	12.83063200	-1.38434700
H	-10.51192900	13.55336300	-1.11398200
C	-10.07838200	11.67521400	-2.06345900
O	-9.20884200	10.77744700	-2.41416500
C	-11.52113300	11.44162300	-2.43078100
C	-12.58090300	11.82491300	-1.59232000
C	-11.83530200	10.81106300	-3.64460700
C	-13.90772200	11.59671800	-1.95917400
H	-12.35890200	12.28795900	-0.63484800
C	-13.16059400	10.58883200	-4.01857200
H	-11.02369000	10.51056800	-4.29828900
C	-14.20469700	10.97929400	-3.17638600
H	-14.71125100	11.89449600	-1.28942900
H	-13.37985600	10.11079100	-4.97054300
H	-15.23792600	10.80056800	-3.46322100
O	-5.91782300	14.03508700	0.17776400
C	-6.35077400	12.78002600	0.46443200
O	-5.52600700	11.93903100	0.75475400
C	-7.84197400	12.56154800	0.36622100
H	-8.30540900	13.24491100	1.09038100
C	-8.32790600	11.17048600	0.71637300
C	-9.55080500	11.04176100	1.26038800
H	-10.13155200	11.92320400	1.51583100
H	-10.01803300	10.08288400	1.45394600
P	-7.40177000	9.63995500	0.34538500
C	-6.44451400	9.84843900	-1.21285400
H	-5.46224400	10.18938000	-0.88375800
H	-6.94547700	10.66856600	-1.73388900
C	-6.28919700	8.66412500	-2.18490400
H	-6.24850700	7.70309000	-1.65930900

C	-4.92835700	8.78660300	-2.93266200
H	-4.94582300	8.01262100	-3.70278300
C	-4.70488400	10.14950600	-3.58926500
H	-4.58472500	10.94837300	-2.85178300
H	-5.55403000	10.39266900	-4.23732800
H	-3.80291300	10.14085400	-4.20801400
C	-8.57234800	8.23956600	0.28546900
C	-8.37757000	7.12353400	1.11721600
C	-9.67633000	8.27929300	-0.58470500
C	-9.28193500	6.06183600	1.08282200
H	-7.52964600	7.07967300	1.79093800
C	-10.57869400	7.21604000	-0.60010800
H	-9.80940800	9.13226200	-1.24783000
C	-10.38531300	6.10939000	0.22992100
H	-9.12318000	5.20167000	1.72696100
H	-11.43342700	7.25639100	-1.26858900
H	-11.09265600	5.28459600	0.21046700
C	-6.26337400	9.26637000	1.71992300
C	-6.62462800	9.61140500	3.03034600
C	-5.05498300	8.59694600	1.48103000
C	-5.78181600	9.28934300	4.09290500
H	-7.55249700	10.14313800	3.21748900
C	-4.21484300	8.27996500	2.55068500
H	-4.75214600	8.32931300	0.47331300
C	-4.57663900	8.62499100	3.85382600
H	-6.06351400	9.56540300	5.10525000
H	-3.27885400	7.76630900	2.35631800
H	-3.91800000	8.38040400	4.68273000
O	-3.92151700	8.45992500	-1.96790100
Si	-2.27153000	8.19463400	-2.15091500
C	-1.82627000	7.21870300	-0.59221900
C	-2.64320500	6.16203500	-0.14392800
C	-0.64466600	7.47763900	0.12585400
C	-2.29074400	5.39118900	0.96619800
H	-3.57208700	5.94413000	-0.66500900
C	-0.28686900	6.71202400	1.23865000
H	0.00409600	8.29364300	-0.18328400
C	-1.10834300	5.66407900	1.65946000
H	-2.93774700	4.57965300	1.29019700
H	0.63159300	6.93396800	1.77641600
H	-0.82997300	5.06392900	2.52221800
C	-1.41205500	9.88210500	-2.08102200
C	-1.94261600	10.84724900	-1.19896900
C	-0.27125700	10.24506500	-2.82020500
C	-1.36767100	12.11145600	-1.06679000
H	-2.82291000	10.61414100	-0.60512600
C	0.31061300	11.50915500	-2.69243100

H	0.17741300	9.53968400	-3.51322300
C	-0.23739300	12.44627800	-1.81598000
H	-1.80696300	12.83280700	-0.38265900
H	1.18993300	11.76070600	-3.28026400
H	0.21232900	13.43107400	-1.71765400
C	-1.85444900	7.12653500	-3.70396800
C	-2.76873300	5.87663900	-3.72543900
H	-2.52459600	5.25883800	-4.60171900
H	-3.83472700	6.12128900	-3.78642800
H	-2.61786400	5.25209900	-2.83788900
C	-0.38962100	6.63256900	-3.61364500
H	-0.16960000	5.97381300	-4.46605300
H	-0.20776000	6.05895700	-2.69829700
H	0.33927400	7.45042200	-3.64254800
C	-2.03917900	7.89785200	-5.03039700
H	-1.44492700	8.81774600	-5.06945400
H	-3.08575500	8.16783200	-5.20737000
H	-1.72501500	7.26703900	-5.87460000
N	-7.39565700	8.63077000	-3.13452100
H	-8.08315600	9.38651700	-3.10800000
C	-7.55634800	7.54889000	-3.95628100
O	-6.80602100	6.57325200	-3.88287600
C	-8.70345500	7.62061900	-4.99348600
C	-8.65947300	8.96348900	-5.75622400
H	-7.69758700	9.09772000	-6.26573900
H	-9.44556200	8.98048900	-6.52108500
H	-8.81878900	9.82194700	-5.09567100
C	-8.53070000	6.45804000	-5.98497400
H	-8.54073900	5.49467500	-5.46855000
H	-9.34492800	6.47409200	-6.71924000
H	-7.57943800	6.53172000	-6.52190200
C	-10.05748000	7.47847400	-4.26468100
H	-10.87410200	7.45934800	-4.99702800
H	-10.09663300	6.54558800	-3.69064700
H	-10.23800700	8.31519300	-3.58454500

10-ts-*re*

C	-11.58092300	15.64005000	-1.43213600
C	-10.38267600	15.12654900	-0.92881000
C	-10.14910100	13.74902300	-0.88157500
C	-11.16044700	12.91018800	-1.37328600
C	-12.35988200	13.39738900	-1.88078100
C	-12.57041100	14.77685300	-1.90560900
H	-11.73790300	16.71471300	-1.45614700
H	-9.60743600	15.79782700	-0.57267500
H	-13.10191200	12.69812700	-2.25266500
H	-13.50310200	15.17164500	-2.29815700

C	-8.89678600	13.08698600	-0.35484700
H	-8.28231500	12.75657200	-1.20483100
C	-7.99124300	13.86812300	0.53649300
H	-8.41048500	14.68229400	1.11813200
C	-6.60180600	13.85116400	0.23399900
O	-6.11254900	13.02882300	-0.59006600
C	-5.66566900	14.80023900	0.94198200
C	-6.01625100	15.53519900	2.08589400
C	-4.36998500	14.95203200	0.42499000
C	-5.10088500	16.39762000	2.68964500
H	-7.00333900	15.42238600	2.52390500
C	-3.45536200	15.81790000	1.02224200
H	-4.10566400	14.37411000	-0.45429800
C	-3.81738800	16.54555200	2.15847900
H	-5.38920200	16.95236300	3.57919200
H	-2.45826100	15.92603600	0.60198000
H	-3.10580600	17.21994200	2.62806200
O	-10.98058100	11.52187600	-1.44812300
C	-10.06581700	10.88350600	-0.67091500
O	-9.84441100	9.70855700	-0.89412200
C	-9.40610600	11.73524100	0.37217800
H	-10.21033200	12.07949600	1.04194700
C	-8.28461000	11.19890900	1.21193200
C	-7.89070500	12.18225300	2.11396100
P	-7.15725000	9.91018500	0.75818800
C	-6.73889200	9.91578300	-1.05105000
H	-5.85945200	10.55661100	-1.13194800
H	-7.54714300	10.45945100	-1.54157900
C	-6.46838800	8.59484800	-1.78278700
H	-5.83041900	7.94160700	-1.18184400
C	-5.66943000	8.85397200	-3.09684400
H	-5.55047900	7.87405300	-3.56685200
C	-6.36292200	9.80222000	-4.07174600
H	-6.41644600	10.81690700	-3.66352400
H	-7.37700000	9.45463200	-4.29605100
H	-5.80119900	9.84930100	-5.01001200
C	-7.80761500	8.27074800	1.27098700
C	-6.99966000	7.12251600	1.21198000
C	-9.10980600	8.16595100	1.77696900
C	-7.49775500	5.89121400	1.63714900
H	-5.97563200	7.17747900	0.85690600
C	-9.60487400	6.93196900	2.20168800
H	-9.72932100	9.05329600	1.84169600
C	-8.80117500	5.79250200	2.13046200
H	-6.86204900	5.01228200	1.58411500
H	-10.61670000	6.86389500	2.59172200
H	-9.18538300	4.83269600	2.46541300

C	-5.59743500	10.10016500	1.69897700
C	-5.39897900	9.39933400	2.90060600
C	-4.60858100	10.99237200	1.25023200
C	-4.22196900	9.57208700	3.62885700
H	-6.15537900	8.71494100	3.26795200
C	-3.43088500	11.15282900	1.98147300
H	-4.77598300	11.60434700	0.36887300
C	-3.23311500	10.44225800	3.16660100
H	-4.08016600	9.02411500	4.55627300
H	-2.67529300	11.84422600	1.62060300
H	-2.31524100	10.57318100	3.73376700
O	-4.39924600	9.40600000	-2.75876000
Si	-2.86687900	8.74915600	-2.56418300
C	-2.85200800	7.46905900	-1.16148600
C	-3.61154200	6.28176000	-1.24177900
C	-2.11418300	7.68240400	0.01943200
C	-3.61288800	5.34741700	-0.20303400
H	-4.24825500	6.08509100	-2.09840400
C	-2.10947400	6.74882600	1.05844800
H	-1.54260300	8.59740800	0.13978800
C	-2.85373900	5.57353500	0.94733700
H	-4.21026500	4.44422500	-0.29927000
H	-1.52828100	6.94395000	1.95633700
H	-2.84843900	4.84362000	1.75332000
C	-1.84642000	10.26935000	-2.08590700
C	-2.42142100	11.55344600	-2.14706800
C	-0.49462100	10.18427000	-1.69525600
C	-1.68137400	12.69683800	-1.83527700
H	-3.46126100	11.65878100	-2.44032900
C	0.24798100	11.32344600	-1.37727300
H	-0.00650100	9.21508600	-1.62862800
C	-0.34463000	12.58545300	-1.44803600
H	-2.15044500	13.67538700	-1.90089500
H	1.28819800	11.22465800	-1.07668600
H	0.23207500	13.47473300	-1.20597800
C	-2.21210100	8.01481500	-4.22533300
C	-3.02616200	6.79085500	-4.69932300
H	-2.62070900	6.42328200	-5.65370000
H	-4.08248800	7.02611400	-4.87047600
H	-2.97710900	5.96122600	-3.98631400
C	-0.74088100	7.57169100	-4.05655400
H	-0.37480000	7.12894100	-4.99433100
H	-0.62669300	6.81382200	-3.27143500
H	-0.08274600	8.41389500	-3.81754100
C	-2.28259800	9.11121500	-5.31181500
H	-1.69878500	9.99710900	-5.03711400
H	-3.31304800	9.43681800	-5.49593500

H	-1.88123800	8.72909000	-6.26171800
N	-7.71400500	7.87945400	-2.06261000
H	-8.58823300	8.35366800	-1.87085100
C	-7.71034600	6.55986000	-2.40590500
O	-6.65706600	5.92850400	-2.53077400
C	-9.06485800	5.87227600	-2.70067900
C	-10.29951600	6.69920200	-2.30451900
H	-10.36487200	7.64558300	-2.85264100
H	-11.20735400	6.12980900	-2.53610800
H	-10.31587500	6.92730600	-1.23354200
C	-9.08861400	5.60003000	-4.22283000
H	-8.21150500	5.01966300	-4.52356900
H	-9.99078400	5.03788500	-4.49205600
H	-9.09331600	6.53710600	-4.79295300
C	-9.07493400	4.52993600	-1.94049300
H	-9.96577200	3.95029800	-2.21053900
H	-8.18442000	3.94551500	-2.18500900
H	-9.09061300	4.69226500	-0.85644600
H	-8.65330200	12.77506100	2.61070200
H	-6.94029500	12.14389300	2.63692300

10-ts-*si*

C	-9.38753800	16.83209200	-1.39455900
C	-9.58794000	15.44957900	-1.37204300
C	-8.60601400	14.58401500	-0.88096700
C	-7.40866700	15.15669400	-0.42412500
C	-7.18846200	16.53077100	-0.43874100
C	-8.18886100	17.37261000	-0.92607100
H	-10.16666200	17.48345200	-1.78020400
H	-10.51553300	15.02744400	-1.74656000
H	-6.24121300	16.91769900	-0.07697400
H	-8.02729400	18.44671200	-0.94095400
C	-8.71928900	13.07782100	-0.80807800
H	-8.15677300	12.64310400	-1.64680500
C	-10.08720000	12.46552100	-0.85003400
H	-10.91292600	13.06251700	-0.47465400
C	-10.36848500	11.48973800	-1.84631500
O	-9.45523200	10.88549300	-2.48325300
C	-11.80303800	11.12286900	-2.12229200
C	-12.82717900	11.27824100	-1.17357400
C	-12.13554200	10.60491400	-3.38360500
C	-14.14212700	10.92604000	-1.47750100
H	-12.59439100	11.65685500	-0.18292800
C	-13.45175900	10.26537300	-3.69387900
H	-11.34761100	10.48642100	-4.11889200
C	-14.46070000	10.42197100	-2.74073600
H	-14.91853200	11.04203200	-0.72549800

H	-13.69103000	9.87794500	-4.68103400
H	-15.48623500	10.15206100	-2.97893900
O	-6.33876600	14.36952400	0.00845100
C	-6.51749300	13.07439300	0.41507000
O	-5.53724000	12.40058200	0.63534800
C	-7.95376200	12.64098400	0.52639100
H	-8.39666100	13.26477900	1.32050100
C	-8.30564600	11.20401500	0.78750100
C	-9.69999100	11.10224300	0.83978700
H	-10.24604000	11.82798700	1.43859500
H	-10.20995000	10.14832800	0.74131500
P	-7.26306400	9.83505000	0.39990700
C	-6.23466600	10.05886200	-1.14021300
H	-5.24101300	10.31028700	-0.76642800
H	-6.62092500	10.95813200	-1.62709100
C	-6.11651200	8.92335500	-2.17350300
H	-6.16325000	7.93915900	-1.69461500
C	-4.72766600	8.97695000	-2.87616100
H	-4.78340200	8.24761600	-3.68645300
C	-4.37013000	10.34498900	-3.45712100
H	-4.22056400	11.09685700	-2.67651500
H	-5.16721600	10.68570100	-4.12746300
H	-3.44379700	10.28718400	-4.03703100
C	-8.25653100	8.29880600	0.31165200
C	-8.04312800	7.26257000	1.23539900
C	-9.25099300	8.15018100	-0.67093300
C	-8.81149000	6.09906000	1.17814900
H	-7.28153700	7.35970300	2.00024800
C	-10.01727500	6.98551100	-0.71705600
H	-9.43054000	8.94441700	-1.38848800
C	-9.79996700	5.95891800	0.20401600
H	-8.63521700	5.30484000	1.89820100
H	-10.78507600	6.88447700	-1.47817200
H	-10.39886700	5.05318500	0.16150100
C	-6.06984800	9.55810500	1.76217700
C	-6.39141900	10.01234500	3.04818200
C	-4.86974800	8.86285000	1.55139800
C	-5.52129800	9.77597400	4.11239300
H	-7.31554500	10.56103100	3.20357300
C	-4.00144100	8.63013600	2.61983400
H	-4.59572500	8.50499500	0.56361500
C	-4.32583200	9.08639600	3.89917000
H	-5.77434700	10.13725500	5.10554200
H	-3.07470000	8.09236500	2.44503800
H	-3.64625400	8.90733200	4.72818000
O	-3.77201500	8.53010900	-1.90772000
Si	-2.20106700	7.96933700	-2.13269800

C	-1.86330300	6.99510500	-0.54708600
C	-2.83224700	6.12528200	-0.00923100
C	-0.62133500	7.07090300	0.10951700
C	-2.57024000	5.35956200	1.12895000
H	-3.80884100	6.05235500	-0.48110700
C	-0.35264500	6.30818300	1.24891900
H	0.14631200	7.74143200	-0.26899500
C	-1.32669900	5.44793600	1.75981700
H	-3.33558900	4.69575400	1.52354500
H	0.61526400	6.38711000	1.73777400
H	-1.11946700	4.85147100	2.64483200
C	-1.04740900	9.47400300	-2.16904900
C	-1.34927700	10.54753800	-1.30525500
C	0.10750600	9.59360700	-2.96373000
C	-0.54149800	11.68319300	-1.24061700
H	-2.23406300	10.49872000	-0.67531900
C	0.92149200	10.72795000	-2.90450100
H	0.38436000	8.79664600	-3.64704400
C	0.59822600	11.77634800	-2.04266400
H	-0.80361100	12.49492300	-0.56685700
H	1.80579300	10.79177100	-3.53377600
H	1.22908000	12.66043200	-1.99619900
C	-2.04448500	6.79567300	-3.65597400
C	-3.16853600	5.73257000	-3.59961000
H	-3.05877800	5.03802200	-4.44537500
H	-4.17554000	6.15831100	-3.65932000
H	-3.10866700	5.13474300	-2.68287600
C	-0.69060800	6.04591200	-3.59485500
H	-0.62236500	5.34123800	-4.43615300
H	-0.58604600	5.46675100	-2.67088900
H	0.17398200	6.71560100	-3.66475500
C	-2.13483000	7.55178200	-5.00125600
H	-1.37288600	8.33338200	-5.09670000
H	-3.11305900	8.02233100	-5.14655200
H	-1.99127800	6.84869000	-5.83449100
N	-7.18265300	9.00203400	-3.17203000
H	-7.85108400	9.76655500	-3.11859300
C	-7.39371000	7.95521800	-4.02953600
O	-6.71816300	6.92690600	-3.95532800
C	-8.50020800	8.10893400	-5.10308500
C	-8.61808300	9.55664900	-5.62287300
H	-7.66314000	9.91424400	-6.02626300
H	-9.35359100	9.59299900	-6.43602300
H	-8.95080400	10.25452800	-4.84873700
C	-8.14938700	7.17209300	-6.27404100
H	-8.03340500	6.14189000	-5.93042400
H	-8.94327800	7.20999400	-7.02928700

H	-7.20927400	7.46996200	-6.75190500
C	-9.84242900	7.66297900	-4.47991900
H	-10.62761000	7.66297300	-5.24616900
H	-9.76406500	6.64790700	-4.07463600
H	-10.15489000	8.34073100	-3.68017400

11-int-*re*

C	-11.45494100	15.84170100	-1.09166600
C	-10.28977600	15.27053600	-0.57271600
C	-10.03511100	13.90342100	-0.70800300
C	-10.98734300	13.12818400	-1.38944900
C	-12.15294800	13.67720800	-1.91423600
C	-12.38559400	15.04490600	-1.76054700
H	-11.63259800	16.90686100	-0.97420600
H	-9.56501500	15.89563300	-0.05813700
H	-12.85445200	13.03326000	-2.43479000
H	-13.29298500	15.48375300	-2.16544300
C	-8.82888200	13.15724000	-0.20528100
H	-8.11820400	13.02429400	-1.03320100
C	-8.01431100	13.69745200	0.98601600
H	-8.60923500	14.39998900	1.57572000
C	-6.70301900	14.33430200	0.55989200
O	-6.14059600	13.98203800	-0.47361000
C	-6.05557000	15.35398000	1.45249300
C	-6.55045200	15.69516000	2.72150200
C	-4.89079400	15.98695300	0.98643000
C	-5.89573700	16.64725300	3.50368500
H	-7.43894200	15.21141100	3.11414600
C	-4.24311700	16.94294900	1.76260400
H	-4.51594800	15.70858200	0.00705700
C	-4.74466600	17.27569200	3.02505100
H	-6.28520800	16.89794700	4.48661900
H	-3.34694100	17.43004900	1.38758600
H	-4.23938100	18.02140300	3.63317200
O	-10.78749400	11.76484000	-1.63187300
C	-9.90396000	11.01793000	-0.90004200
O	-9.73505800	9.86162400	-1.22747300
C	-9.27372000	11.73942400	0.25469700
H	-10.12495600	11.90569800	0.95071500
C	-8.08287700	11.21800000	1.01649200
C	-7.69181600	12.39926800	1.87421200
P	-7.05394800	9.92206100	0.62847700
C	-6.61192400	9.77700300	-1.19131400
H	-5.68567400	10.34582100	-1.30092600
H	-7.38688000	10.34204400	-1.71287400
C	-6.40904800	8.42016000	-1.87893500
H	-5.80404700	7.75852200	-1.25391100

C	-5.60995000	8.58212900	-3.20847700
H	-5.51770700	7.57528500	-3.62477000
C	-6.27900100	9.49102000	-4.23637100
H	-6.31102300	10.52801800	-3.88574200
H	-7.30106300	9.15701800	-4.44273200
H	-5.71467500	9.47322900	-5.17430800
C	-7.65149700	8.25478800	1.17284100
C	-6.82162800	7.12165400	1.18975100
C	-8.97049500	8.13909400	1.62916100
C	-7.31197700	5.89587700	1.64168300
H	-5.78529800	7.18676200	0.87250300
C	-9.46351400	6.91040800	2.07280600
H	-9.59988700	9.02361700	1.64451400
C	-8.63445200	5.78640000	2.07949300
H	-6.65688600	5.02930800	1.65154600
H	-10.49023400	6.83364700	2.42109700
H	-9.01336300	4.83121100	2.43377400
C	-5.46999600	10.14693500	1.52721200
C	-5.27147500	9.55582400	2.78451500
C	-4.46903000	10.98088300	1.00284000
C	-4.09042400	9.78015000	3.49349400
H	-6.03725300	8.91553100	3.20892700
C	-3.28475200	11.19547100	1.70921200
H	-4.60958500	11.48935800	0.05474000
C	-3.09284900	10.59388500	2.95445100
H	-3.95136300	9.31577600	4.46609200
H	-2.51560200	11.83006100	1.27895200
H	-2.17044600	10.76197500	3.50413900
O	-4.32106400	9.12021600	-2.90937300
Si	-2.80711900	8.44346600	-2.66305900
C	-2.83775100	7.17676900	-1.24992900
C	-3.63593700	6.01500600	-1.32603000
C	-2.08830000	7.36678900	-0.07213800
C	-3.66250800	5.08107700	-0.28741100
H	-4.28537600	5.83957700	-2.17779600
C	-2.10810500	6.43305100	0.96649500
H	-1.48845700	8.26366200	0.04735900
C	-2.89016900	5.28203100	0.85876000
H	-4.29292800	4.20034200	-0.37901200
H	-1.51673200	6.60920800	1.86172000
H	-2.90462000	4.55242500	1.66491400
C	-1.77907600	9.95903500	-2.17744900
C	-2.34441100	11.24585900	-2.26242700
C	-0.43899200	9.87114500	-1.74995600
C	-1.61358600	12.38859200	-1.92805000
H	-3.37110900	11.35028700	-2.59947100
C	0.29755300	11.00920800	-1.41342700

H	0.04451100	8.90088000	-1.66776200
C	-0.28935700	12.27304200	-1.49994600
H	-2.07912700	13.36811300	-2.00575900
H	1.32894100	10.90799700	-1.08455900
H	0.28261300	13.16057200	-1.24073200
C	-2.12318400	7.68191000	-4.29801300
C	-2.97926600	6.49577200	-4.79354400
H	-2.56200700	6.10274500	-5.73256300
H	-4.01602300	6.78442700	-4.99991200
H	-2.99525600	5.66883000	-4.07577600
C	-0.68004300	7.17517100	-4.07882600
H	-0.29797000	6.72132200	-5.00491000
H	-0.62784700	6.41067900	-3.29388400
H	0.00449100	7.98747400	-3.81075600
C	-2.11160900	8.77598600	-5.38881500
H	-1.49050900	9.63244300	-5.10264300
H	-3.11979200	9.15233800	-5.59797800
H	-1.70733000	8.37022300	-6.32769700
N	-7.69110800	7.75852700	-2.12382700
H	-8.53635100	8.28544800	-1.94120200
C	-7.75957700	6.43141500	-2.42441300
O	-6.74271100	5.74280700	-2.55240200
C	-9.15467100	5.80821100	-2.67177300
C	-10.33365900	6.69744400	-2.24052300
H	-10.38004300	7.63826800	-2.79999000
H	-11.27541900	6.16678800	-2.42431300
H	-10.29335500	6.94035400	-1.17308700
C	-9.24078200	5.53032900	-4.19068300
H	-8.40561700	4.90206200	-4.51429600
H	-10.17951300	5.01586900	-4.42891400
H	-9.21294400	6.46351200	-4.76663800
C	-9.20676000	4.47182400	-1.90341500
H	-10.13252800	3.93496700	-2.14330000
H	-8.35321400	3.84397100	-2.17063100
H	-9.18115800	4.63959500	-0.82050700
H	-8.31389500	12.48677100	2.78109100
H	-6.65291000	12.38343900	2.21403800

11-int-*si*

C	-10.90030800	16.26879600	-0.43000500
C	-10.93740200	14.88501000	-0.62447400
C	-9.87914500	14.22257900	-1.24883300
C	-8.78461100	14.98932200	-1.67729400
C	-8.73121600	16.36769200	-1.49832400
C	-9.79985100	17.00769800	-0.86759400
H	-11.73238100	16.76694800	0.05917000
H	-11.79973100	14.31604100	-0.28895000

H	-7.86445700	16.91603700	-1.85293900
H	-9.76927100	18.08355800	-0.72073700
C	-9.77162400	12.74768400	-1.52325800
H	-10.00592200	12.57527900	-2.58428800
C	-10.58993000	11.72128400	-0.73453700
H	-10.49610700	11.92632600	0.33834500
C	-12.05540800	11.65491600	-1.14905400
O	-12.38705200	11.97789600	-2.28294300
C	-13.08284700	11.14816200	-0.17911900
C	-12.76482600	10.66252200	1.09931700
C	-14.42737500	11.15651100	-0.58828400
C	-13.76943200	10.19773300	1.94870200
H	-11.73377600	10.63256900	1.43618200
C	-15.42953900	10.69918100	0.26138100
H	-14.65668000	11.52781500	-1.58168700
C	-15.10216700	10.21833000	1.53335100
H	-13.51124300	9.81984000	2.93414300
H	-16.46599300	10.71418600	-0.06465400
H	-15.88384800	9.85939600	2.19766300
O	-7.71539300	14.40561600	-2.35829400
C	-7.46268300	13.05703300	-2.29164700
O	-6.58967200	12.62253400	-3.01221000
C	-8.30239100	12.31585700	-1.26920500
H	-7.99918300	12.78616100	-0.31133900
C	-8.36130400	10.79784900	-1.20414900
C	-9.84006600	10.39054300	-1.07640100
H	-10.04478900	9.62227500	-0.32242000
H	-10.25482900	10.00775700	-2.02235400
P	-7.11991300	9.82136300	-0.56782800
C	-5.48277200	10.05655500	-1.40956200
H	-4.69846800	9.76075500	-0.71006200
H	-5.39139300	11.13509900	-1.57183800
C	-5.23756300	9.28911200	-2.71830000
H	-5.50003600	8.23573500	-2.58311600
C	-3.72654300	9.28713800	-3.08990600
H	-3.65387700	8.72702300	-4.02706000
C	-3.12407300	10.67692500	-3.28436300
H	-3.11643200	11.24065900	-2.34558500
H	-3.70051800	11.24417200	-4.02280400
H	-2.09108200	10.59955800	-3.63714100
C	-7.63238900	8.06634000	-0.69379600
C	-7.57884900	7.18720700	0.39845200
C	-8.09929800	7.59021500	-1.93103500
C	-7.97928200	5.85711400	0.25322500
H	-7.23527100	7.53982500	1.36445200
C	-8.49267900	6.26014000	-2.07295000
H	-8.15202400	8.26478100	-2.77830700

C	-8.43342800	5.39103400	-0.98130400
H	-7.93855200	5.18762700	1.10827800
H	-8.83960900	5.90456800	-3.03873000
H	-8.74021200	4.35456200	-1.09300100
C	-6.62645500	10.04917100	1.22835800
C	-7.47148300	10.83153400	2.02701800
C	-5.46365300	9.50949600	1.80724000
C	-7.17115200	11.07396800	3.36943100
H	-8.37083600	11.25359300	1.58549100
C	-5.16421000	9.74637400	3.14962300
H	-4.79012300	8.88447700	1.22658500
C	-6.01509000	10.53198700	3.93218400
H	-7.83619700	11.68717400	3.97200100
H	-4.26473100	9.31665100	3.58250000
H	-5.77567000	10.72051900	4.97534800
O	-3.05557300	8.58081100	-2.04303500
Si	-1.80254100	7.46742200	-2.05015700
C	-1.96283900	6.68308300	-0.33607400
C	-3.22952400	6.39971000	0.21160800
C	-0.83327400	6.34178600	0.42920900
C	-3.36161500	5.79883100	1.46537100
H	-4.12643500	6.65497200	-0.34712200
C	-0.95832200	5.73915300	1.68339900
H	0.16156400	6.55864700	0.04701900
C	-2.22406600	5.46578700	2.20465200
H	-4.35196500	5.58863300	1.86233400
H	-0.06801000	5.48783100	2.25467400
H	-2.32393700	4.99729100	3.18067800
C	-0.16050200	8.41649100	-2.12149100
C	-0.10476200	9.68485900	-1.50826400
C	1.02960700	7.92734500	-2.69222400
C	1.07506400	10.42909100	-1.47096800
H	-1.00263600	10.09840700	-1.05663500
C	2.21535300	8.66582900	-2.65758300
H	1.04356800	6.95584400	-3.17722100
C	2.24084200	9.92026200	-2.04728600
H	1.08476200	11.40570100	-0.99320300
H	3.11727600	8.26093800	-3.10993400
H	3.16171100	10.49734200	-2.02103200
C	-1.99785700	6.12894700	-3.42501400
C	-3.46563800	5.63960200	-3.45784700
H	-3.55511800	4.79912500	-4.16170300
H	-4.16710000	6.41080300	-3.79166600
H	-3.79321100	5.27362400	-2.47701600
C	-1.10296200	4.91074900	-3.09063400
H	-1.22862400	4.13826000	-3.86293900
H	-1.37070100	4.46276700	-2.12757400

H	-0.03589100	5.15865600	-3.05397600
C	-1.61719400	6.66377100	-4.82451800
H	-0.58270400	7.02122900	-4.86963400
H	-2.26663500	7.48636800	-5.14430300
H	-1.72368900	5.86375400	-5.57136300
N	-6.04920600	9.80407000	-3.81428300
H	-6.42644000	10.74094300	-3.71379200
C	-6.33432400	9.02947100	-4.89737000
O	-5.90043100	7.87790400	-5.00136000
C	-7.20728200	9.63392300	-6.02347300
C	-7.82818700	10.99843900	-5.68036800
H	-7.07205700	11.77357000	-5.51627000
H	-8.46164000	11.33142800	-6.51076700
H	-8.45893100	10.94315600	-4.78528400
C	-6.29285600	9.77171300	-7.26193100
H	-5.84117000	8.80774600	-7.51374100
H	-6.87196600	10.12471300	-8.12362500
H	-5.48598700	10.49231700	-7.08058600
C	-8.32815700	8.61753700	-6.32682700
H	-8.90200100	8.93711200	-7.20475900
H	-7.90519600	7.62848100	-6.51934600
H	-9.02636100	8.53553200	-5.48419300

12

C	-1.16323500	0.33319700	0.00002500
C	0.23078200	0.32669900	0.00058300
C	0.93386800	1.54007200	0.00013300
C	0.23331100	2.75426800	-0.00087800
C	-1.15924500	2.75583000	-0.00144000
C	-1.85852500	1.54535300	-0.00098400
H	-1.70785000	-0.60689200	0.00038100
H	0.77724900	-0.60990800	0.00137000
H	0.79851000	3.68050600	-0.00121300
H	-1.70024300	3.69794900	-0.00223100
H	-2.94536500	1.54674700	-0.00142000
C	2.41910300	1.59511900	0.00069200
O	3.07912000	2.61578300	0.00022200
O	2.99901300	0.36647200	0.00174400
H	3.96017100	0.53197600	0.00203200

13-int

P	-0.22758200	-0.32569900	1.50797600
C	0.68662800	0.17596200	-0.07695600
H	1.68388500	0.54369300	0.18129600
H	0.14176700	0.99737500	-0.55326400
C	0.83656500	-1.00219700	-1.04789600
H	1.23947000	-1.86066900	-0.50705500

C	1.82444900	-0.72925600	-2.21682600
H	1.74148400	-1.59147600	-2.88810900
C	1.52733100	0.54331000	-3.00701400
H	1.71254800	1.43078700	-2.39289900
H	0.48447000	0.56613100	-3.33891900
H	2.17826700	0.60097400	-3.88557100
C	0.93963900	0.28991200	2.81085100
C	0.52579200	1.06726800	3.90436000
C	2.27205100	-0.16039900	2.78034300
C	1.42278500	1.40889000	4.91936000
H	-0.50060000	1.41628900	3.96621000
C	3.16708300	0.18523500	3.79235400
H	2.62053200	-0.79450300	1.96884100
C	2.74769000	0.97484900	4.86540200
H	1.08187500	2.01867200	5.75258400
H	4.19190700	-0.17230000	3.74049100
H	3.44513100	1.24289400	5.65464900
C	-1.58383000	0.94239600	1.56322800
C	-2.89962900	0.48637500	1.73285000
C	-1.37135000	2.32532600	1.41930200
C	-3.97738200	1.37749500	1.75012500
H	-3.07976100	-0.57940200	1.84853900
C	-2.44364600	3.22112100	1.44027000
H	-0.35979600	2.70585100	1.30529700
C	-3.75249200	2.74647600	1.60245000
H	-4.98893300	1.00205900	1.87920500
H	-2.26039700	4.28823700	1.33972700
H	-4.58606100	3.44347900	1.61768200
O	3.15202300	-0.63275800	-1.70577100
Si	4.42444300	-1.71732400	-1.55450900
C	4.24213100	-2.71983400	0.04754000
C	3.18177400	-3.63442700	0.23163500
C	5.13506100	-2.54404500	1.12345300
C	3.03195500	-4.34128100	1.42685600
H	2.44353700	-3.79220400	-0.54992900
C	4.99192400	-3.25537200	2.31784100
H	5.95376500	-1.83621600	1.03394900
C	3.93964700	-4.15852800	2.47192900
H	2.20060800	-5.03227300	1.53964500
H	5.70142300	-3.10060400	3.12738800
H	3.82308400	-4.71038200	3.40128000
C	5.91318800	-0.55207900	-1.41891500
C	5.74471000	0.82827000	-1.63696600
C	7.21609300	-1.00396900	-1.12653100
C	6.82208000	1.71471700	-1.56929000
H	4.75198400	1.20872100	-1.85644200
C	8.29717900	-0.12303700	-1.05597500

H	7.39733300	-2.06045500	-0.94467000
C	8.10243300	1.24149700	-1.27897100
H	6.66041000	2.77615800	-1.74179600
H	9.29002400	-0.50231800	-0.82665700
H	8.94230500	1.92974300	-1.22505200
C	4.59731300	-2.84518600	-3.11293100
C	3.54034600	-3.97190200	-3.16953900
H	3.64176800	-4.52228000	-4.11677200
H	2.50742900	-3.61490400	-3.10554400
H	3.68278700	-4.69469200	-2.35911800
C	5.99085800	-3.51643100	-3.11324800
H	6.07537400	-4.19180200	-3.97707100
H	6.15649700	-4.12156900	-2.21263000
H	6.80259000	-2.78560900	-3.18752100
C	4.48114600	-1.96825500	-4.38037500
H	5.20870700	-1.14768300	-4.37819900
H	3.48295300	-1.53005500	-4.49056600
H	4.67135500	-2.57668900	-5.27671100
N	-0.46592700	-1.42722400	-1.55791700
H	-1.16407200	-0.71639500	-1.74020600
C	-0.70095400	-2.73373700	-1.86596400
O	0.18008000	-3.59281400	-1.76506700
C	-2.11781700	-3.14062100	-2.33546000
C	-3.13438400	-1.98766700	-2.36716700
H	-2.84420000	-1.19556400	-3.06591200
H	-4.10995900	-2.36858600	-2.69125200
H	-3.27145500	-1.53445800	-1.37838400
C	-1.97026300	-3.74606400	-3.74841600
H	-1.23125200	-4.55175500	-3.74184200
H	-2.93046900	-4.14974200	-4.09134600
H	-1.64482000	-2.98929600	-4.47325600
C	-2.60777800	-4.23230800	-1.35845900
H	-3.57442300	-4.63026900	-1.69010300
H	-1.88573100	-5.05114600	-1.30809400
H	-2.73512000	-3.82756700	-0.34718200
C	-4.08982500	3.89604900	-5.70221200
C	-3.77359600	3.61810800	-4.37366900
C	-2.97499300	2.50734100	-4.06258400
C	-2.49751500	1.68226100	-5.09096900
C	-2.81573500	1.96389600	-6.41711500
C	-3.61239200	3.07077500	-6.72375600
H	-4.70869000	4.75621700	-5.94134200
H	-4.14058600	4.25386200	-3.57586200
H	-1.87837100	0.82887100	-4.83573500
H	-2.44331700	1.32318500	-7.21128800
H	-3.86052900	3.29023700	-7.75873500
C	-2.61470300	2.17333800	-2.65986600

O	-1.93820700	1.20911800	-2.33132000
O	-3.10812200	3.04515600	-1.76319700
H	-2.82968400	2.75774900	-0.86861100

14-ts

C	-10.58792600	15.23687900	-4.12732600
C	-10.54445600	15.48598600	-2.76078200
C	-10.49748200	14.44102100	-1.81640600
C	-10.49403800	13.12190700	-2.32192000
C	-10.54254700	12.86220100	-3.68967600
C	-10.58938200	13.91952800	-4.59563000
H	-10.63037000	16.06619300	-4.82732500
H	-10.57027200	16.51175500	-2.40631700
H	-10.53751700	11.83054600	-4.02353700
H	-10.62774700	13.71532100	-5.66167500
C	-10.46614800	14.68641100	-0.37562000
H	-10.67556000	13.84200500	0.27471600
C	-10.19533600	15.85445700	0.24196900
H	-9.95204000	16.74567000	-0.32600100
C	-10.16087700	15.92526100	1.72261000
O	-10.18766900	14.90322600	2.40977500
C	-10.07243000	17.26877700	2.39013900
C	-10.22746700	18.48659800	1.70913500
C	-9.84616200	17.29286700	3.77679500
C	-10.14818700	19.69799600	2.39705400
H	-10.43016800	18.50155900	0.64346100
C	-9.76018100	18.50075000	4.46200500
H	-9.74597300	16.34424300	4.29380700
C	-9.90965400	19.70830000	3.77237800
H	-10.27493000	20.63326200	1.85863200
H	-9.57980700	18.50465200	5.53362300
H	-9.84431500	20.65280900	4.30605000
O	-10.53736900	12.02041200	-1.47781800
C	-9.49890900	11.75609100	-0.60150400
O	-8.43431900	12.37982500	-0.63997800
C	-9.83814900	10.68577700	0.28030000
H	-10.70748000	10.08704300	0.02271500
C	-9.10317500	10.32931100	1.36797900
C	-9.13469900	9.94894400	2.63364300
H	-9.97988500	10.25464000	3.24872600
H	-8.35009500	9.37524700	3.11398100
P	-6.59223300	10.38432600	0.96618200
C	-5.52529500	10.26077900	-0.57971100
H	-4.47640900	10.36764300	-0.28684700
H	-5.79859600	11.13491500	-1.18035100
C	-5.65714500	8.96911900	-1.39451900
H	-5.59428100	8.10663300	-0.73149300

C	-4.49181300	8.77462000	-2.41055400
H	-4.73024500	7.85024900	-2.95045100
C	-4.32702900	9.90906100	-3.41841800
H	-3.99223700	10.82364600	-2.91761500
H	-5.26706700	10.12210600	-3.93468700
H	-3.57160100	9.64193900	-4.16421200
C	-5.91643800	9.10218500	2.11131800
C	-5.23142500	9.41377900	3.29817000
C	-6.18128900	7.74772600	1.82491100
C	-4.79598100	8.40142800	4.15593100
H	-5.03339500	10.44842500	3.55541700
C	-5.72749400	6.73979100	2.67505000
H	-6.74564700	7.46736900	0.93953900
C	-5.03339900	7.06309400	3.84286500
H	-4.26598600	8.66391800	5.06798700
H	-5.91421300	5.70181800	2.41767200
H	-4.68511100	6.27603100	4.50637800
C	-6.01313800	11.99167600	1.66802700
C	-6.95368200	12.92845200	2.12435900
C	-4.64706400	12.32026700	1.74260500
C	-6.54058100	14.15723300	2.64337300
H	-8.01335000	12.71331100	2.06788000
C	-4.23601900	13.54875800	2.26054000
H	-3.89322900	11.61258800	1.40917700
C	-5.18322800	14.47068000	2.71246500
H	-7.29039800	14.86430000	2.98598000
H	-3.17595000	13.78390100	2.31108200
H	-4.86241500	15.42830400	3.11453400
O	-3.27273900	8.62764900	-1.68342300
Si	-2.17479100	7.37998500	-1.45719600
C	-2.96808300	5.92523800	-0.53662000
C	-4.20979900	5.40416100	-0.95287200
C	-2.34604000	5.30356800	0.56368700
C	-4.79432000	4.30828700	-0.31494100
H	-4.76467600	5.86554100	-1.76299700
C	-2.91862200	4.19917000	1.19796500
H	-1.40724200	5.69223100	0.94674300
C	-4.14295000	3.69358200	0.75588800
H	-5.76171800	3.94923600	-0.65492900
H	-2.41057900	3.73821000	2.04177600
H	-4.58953900	2.83313200	1.24867900
C	-0.83440200	8.21689800	-0.41331100
C	-0.93596000	9.59035200	-0.12052300
C	0.29960300	7.53804500	0.07638500
C	0.03919100	10.25214700	0.62830200
H	-1.79109100	10.14557900	-0.49321500
C	1.27765600	8.19349400	0.82766600

H	0.43195300	6.47838900	-0.12522000
C	1.14940300	9.55486000	1.10702500
H	-0.06710600	11.31424500	0.83636600
H	2.13951500	7.64027000	1.19261800
H	1.90965800	10.06785600	1.69068100
C	-1.43677600	6.77781900	-3.13819500
C	-2.54006300	6.23941400	-4.07621500
H	-2.08952100	5.86105300	-5.00582500
H	-3.25813700	7.01622500	-4.36232700
H	-3.09726500	5.41078200	-3.62409700
C	-0.42269100	5.63818800	-2.88970000
H	-0.03731100	5.26679700	-3.85052900
H	-0.87467900	4.78793000	-2.36565800
H	0.44130500	5.97870300	-2.30828200
C	-0.70596000	7.94589000	-3.83526400
H	0.11596800	8.33263000	-3.22226400
H	-1.38065100	8.78236300	-4.05009100
H	-0.27874400	7.61049300	-4.79181100
N	-6.95086900	8.87716000	-2.07024400
H	-7.29823700	9.70633300	-2.54282300
C	-7.60131500	7.68273900	-2.17602200
O	-7.16528800	6.65831000	-1.63968100
C	-8.90577600	7.60799800	-3.00426700
C	-9.40024800	8.95564600	-3.55224300
H	-8.67187600	9.43139500	-4.21683000
H	-10.31829700	8.79399300	-4.13048900
H	-9.64009400	9.66046300	-2.74925700
C	-8.61759200	6.64785000	-4.18124500
H	-8.25157800	5.68652000	-3.81073600
H	-9.53192200	6.47781600	-4.76215700
H	-7.86192800	7.06609200	-4.85780200
C	-9.98747400	6.99046200	-2.09257900
H	-10.90576900	6.81108700	-2.66435700
H	-9.63968400	6.04272100	-1.67403100
H	-10.23131600	7.66286700	-1.26143700
C	-5.30010100	14.49559200	-7.23385000
C	-5.49735900	15.28249200	-6.09598100
C	-6.05002200	14.72235100	-4.94524000
C	-6.40501800	13.36577700	-4.92800000
C	-6.20726700	12.58090600	-6.07227400
C	-5.65710000	13.14425900	-7.22142500
H	-4.86896600	14.93499400	-8.12961700
H	-5.21996300	16.33302100	-6.10609000
H	-6.20963300	15.32422700	-4.05775300
H	-6.49372200	11.53495600	-6.04326500
H	-5.50551500	12.53227500	-8.10631700
C	-6.99647800	12.72891900	-3.71623600

O	-7.16733200	13.57265500	-2.70273700
H	-7.64416800	13.11188700	-1.95575400
O	-7.29148100	11.53716000	-3.66399200

15-int

C	-10.09037200	16.05534300	-3.81537700
C	-10.03802800	16.15453500	-2.43135200
C	-10.02453900	15.01190400	-1.60434900
C	-10.04057800	13.74857300	-2.24212200
C	-10.10678600	13.64627100	-3.63267700
C	-10.13291200	14.79410900	-4.42007700
H	-10.10102400	16.95525700	-4.42368200
H	-10.00628800	17.13803200	-1.97253100
H	-10.12255400	12.65524500	-4.07354000
H	-10.17749900	14.70602800	-5.50205600
C	-10.02939500	15.09558900	-0.14764300
H	-9.97305900	14.15784400	0.39709200
C	-10.12790300	16.21662500	0.60227100
H	-10.23293700	17.19127300	0.13925700
C	-10.13738800	16.13713000	2.07576600
O	-10.06659600	15.05667700	2.66841100
C	-10.24891900	17.40657000	2.87635900
C	-10.04137600	18.68805500	2.34169000
C	-10.55858900	17.28678600	4.24161400
C	-10.14498300	19.81973900	3.15174300
H	-9.77545000	18.81444100	1.29739500
C	-10.67256100	18.41556500	5.04749700
H	-10.70871200	16.29078000	4.64481900
C	-10.46574700	19.68723200	4.50380100
H	-9.97392700	20.80476900	2.72587700
H	-10.92223400	18.30746100	6.09968300
H	-10.55251800	20.57006400	5.13177800
O	-10.11025600	12.57942100	-1.52128600
C	-8.99220500	12.10630700	-0.80124500
O	-7.85814900	12.62173800	-1.00116000
C	-9.34960000	11.09210200	0.06769600
H	-10.40770600	10.86236200	0.13704900
C	-8.49710500	10.34705600	0.95409600
C	-8.97709400	9.46920400	1.87933900
H	-10.05062200	9.31536600	1.95454100
H	-8.35736700	8.90530600	2.55971700
P	-6.68347000	10.59423700	0.98206600
C	-5.78717900	10.39950700	-0.62634700
H	-4.72868500	10.51160700	-0.36665400
H	-6.09517300	11.25036000	-1.23653100
C	-5.95159700	9.06406800	-1.37231000
H	-5.92953200	8.23252700	-0.67066600

C	-4.76498700	8.77820700	-2.34225500
H	-5.03305800	7.84347200	-2.84842200
C	-4.51739000	9.85526400	-3.39220300
H	-4.19752900	10.79260800	-2.92393200
H	-5.42139800	10.04845700	-3.97493300
H	-3.72327700	9.53769200	-4.07514200
C	-5.92803600	9.38766600	2.13443000
C	-5.15801800	9.83491200	3.22013000
C	-6.07566300	8.00317700	1.92065300
C	-4.53648800	8.91726500	4.06812800
H	-5.04281200	10.89587800	3.40966200
C	-5.44550100	7.09419700	2.76988100
H	-6.69183000	7.62308600	1.11199900
C	-4.67429300	7.54816600	3.84150200
H	-3.94477900	9.27796900	4.90476800
H	-5.55174200	6.03111400	2.58020400
H	-4.18299800	6.83491200	4.49760100
C	-6.29414000	12.25219800	1.62052900
C	-7.26882900	12.96844700	2.32875400
C	-5.00863700	12.79560700	1.46303600
C	-6.95669100	14.21169900	2.88073300
H	-8.27479500	12.57688700	2.43329400
C	-4.70393300	14.03846400	2.01603600
H	-4.24039300	12.25634300	0.91761800
C	-5.67644300	14.74519200	2.72752100
H	-7.73051900	14.75795800	3.40900700
H	-3.70841400	14.45410100	1.88720600
H	-5.43676500	15.71480800	3.15573200
O	-3.58160700	8.60215500	-1.56163100
Si	-2.52813000	7.31710700	-1.31276900
C	-3.38562700	5.90376500	-0.38070800
C	-4.67475200	5.47056100	-0.75093400
C	-2.75999900	5.22191800	0.68144100
C	-5.30504300	4.40632600	-0.10203200
H	-5.23131700	5.97531900	-1.53352300
C	-3.37641400	4.14564600	1.32345900
H	-1.78138500	5.53962900	1.02838200
C	-4.65129400	3.73126500	0.93061200
H	-6.30814100	4.11955800	-0.40476300
H	-2.86280800	3.63505800	2.13477700
H	-5.13313900	2.89424400	1.43021900
C	-1.17024700	8.11454300	-0.26022100
C	-1.23936600	9.48743400	0.04336800
C	-0.05473800	7.40495100	0.22874400
C	-0.25241900	10.11970400	0.80202800
H	-2.07861900	10.06618200	-0.32946300
C	0.93513300	8.03040800	0.98978300

H	0.05509400	6.34474200	0.01754800
C	0.83830500	9.39206900	1.28011900
H	-0.33385800	11.18233100	1.01825000
H	1.78224800	7.45383000	1.35294200
H	1.60817900	9.88191800	1.87090300
C	-1.79928800	6.65350600	-2.97407600
C	-2.92105100	6.19093800	-3.93058400
H	-2.48186700	5.74849500	-4.83673700
H	-3.55701300	7.02099100	-4.25716400
H	-3.56262300	5.42757300	-3.47477200
C	-0.88163400	5.44096600	-2.69690200
H	-0.50023800	5.03952300	-3.64691900
H	-1.41173100	4.62909400	-2.18574600
H	-0.00931200	5.71385900	-2.09275700
C	-0.96780100	7.75735900	-3.66341700
H	-0.12887600	8.08329000	-3.03829200
H	-1.57063100	8.64299200	-3.89493800
H	-0.55187700	7.38417300	-4.61071800
N	-7.23392600	8.97785600	-2.05752700
H	-7.54164900	9.77387600	-2.60997800
C	-7.98461100	7.84030400	-1.97758700
O	-7.60356000	6.86280400	-1.32281300
C	-9.32768500	7.77660300	-2.73811300
C	-9.73468500	9.08508700	-3.43472400
H	-9.00902700	9.40086000	-4.19148500
H	-10.69527800	8.93691000	-3.94251700
H	-9.86348000	9.90528400	-2.72045300
C	-9.18503200	6.65016500	-3.78708600
H	-8.88310200	5.71415200	-3.30870800
H	-10.14114300	6.49145200	-4.29966400
H	-8.43537500	6.90718000	-4.54579500
C	-10.40771900	7.38295600	-1.70657400
H	-11.36880800	7.22798100	-2.21127200
H	-10.12731900	6.46241500	-1.18815900
H	-10.53995700	8.17292800	-0.95865400
C	-5.35324600	13.68186400	-8.01120000
C	-5.27175800	14.56442400	-6.93082500
C	-5.77361100	14.19247300	-5.68432500
C	-6.35861200	12.92983300	-5.51277300
C	-6.44051800	12.04912900	-6.59985300
C	-5.93985300	12.42402800	-7.84481900
H	-4.96152400	13.97426600	-8.98205800
H	-4.81743100	15.54300700	-7.06059300
H	-5.71952500	14.86985000	-4.83949000
H	-6.90216700	11.07881400	-6.44945900
H	-6.00594300	11.73816500	-8.68506800
C	-6.90480300	12.49560600	-4.19199500

O	-6.78240400	13.40576700	-3.23941900
H	-7.25381300	13.09513300	-2.39291200
O	-7.41122800	11.38580500	-4.02118900

16-ts-*re*

C	-13.18058400	12.27982400	3.53265400
C	-11.85108400	12.02244800	3.87222100
C	-10.81191900	12.13194700	2.93663500
C	-11.17274900	12.52200300	1.63373600
C	-12.49288300	12.78201000	1.27657200
C	-13.50317200	12.66150800	2.23085600
H	-13.95747300	12.18491300	4.28589800
H	-11.59280400	11.74324600	4.88859300
H	-12.70641700	13.08119100	0.25538900
H	-14.53328600	12.86652300	1.95359100
C	-9.39067700	11.84933100	3.31571400
H	-8.71985700	12.70594300	3.26401000
C	-9.10403800	10.94238200	4.35265900
H	-9.84724400	10.19735100	4.61579800
C	-7.85579100	10.96284000	5.03720700
O	-6.94020300	11.79095300	4.78203000
C	-7.61712900	9.98755700	6.16549100
C	-8.33649900	8.79379200	6.33367600
C	-6.60408800	10.29157000	7.08847500
C	-8.05591300	7.93487800	7.39684000
H	-9.10579900	8.51630300	5.61958000
C	-6.32845000	9.44016400	8.15663100
H	-6.04702300	11.21143200	6.94392000
C	-7.05414900	8.25658600	8.31539800
H	-8.61799100	7.01058000	7.50541600
H	-5.54702600	9.69848600	8.86733700
H	-6.83893300	7.58851700	9.14551300
O	-10.23547800	12.74153300	0.62358800
C	-9.02289100	12.11334000	0.61420500
O	-8.17061500	12.58536100	-0.15143100
C	-8.82734700	11.01691700	1.51609700
H	-9.67431400	10.33946900	1.57564600
C	-7.54211300	10.34066400	1.63488500
C	-7.46562400	9.15381500	2.28830100
P	-5.97162900	10.98090900	0.94870100
C	-5.85057200	10.62217700	-0.86097900
H	-4.78581300	10.63837100	-1.10893200
H	-6.32822400	11.46299800	-1.36805700
C	-6.46577800	9.29392700	-1.34747800
H	-6.38802400	8.51862500	-0.58065600
C	-5.72986700	8.73752800	-2.60361500
H	-6.29941500	7.84901100	-2.89196100

C	-5.67988000	9.71132700	-3.77894100
H	-5.01347100	10.55262800	-3.55897400
H	-6.67021500	10.11388800	-4.00775600
H	-5.29238900	9.20594400	-4.66900000
C	-4.60746500	10.12219100	1.81027400
C	-4.48178200	10.29335400	3.20249900
C	-3.65775400	9.36269500	1.11213900
C	-3.41060100	9.70625600	3.87311300
H	-5.21673500	10.86163400	3.77020600
C	-2.58473000	8.78623100	1.79583500
H	-3.74411300	9.19767200	0.04484900
C	-2.45943900	8.95815900	3.17361300
H	-3.32578700	9.83380100	4.94848900
H	-1.85685300	8.20174700	1.24179900
H	-1.62527200	8.50680100	3.70424200
C	-5.62602200	12.76293000	1.16385700
C	-5.93625700	13.41041000	2.36838000
C	-4.96920100	13.46672300	0.14173900
C	-5.60110300	14.75623200	2.53156400
H	-6.42202200	12.87899600	3.18506900
C	-4.63612500	14.80879800	0.31960700
H	-4.71598000	12.98559000	-0.79662300
C	-4.95510800	15.45803700	1.51351200
H	-5.84916500	15.25144600	3.46618600
H	-4.13122500	15.34533200	-0.47885000
H	-4.69806000	16.50521100	1.64867400
O	-4.39412100	8.37614800	-2.24123600
Si	-3.56622600	6.90734100	-2.22811100
C	-4.17617900	5.84577200	-0.78247700
C	-5.55885400	5.64439300	-0.58879600
C	-3.30095300	5.23634200	0.13762600
C	-6.04019900	4.86711300	0.46673000
H	-6.29292600	6.11325000	-1.23688500
C	-3.77769000	4.45007800	1.18859000
H	-2.22908100	5.38507700	0.04919200
C	-5.15051600	4.26120700	1.35507500
H	-7.11299800	4.74702500	0.59083700
H	-3.07596200	3.99297900	1.88209300
H	-5.52321500	3.65424600	2.17643800
C	-1.77436700	7.47389800	-1.97804300
C	-1.45643400	8.84390800	-2.05519300
C	-0.70684800	6.58009900	-1.75392200
C	-0.14572000	9.30157500	-1.90366500
H	-2.25146600	9.55916800	-2.24142700
C	0.60604800	7.03101100	-1.60044200
H	-0.89127600	5.51077800	-1.69931300
C	0.89046900	8.39571700	-1.67267000

H	0.06587800	10.36614000	-1.96712000
H	1.40576300	6.31543000	-1.42656600
H	1.91143700	8.74913400	-1.55351600
C	-3.71869900	5.94771000	-3.89677900
C	-5.18459600	5.59758200	-4.23616800
H	-5.21750700	4.97289000	-5.14077800
H	-5.79024400	6.48680500	-4.44334600
H	-5.67251100	5.03380100	-3.43314200
C	-2.92945100	4.62208800	-3.78137400
H	-3.03567400	4.04604300	-4.71183400
H	-3.29542300	3.99300200	-2.96150200
H	-1.85782000	4.79338400	-3.63053900
C	-3.11604300	6.78004400	-5.04931000
H	-2.06226900	7.02147500	-4.87049000
H	-3.65148700	7.72451600	-5.19905500
H	-3.17290700	6.21688500	-5.99219100
N	-7.88946700	9.46284400	-1.63088200
H	-8.19064700	10.35363500	-2.01634000
C	-8.73102500	8.38855800	-1.60042600
O	-8.33292000	7.26913400	-1.26634600
C	-10.21055800	8.58424300	-2.00764900
C	-10.60655300	10.03405100	-2.33082700
H	-10.03579800	10.44598700	-3.16931500
H	-11.66641600	10.06523300	-2.60942900
H	-10.47783200	10.70030700	-1.47074800
C	-10.43953500	7.69862000	-3.25403800
H	-10.14947000	6.66466800	-3.04828400
H	-11.49751800	7.71834800	-3.54131900
H	-9.85264300	8.05754200	-4.10859900
C	-11.07457400	8.05613800	-0.84219600
H	-12.13347200	8.05998600	-1.12620900
H	-10.78181700	7.03658700	-0.57910400
H	-10.96462600	8.68582800	0.04916200
H	-8.37673900	8.66989900	2.62474000
H	-6.53931700	8.62758700	2.47675800
C	-8.73133600	15.17420100	-7.19594100
C	-8.94772900	15.91897300	-6.03353200
C	-8.83630600	15.31247800	-4.78322900
C	-8.50632500	13.95245800	-4.69184100
C	-8.29055400	13.20943200	-5.86051100
C	-8.40289800	13.81836100	-7.10818100
H	-8.81897600	15.64969100	-8.16935400
H	-9.20362900	16.97271800	-6.10186000
H	-9.00232800	15.88151600	-3.87537400
H	-8.03608800	12.15864000	-5.77133300
H	-8.23494400	13.23866000	-8.01166500
C	-8.37368600	13.26809800	-3.37346500

O	-8.60188100	14.06336600	-2.33539100
H	-8.49509300	13.54623600	-1.48325500
O	-8.07752600	12.07772700	-3.26993600

16-ts-*si*

C	-9.76741400	17.14658100	1.59991500
C	-9.51742000	15.90536100	2.18964300
C	-9.33888700	14.74808600	1.42043600
C	-9.43203100	14.89169100	0.02475200
C	-9.68105800	16.11872800	-0.58186900
C	-9.84999600	17.25416000	0.21179900
H	-9.90133000	18.02371300	2.22660100
H	-9.47271100	15.81346400	3.27011900
H	-9.74479300	16.16354300	-1.66437400
H	-10.04632300	18.21426400	-0.25692800
C	-9.06736900	13.40949600	2.03468900
H	-9.85672200	12.67767400	1.86396500
C	-8.47470400	13.30905800	3.31292900
H	-7.89606300	14.14116600	3.69870500
C	-8.59372500	12.10545900	4.06320900
O	-9.21920400	11.09772800	3.63822600
C	-7.99088100	12.02664400	5.44669000
C	-6.99195900	12.89293600	5.91818200
C	-8.45433700	11.01580800	6.30334900
C	-6.47755500	12.75602900	7.20806300
H	-6.59297300	13.66868100	5.27143600
C	-7.94850400	10.88204100	7.59509000
H	-9.22105900	10.34664800	5.92654300
C	-6.95635800	11.75272400	8.05361100
H	-5.69869500	13.43223300	7.55216700
H	-8.32858000	10.09897700	8.24701500
H	-6.55756900	11.64918700	9.05962300
O	-9.35001300	13.80017100	-0.84192500
C	-8.64797200	12.67425400	-0.52205700
O	-8.85330800	11.68322200	-1.23581700
C	-7.79732900	12.73816700	0.63851800
H	-7.22311800	13.66056500	0.68529000
C	-7.03991900	11.58920800	1.13203200
C	-6.09984200	11.80611000	2.08758800
H	-5.86266900	12.82311400	2.38157900
H	-5.53936400	11.01876700	2.57320100
P	-7.19485600	9.86693400	0.52396100
C	-6.26225900	9.92204000	-1.07494100
H	-5.24239000	10.16193200	-0.75426700
H	-6.65902400	10.80533600	-1.58120800
C	-6.21448700	8.73328100	-2.05338600
H	-6.15919500	7.77801900	-1.52810100

C	-4.93588400	8.78116600	-2.94130200
H	-5.10846800	8.02302000	-3.71285000
C	-4.68559500	10.13232400	-3.61229900
H	-4.40770600	10.89516400	-2.87818900
H	-5.57796100	10.47330200	-4.14691100
H	-3.86435900	10.05898100	-4.33169700
C	-8.85396700	9.11710200	0.42800400
C	-9.95995900	9.69739200	1.06120000
C	-8.97715900	7.85612300	-0.17963500
C	-11.18993300	9.03705800	1.04524300
H	-9.85119700	10.60534100	1.64175500
C	-10.21068500	7.20672200	-0.18872900
H	-8.13039500	7.36015500	-0.63934000
C	-11.32256300	7.80117900	0.41306000
H	-12.03993900	9.49103400	1.54642500
H	-10.29284100	6.23187700	-0.66085400
H	-12.28231400	7.29126500	0.40654500
C	-6.30479100	8.81017100	1.71259300
C	-6.87073100	8.66776300	2.99442000
C	-5.10130700	8.16299700	1.39900600
C	-6.22426700	7.88591300	3.94894700
H	-7.78738900	9.19264700	3.25797400
C	-4.46756400	7.37589000	2.36454300
H	-4.64684700	8.25900300	0.41760100
C	-5.02623600	7.23777800	3.63496800
H	-6.65533000	7.79274700	4.94150800
H	-3.53629700	6.87786100	2.11453800
H	-4.52610200	6.62908500	4.38367300
O	-3.83363300	8.36982700	-2.12353800
Si	-2.29674000	7.87667800	-2.61428500
C	-1.64324900	6.90074600	-1.13531200
C	-2.45359700	5.95999600	-0.47015300
C	-0.30896000	7.02834900	-0.70705500
C	-1.95011500	5.17293100	0.56852200
H	-3.49400400	5.84307500	-0.76278700
C	0.19884300	6.24819700	0.33446900
H	0.34409300	7.75212600	-1.18850000
C	-0.62033200	5.31565400	0.97286000
H	-2.59494100	4.44792200	1.05935200
H	1.23284900	6.36953100	0.64742800
H	-0.22613200	4.70388100	1.78033200
C	-1.23105500	9.43184300	-2.82025300
C	-1.39114800	10.45338300	-1.86085300
C	-0.26925500	9.64134800	-3.82529600
C	-0.63377700	11.62395400	-1.90361800
H	-2.12310900	10.33081700	-1.06593300
C	0.49244000	10.81203600	-3.87610500

H	-0.10201800	8.88718700	-4.58821800
C	0.31200500	11.80689400	-2.91511500
H	-0.78090300	12.39285500	-1.14925000
H	1.22591000	10.94478600	-4.66745100
H	0.90367400	12.71792800	-2.95285600
C	-2.35037400	6.72138800	-4.16078400
C	-3.38037300	5.58956900	-3.92114500
H	-3.40597300	4.92451700	-4.79647000
H	-4.40047900	5.95541700	-3.75992700
H	-3.10876100	4.97385600	-3.05652600
C	-0.96451700	6.05486400	-4.34679400
H	-1.00588100	5.34700800	-5.18704800
H	-0.65929300	5.49372200	-3.45714600
H	-0.17099400	6.77626400	-4.57187200
C	-2.72033500	7.46665400	-5.46325300
H	-2.04171000	8.29928300	-5.67775000
H	-3.73868600	7.86821500	-5.43721500
H	-2.67029800	6.77376700	-6.31559300
N	-7.40108300	8.67922600	-2.89627600
H	-7.73311600	9.54837300	-3.30972400
C	-7.86591000	7.47134100	-3.34590100
O	-7.38881700	6.41284600	-2.92919000
C	-8.99768000	7.49938100	-4.40008600
C	-8.41537300	8.03979300	-5.72792400
H	-7.57187100	7.42549400	-6.06523000
H	-9.18498600	8.00967300	-6.50863900
H	-8.07472300	9.07502100	-5.63144700
C	-9.49926800	6.06085400	-4.61103200
H	-9.91510400	5.64635100	-3.68709900
H	-10.28353100	6.05186400	-5.37712500
H	-8.68845300	5.40071600	-4.92983000
C	-10.17385700	8.38823600	-3.94096200
H	-10.96894900	8.36146900	-4.69588300
H	-10.59447700	8.03311000	-2.99454500
H	-9.88504400	9.43421500	-3.80666100
C	-8.30105900	12.97564000	-8.20065200
C	-9.36994200	13.84471000	-8.43749500
C	-10.27566800	14.13651700	-7.41409600
C	-10.11618000	13.56197700	-6.15411000
C	-9.04677100	12.68661400	-5.91454400
C	-8.13983200	12.39786000	-6.94358900
H	-7.59560700	12.74984200	-8.99549400
H	-9.49641900	14.29475500	-9.41867900
H	-11.10606800	14.81251200	-7.59826200
H	-10.81223000	13.78339800	-5.35281900
H	-7.31560400	11.72276800	-6.73960700
C	-8.84329300	12.05271600	-4.58174900

O	-9.75159700	12.40308000	-3.67880100
H	-9.49694700	12.04567400	-2.77825300
O	-7.92160000	11.27032600	-4.34812300

17-int-*re*

C	-12.82179300	13.20200600	3.98568000
C	-11.57995700	12.58597400	4.15142000
C	-10.65922800	12.50763600	3.09945300
C	-11.04401200	13.08518500	1.88692100
C	-12.27378900	13.70362700	1.68943000
C	-13.17322400	13.75822800	2.75377700
H	-13.51541300	13.24610900	4.82061600
H	-11.29644300	12.15606500	5.10664900
H	-12.50322200	14.13462600	0.72014000
H	-14.13937900	14.23586700	2.61892000
C	-9.29214900	11.85635600	3.23310900
H	-8.54172400	12.63528200	3.42131300
C	-9.17887600	10.85905000	4.32607100
H	-10.03258300	10.22597000	4.54322400
C	-8.00756900	10.78770800	5.06758400
O	-6.98768900	11.54033700	4.85752000
C	-7.89658200	9.79823000	6.20434500
C	-8.71955800	8.66892200	6.34382900
C	-6.90052400	10.01306200	7.16971600
C	-8.56103800	7.79297200	7.41804400
H	-9.47881600	8.45749300	5.59623300
C	-6.74317600	9.14304300	8.24818100
H	-6.26108800	10.88107500	7.04618100
C	-7.57386100	8.02749100	8.37866400
H	-9.20558200	6.92081200	7.50186900
H	-5.97050200	9.33476200	8.98961600
H	-7.45065000	7.34474600	9.21587300
O	-10.16334300	13.12875900	0.79198200
C	-9.09624200	12.32033000	0.72228700
O	-8.31480800	12.49058100	-0.20771000
C	-8.95767100	11.25414200	1.77037600
H	-9.79329400	10.56398100	1.58091900
C	-7.68701700	10.43676300	1.70972700
C	-7.72498300	9.18222500	2.20075100
P	-6.07553000	11.00557700	1.07994600
C	-5.90684000	10.67641500	-0.73404400
H	-4.83985400	10.73952400	-0.96391200
H	-6.40862300	11.49622100	-1.24933800
C	-6.46154100	9.32459800	-1.22843100
H	-6.34705700	8.55064600	-0.46513000
C	-5.70647300	8.79975700	-2.48740600
H	-6.24565700	7.89211700	-2.77747300

C	-5.69365500	9.77594000	-3.66159000
H	-5.06381200	10.64393000	-3.43704100
H	-6.69824100	10.13571200	-3.89848800
H	-5.27817600	9.28760700	-4.54846900
C	-4.76072100	10.07305700	1.94232500
C	-4.73362500	10.07857100	3.35004000
C	-3.73827500	9.43098300	1.22509200
C	-3.68597100	9.44369400	4.01621700
H	-5.51865800	10.56660500	3.93337400
C	-2.69302200	8.80656400	1.90771100
H	-3.74626600	9.38649500	0.14319100
C	-2.66489400	8.81285300	3.30203100
H	-3.67814100	9.44477400	5.10250200
H	-1.91082400	8.31281900	1.33963100
H	-1.85146700	8.32322600	3.83117800
C	-5.74117700	12.78094800	1.34449100
C	-5.93236000	13.33797100	2.61985600
C	-5.22413400	13.57066400	0.30437600
C	-5.61749800	14.68210700	2.83313900
H	-6.32724000	12.73844100	3.44389300
C	-4.90783700	14.90863800	0.53514600
H	-5.06744900	13.15960200	-0.68668400
C	-5.10655300	15.46759700	1.79927200
H	-5.77397300	15.10900700	3.81976000
H	-4.51039800	15.51280400	-0.27557800
H	-4.86205400	16.51171600	1.97567300
O	-4.35891400	8.48706200	-2.12957100
Si	-3.46958900	7.05372100	-2.14000900
C	-4.03828100	5.94218200	-0.71617000
C	-5.41297300	5.69413500	-0.52251400
C	-3.14179300	5.34288300	0.18992100
C	-5.86796500	4.88436500	0.52003100
H	-6.16251200	6.15178300	-1.16037700
C	-3.59117900	4.52291700	1.22702700
H	-2.07532100	5.52721600	0.10257400
C	-4.95716500	4.28985900	1.39450800
H	-6.93636200	4.73153500	0.64552400
H	-2.87376200	4.07496400	1.91030700
H	-5.30848000	3.65828400	2.20657600
C	-1.70446300	7.69046400	-1.87606200
C	-1.44502500	9.07432700	-1.91582200
C	-0.60008000	6.83744600	-1.67249500
C	-0.15564400	9.58343600	-1.74642500
H	-2.26870300	9.76044000	-2.08714700
C	0.69181500	7.33985000	-1.50190300
H	-0.73858700	5.76013600	-1.64719000
C	0.91771700	8.71688700	-1.53550800

H	0.01011600	10.65742800	-1.78006400
H	1.52091900	6.65465900	-1.34416300
H	1.92213800	9.11024200	-1.40200700
C	-3.58310000	6.11705400	-3.82523800
C	-5.03596800	5.72608200	-4.17594300
H	-5.04642100	5.11468200	-5.09010000
H	-5.66800700	6.59945600	-4.37149000
H	-5.50833500	5.13473500	-3.38329700
C	-2.75258200	4.81545600	-3.72734100
H	-2.83327900	4.25355400	-4.66894300
H	-3.10374000	4.16018900	-2.92176400
H	-1.68818400	5.01844100	-3.56500500
C	-3.00217000	6.98617900	-4.96152200
H	-1.95571600	7.25290600	-4.77593300
H	-3.56268500	7.91846200	-5.09510800
H	-3.04203200	6.43899000	-5.91462200
N	-7.89240800	9.42664400	-1.50464000
H	-8.23140400	10.28236900	-1.93424700
C	-8.70022600	8.33318600	-1.37979300
O	-8.26251000	7.25432000	-0.97136700
C	-10.19154500	8.45595600	-1.77232500
C	-10.64859200	9.87825800	-2.13466700
H	-10.12252500	10.27852100	-3.00709900
H	-11.71842100	9.86596000	-2.37305300
H	-10.50860600	10.57968300	-1.30411900
C	-10.40108700	7.52213100	-2.98677300
H	-10.07371200	6.50600900	-2.74980200
H	-11.46168900	7.49492800	-3.26337300
H	-9.83478400	7.87218000	-3.85853200
C	-11.01952500	7.93203900	-0.57950600
H	-12.08097700	7.88407800	-0.84910700
H	-10.68204100	6.93505400	-0.28631100
H	-10.92514300	8.59347800	0.29109700
H	-8.66565800	8.74885600	2.52215500
H	-6.84868300	8.55178300	2.29884400
C	-8.88845200	14.88313000	-7.36220700
C	-9.07036600	15.67945800	-6.22839600
C	-8.95607700	15.12140700	-4.95614700
C	-8.65747200	13.75824100	-4.81460800
C	-8.47631400	12.96307200	-5.95478900
C	-8.59166800	13.52412500	-7.22427600
H	-8.97832900	15.32115500	-8.35276900
H	-9.30152100	16.73552800	-6.33625800
H	-9.09561600	15.73125700	-4.07059700
H	-8.24670900	11.91057400	-5.82686700
H	-8.45069300	12.90476800	-8.10566300
C	-8.52272800	13.12433900	-3.47381100

O	-8.72117000	13.96630500	-2.46134200
H	-8.61839100	13.47191200	-1.60313000
O	-8.25104000	11.93537400	-3.31804500

17-int-*si*

C	-11.24914200	16.53453600	1.84129200
C	-10.47684300	15.48504400	2.34300300
C	-10.04146800	14.44240000	1.51639200
C	-10.43000300	14.50087800	0.17549000
C	-11.19681000	15.53290700	-0.35422300
C	-11.60721800	16.56297900	0.49171900
H	-11.57173600	17.33011400	2.50699000
H	-10.20443100	15.45063500	3.39276900
H	-11.46481700	15.51009000	-1.40562900
H	-12.20636300	17.37836700	0.09679000
C	-9.21770100	13.26584400	2.00873800
H	-9.88898200	12.41497700	2.17987700
C	-8.46564200	13.49684400	3.26532100
H	-8.07488900	14.48704600	3.47291000
C	-8.31725800	12.44269000	4.15889900
O	-8.78955600	11.26974900	3.94075800
C	-7.59161200	12.65342100	5.46673700
C	-6.70898800	13.71772300	5.71151600
C	-7.80164700	11.71958500	6.49344000
C	-6.06861000	13.85046500	6.94401000
H	-6.50235900	14.43987100	4.92658400
C	-7.16823500	11.85254700	7.72867800
H	-8.47616600	10.89385500	6.29124100
C	-6.29757000	12.92005800	7.96099600
H	-5.38408500	14.67951500	7.10913400
H	-7.35389400	11.12207300	8.51315500
H	-5.79870500	13.02424300	8.92145000
O	-10.11267200	13.46420300	-0.71705300
C	-9.12370500	12.59144400	-0.46393400
O	-8.96181000	11.67833500	-1.26717900
C	-8.28153300	12.84424000	0.75172300
H	-7.74633700	13.77753600	0.52061000
C	-7.22678600	11.81068700	1.07668800
C	-6.15318100	12.24458300	1.76862700
H	-6.02776800	13.30164100	1.97508500
H	-5.38571800	11.58348400	2.15429700
P	-7.21594800	10.03913400	0.61879400
C	-6.33379800	10.04766500	-1.01898500
H	-5.31011800	10.29399800	-0.71689400
H	-6.73445200	10.92404200	-1.53205800
C	-6.29360300	8.85016000	-1.99133000
H	-6.28989800	7.89494000	-1.46303300

C	-4.98757400	8.84230900	-2.84224200
H	-5.18017900	8.11189800	-3.63495000
C	-4.63749700	10.18793300	-3.47930600
H	-4.35502200	10.92765900	-2.72352800
H	-5.48557300	10.58018000	-4.05009300
H	-3.78738600	10.07989800	-4.15955200
C	-8.83515700	9.20477500	0.66979000
C	-9.78332700	9.61208900	1.62221800
C	-9.07845600	8.06925300	-0.12043500
C	-10.99065700	8.91805700	1.72887800
H	-9.55996100	10.40866400	2.33310900
C	-10.28567400	7.38250600	0.00523800
H	-8.34339800	7.69158600	-0.81721800
C	-11.25145600	7.81485400	0.91652100
H	-11.71845300	9.23979300	2.46847900
H	-10.46004500	6.50232000	-0.60718800
H	-12.19200700	7.27799500	1.00830300
C	-6.15413600	9.17742700	1.82868100
C	-6.51307000	9.24089900	3.18893000
C	-5.02605100	8.44356500	1.42895600
C	-5.73170300	8.57676500	4.13298200
H	-7.36820000	9.83507100	3.51941500
C	-4.25832800	7.77857400	2.38854900
H	-4.72679600	8.37984200	0.38764600
C	-4.60920500	7.84515800	3.73693000
H	-6.00120200	8.64596800	5.18303000
H	-3.38822100	7.21255100	2.07264700
H	-4.00453800	7.33133200	4.47983000
O	-3.93775400	8.35052800	-2.00372400
Si	-2.42805200	7.74141900	-2.45022500
C	-1.92675900	6.67020000	-0.97856700
C	-2.84183500	5.77632900	-0.38875600
C	-0.61046000	6.67851000	-0.48128600
C	-2.45613700	4.91995100	0.64511600
H	-3.87200000	5.75419000	-0.73474400
C	-0.21946900	5.82720200	0.55487200
H	0.12014100	7.36377800	-0.90423700
C	-1.14164200	4.94321100	1.11825600
H	-3.18138900	4.23826000	1.08231400
H	0.80313600	5.85546800	0.92270300
H	-0.83922000	4.27731500	1.92252600
C	-1.23702300	9.21244100	-2.56464100
C	-1.38561600	10.23838900	-1.60816700
C	-0.19378100	9.35195500	-3.49813800
C	-0.53913200	11.34689300	-1.58571400
H	-2.17986700	10.16857300	-0.86877200
C	0.65692600	10.46065300	-3.48386300

H	-0.03173500	8.59050800	-4.25492200
C	0.48624900	11.46151100	-2.52726600
H	-0.67871100	12.12080000	-0.83502300
H	1.45219500	10.54042900	-4.22073800
H	1.14726800	12.32427500	-2.51410900
C	-2.50855900	6.63939700	-4.03482300
C	-3.63910300	5.59222100	-3.87989300
H	-3.68281300	4.96299000	-4.78058800
H	-4.63168500	6.03477600	-3.74222100
H	-3.45441800	4.92655500	-3.02981200
C	-1.17726500	5.86163000	-4.18292400
H	-1.24055100	5.18525000	-5.04740200
H	-0.96228600	5.25016800	-3.30017400
H	-0.31538300	6.51726500	-4.34983800
C	-2.75071000	7.45558800	-5.32514900
H	-1.99187700	8.23060400	-5.48037300
H	-3.73010000	7.94584000	-5.32990800
H	-2.72202500	6.78826800	-6.19865700
N	-7.45607500	8.83769100	-2.87169000
H	-7.74677000	9.71868100	-3.28999400
C	-7.93159400	7.64658400	-3.35529400
O	-7.48312100	6.57358800	-2.94464100
C	-9.05143400	7.70997700	-4.41975100
C	-8.45652700	8.26906700	-5.73378900
H	-7.62733300	7.64344300	-6.08510500
H	-9.22578800	8.27478700	-6.51536700
H	-8.09018600	9.29306100	-5.61450600
C	-9.56980600	6.28223500	-4.66180800
H	-9.99792200	5.85540900	-3.74926100
H	-10.34762800	6.29785200	-5.43423800
H	-8.76487500	5.61794400	-4.98666000
C	-10.21857800	8.60228400	-3.94381800
H	-11.02349300	8.58258800	-4.68825300
H	-10.62940100	8.24559400	-2.99303100
H	-9.92378000	9.64657900	-3.80863700
C	-7.90602400	13.36433800	-8.10165800
C	-8.97364700	14.22020400	-8.38753600
C	-9.97525500	14.43398200	-7.43675800
C	-9.91286600	13.79468000	-6.19981000
C	-8.84436200	12.93278900	-5.91144700
C	-7.84096000	12.72194100	-6.86773900
H	-7.12685400	13.19911900	-8.84043800
H	-9.02467800	14.72076500	-9.35081600
H	-10.80447100	15.09953700	-7.65959900
H	-10.68433700	13.95576900	-5.45520600
H	-7.02011500	12.05510500	-6.62632600
C	-8.74136600	12.22994500	-4.60457800

O	-9.73907200	12.50466900	-3.76553700
H	-9.55522100	12.07930800	-2.88461500
O	-7.82557800	11.45755100	-4.32540800

18-ts-*re*

C	-12.94830800	12.41350600	4.06582300
C	-11.64124300	11.93423800	4.18173500
C	-10.72319200	12.07243800	3.13575800
C	-11.17209000	12.71951100	1.97661100
C	-12.46591100	13.20794200	1.83617100
C	-13.36229200	13.04882900	2.89368400
H	-13.64125300	12.29184400	4.89350500
H	-11.31359800	11.44963500	5.09620200
H	-12.74983800	13.70473500	0.91403000
H	-14.37745600	13.42324600	2.79928000
C	-9.28644600	11.60061900	3.16919700
H	-8.64226000	12.46492000	3.37683900
C	-8.91518600	10.53895900	4.15435200
H	-9.66941900	9.79978400	4.40455900
C	-7.85411900	10.78923200	5.06650000
O	-7.05673400	11.75795500	4.92779900
C	-7.62125400	9.84846900	6.22471300
C	-8.23167700	8.59009200	6.35026700
C	-6.73532700	10.26261300	7.23152600
C	-7.96766800	7.77489500	7.45129300
H	-8.90473800	8.22923800	5.57851300
C	-6.47621500	9.45346200	8.33631500
H	-6.26281400	11.23286700	7.12054000
C	-7.09187800	8.20455200	8.45113000
H	-8.44431600	6.80057300	7.52652400
H	-5.79278600	9.79604900	9.10963200
H	-6.88964900	7.56983200	9.31020400
O	-10.30777800	12.96903900	0.89521200
C	-9.17655000	12.25624700	0.72015700
O	-8.44153400	12.55831800	-0.20905800
C	-8.94141200	11.12931700	1.67569300
H	-9.72351300	10.38788700	1.43418200
C	-7.61452500	10.43777400	1.71237100
C	-7.68285600	9.37168100	2.60664000
P	-6.08097600	11.02735200	1.08245400
C	-5.89932300	10.68467200	-0.73560300
H	-4.83136600	10.72105100	-0.96849900
H	-6.37844300	11.51678000	-1.25608300
C	-6.49089700	9.35035600	-1.23380900
H	-6.41872400	8.57758600	-0.46361500
C	-5.74112000	8.79680900	-2.48347900
H	-6.30828000	7.90997600	-2.78184700

C	-5.67781900	9.77513200	-3.65458000
H	-5.01186200	10.61376100	-3.42357000
H	-6.66487900	10.18023000	-3.89284300
H	-5.28203200	9.27282400	-4.54277500
C	-4.71254200	10.17049900	1.94552300
C	-4.55532400	10.36470100	3.33078200
C	-3.79093400	9.37469000	1.24965700
C	-3.48803400	9.76465200	3.99702000
H	-5.26831500	10.95886700	3.89770700
C	-2.72049000	8.78557800	1.92642700
H	-3.89546100	9.19420600	0.18686700
C	-2.56713900	8.97977300	3.29838500
H	-3.38244000	9.91213900	5.06808200
H	-2.01540400	8.17429500	1.37181200
H	-1.73515800	8.51847400	3.82400500
C	-5.74672000	12.82598400	1.26287200
C	-6.03771800	13.46041800	2.48111100
C	-5.15513500	13.56337800	0.22451200
C	-5.74844800	14.81702400	2.64351700
H	-6.47530100	12.90495900	3.30907200
C	-4.86772700	14.91709500	0.39801800
H	-4.91374200	13.09702800	-0.72408200
C	-5.16733200	15.54814200	1.60677200
H	-5.98018300	15.29802900	3.58977600
H	-4.41204900	15.47699500	-0.41409700
H	-4.94579200	16.60390100	1.73899900
O	-4.40927900	8.43022400	-2.11145700
Si	-3.57055200	6.96912400	-2.13185400
C	-4.18696900	5.86054800	-0.72520300
C	-5.57023900	5.65223700	-0.54463800
C	-3.31501100	5.21870100	0.17570300
C	-6.05520300	4.83562400	0.47897700
H	-6.30142700	6.14671800	-1.17674400
C	-3.79554400	4.39439900	1.19526300
H	-2.24285300	5.37154100	0.09702800
C	-5.16885600	4.19806100	1.34814800
H	-7.12843700	4.71069800	0.59466000
H	-3.09649100	3.91311100	1.87498200
H	-5.54463000	3.56102900	2.14499800
C	-1.78533700	7.53857300	-1.84537900
C	-1.47904300	8.91318500	-1.85902800
C	-0.71190600	6.64487700	-1.65147200
C	-0.17368200	9.37473600	-1.67688900
H	-2.27895000	9.62932700	-2.01828800
C	0.59584200	7.09958500	-1.46802900
H	-0.88763700	5.57275600	-1.64386500
C	0.86878400	8.46849400	-1.47811600

H	0.02865400	10.44288400	-1.69047600
H	1.40038800	6.38377300	-1.31883900
H	1.88567200	8.82492400	-1.33509900
C	-3.70002200	6.05405800	-3.82805000
C	-5.16210700	5.71750300	-4.19661300
H	-5.18422600	5.11433000	-5.11611900
H	-5.76203600	6.61339000	-4.39123600
H	-5.66316200	5.13663400	-3.41394700
C	-2.91684700	4.72277900	-3.73858300
H	-3.01150800	4.17347600	-4.68640500
H	-3.29656700	4.07233300	-2.94208000
H	-1.84682300	4.88543200	-3.56792500
C	-3.07870900	6.91498400	-4.94924500
H	-2.02588300	7.14479500	-4.75054700
H	-3.60645700	7.86675400	-5.07805500
H	-3.12713000	6.37937600	-5.90857900
N	-7.91389500	9.50092900	-1.54053200
H	-8.22185600	10.38848700	-1.92532300
C	-8.74543000	8.42021000	-1.51916700
O	-8.34495000	7.30442000	-1.17534400
C	-10.22038700	8.59967000	-1.95277500
C	-10.63283900	10.04733500	-2.26412700
H	-10.05751900	10.47762900	-3.08990400
H	-11.68994400	10.06907900	-2.55367100
H	-10.52106500	10.70468400	-1.39444900
C	-10.41106500	7.72896900	-3.21601100
H	-10.10946200	6.69672000	-3.01837800
H	-11.46298600	7.73679400	-3.52543700
H	-9.81164000	8.10856300	-4.05265200
C	-11.09924700	8.04172500	-0.81294400
H	-12.15212900	8.02973100	-1.11837100
H	-10.79238500	7.02502300	-0.55560300
H	-11.01975800	8.66111700	0.08951900
H	-8.56422000	8.73740200	2.59877400
H	-6.79471200	8.89106700	3.00462300
C	-8.56794900	15.15653000	-7.30235700
C	-8.88531300	15.90419000	-6.16525400
C	-8.85454900	15.30833200	-4.90537700
C	-8.50375200	13.95623900	-4.77946600
C	-8.18585600	13.21018700	-5.92268200
C	-8.21836100	13.80862100	-7.17993800
H	-8.59315100	15.62381900	-8.28330300
H	-9.15702200	16.95180900	-6.26088300
H	-9.09951600	15.88027500	-4.01742300
H	-7.91652900	12.16581000	-5.80615700
H	-7.97166200	13.22705100	-8.06386000
C	-8.45599100	13.28200400	-3.45176100

O	-8.79087700	14.07822500	-2.43753600
H	-8.72624100	13.56574500	-1.58738000
O	-8.13795600	12.10353400	-3.30936100

18-ts-*si*

C	-10.39193000	16.93838600	1.99913000
C	-9.79441800	15.76138000	2.45714200
C	-9.56364100	14.68397200	1.59610700
C	-9.96786900	14.83648500	0.26277100
C	-10.56677600	15.99511400	-0.21794900
C	-10.77696100	17.05722700	0.66258500
H	-10.55890900	17.76054000	2.68921700
H	-9.50675600	15.66256400	3.49914000
H	-10.86191400	16.04750000	-1.26096300
H	-11.24257900	17.97016200	0.30282400
C	-8.93905300	13.36534700	1.99149600
H	-9.74179000	12.62492900	2.09805300
C	-8.12983600	13.30928400	3.24412600
H	-7.62905000	14.21441900	3.57110000
C	-8.39585600	12.26842600	4.17434500
O	-9.12145200	11.27917000	3.88226900
C	-7.76148500	12.29760400	5.54514300
C	-6.73837500	13.18265700	5.92079800
C	-8.22652100	11.37575500	6.49603600
C	-6.20010700	13.14822900	7.20761700
H	-6.34243000	13.89590200	5.20419800
C	-7.69515200	11.34365300	7.78417300
H	-9.01398000	10.69324900	6.19386700
C	-6.67801100	12.23046100	8.14595500
H	-5.40365500	13.83767500	7.47702000
H	-8.07470100	10.62662300	8.50833200
H	-6.25993800	12.20642000	9.14916700
O	-9.84469600	13.78531000	-0.66206400
C	-8.96050000	12.78076300	-0.47922100
O	-8.94469900	11.87803700	-1.30534700
C	-8.06594000	12.90109400	0.71281700
H	-7.43736700	13.78325800	0.49878600
C	-7.17575100	11.77224700	1.13289400
C	-6.38772500	12.19903300	2.19629800
H	-5.97803800	13.20442500	2.17523100
H	-5.84472700	11.50938800	2.83375800
P	-7.22104400	10.06961200	0.64270600
C	-6.33163800	10.05521500	-0.99297200
H	-5.31311000	10.33495200	-0.70266600
H	-6.75455600	10.91824500	-1.51340600
C	-6.27249600	8.84931100	-1.95021400
H	-6.22506600	7.90250600	-1.40915700

C	-4.99363800	8.86749500	-2.83954200
H	-5.18136100	8.10383900	-3.60110200
C	-4.72058900	10.20346300	-3.53101400
H	-4.43804800	10.97485200	-2.80756500
H	-5.60421000	10.54728600	-4.07824200
H	-3.89402000	10.10507700	-4.24154800
C	-8.84265400	9.21745200	0.59017200
C	-9.92169000	9.72183300	1.32993400
C	-8.97497900	7.98610900	-0.07341700
C	-11.13361500	9.02778700	1.35611300
H	-9.79750900	10.59755500	1.95790000
C	-10.18720100	7.29802300	-0.03597000
H	-8.14837400	7.53679500	-0.60827300
C	-11.27467100	7.82471800	0.66540000
H	-11.96037700	9.42738500	1.93663900
H	-10.27104500	6.34492500	-0.55070700
H	-12.21859200	7.28647800	0.69132700
C	-6.23622200	9.11983600	1.85171900
C	-6.75682700	8.98257000	3.15242000
C	-4.99960300	8.54276500	1.52919400
C	-6.03221100	8.28606600	4.11746800
H	-7.70122100	9.45038800	3.42214000
C	-4.28585900	7.84069800	2.50459200
H	-4.58144400	8.62428100	0.53065300
C	-4.79881900	7.71364300	3.79526900
H	-6.43176700	8.19991100	5.12384600
H	-3.33054000	7.39568900	2.24591300
H	-4.23699300	7.17141100	4.55139300
O	-3.88993200	8.45059100	-2.02540700
Si	-2.40752000	7.83161700	-2.54255600
C	-1.73389100	6.92818000	-1.02722300
C	-2.55592900	6.08399400	-0.25571000
C	-0.37362800	7.01082200	-0.67566000
C	-2.03971800	5.34604000	0.81232500
H	-3.61554600	6.00682100	-0.48500800
C	0.14744400	6.27917100	0.39393100
H	0.28977600	7.66101600	-1.24075900
C	-0.68472800	5.44174400	1.13877000
H	-2.69548000	4.69788100	1.38850600
H	1.20156900	6.36415800	0.64592000
H	-0.28104100	4.86773800	1.96902800
C	-1.26446300	9.29851400	-2.91793600
C	-1.35674100	10.42622100	-2.07595900
C	-0.30012700	9.33967700	-3.94174400
C	-0.53269800	11.53844800	-2.24864000
H	-2.09003800	10.43474300	-1.27336400
C	0.52843000	10.45061400	-4.12198200

H	-0.18482200	8.49795600	-4.61763100
C	0.41382500	11.55364500	-3.27572600
H	-0.62861800	12.39278100	-1.58312800
H	1.26199900	10.45268900	-4.92432500
H	1.05724200	12.41875600	-3.41419800
C	-2.60379400	6.57080600	-3.99490200
C	-3.70779200	5.54635800	-3.63468700
H	-3.81712200	4.82064000	-4.45368100
H	-4.69098500	5.99910200	-3.46765600
H	-3.44801600	4.97841200	-2.73427900
C	-1.28246700	5.78260200	-4.17359200
H	-1.41288700	5.01761600	-4.95251400
H	-0.98524800	5.26919400	-3.25312300
H	-0.44515100	6.41772900	-4.48380900
C	-2.95727700	7.24767600	-5.33903600
H	-2.21228800	7.99247300	-5.64010000
H	-3.93123200	7.74760500	-5.31280300
H	-3.00583800	6.49110200	-6.13558400
N	-7.45483300	8.79043600	-2.80329800
H	-7.78226100	9.65682400	-3.22361700
C	-7.92586600	7.58511000	-3.24936400
O	-7.45278700	6.52432900	-2.83294300
C	-9.06516100	7.61554300	-4.29565400
C	-8.47956900	8.11112600	-5.63940800
H	-7.65637600	7.46626000	-5.96956900
H	-9.25611700	8.08611600	-6.41354200
H	-8.10914700	9.13817300	-5.56747400
C	-9.60121900	6.18442700	-4.47004200
H	-10.02714600	5.80380500	-3.53597900
H	-10.38579500	6.17457000	-5.23585400
H	-8.80618400	5.49757700	-4.77117400
C	-10.21724900	8.54119000	-3.84849500
H	-11.02535400	8.50385100	-4.58903600
H	-10.62797000	8.22595700	-2.88351300
H	-9.90650300	9.58569000	-3.75608800
C	-7.98774100	13.03200700	-8.25613000
C	-8.98219200	13.96537000	-8.56261200
C	-9.92257400	14.33129200	-7.59589200
C	-9.87155200	13.76705500	-6.32236900
C	-8.87645400	12.82816300	-6.01281900
C	-7.93472700	12.46423500	-6.98545000
H	-7.25598100	12.74863600	-9.00745600
H	-9.02386000	14.40769100	-9.55448300
H	-10.69503500	15.05689900	-7.83490700
H	-10.59561000	14.04537500	-5.56488600
H	-7.17019500	11.73926100	-6.72741000
C	-8.78725300	12.20379000	-4.66444700

O	-9.71864900	12.63122000	-3.81361100
H	-9.54159400	12.25011500	-2.91115500
O	-7.93751300	11.36695300	-4.36544700

19-int-*re*

C	-12.94236300	11.33019200	3.96284600
C	-11.56364500	11.15023200	4.10765900
C	-10.68503500	11.46852500	3.06989200
C	-11.23766500	11.98080000	1.88562800
C	-12.60462300	12.16856600	1.71891100
C	-13.46145100	11.83672900	2.77035100
H	-13.60833800	11.07469300	4.78198800
H	-11.16398500	10.75899400	5.03920400
H	-12.97719400	12.57111000	0.78256800
H	-14.53221700	11.97664000	2.65416400
C	-9.18642000	11.32879200	3.07603900
H	-8.74334600	12.31500200	3.27236300
C	-8.49122800	10.34131300	4.02499400
H	-9.17095800	9.52547100	4.28673200
C	-7.95887300	11.00929600	5.28471900
O	-7.67830300	12.20452600	5.29194900
C	-7.71896000	10.17707500	6.50977700
C	-7.88191200	8.78264300	6.53676600
C	-7.30126700	10.83553400	7.67896400
C	-7.63380000	8.06495300	7.70729500
H	-8.18698700	8.24616700	5.64404900
C	-7.06237200	10.12042400	8.84816100
H	-7.17172900	11.91207000	7.64094700
C	-7.22853500	8.73173900	8.86499000
H	-7.75673100	6.98542400	7.71429700
H	-6.74467400	10.64144200	9.74733500
H	-7.04073300	8.17166700	9.77727500
O	-10.41979400	12.39327000	0.82193300
C	-9.13577600	11.95698800	0.70465500
O	-8.43981500	12.45580800	-0.16340300
C	-8.72735500	10.88312800	1.66037300
H	-9.36379100	10.01579100	1.38141700
C	-7.29385400	10.47585600	1.84707000
C	-7.26815200	9.75125800	3.18391400
P	-5.92959800	11.02885300	1.00874100
C	-5.86484100	10.71826200	-0.83968000
H	-4.79850500	10.68211000	-1.08602400
H	-6.29910300	11.58212600	-1.34802600
C	-6.52069800	9.41584700	-1.32903100
H	-6.49579800	8.65732800	-0.54404300
C	-5.78775900	8.80138400	-2.55944400
H	-6.38570800	7.93086200	-2.84809300

C	-5.67348300	9.75197300	-3.75046500
H	-4.98254200	10.57143200	-3.52606500
H	-6.64338500	10.18594900	-4.00959000
H	-5.28538900	9.21870700	-4.62417000
C	-4.47390400	10.16107300	1.71272800
C	-3.36790600	10.86486400	2.21309700
C	-4.45566100	8.75597400	1.74369700
C	-2.27019600	10.17805000	2.73594500
H	-3.36253700	11.94911100	2.20379100
C	-3.35581700	8.07346200	2.26381600
H	-5.30145400	8.18870500	1.37020700
C	-2.26112100	8.78348600	2.76120700
H	-1.42369400	10.73761600	3.12503100
H	-3.35751300	6.98783000	2.26929000
H	-1.40384800	8.25082000	3.16406500
C	-5.47831600	12.83390900	1.11231900
C	-5.85478400	13.52598100	2.27310100
C	-4.78415000	13.52537500	0.10664200
C	-5.54384300	14.87833700	2.42624300
H	-6.39154400	13.00526100	3.06284500
C	-4.47967800	14.87961600	0.25764000
H	-4.47365000	13.01561000	-0.79997700
C	-4.85991000	15.55886300	1.41704900
H	-5.84250000	15.39883400	3.33230300
H	-3.94667000	15.40333600	-0.53178300
H	-4.62440900	16.61370900	1.53198600
O	-4.47842600	8.39214400	-2.16223400
Si	-3.65062100	6.93465400	-2.14895100
C	-4.35718600	5.78036800	-0.81936000
C	-5.74832900	5.67895600	-0.61444800
C	-3.52578700	4.98279800	-0.00821200
C	-6.28056900	4.82158700	0.35235700
H	-6.44859800	6.28257900	-1.18391000
C	-4.05376000	4.11720700	0.95289400
H	-2.44599400	5.04563000	-0.11089400
C	-5.43590900	4.03401600	1.13586000
H	-7.35806500	4.78029700	0.48799100
H	-3.38504800	3.51252100	1.56122500
H	-5.84903300	3.36418200	1.88614400
C	-1.88779600	7.47203100	-1.71011500
C	-1.61311200	8.83107200	-1.46738100
C	-0.80661400	6.57248600	-1.61803700
C	-0.32781600	9.27095800	-1.14540800
H	-2.42212100	9.55118000	-1.53157200
C	0.48160100	7.00531800	-1.29651300
H	-0.96055000	5.51213700	-1.79810100
C	0.72492000	8.35891000	-1.05871400

H	-0.14922500	10.32758200	-0.96130700
H	1.29376300	6.28511700	-1.23383800
H	1.72677300	8.69933800	-0.80895100
C	-3.69558600	6.05493500	-3.86912400
C	-5.15090600	5.80617900	-4.32497700
H	-5.15544100	5.22718300	-5.26023800
H	-5.68962700	6.73841400	-4.52534500
H	-5.72444000	5.23486200	-3.58551800
C	-2.99001600	4.68136600	-3.78310700
H	-3.07062200	4.16226600	-4.74941800
H	-3.43974700	4.03223000	-3.02298300
H	-1.92122300	4.77736400	-3.56228200
C	-2.97186700	6.91759800	-4.92546300
H	-1.92026400	7.08041800	-4.66386400
H	-3.43920400	7.90200900	-5.04209800
H	-2.99864800	6.42064800	-5.90644700
N	-7.93525300	9.61901900	-1.65350000
H	-8.19522600	10.50494000	-2.07694500
C	-8.80743300	8.57150000	-1.62845300
O	-8.46575600	7.45258300	-1.23208300
C	-10.25778000	8.78491100	-2.12898500
C	-10.60404800	10.22753800	-2.53129100
H	-9.97561200	10.59346300	-3.34932700
H	-11.64449500	10.26664900	-2.87530900
H	-10.51403700	10.92698400	-1.69296100
C	-10.42900200	7.85866900	-3.35527400
H	-10.17853600	6.82716200	-3.09354800
H	-11.46519200	7.89188200	-3.71262900
H	-9.77817100	8.17170300	-4.18101100
C	-11.20435300	8.31652900	-1.00332100
H	-12.24237600	8.31883800	-1.35672700
H	-10.94212200	7.30603600	-0.68006200
H	-11.14691000	8.98108500	-0.13212000
H	-7.44649100	8.66765500	3.08851300
H	-6.32309500	9.85494200	3.72583000
C	-8.69733900	15.59094400	-7.05499200
C	-9.14336200	16.18785300	-5.87288200
C	-9.07855800	15.48846000	-4.66870000
C	-8.56578800	14.18345000	-4.64438500
C	-8.11924300	13.58845800	-5.83232900
C	-8.18488000	14.29057700	-7.03351100
H	-8.74867000	16.13861700	-7.99239000
H	-9.54138100	17.19869600	-5.88977600
H	-9.42141100	15.94288600	-3.74593000
H	-7.72500000	12.57848200	-5.79338900
H	-7.83766800	13.82634200	-7.95243600
C	-8.48051200	13.40042800	-3.37939500

O	-8.93013300	14.06039600	-2.31299400
H	-8.82213900	13.48961600	-1.50591100
O	-8.04026500	12.25451600	-3.33059700

19-int-*si*

C	-7.13520500	17.03404900	1.95796200
C	-7.47550000	15.76560500	2.43676200
C	-7.93607300	14.77405100	1.56888200
C	-8.05079400	15.09961200	0.20888000
C	-7.72489700	16.35581200	-0.28837700
C	-7.26085000	17.32864100	0.59937700
H	-6.77646800	17.79160200	2.64873800
H	-7.38693000	15.54627700	3.49638000
H	-7.83768800	16.55510200	-1.34913000
H	-7.00029300	18.31475800	0.22598300
C	-8.34086600	13.36950000	1.92635200
H	-9.44021800	13.32770200	1.94537600
C	-7.86881300	12.67523500	3.20701200
H	-6.78012900	12.76927100	3.29633800
C	-8.55927600	13.17371700	4.47065700
O	-9.63458000	13.75599900	4.39820200
C	-7.92980100	12.91368600	5.80808700
C	-6.73109200	12.20057300	5.96895100
C	-8.58467200	13.41095300	6.94770100
C	-6.20032700	11.99045800	7.24214100
H	-6.21033700	11.79749200	5.10670300
C	-8.05236500	13.20540700	8.21653900
H	-9.51236800	13.95594500	6.80711000
C	-6.85743100	12.49391300	8.36637700
H	-5.27403300	11.43396600	7.35511700
H	-8.56605700	13.59682700	9.09047400
H	-6.44091400	12.33194200	9.35710800
O	-8.55740900	14.17545500	-0.71619600
C	-8.56995100	12.83943800	-0.44960300
O	-9.11438300	12.10390200	-1.25694400
C	-7.83881500	12.42455100	0.80390000
H	-6.79038800	12.72148800	0.58304300
C	-7.96970200	11.04685300	1.40190600
C	-8.22422400	11.18005300	2.90238200
H	-7.61899600	10.50171100	3.51708200
H	-9.27347100	11.01083700	3.20103700
P	-7.64131600	9.56239100	0.64755700
C	-6.69981700	9.87415300	-0.91586400
H	-5.72875900	10.26103500	-0.59046000
H	-7.22581100	10.69520500	-1.40970200
C	-6.47852300	8.72690500	-1.91646700
H	-6.30227200	7.77990600	-1.40196400

C	-5.22135100	8.95560300	-2.80536400
H	-5.28627300	8.18468300	-3.58013100
C	-5.16244100	10.32986300	-3.47286500
H	-4.99938600	11.12251200	-2.73571700
H	-6.09201900	10.54102500	-4.01093300
H	-4.33565000	10.37652500	-4.18871300
C	-9.11492500	8.49222200	0.28699400
C	-10.37863100	9.09472800	0.30518300
C	-9.01549800	7.11062000	0.05591200
C	-11.52975900	8.33162000	0.09704100
H	-10.44540100	10.16360000	0.48836000
C	-10.16549000	6.34900900	-0.15260700
H	-8.04584300	6.62264000	0.03354500
C	-11.42444300	6.95771200	-0.12793400
H	-12.50611000	8.80877300	0.11624900
H	-10.07750200	5.28093700	-0.33218100
H	-12.31958500	6.36040400	-0.28152700
C	-6.63465800	8.46861900	1.73297400
C	-7.25767400	7.83013600	2.82007400
C	-5.24012300	8.37315100	1.60162900
C	-6.50321200	7.12826800	3.76132900
H	-8.33772600	7.87275100	2.92732400
C	-4.48952200	7.66050600	2.53839400
H	-4.72852400	8.83209600	0.76171900
C	-5.11718700	7.04262900	3.62224200
H	-7.00089900	6.64509800	4.59802100
H	-3.41466300	7.58068700	2.40971100
H	-4.52864400	6.49191000	4.35138500
O	-4.06498600	8.70503400	-1.99431400
Si	-2.49509100	8.36913400	-2.50098700
C	-1.69446800	7.53503000	-1.00719200
C	-2.37712100	6.54717800	-0.27181800
C	-0.36637500	7.82028400	-0.63915900
C	-1.75532200	5.86417900	0.77630300
H	-3.41042400	6.31370600	-0.51424000
C	0.25933100	7.14402500	0.41041500
H	0.18774700	8.58631000	-1.17625300
C	-0.43372200	6.16048500	1.11854300
H	-2.30356500	5.10246400	1.32525600
H	1.28582600	7.38528500	0.67525900
H	0.05199500	5.62865700	1.93295600
C	-1.61084700	10.02243800	-2.79979500
C	-1.92482600	11.09505800	-1.93988400
C	-0.63249200	10.25545500	-3.78362200
C	-1.29869300	12.33608600	-2.05741300
H	-2.67876200	10.95667200	-1.16878100
C	-0.00092200	11.49590300	-3.90872600

H	-0.35141700	9.46416700	-4.47183300
C	-0.33302600	12.54034100	-3.04575700
H	-1.56432900	13.14386500	-1.37988000
H	0.74881700	11.64465500	-4.68185700
H	0.15617800	13.50629200	-3.14184900
C	-2.45058700	7.15565200	-4.00248000
C	-3.37666500	5.94858900	-3.71132500
H	-3.33774800	5.24437300	-4.55513400
H	-4.42663800	6.22369700	-3.56538800
H	-3.05159900	5.40102900	-2.81941200
C	-1.01544600	6.60078800	-4.18040700
H	-1.00445600	5.86677600	-4.99903800
H	-0.65857200	6.09372600	-3.27762200
H	-0.28502900	7.37703400	-4.43463000
C	-2.88854000	7.82284700	-5.32621900
H	-2.27517100	8.69507400	-5.57849200
H	-3.93335300	8.15015500	-5.30260000
H	-2.79605300	7.10499300	-6.15423900
N	-7.64988900	8.52600900	-2.76083800
H	-8.11010300	9.34566500	-3.14504400
C	-7.94311600	7.27985400	-3.24601300
O	-7.29246100	6.29123700	-2.90142300
C	-9.09477600	7.17815100	-4.27441900
C	-8.53456700	7.64054800	-5.64115200
H	-7.66933800	7.03526900	-5.93599500
H	-9.30350900	7.53282200	-6.41586800
H	-8.23214900	8.69239800	-5.60926500
C	-9.52927000	5.70542700	-4.36696000
H	-9.94594600	5.35895600	-3.41506800
H	-10.29868800	5.59266600	-5.14002600
H	-8.68264900	5.05957500	-4.61134700
C	-10.30792700	8.04933300	-3.88836100
H	-11.11719500	7.88493600	-4.61057500
H	-10.68496400	7.79059400	-2.89385900
H	-10.07742500	9.11868400	-3.90149200
C	-8.81602700	12.14650000	-8.42899900
C	-9.60009800	13.25506600	-8.76043900
C	-10.23072200	13.99235900	-7.75481500
C	-10.07912800	13.62489300	-6.41874800
C	-9.29608200	12.51064200	-6.08296300
C	-8.66494500	11.77471800	-7.09511300
H	-8.32438400	11.57336900	-9.21019100
H	-9.71918700	13.54416400	-9.80138000
H	-10.84036100	14.85412700	-8.01219500
H	-10.56358600	14.19109200	-5.63128500
H	-8.05939500	10.91787800	-6.82025000
C	-9.11604400	12.08459800	-4.66547500

O	-9.71215300	12.89251100	-3.79244700
H	-9.52728400	12.58290700	-2.86356600
O	-8.47872800	11.08548200	-4.33960600

I. References

(1) (a) Jin, Z.; Yang. R.; Du, Y.; Tiwari, B.; Ganguly, R.; Chi, Y. R. *Org. Lett.* **2012**, *14*, 3226. (b) Christensen, J.; Albrecht, Ł.; Jorgensen, K. H. *Chem. Asian J.* **2013**, *8*, 648.

(2) O. Kwon, F. Tamanoi, H. Fiji, M. Watanabe, WO 2010014054, **2010**.

(3) Frisch, M. J.; Trucks, G. W.; Schlegel, H. B.; Scuseria, G. E.; Robb, M. A.; Cheeseman, J. R.; Scalmani, G.; Barone, V.; Mennucci, B.; Petersson, G. A.; Nakatsuji, H.; Caricato, M.; Li, X.; Hratchian, H. P.; Izmaylov, A. F.; Bloino, J.; Zheng, G.; Sonnenberg, J. L.; Hada, M.; Ehara, M.; Toyota, K.; Fukuda, R.; Hasegawa, J.; Ishida, M.; Nakajima, T.; Honda, Y.; Kitao, O.; Nakai, H.; Vreven, T.; Montgomery, J. A. Jr.; Peralta, J. E.; Ogliaro, F.; Bearpark, M.; Heyd, J. J.; Brothers, E.; Kudin, K. N.; Staroverov, V. N.; Keith, T.; Kobayashi, R.; Normand, J.; Raghavachari, K.; Rendell, A.; Burant, J. C.; Iyengar, S. S.; Tomasi, J.; Cossi, M.; Rega, N.; Millam, J. M.; Klene, M.; Knox, J. E.; Cross, J. B.; Bakken, V.; Adamo, C.; Jaramillo, J.; Gomperts, R.; Stratmann, R. E.; Yazyev, O.; Austin, A. J.; Cammi, R.; Pomelli, C.; Ochterski, J. W.; Martin, R. L.; Morokuma, K.; Zakrzewski, V. G.; Voth, G. A.; Salvador, P.; Dannenberg, J. J.; Dapprich, S.; Daniels, A. D.; Farkas, O.; Foresman, J. B.; Ortiz, J. V.; Cioslowski, J.; and Fox, D. J. Gaussian 09, revision D.01; Gaussian, Inc.: Wallingford, CT, **2013**.

(4) (a) Becke, A. D. *J. Chem. Phys*. **1993**, *98*, 5648. (b) Lee, C.; Yang, W.; Parr, R. G. *Phys. Rev. B: Condens. Matter Mater. Phys*. **1988**, *37*, 785. (c) Stephens, P. J.; Devlin, F. J. Chabalowski, C. F.; Frisch, M. J. *J. Phys. Chem*. **1994**, *98*, 11623.

(5) (a) Hehre, W. J.; Ditchfield, R.; Pople, J. A. *J. Chem. Phys*. **1972**, *56*, 2257. (b) Dill, J. D.; Pople, J. A. *J. Chem. Phys*. **1975**, *62*, 2921. (c) Francl, M. M.; Pietro, W. J.; Hehre, W. J.; Binkley, J. S.; Gordon, M. S.; DeFrees, D. J.; Pople, J. A. *J. Chem. Phys*. **1982**, *77*, 3654.

(6) (a) Barone, V.; Cossi, M. *J. Phys. Chem. A* **1998**, *102*, 1995. (b) Cossi, M.; Rega, N.; Scalmani, G.; Barone, V. *J. Comp. Chem*. **2003**, *24*, 669.

(7) Peverati, R.; Truhlar, D. G. *J. Phys. Chem. Lett*. **2011**, *2*, 2810.

(8) (a) Krishnan, R.; Binkley, J. S.; Seeger, R.; Pople, J. A. *J. Chem. Phys*. **1980**, *72*, 650. (b) McLean, A. D.; Chandler, G. S. *J. Chem. Phys*. **1980**, *72*, 5639.

J NMR spectra of the products

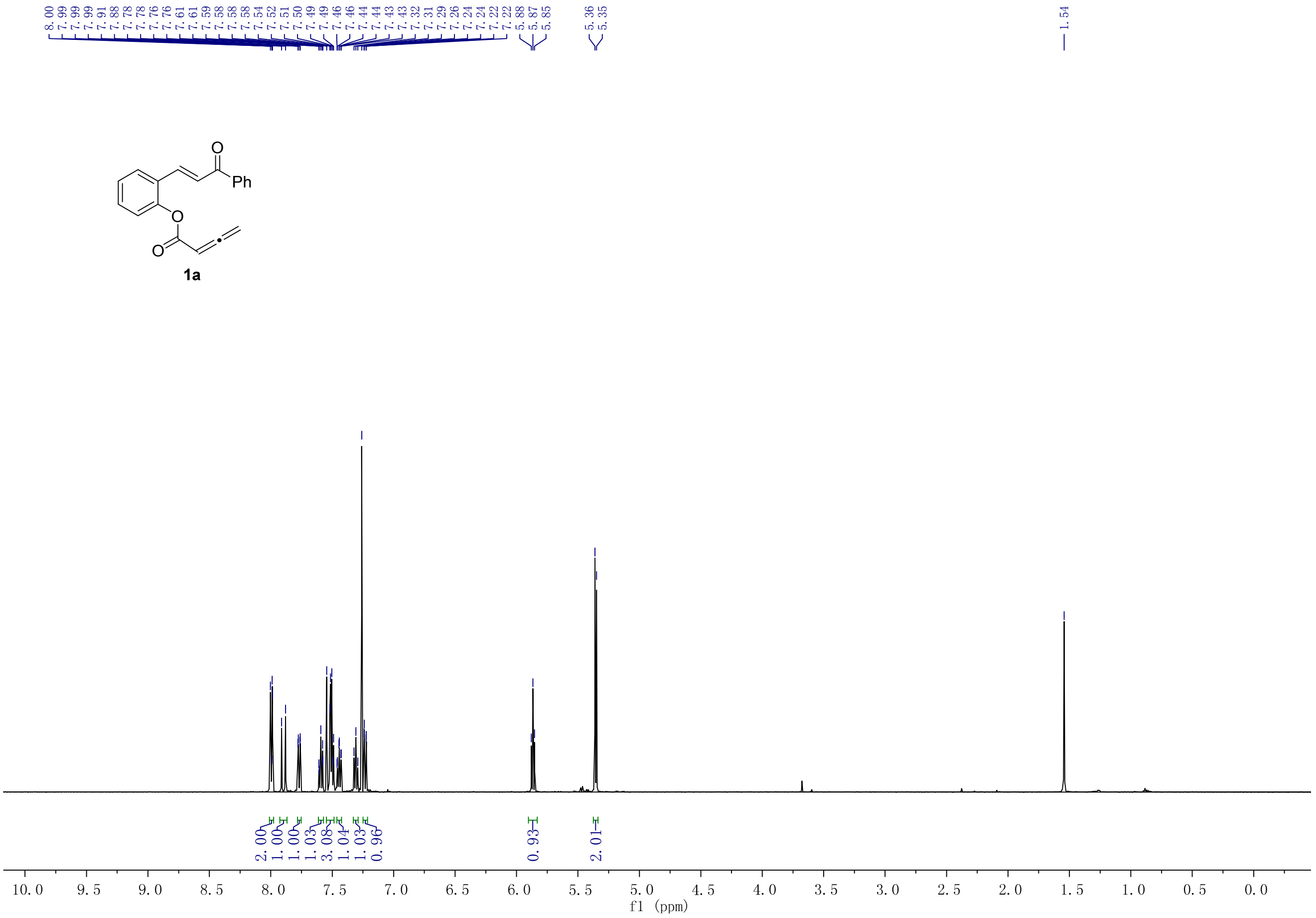

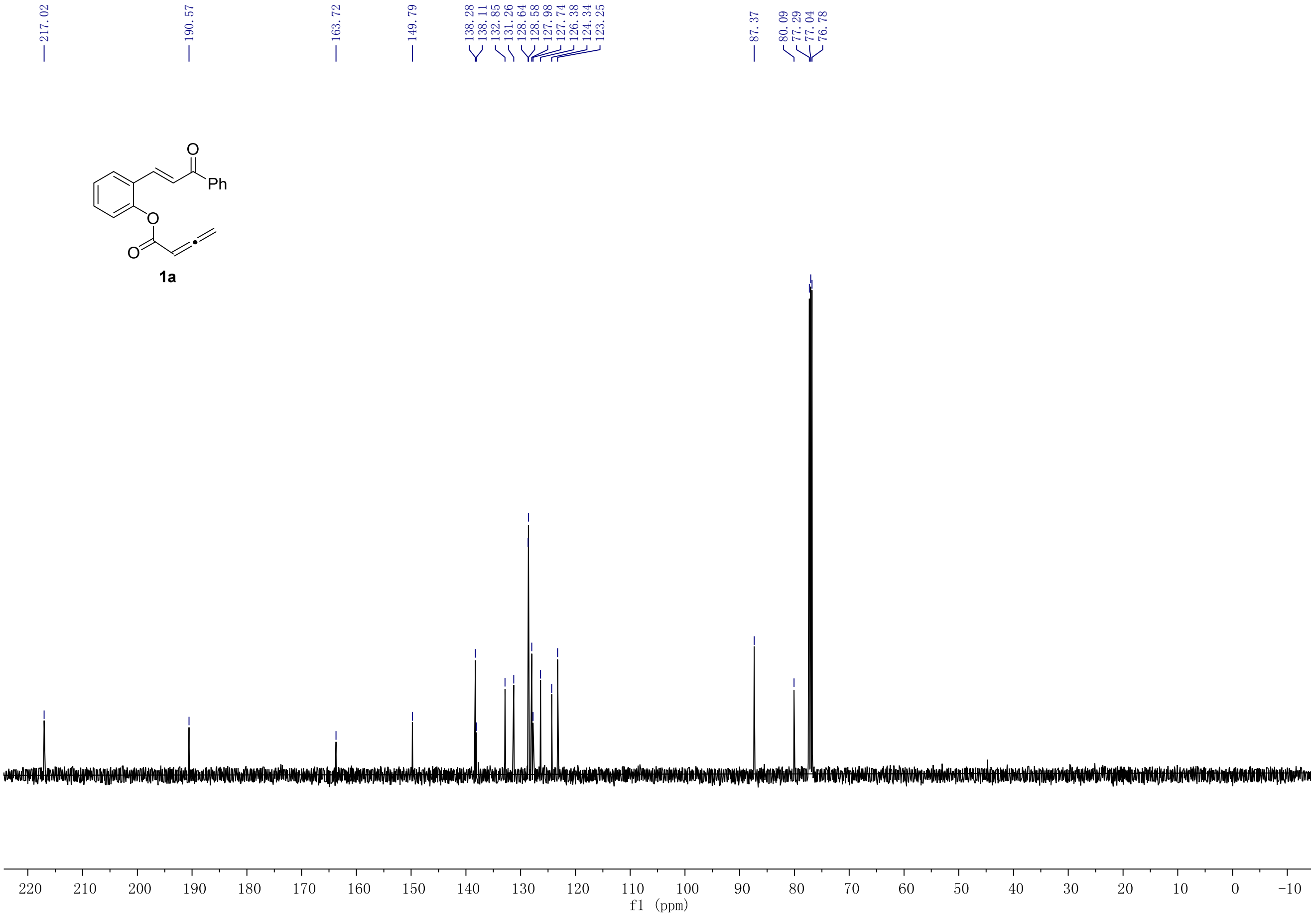

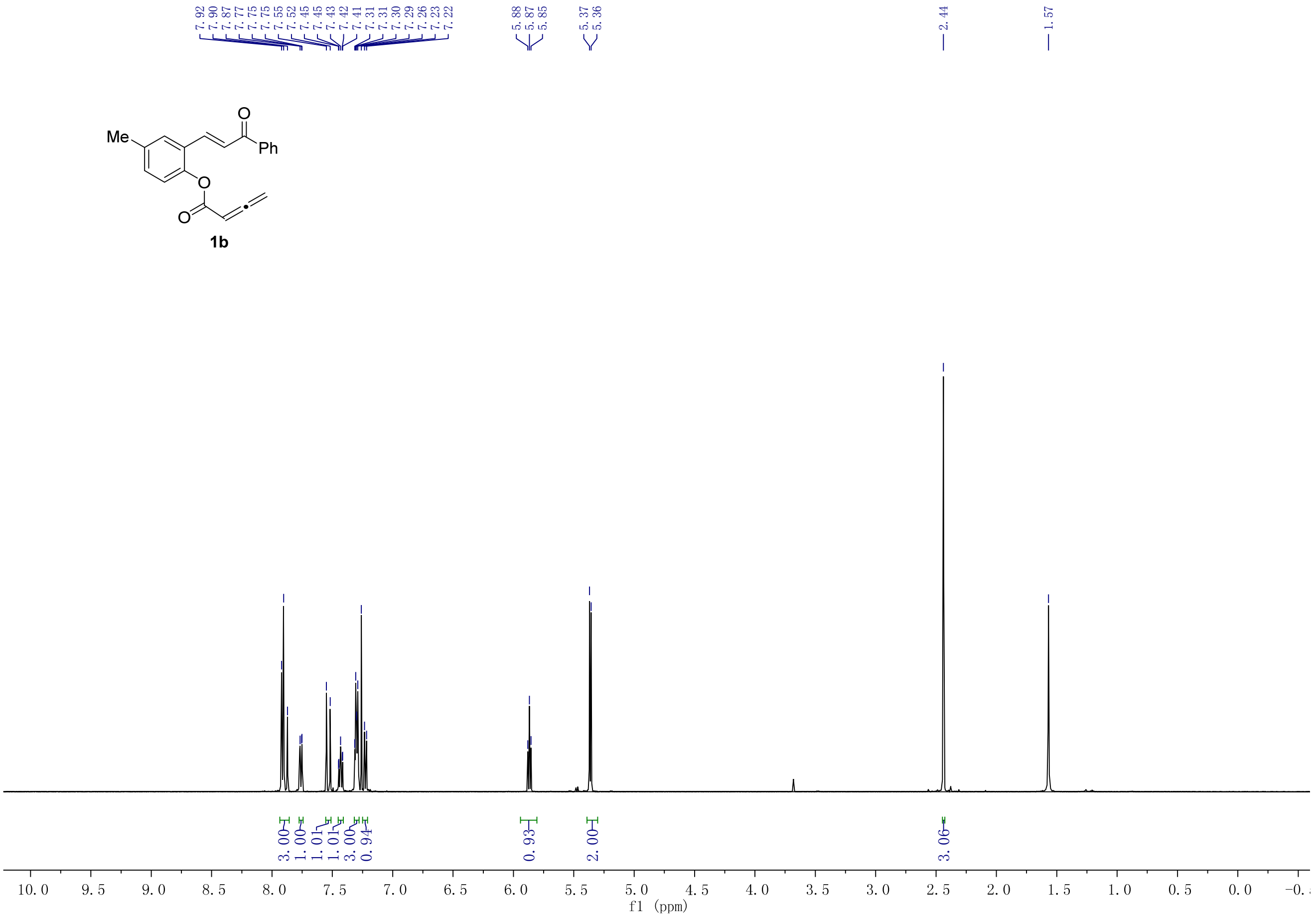
7.92
7.90
7.87
7.77
7.75
7.55
7.52
7.45
7.45
7.43
7.42
7.41
7.31
7.31
7.30
7.29
7.26
7.23
7.22
5.88
5.87
5.85
5.37
5.36
2.44
1.57
Me
O
Ph
O
O
1b
3.00
1.00
1.01
1.01
3.06
0.94
0.93
2.00
3.06
f1 (ppm)

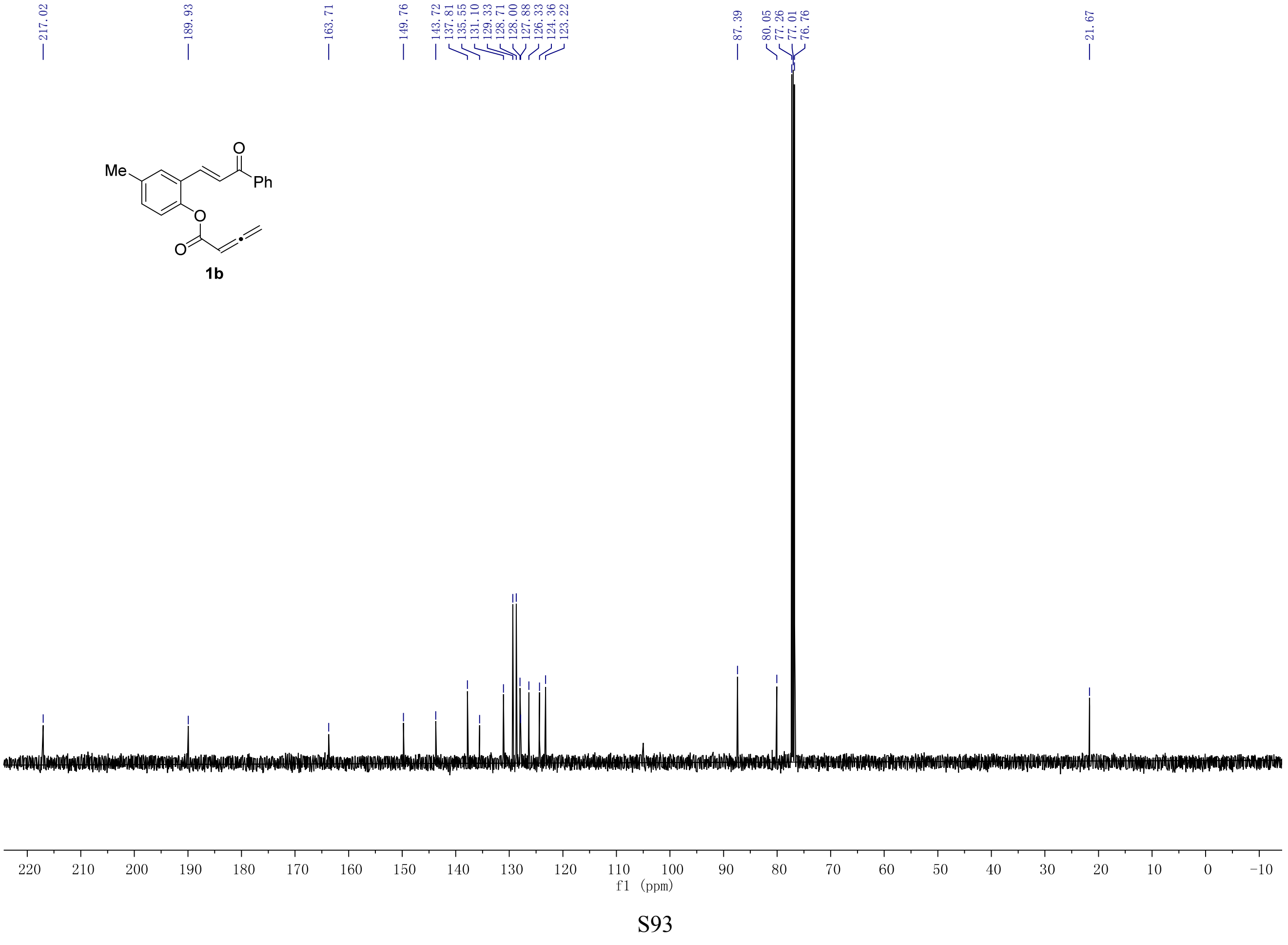
217.02
189.93
163.71
149.76
143.72
137.81
135.55
131.10
129.33
128.71
128.00
127.88
126.33
124.36
123.22
87.39
80.05
77.26
77.01
76.76
21.67
Me
O
Ph
O
O
1b
f1 (ppm)

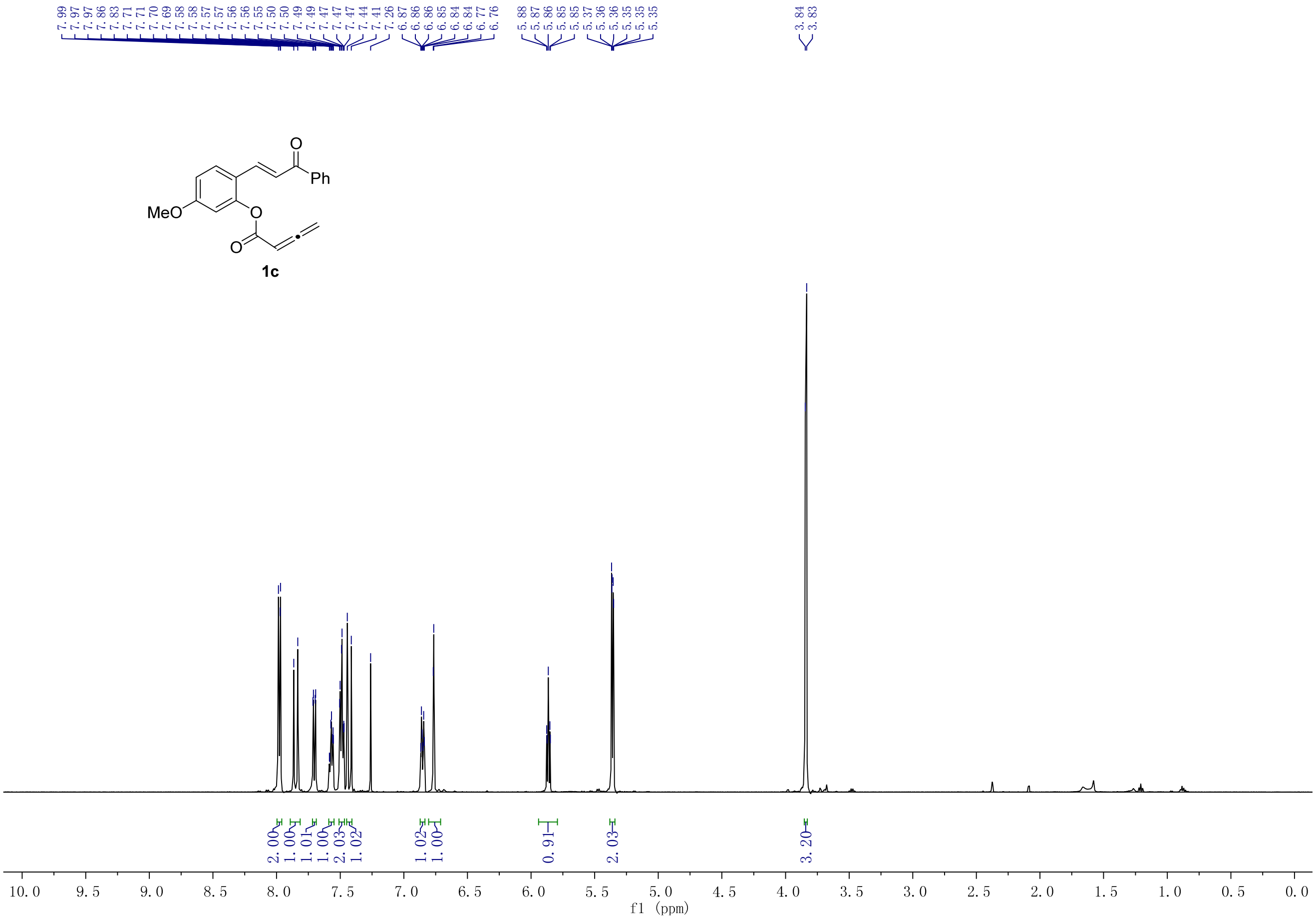

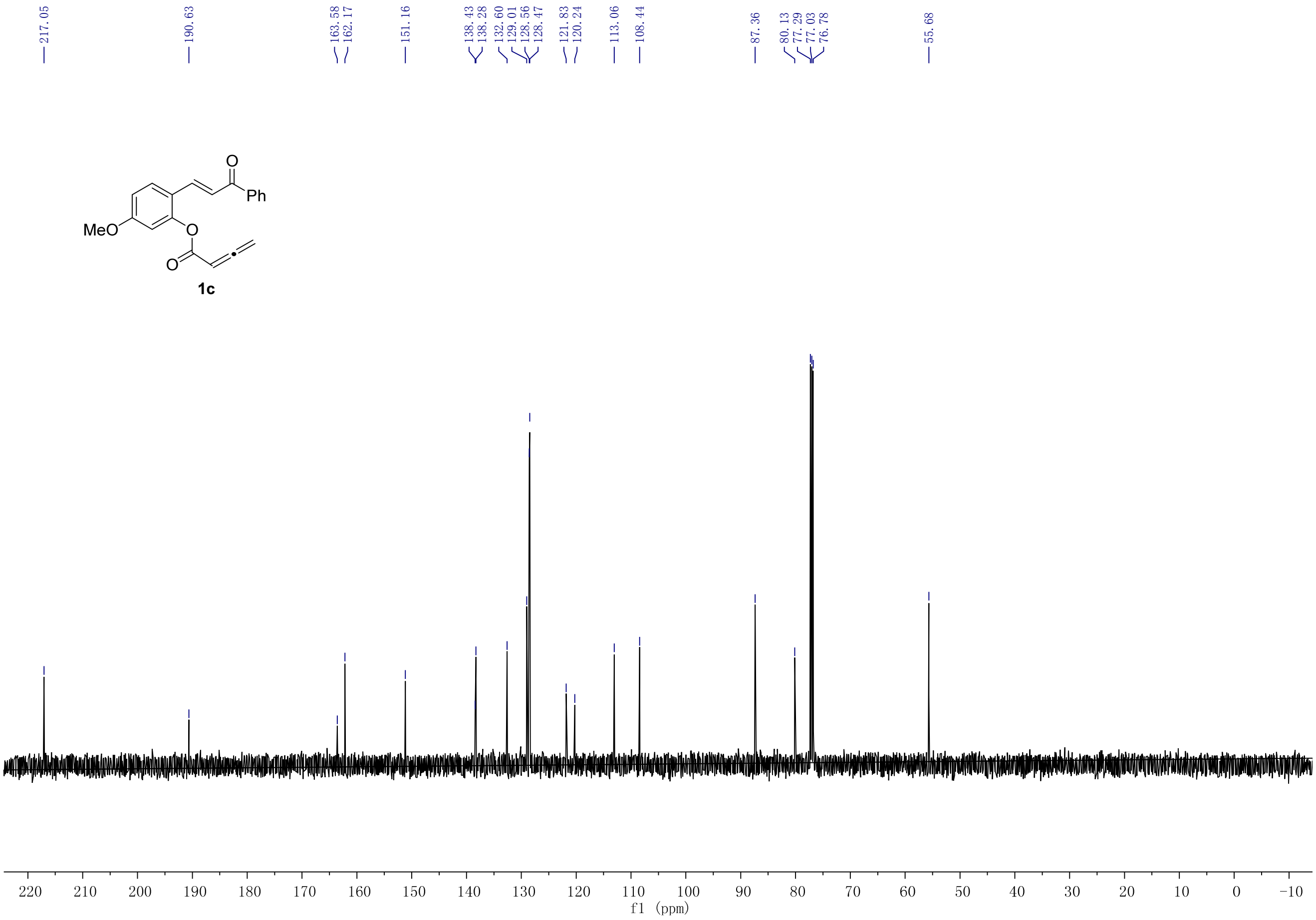

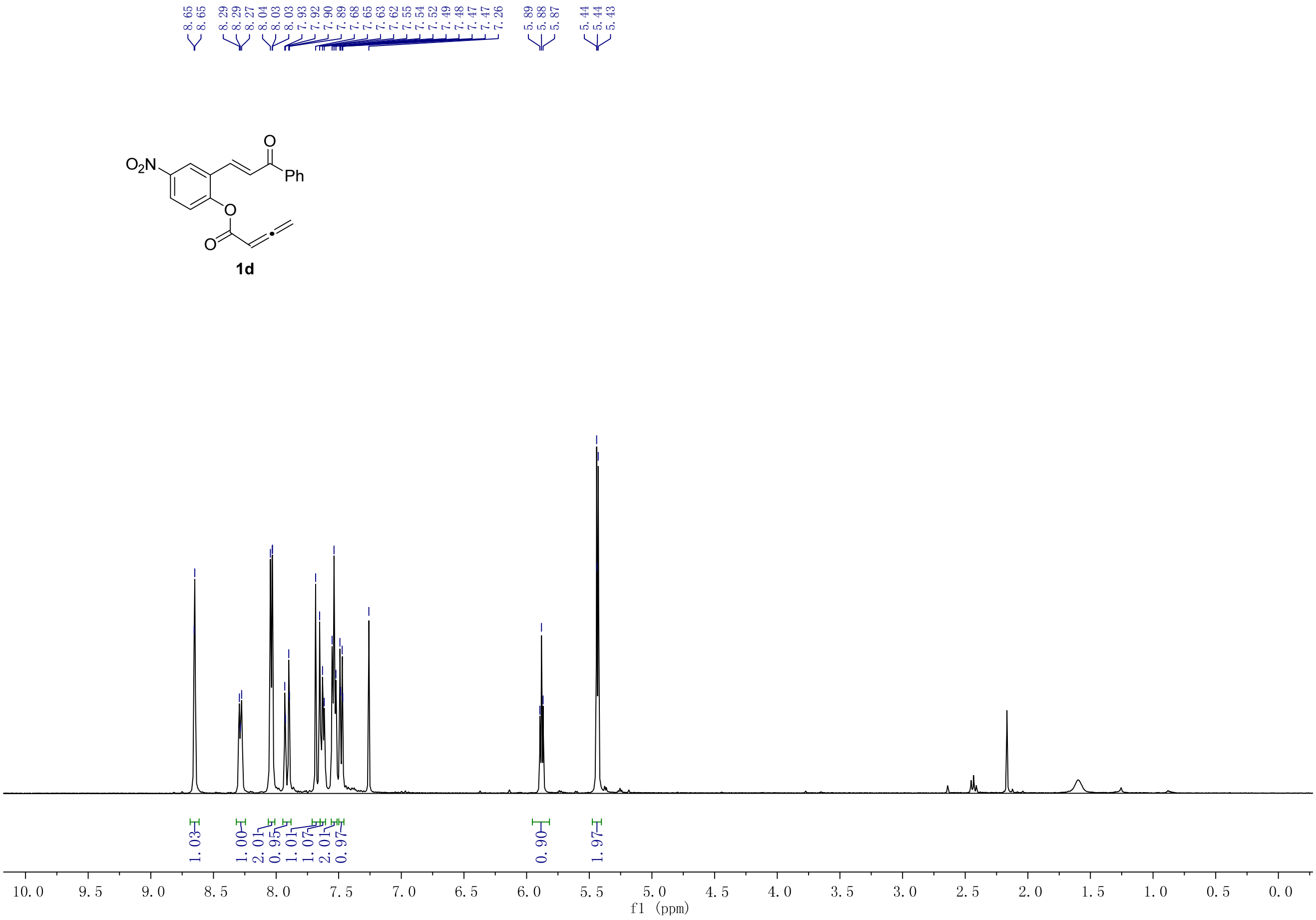

8.65
8.65
8.29
8.29
8.27
8.04
8.03
8.03
7.93
7.92
7.90
7.89
7.68
7.65
7.63
7.62
7.55
7.54
7.52
7.49
7.48
7.47
7.47
7.26
5.89
5.88
5.87
5.44
5.44
5.43
O_2N
O
Ph
O
O
1d
1.03
1.00
2.01
0.95
1.01
1.07
2.01
0.97
0.90
1.97
10.0
9.5
9.0
8.5
8.0
7.5
7.0
6.5
6.0
5.5
5.0
4.5
4.0
3.5
3.0
2.5
2.0
1.5
1.0
0.5
0.0
f1 (ppm)

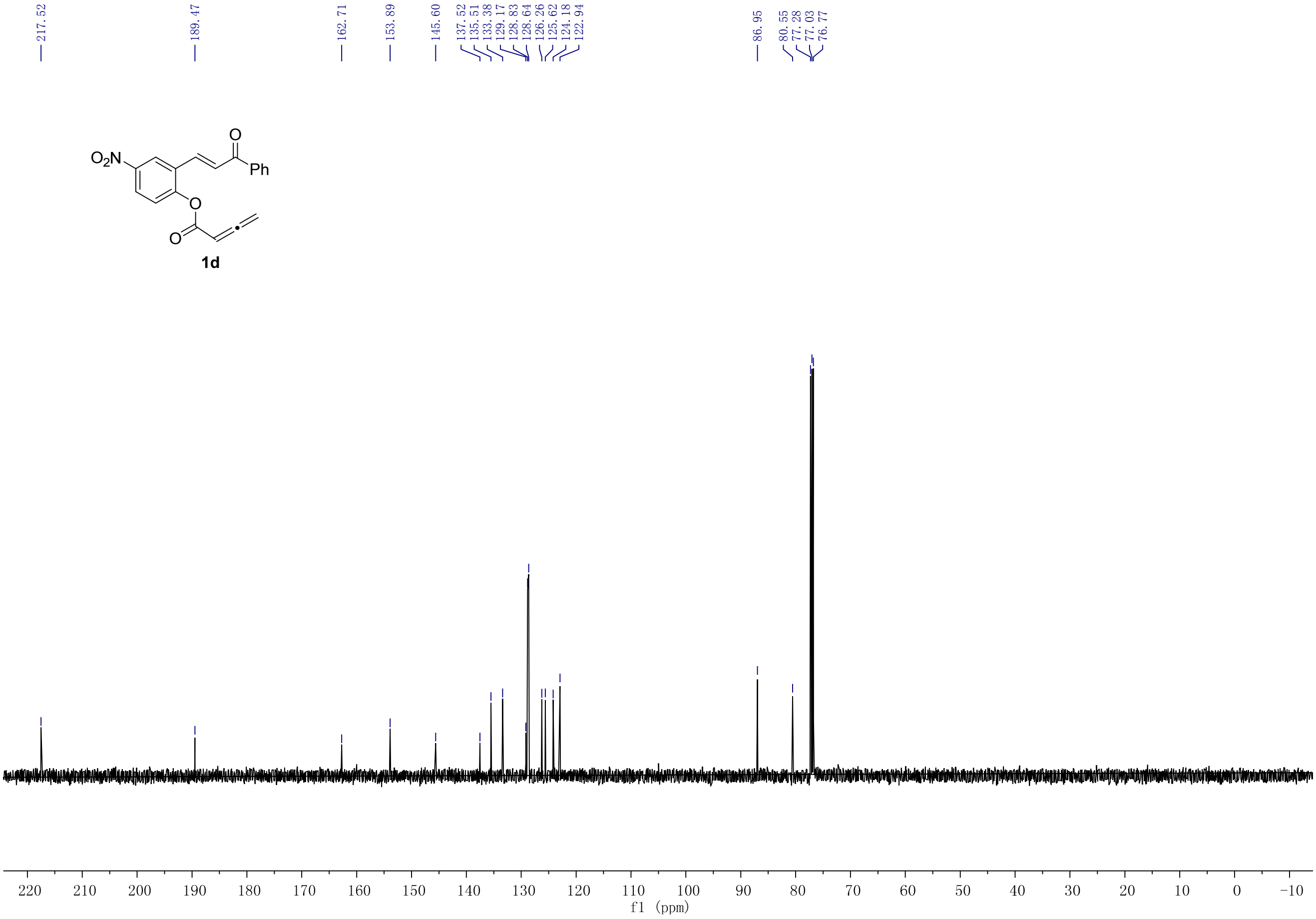

217.52
189.47
162.71
153.89
145.60
137.52
135.51
133.38
129.17
128.83
128.64
126.26
125.62
124.18
122.94
86.95
80.55
77.28
77.03
76.77
O_2N
O
Ph
O
O
1d
220
210
200
190
180
170
160
150
140
130
120
110
100
90
80
70
60
50
40
30
20
10
0
−10
f1 (ppm)

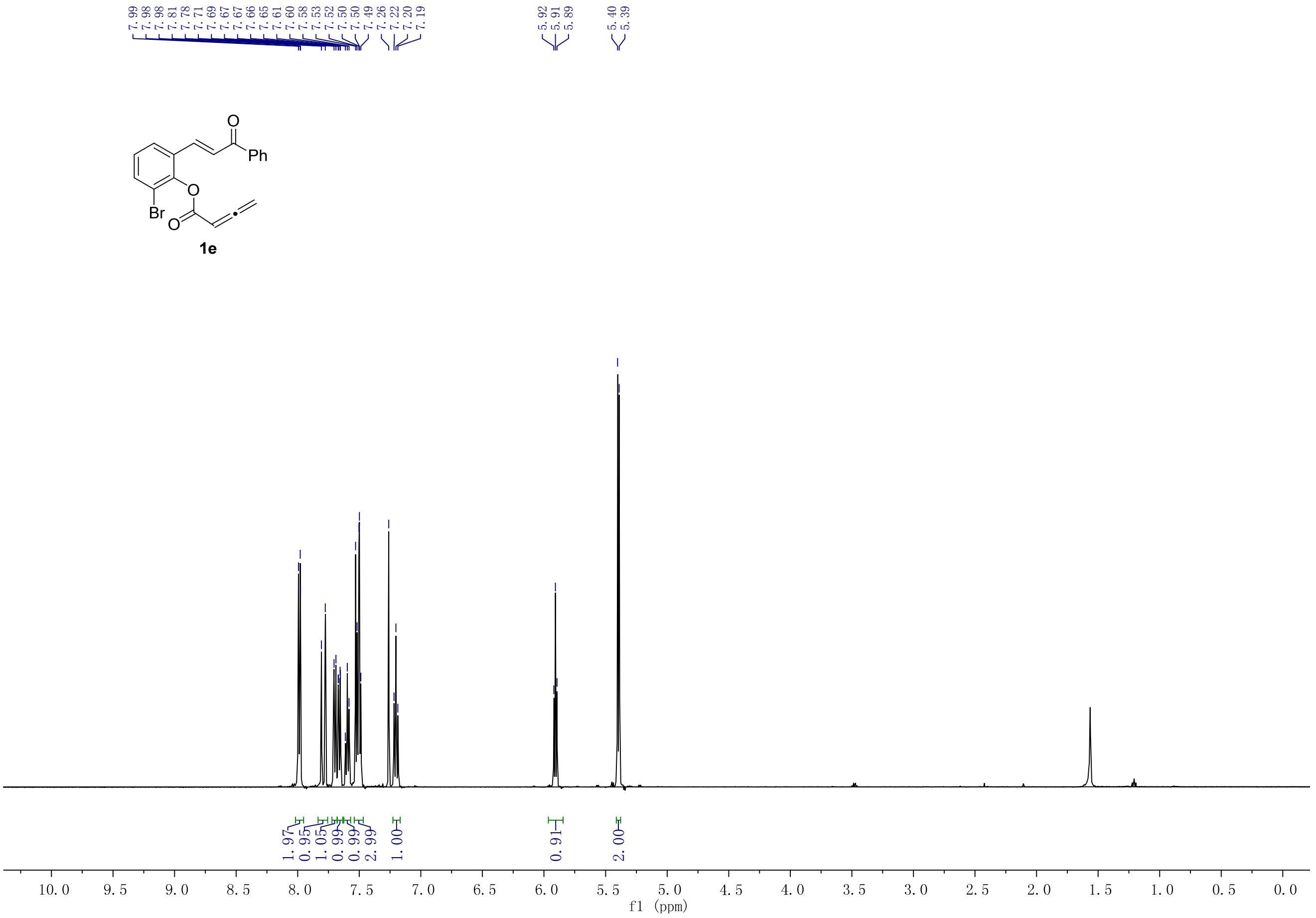
7.99 7.98 7.98 7.81 7.78 7.71 7.69 7.67 7.66 7.65 7.61 7.60 7.58 7.53 7.52 7.50 7.50 7.49 7.26 7.22 7.20 7.19
5.92 5.91 5.89
5.40 5.39
O
Ph
O
Br
O
1e
1.97 0.95 1.05 0.99 0.99 2.99 1.00
0.91
2.00
10.0 9.5 9.0 8.5 8.0 7.5 7.0 6.5 6.0 5.5 5.0 4.5 4.0 3.5 3.0 2.5 2.0 1.5 1.0 0.5 0.0
f1 (ppm)

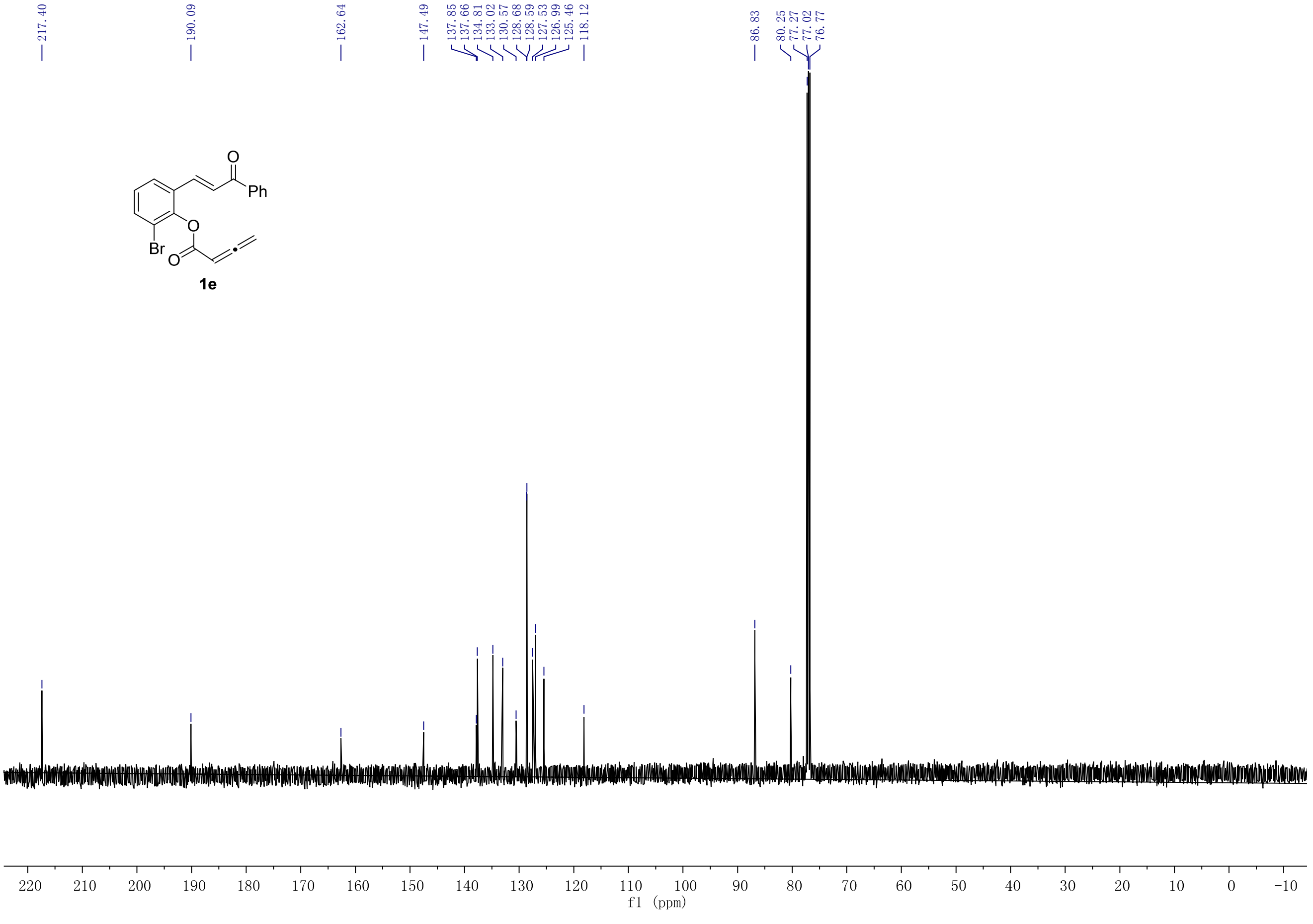
217.40
190.09
162.64
147.49
137.85 137.66 134.81 133.02 130.57 128.68 128.59 127.53 126.99 125.46 118.12
86.83
80.25 77.27 77.02 76.77
O
Ph
O
Br
O
1e
220 210 200 190 180 170 160 150 140 130 120 110 100 90 80 70 60 50 40 30 20 10 0 -10
f1 (ppm)

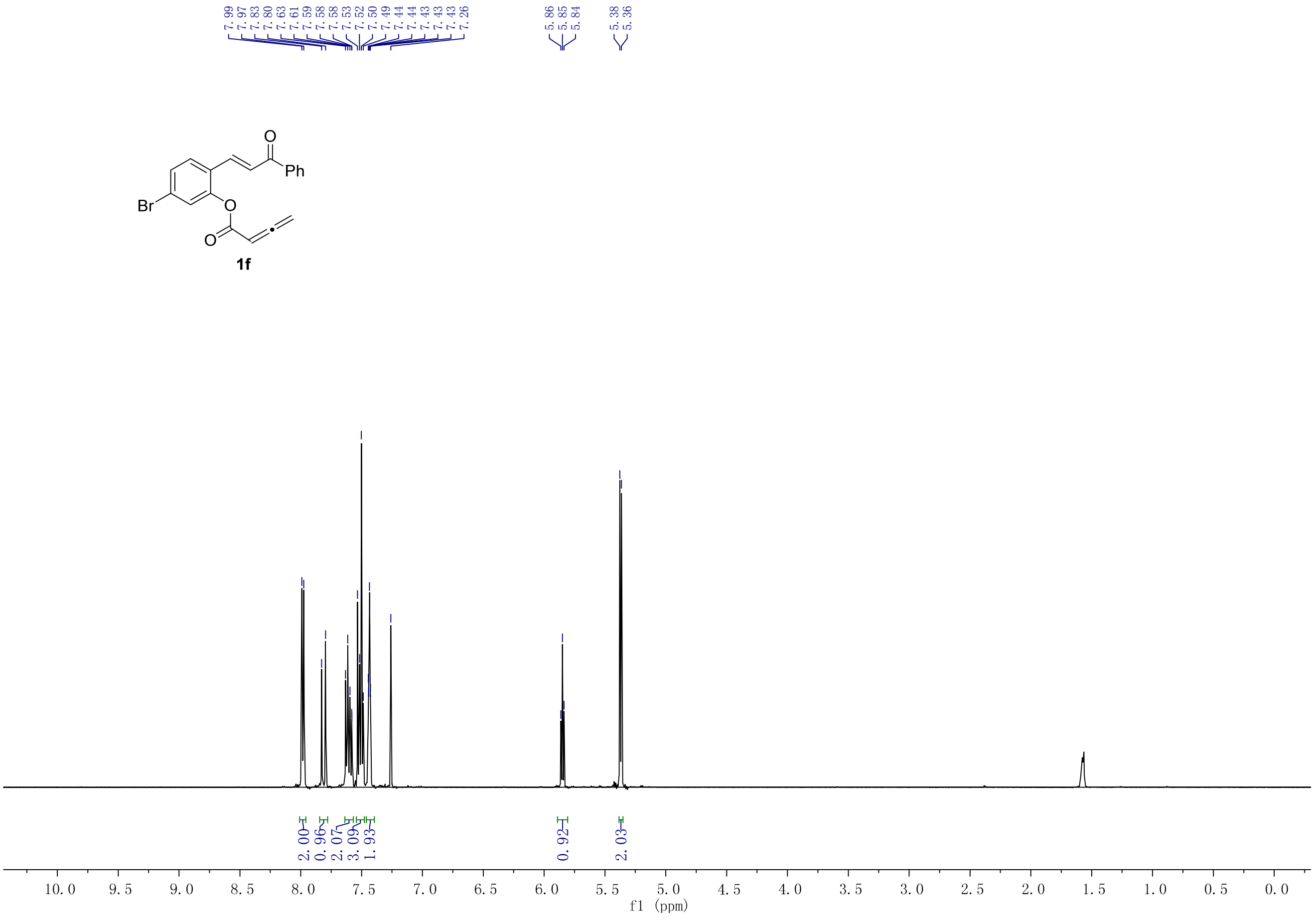

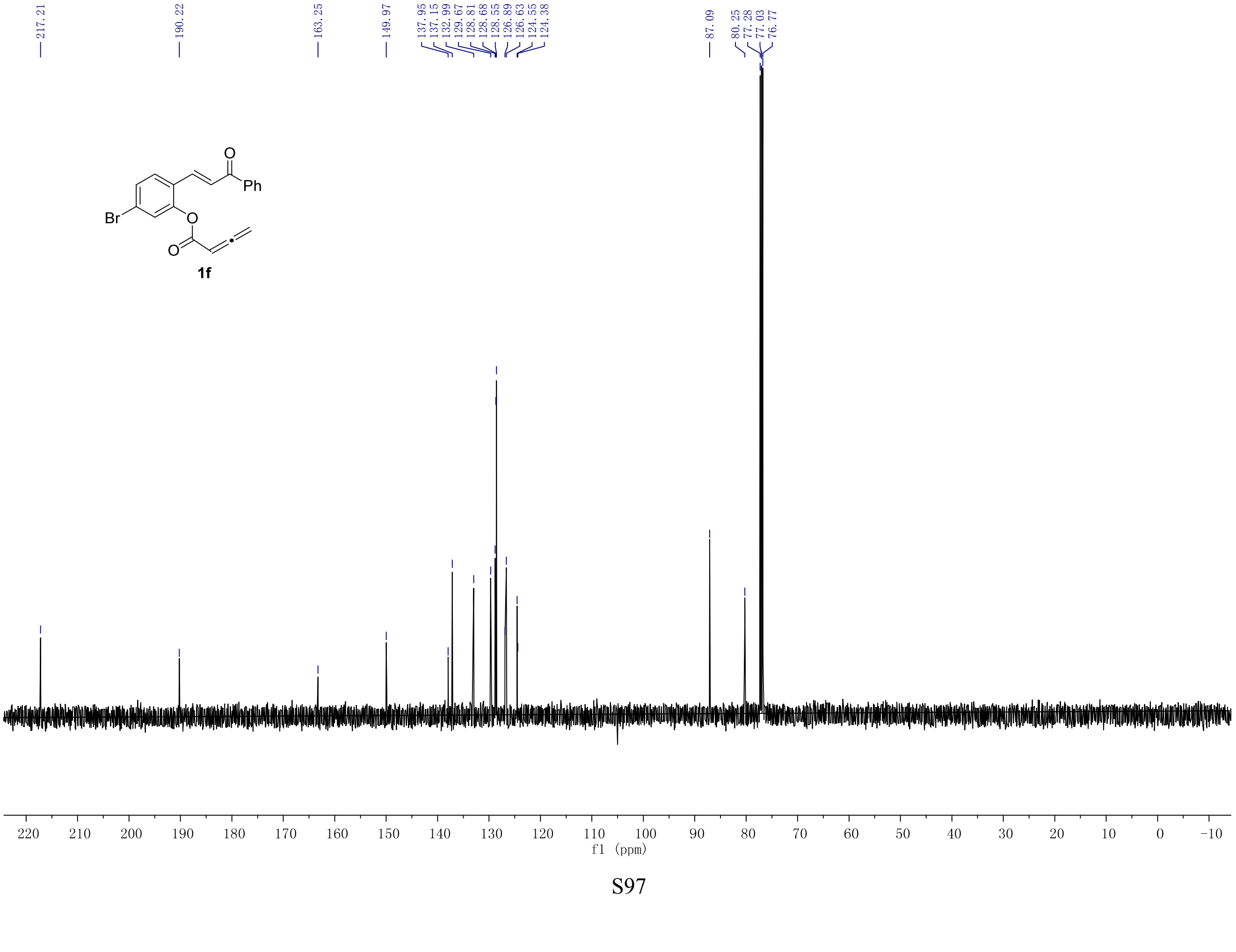

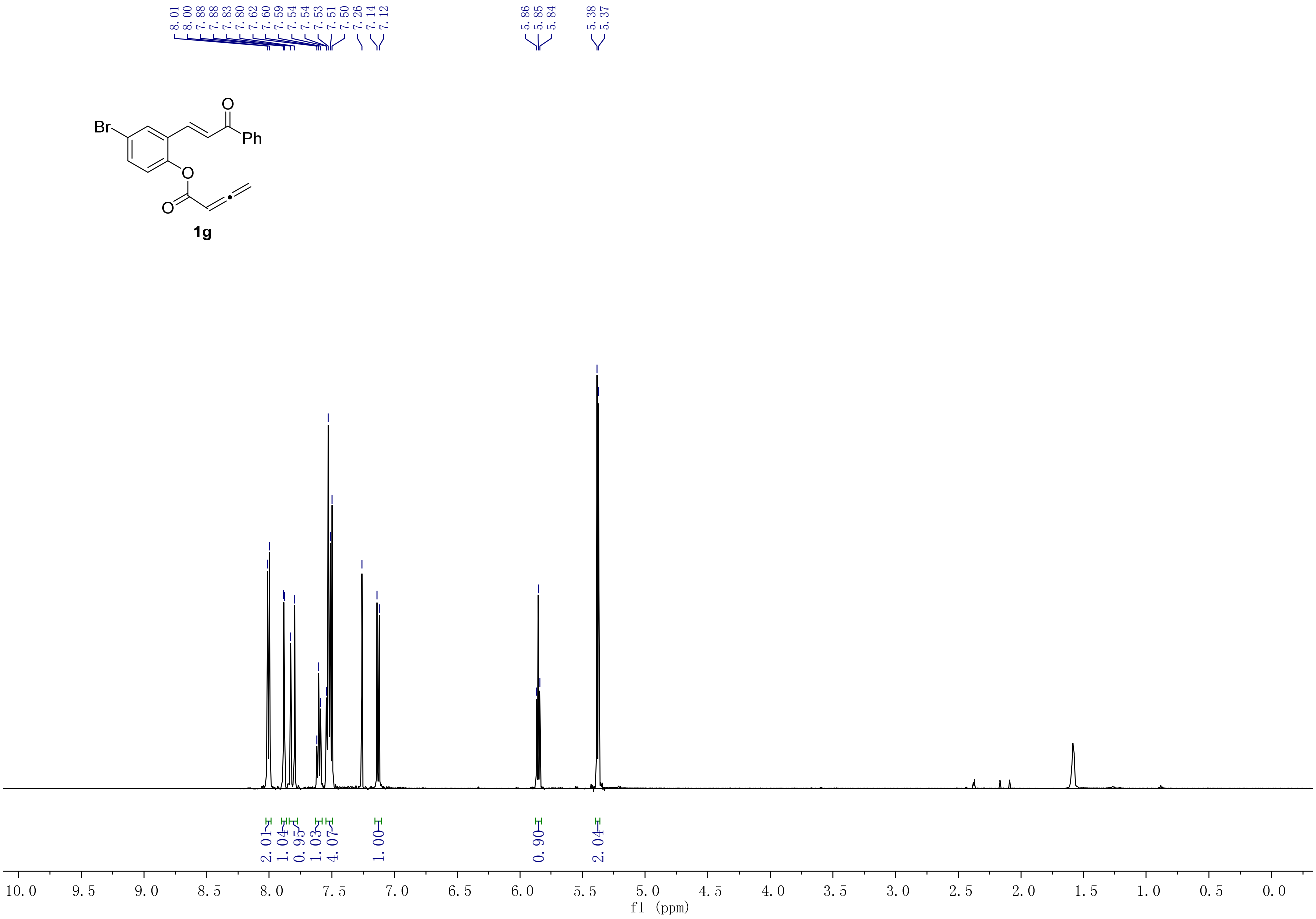
8.01
8.00
7.88
7.83
7.80
7.62
7.60
7.59
7.54
7.54
7.54
7.53
7.51
7.50
7.26
7.14
7.12
5.86
5.85
5.84
5.38
5.37
Br
O
Ph
O
O
1g
2.01
1.04
0.95
1.03
4.07
1.00
0.90
2.04
f1 (ppm)

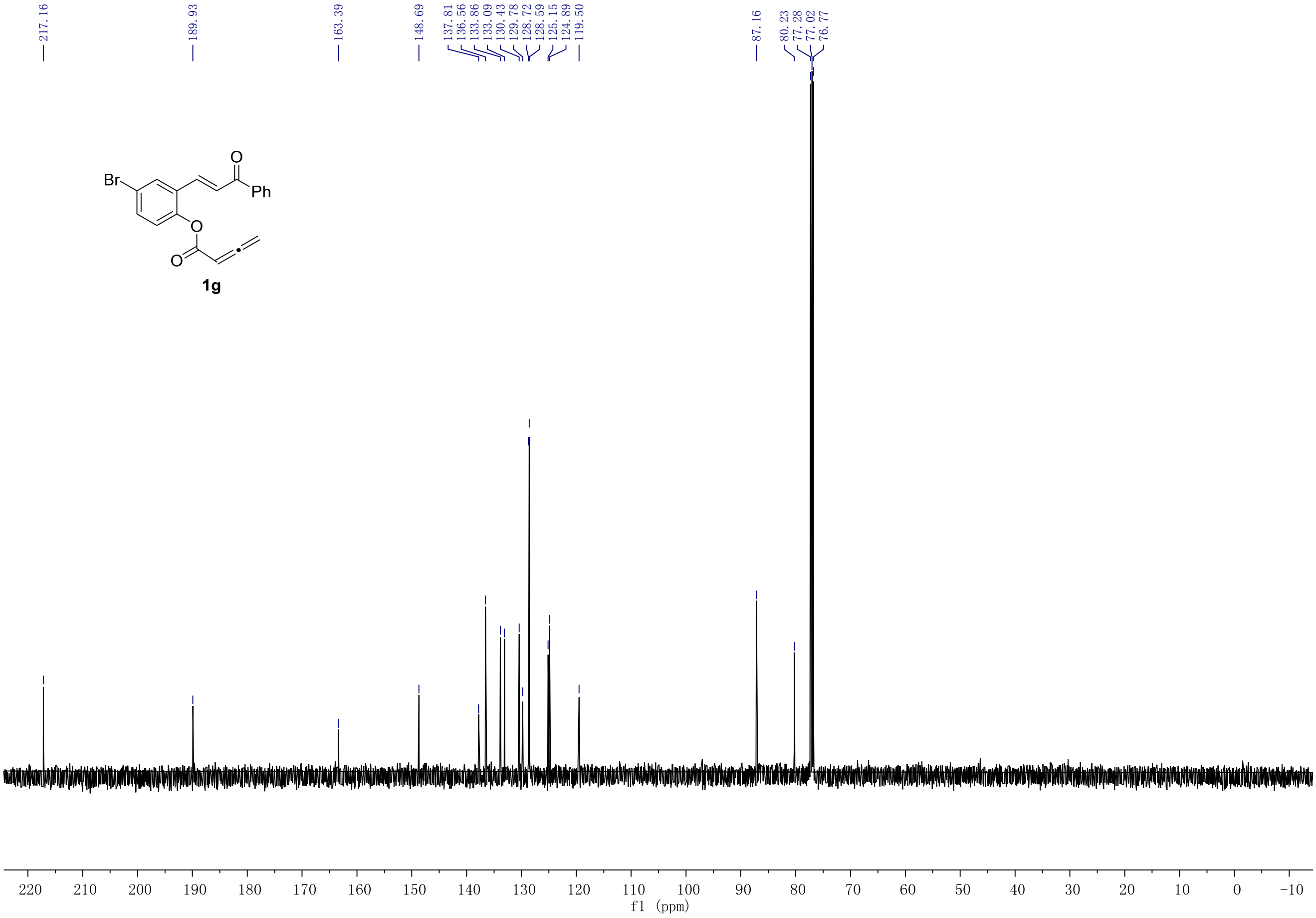
217.16
189.93
163.39
148.69
137.81
136.56
133.86
133.09
130.43
129.78
128.72
128.59
125.15
124.89
119.50
87.16
80.23
77.28
77.02
76.77
Br
O
Ph
O
O
1g
f1 (ppm)

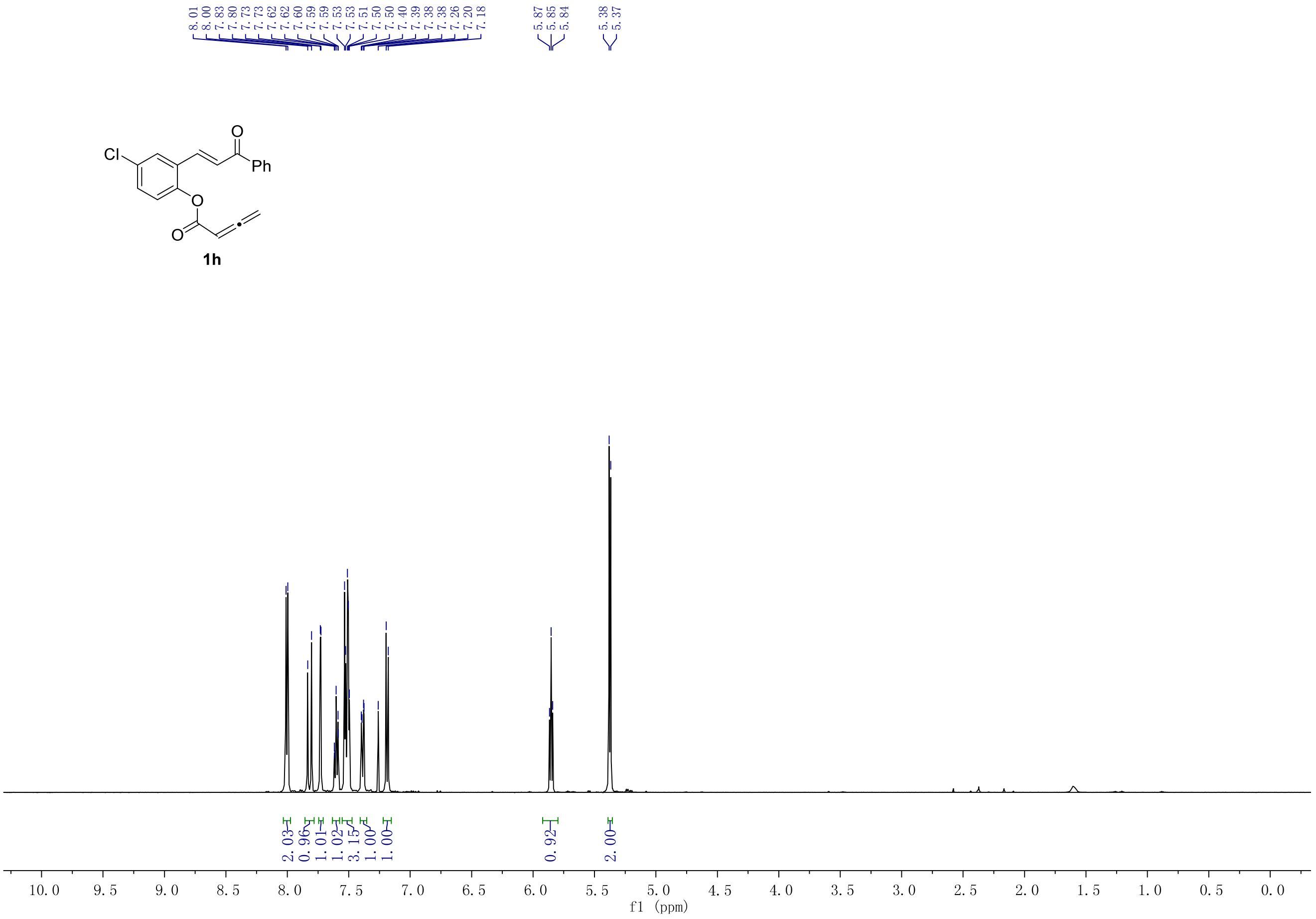
8.01 8.00 7.83 7.80 7.73 7.73 7.62 7.60 7.59 7.59 7.53 7.53 7.51 7.50 7.50 7.40 7.39 7.38 7.38 7.38 7.26 7.20 7.18
5.87 5.85 5.84
5.38 5.37
Cl
O
Ph
O
O
1h
2.03 0.96 1.01 1.02 3.15 1.00 1.00
0.92
2.00
10.0 9.5 9.0 8.5 8.0 7.5 7.0 6.5 6.0 5.5 5.0 4.5 4.0 3.5 3.0 2.5 2.0 1.5 1.0 0.5 0.0
f1 (ppm)

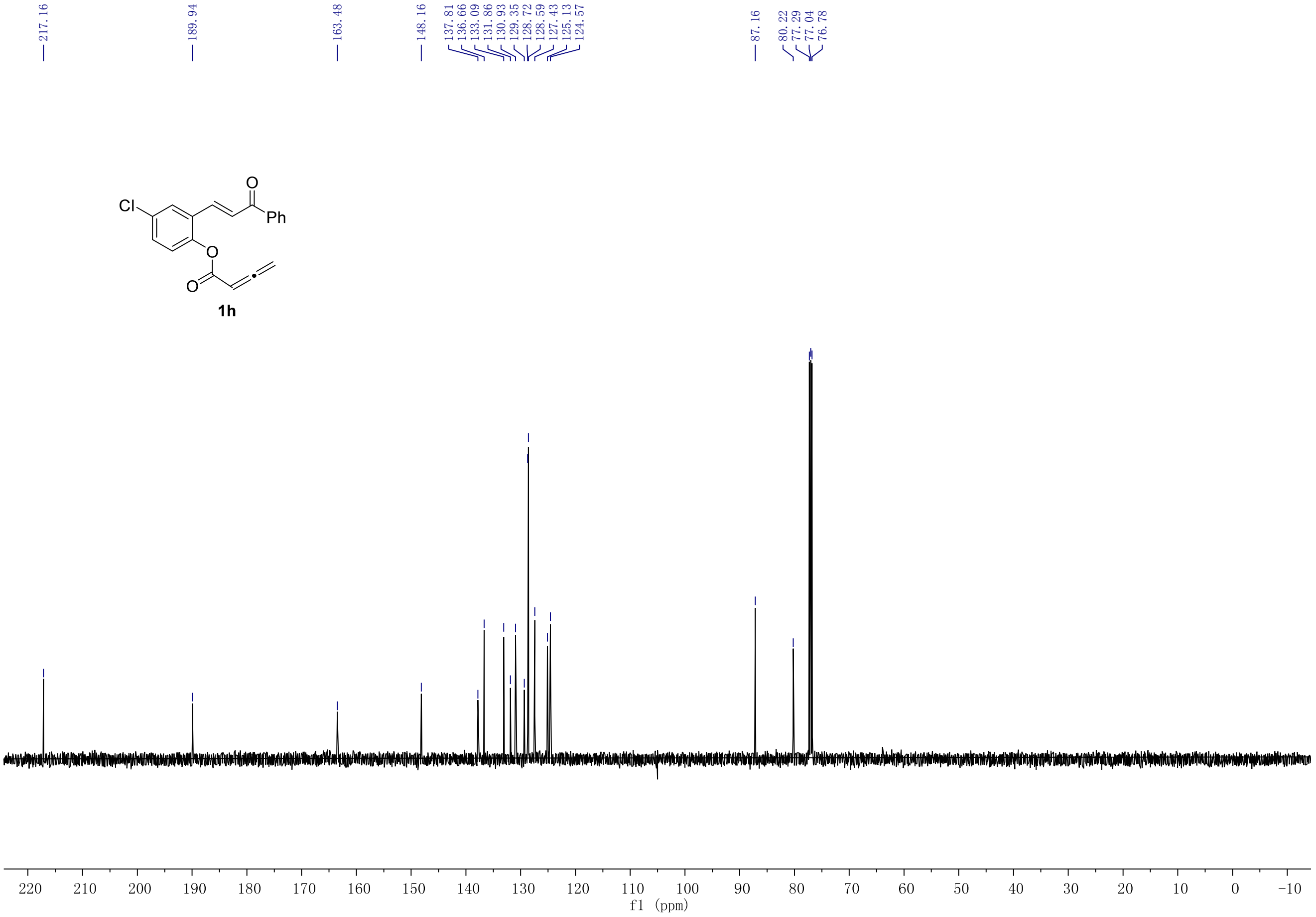
217.16
189.94
163.48
148.16
137.81 136.66 133.09 131.86 130.93 129.35 128.72 128.59 127.43 125.13 124.57
87.16
80.22 77.29 77.04 76.78
Cl
O
Ph
O
O
1h
220 210 200 190 180 170 160 150 140 130 120 110 100 90 80 70 60 50 40 30 20 10 0 -10
f1 (ppm)

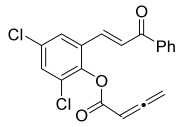

1i

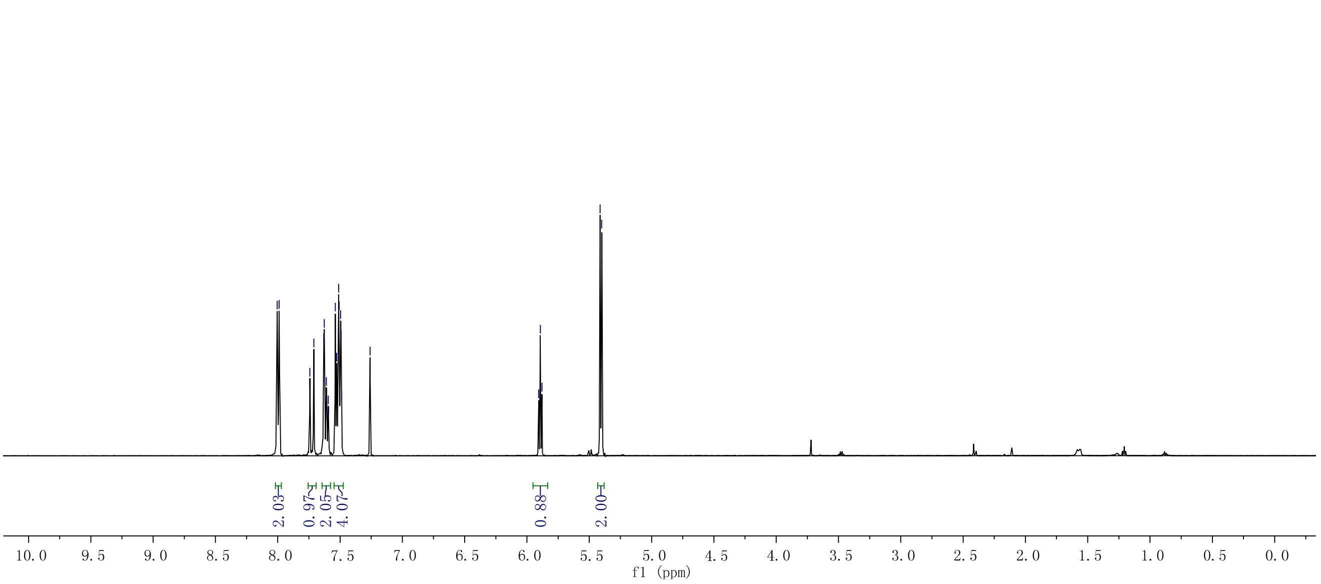
8.00
7.99
7.63
7.54
7.51
7.51
7.50
7.28
5.91
5.89
5.88
5.41
5.40
2.03
0.97
2.05
4.07
0.88
2.00
10.0 9.5 9.0 8.5 8.0 7.5 7.0 6.5 6.0 5.5 5.0 4.5 4.0 3.5 3.0 2.5 2.0 1.5 1.0 0.5 0.0
f1 (ppm)

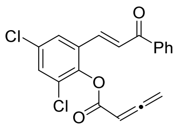

1i

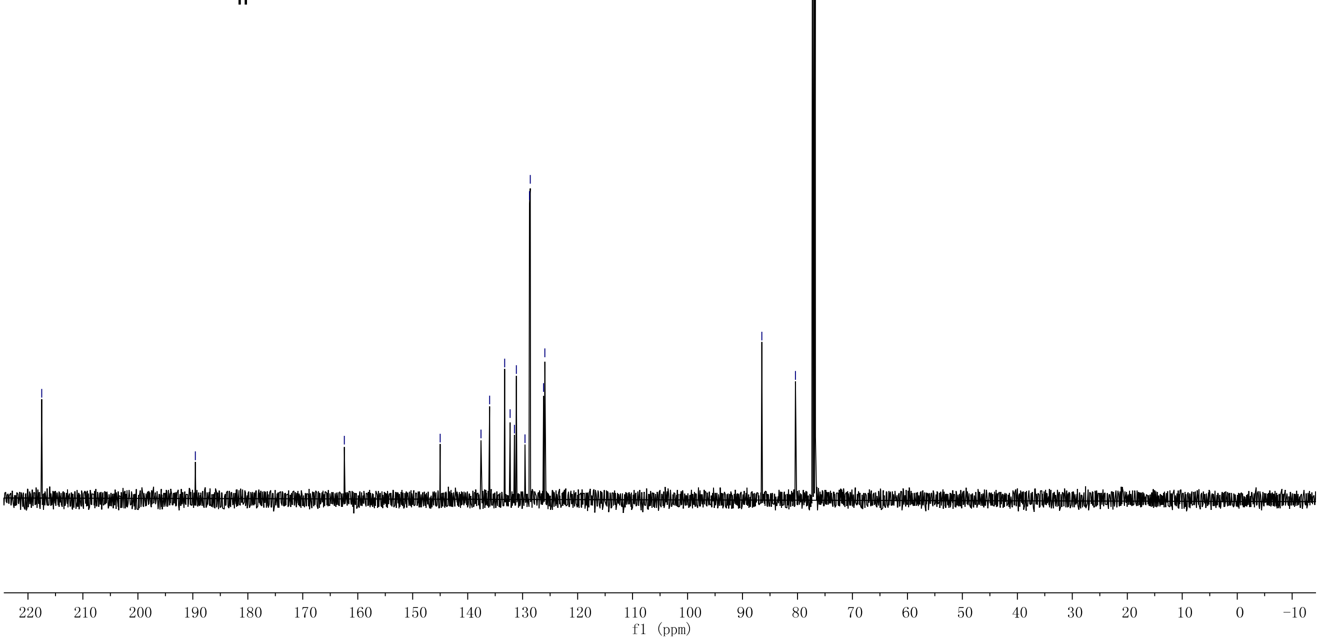
217.50
189.53
162.45
144.99
137.57
136.00
133.27
132.28
131.50
131.15
129.58
128.76
128.61
126.20
125.96
86.49
80.38
77.28
77.03
76.77
220 210 200 190 180 170 160 150 140 130 120 110 100 90 80 70 60 50 40 30 20 10 0 -10
f1 (ppm)

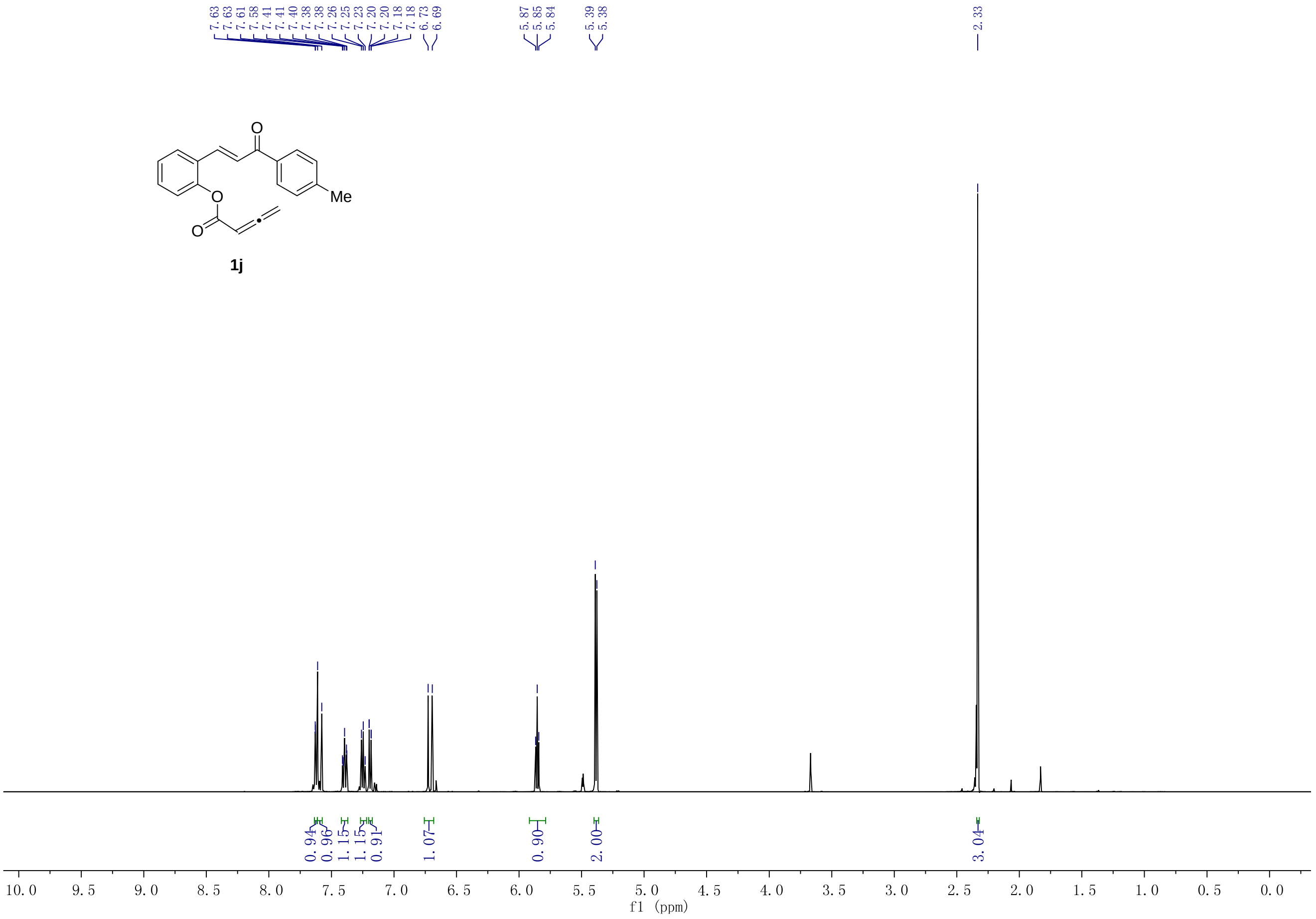
7.63
7.63
7.61
7.58
7.41
7.41
7.40
7.38
7.38
7.26
7.25
7.23
7.20
7.20
7.18
6.73
6.69
5.87
5.85
5.84
5.39
5.38
2.33
O
O
O
Me
1j
0.94
0.96
1.15
1.15
0.91
1.07
0.90
2.00
3.04
10.0 9.5 9.0 8.5 8.0 7.5 7.0 6.5 6.0 5.5 5.0 4.5 4.0 3.5 3.0 2.5 2.0 1.5 1.0 0.5 0.0
f1 (ppm)

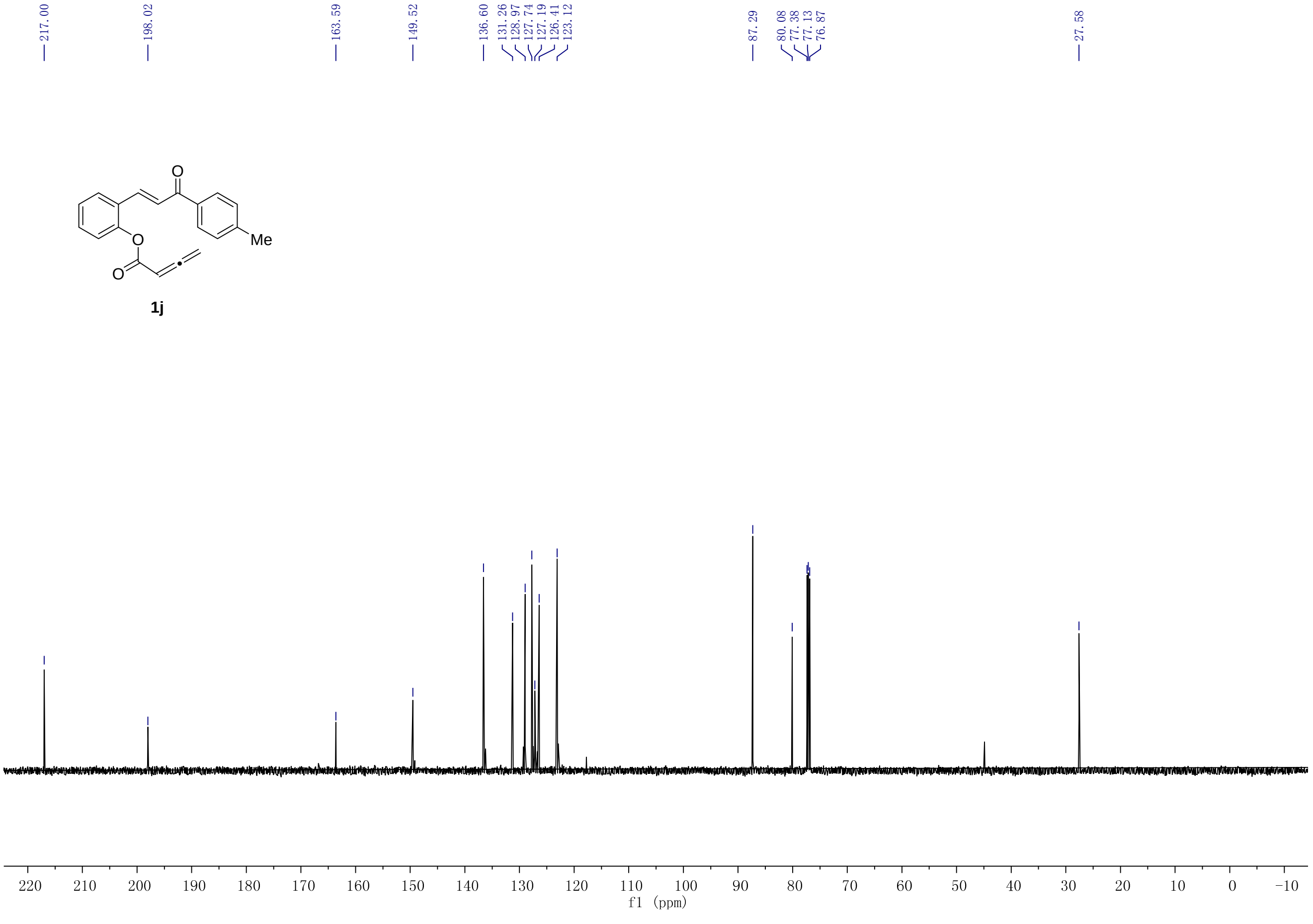
217.00
198.02
163.59
149.52
136.60
131.26
128.97
127.74
127.19
126.41
123.12
87.29
80.08
77.38
77.13
76.87
27.58
O
O
O
Me
1j
220 210 200 190 180 170 160 150 140 130 120 110 100 90 80 70 60 50 40 30 20 10 0 -10
f1 (ppm)

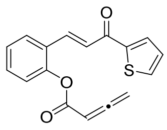

1k

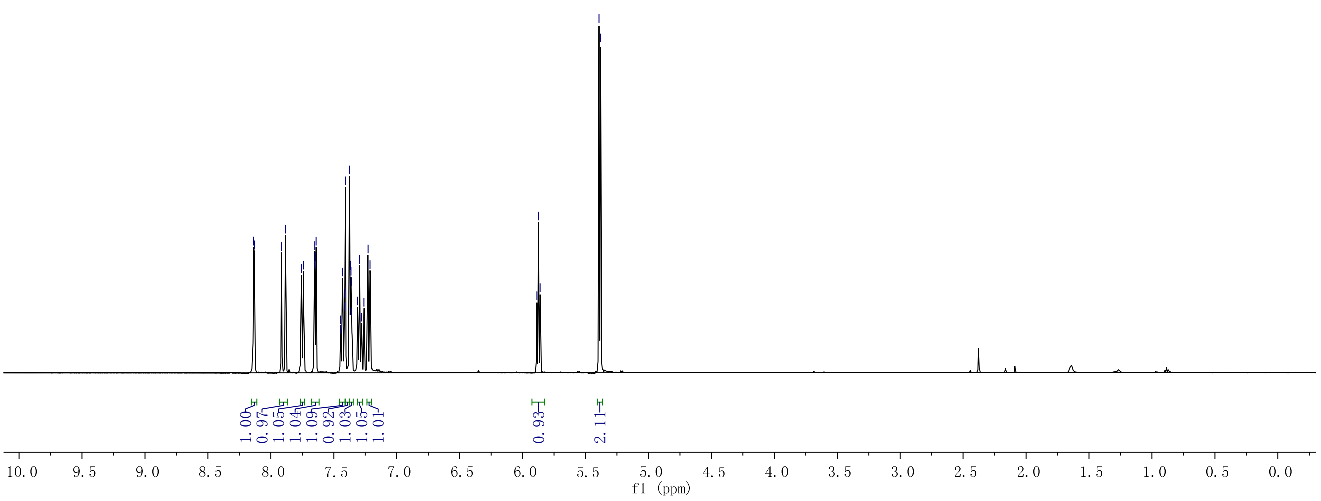

8.14
8.13
7.91
7.88
7.76
7.74
7.65
7.65
7.64
7.64
7.43
7.41
7.41
7.38
7.37
7.36
7.36
7.31
7.29
7.23
7.21
5.89
5.87
5.86
5.39
5.38
1.00
0.97
1.05
1.04
1.09
0.92
1.03
1.05
1.01
0.93
2.11
10.0 9.5 9.0 8.5 8.0 7.5 7.0 6.5 6.0 5.5 5.0 4.5 4.0 3.5 3.0 2.5 2.0 1.5 1.0 0.5 0.0
f1 (ppm)

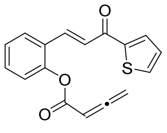

1k

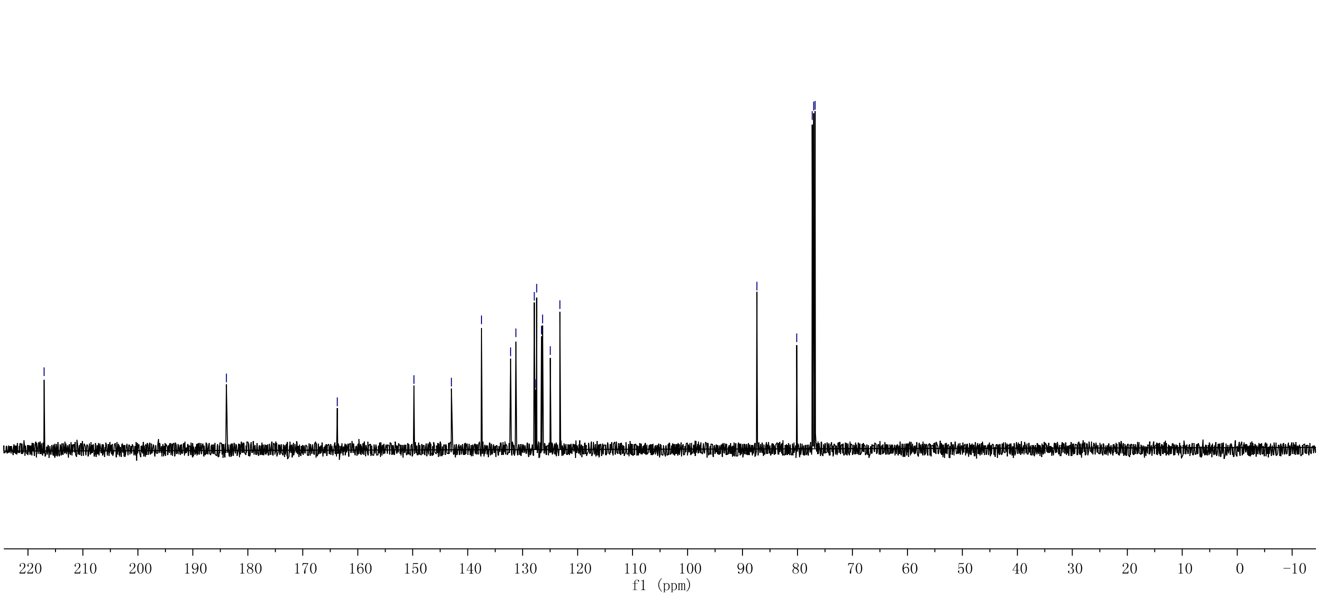

217.05
183.91
163.73
149.78
142.96
137.48
132.18
131.24
127.88
127.70
127.46
126.57
126.37
124.96
123.23
87.38
80.14
77.31
77.05
76.80
220 210 200 190 180 170 160 150 140 130 120 110 100 90 80 70 60 50 40 30 20 10 0 -10
f1 (ppm)

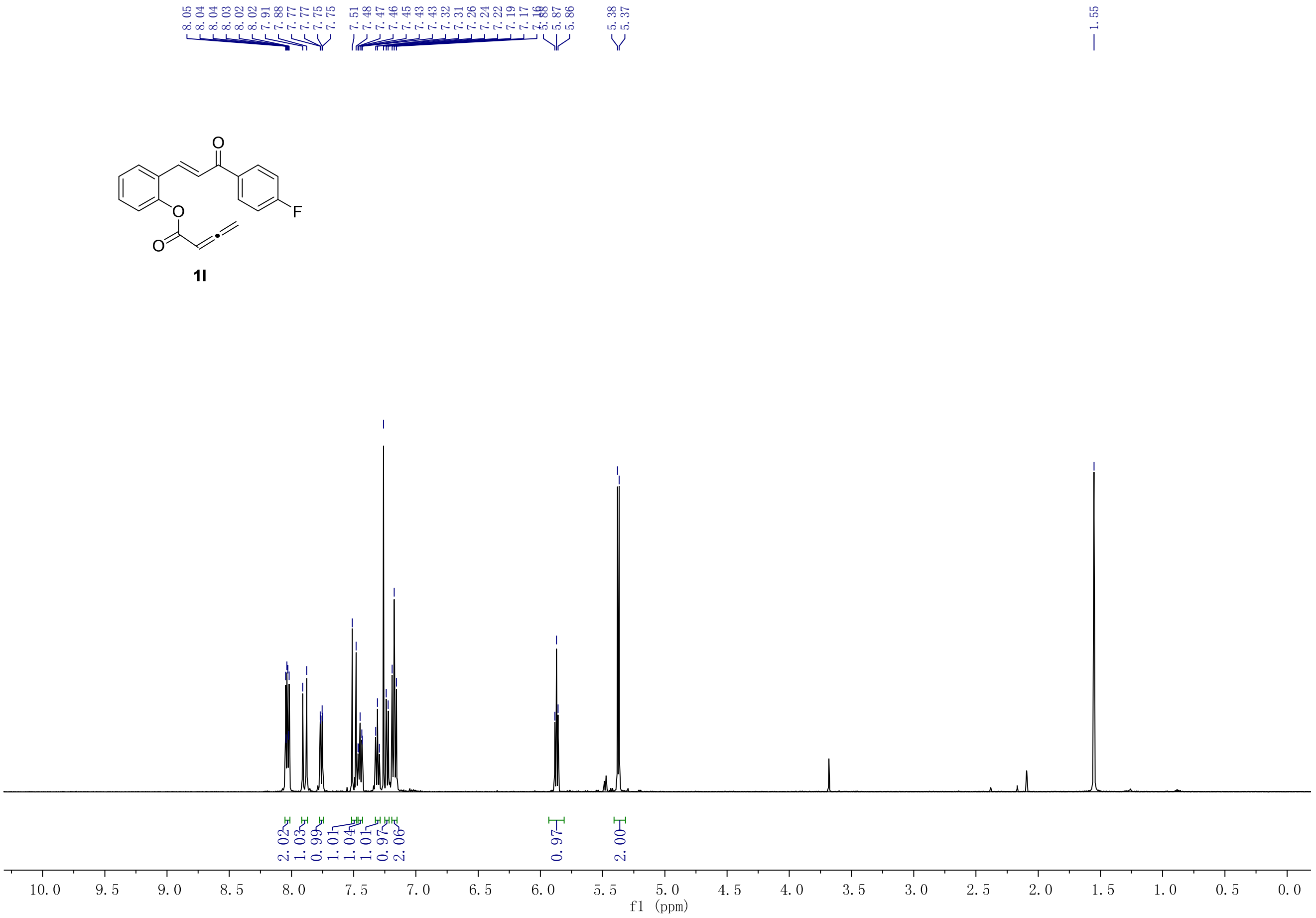
O
F
O
O
1l
f1 (ppm)

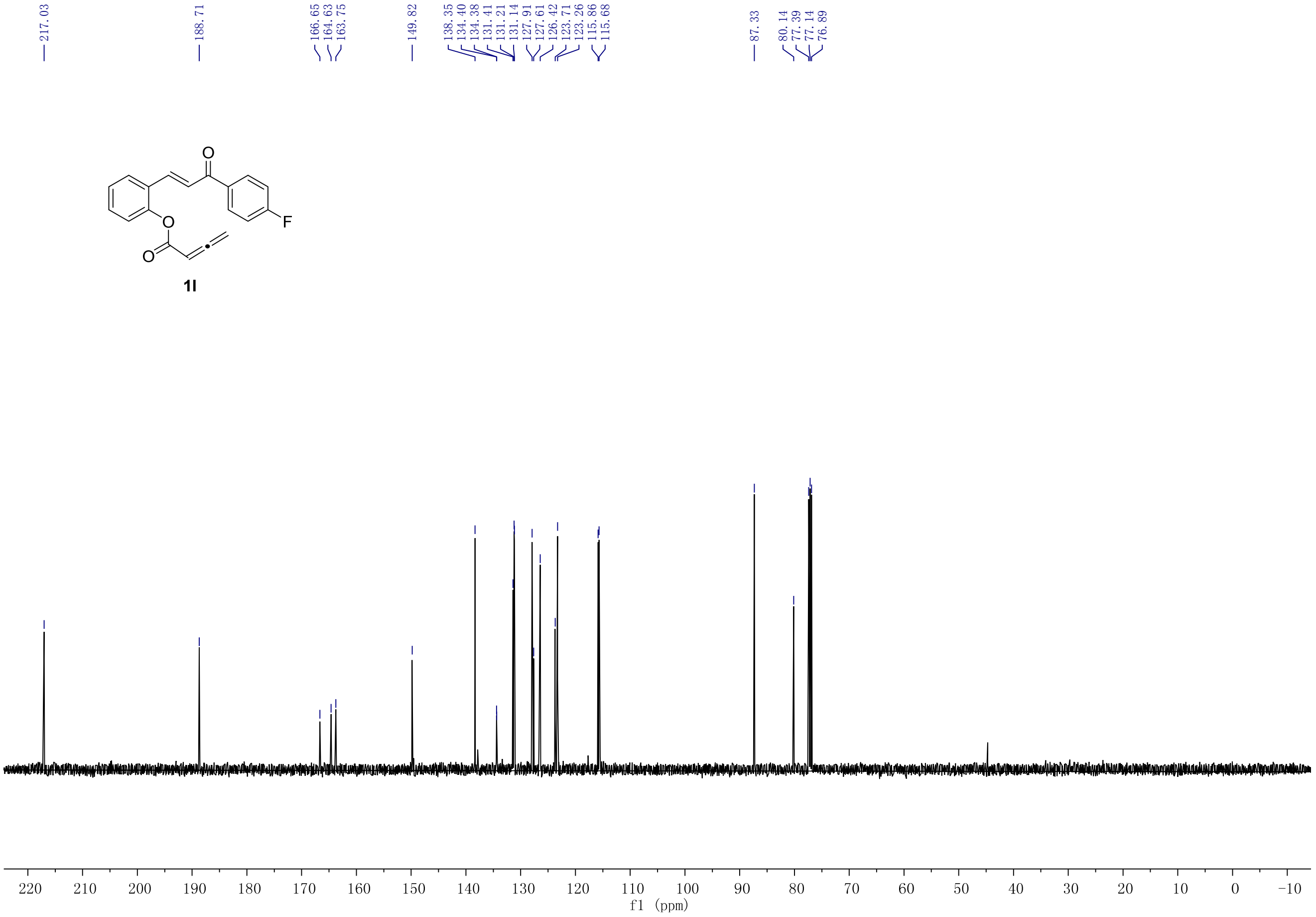
O
F
O
O
1l
f1 (ppm)

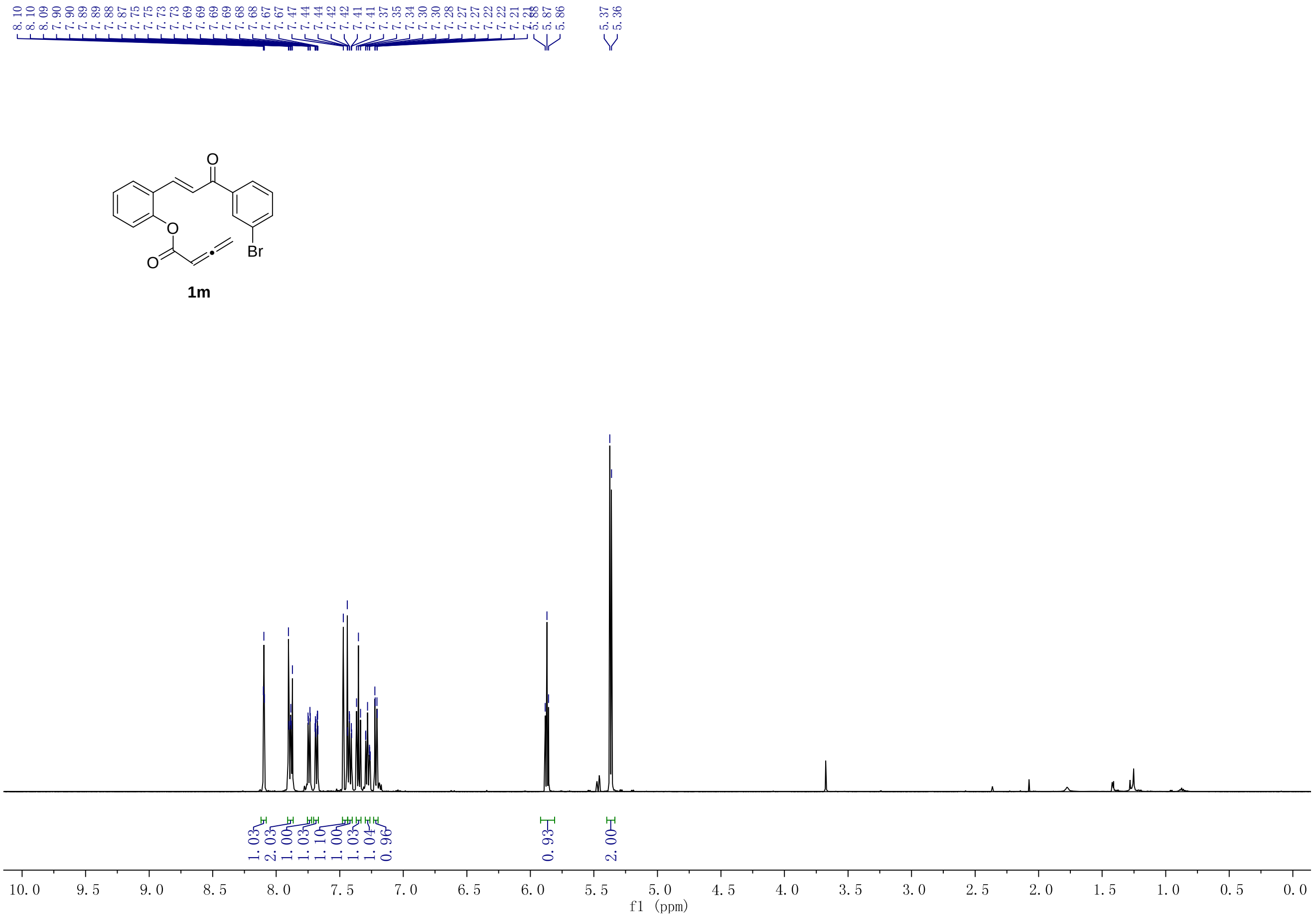

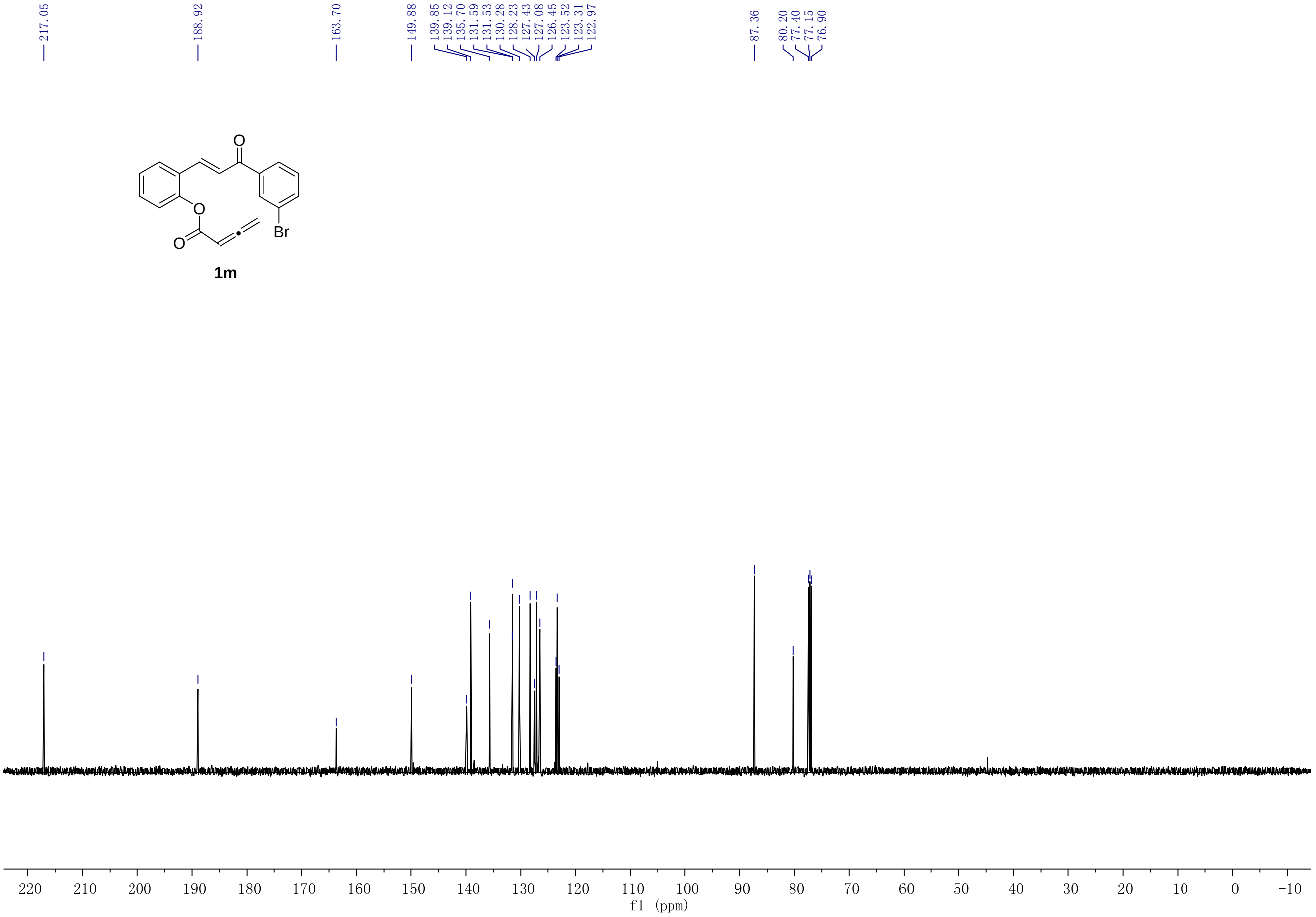

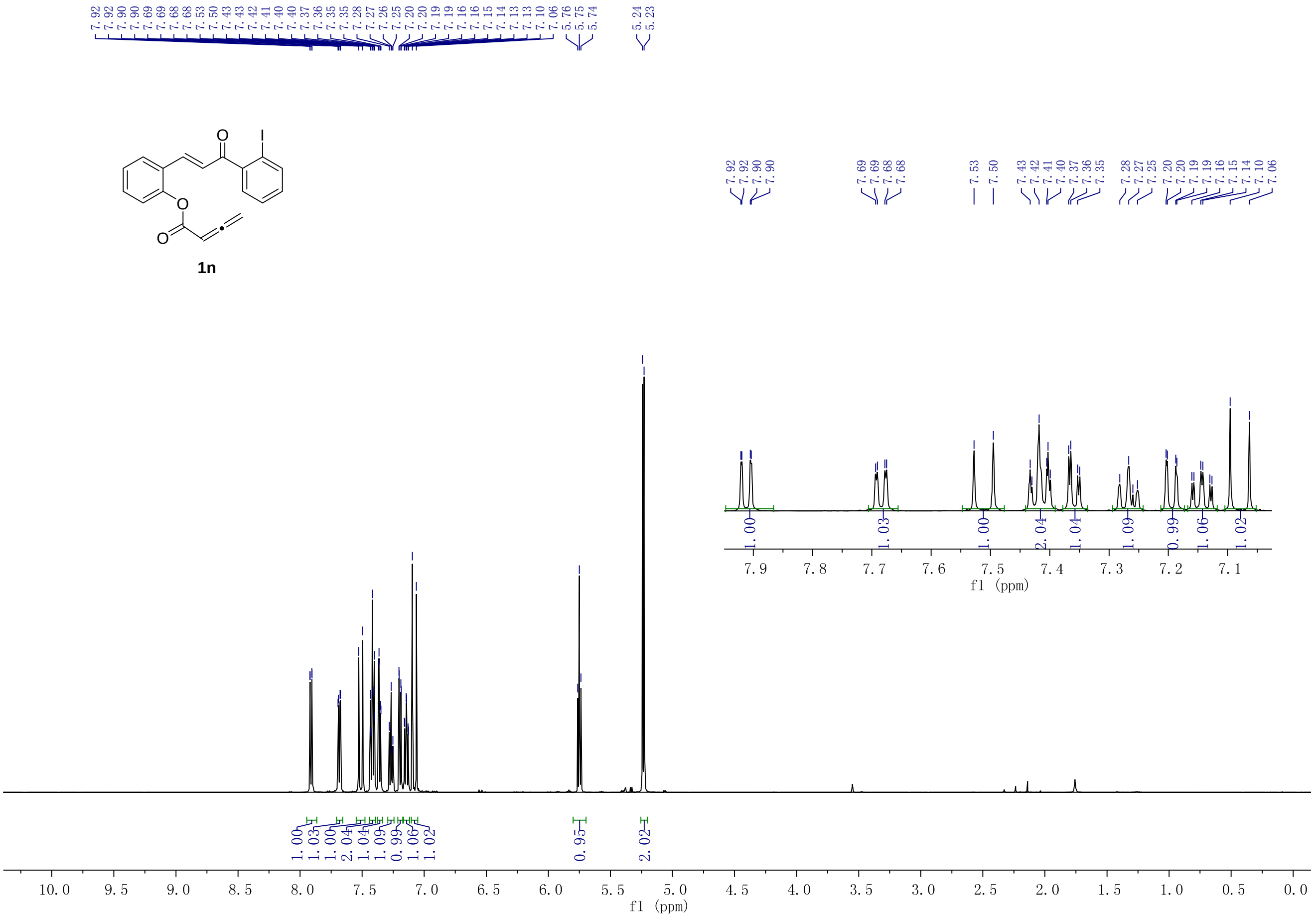
1n
f1 (ppm)

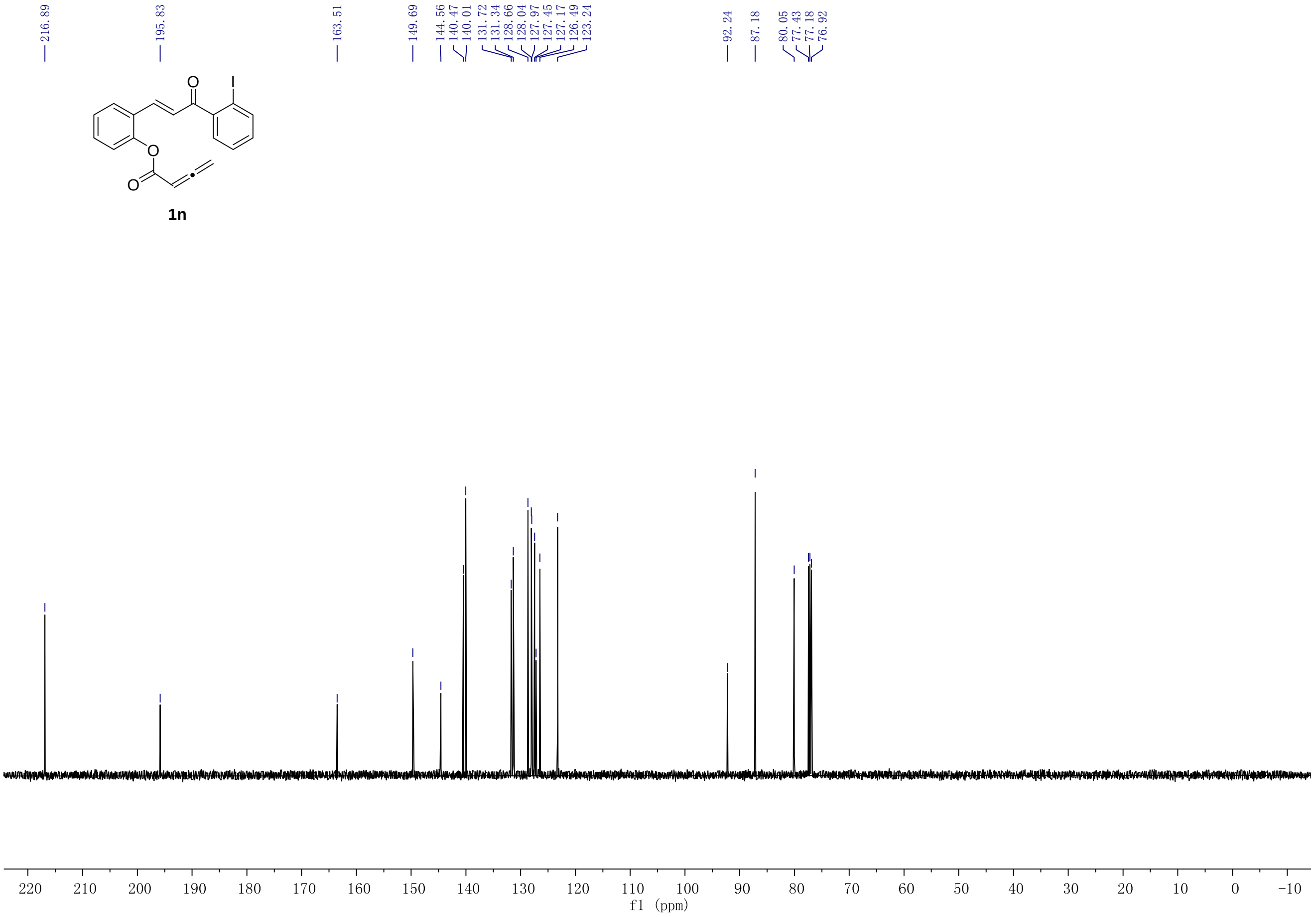
1n
f1 (ppm)

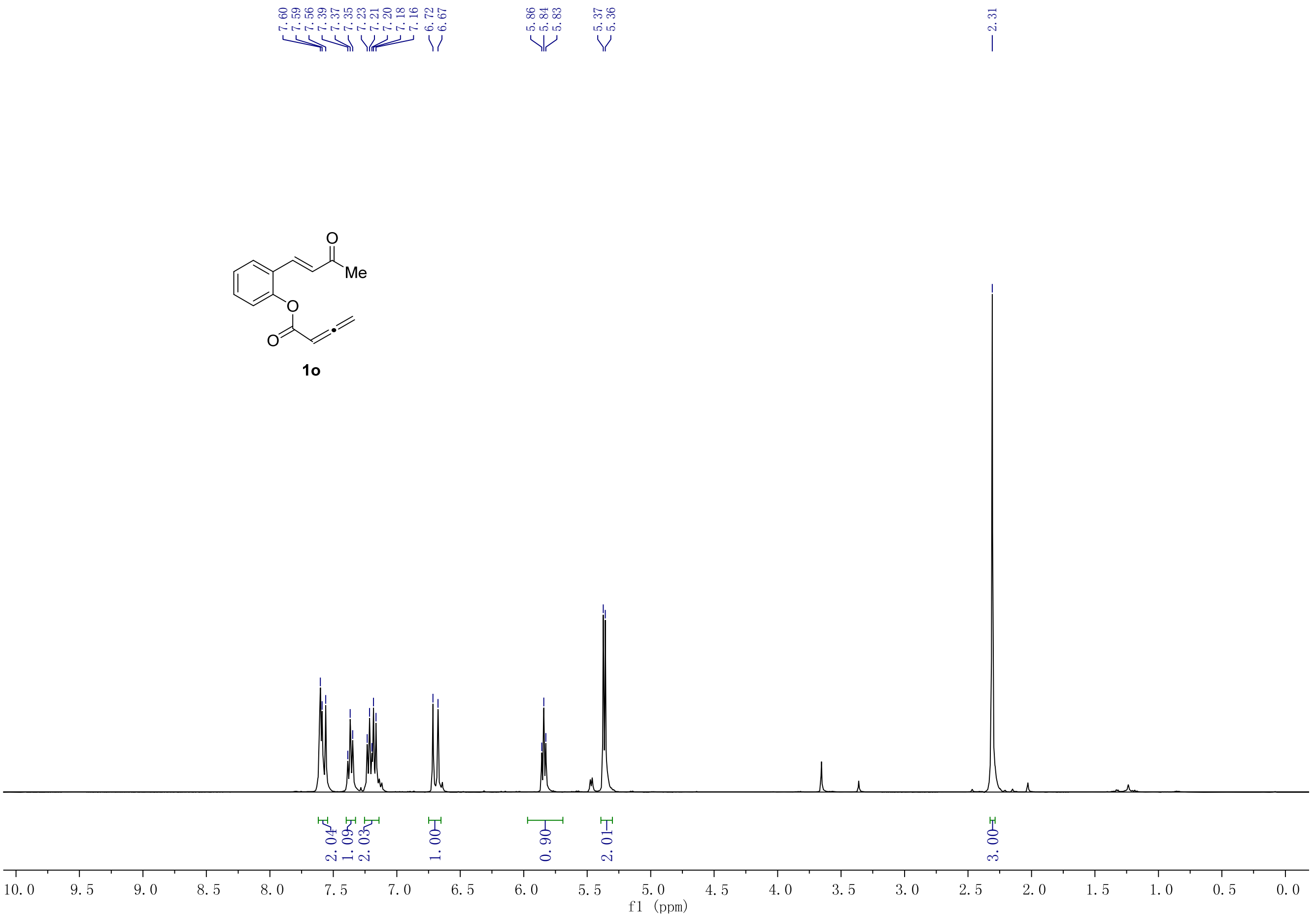
7.60 7.59 7.56 7.39 7.37 7.35 7.23 7.21 7.20 7.18 7.16 6.72 6.67
5.86 5.84 5.83
5.37 5.36
2.31
O
Me
O
O
1o
2.04 1.09 2.03 1.00 0.90 2.01 3.00
f1 (ppm)

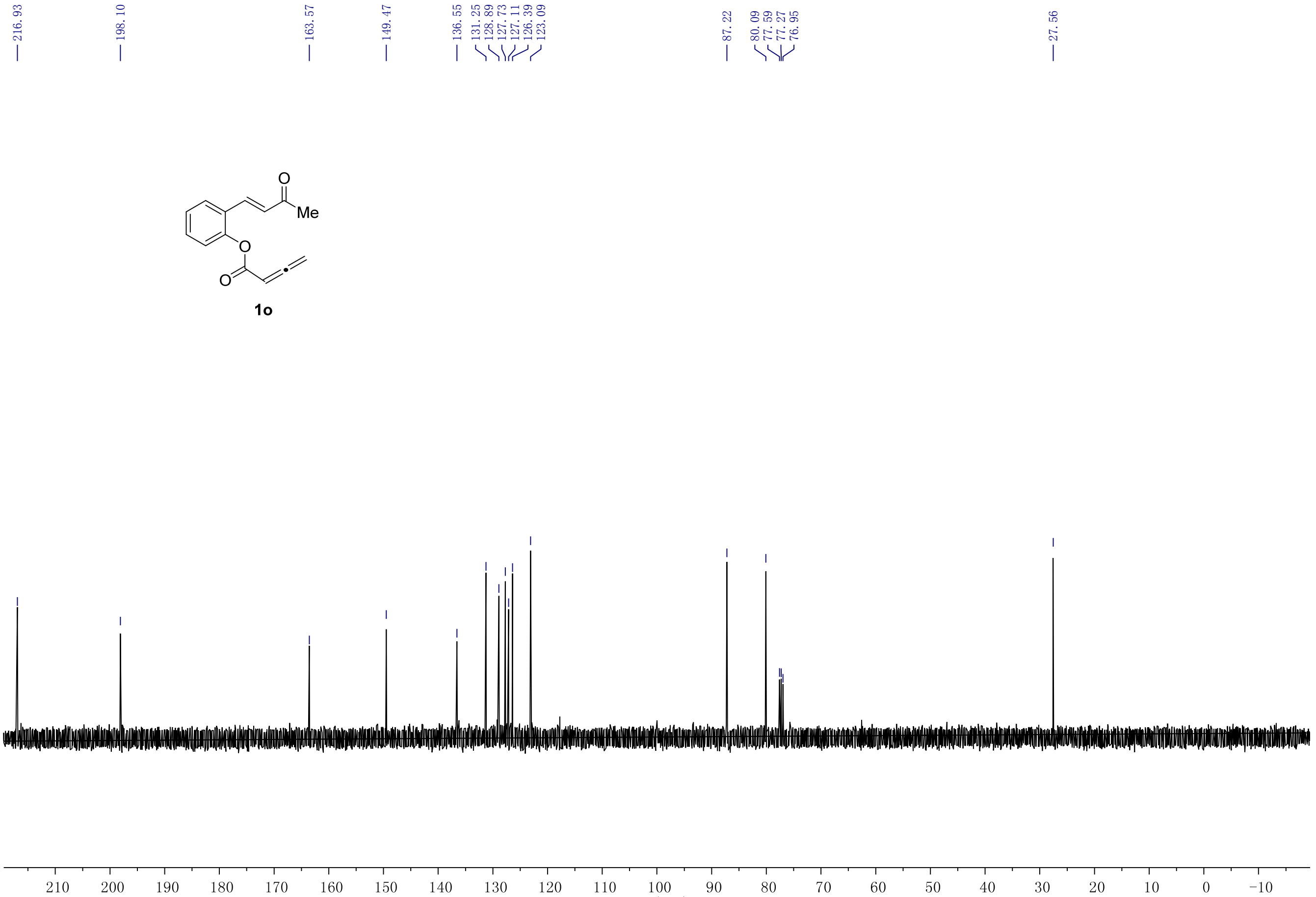
216.93
198.10
163.57
149.47
136.55 131.25 128.89 127.73 127.11 126.39 123.09
87.22
80.09 77.59 77.27 76.95
27.56
O
Me
O
O
1o
f1 (ppm)

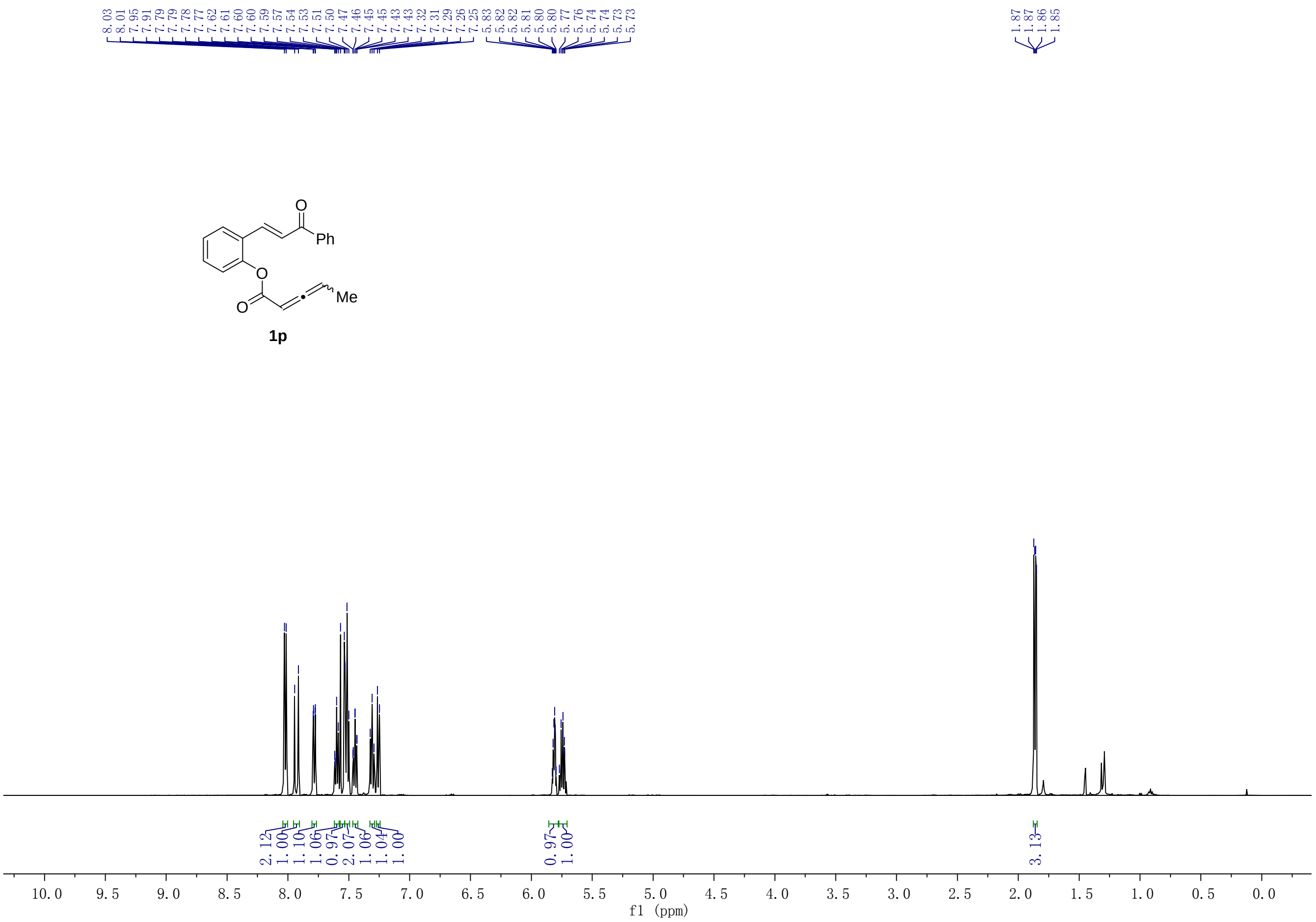

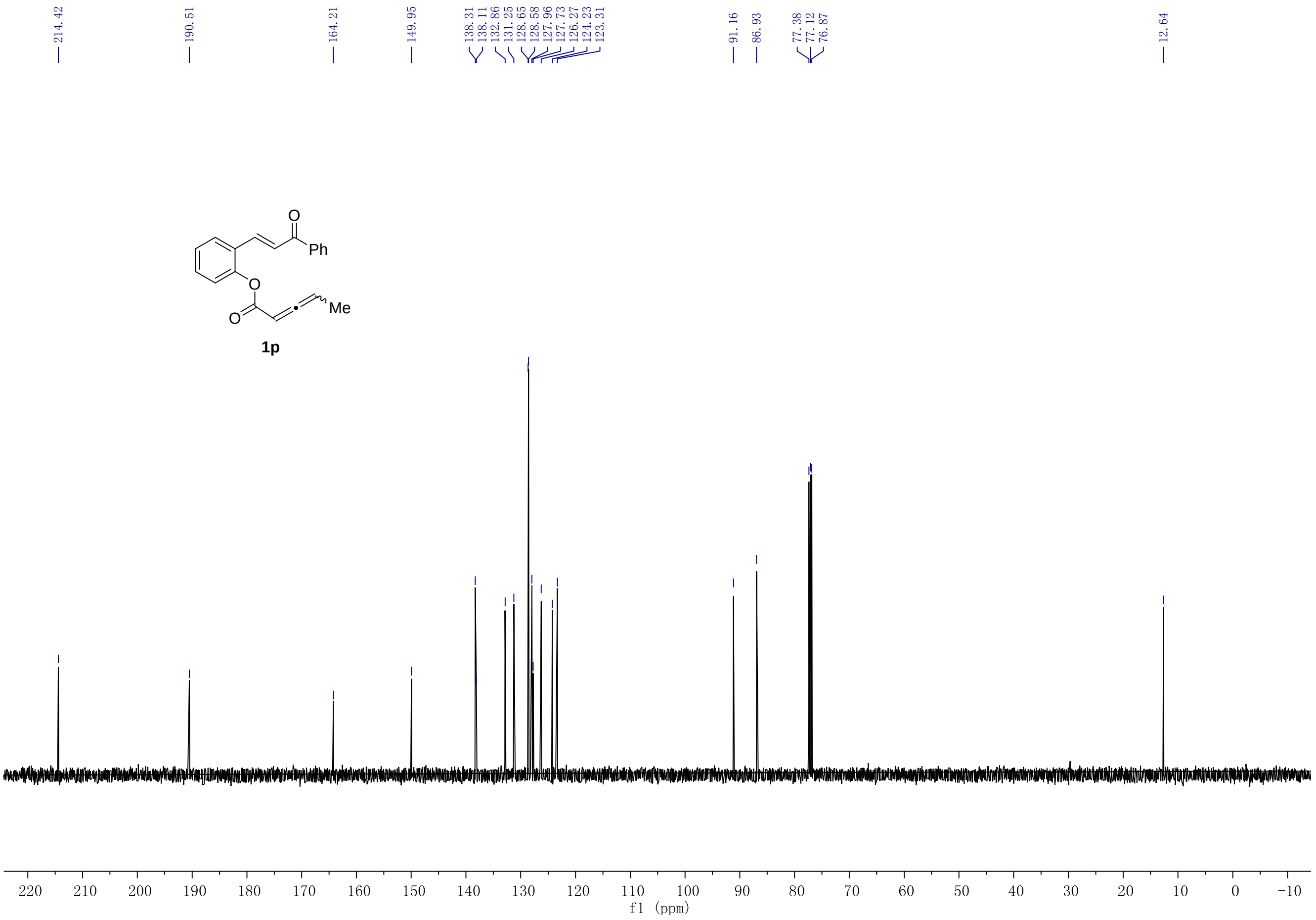

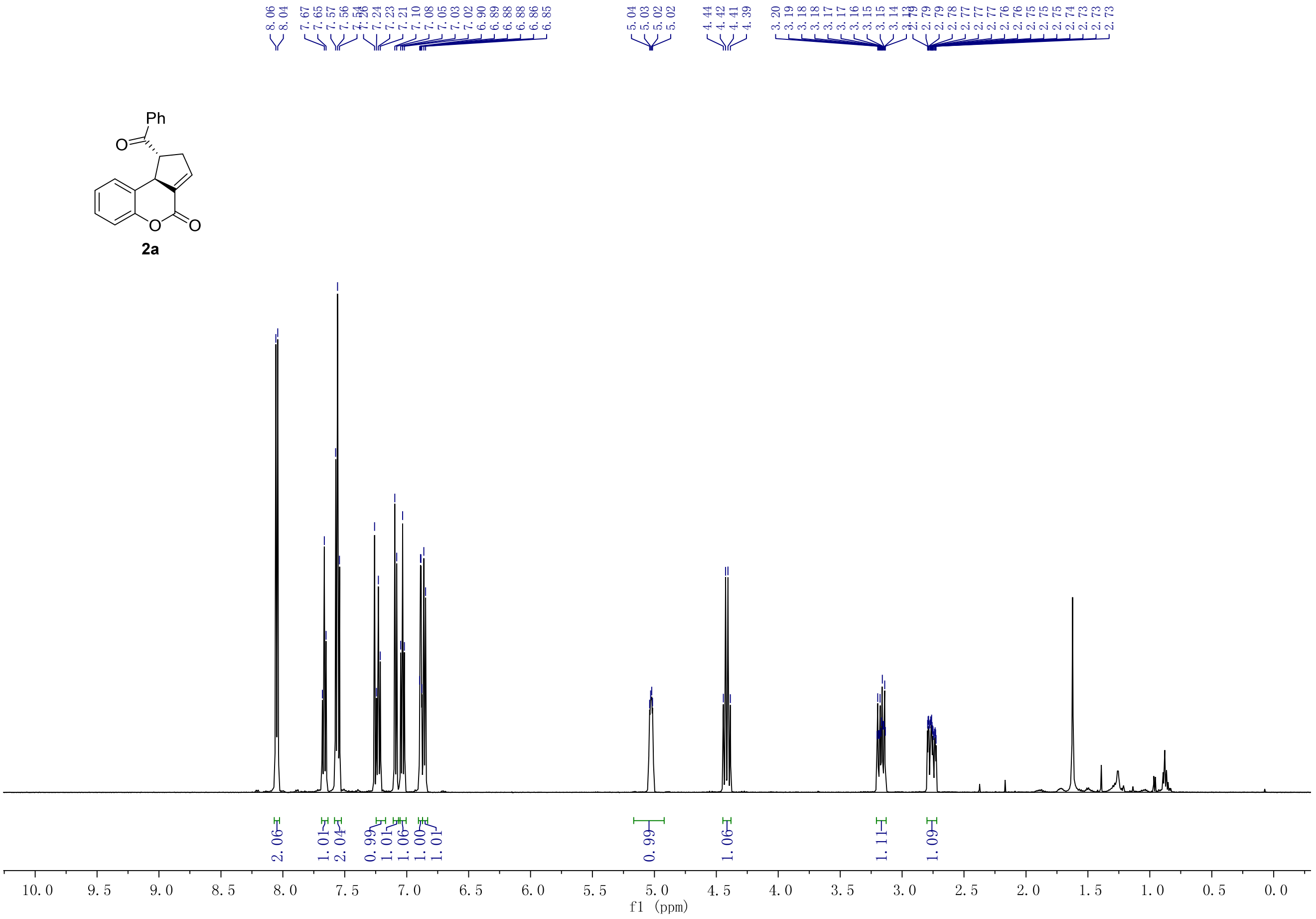
Ph
O
O
O
2a
f1 (ppm)

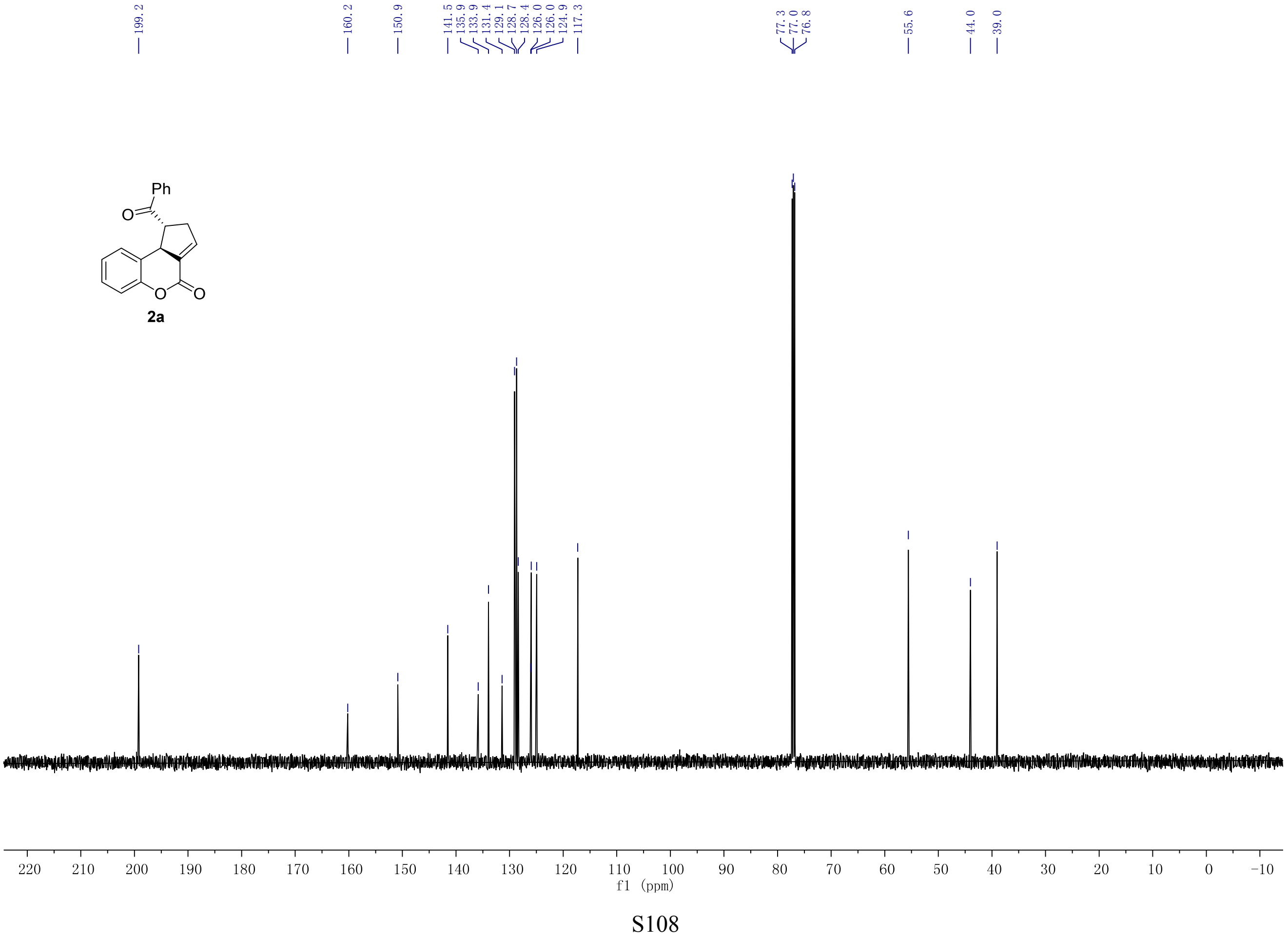
Ph
O
O
O
2a
f1 (ppm)

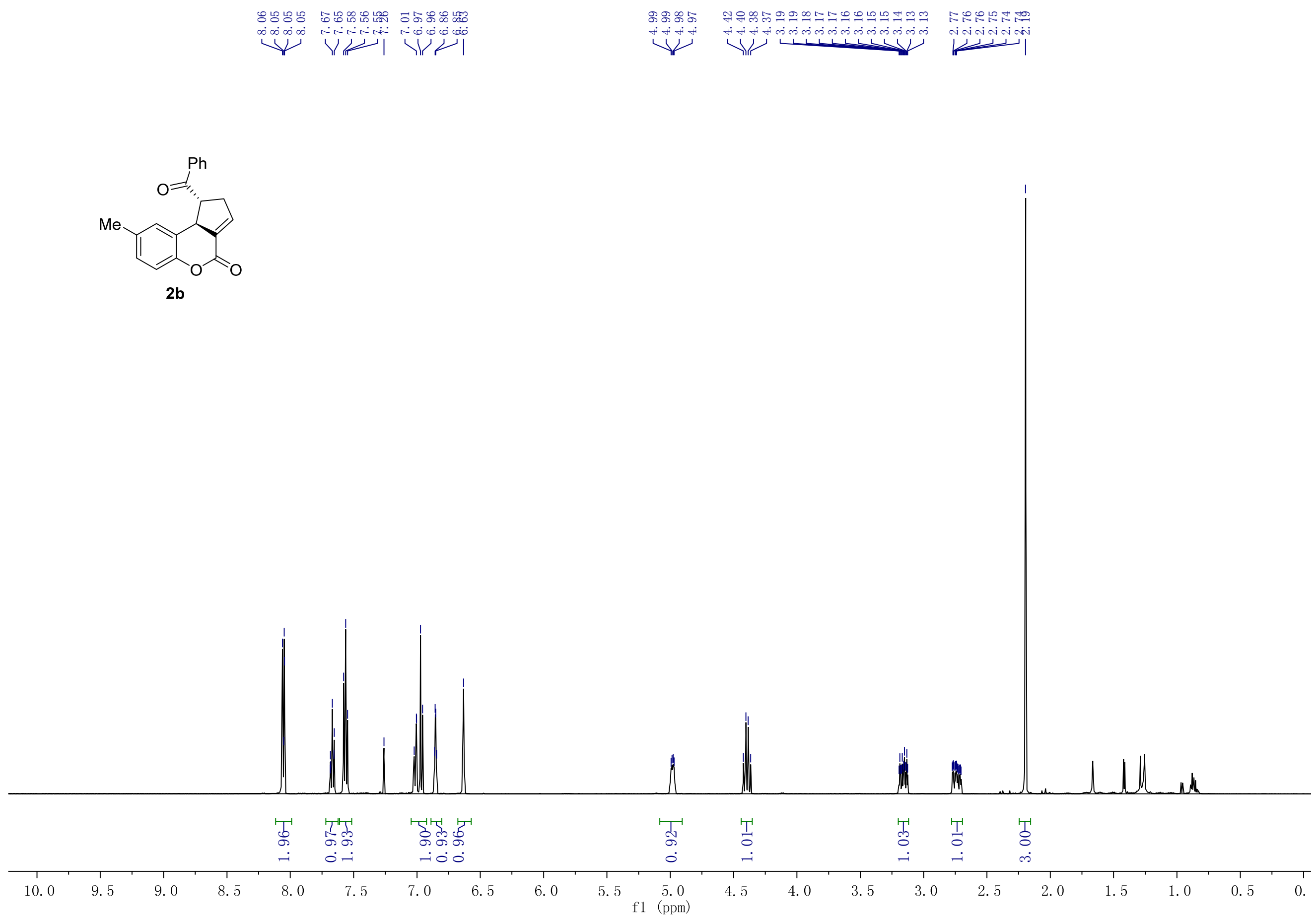
8.06 8.05 8.05 8.05
7.67 7.65 7.58 7.56
7.01 6.97 6.96 6.86
4.99 4.99 4.98 4.97
4.42 4.40 4.38 4.37 3.19 3.19 3.18 3.17 3.17 3.16 3.16 3.15 3.15 3.14 3.13 3.13
2.77 2.76 2.76 2.75 2.74
Ph
O
Me
O
O
2b
1.96
0.97
1.93
1.90
0.93
0.96
0.92
1.01
1.03
1.01
3.00
10.0 9.5 9.0 8.5 8.0 7.5 7.0 6.5 6.0 5.5 5.0 4.5 4.0 3.5 3.0 2.5 2.0 1.5 1.0 0.5 0.
f1 (ppm)

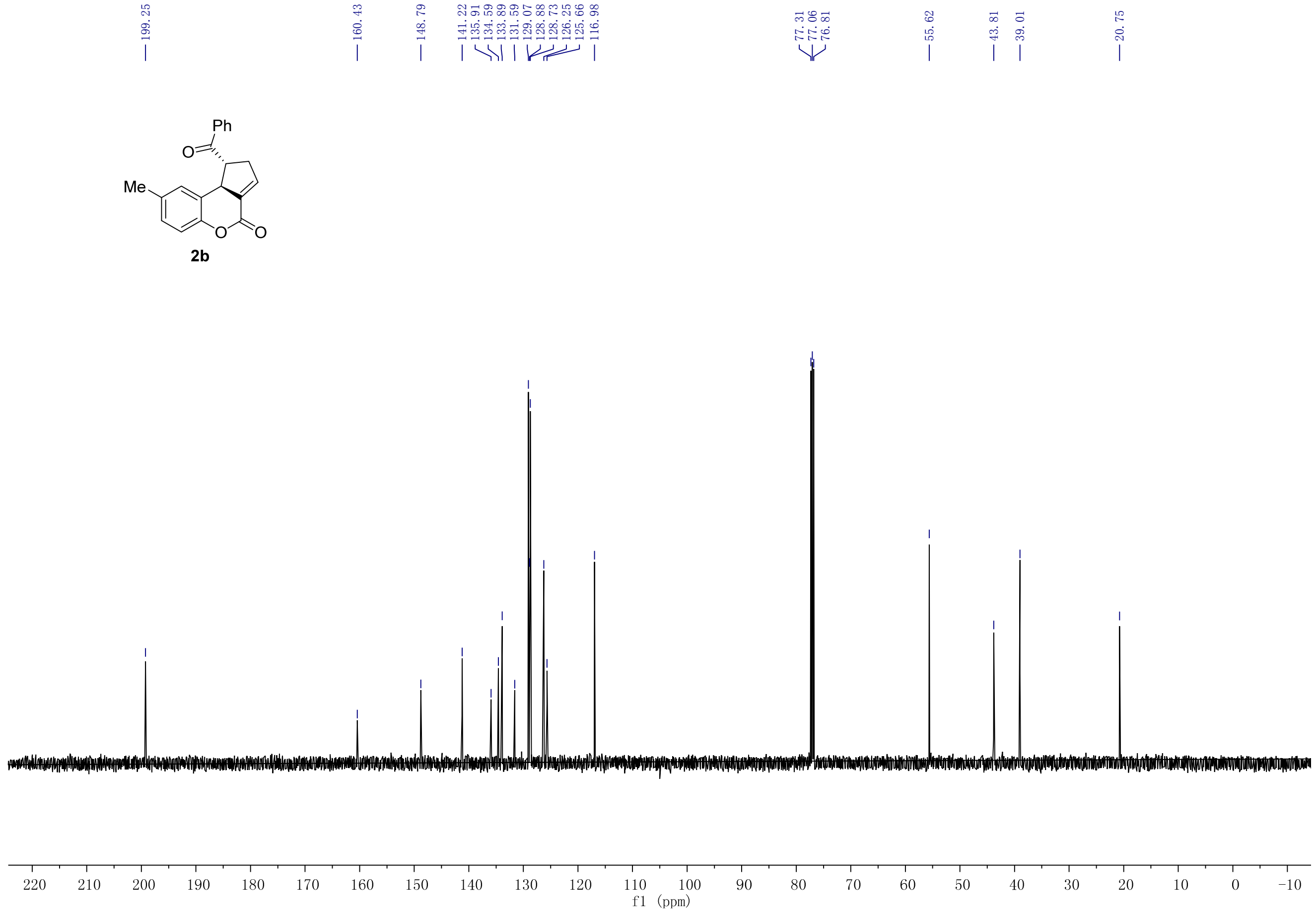
199.25
160.43
148.79
141.22 135.91 134.59 133.89 131.59 129.07 128.88 128.73 126.25 125.66 116.98
77.31 77.06 76.81
55.62
43.81
39.01
20.75
Ph
O
Me
O
O
2b
220 210 200 190 180 170 160 150 140 130 120 110 100 90 80 70 60 50 40 30 20 10 0 -10
f1 (ppm)

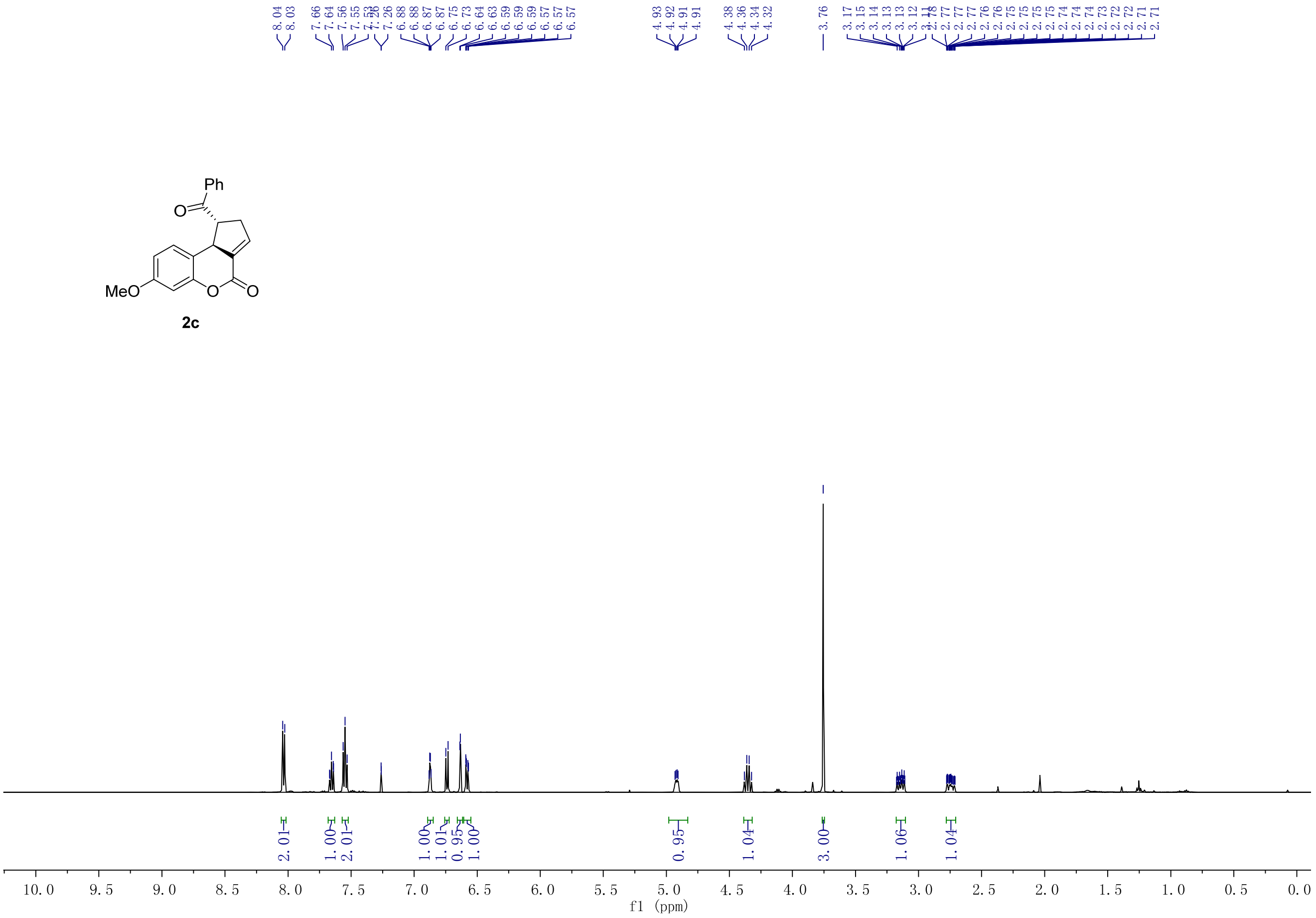
8.04
8.03
7.66
7.64
7.56
7.55
7.26
6.88
6.88
6.87
6.87
6.75
6.73
6.64
6.63
6.59
6.59
6.57
6.57
4.93
4.92
4.91
4.91
4.38
4.36
4.34
4.32
3.76
3.17
3.15
3.14
3.13
3.13
3.12
2.78
2.77
2.77
2.76
2.75
2.75
2.75
2.74
2.74
2.73
2.72
2.72
2.71
2.71
Ph
O
MeO
O
O
2c
2.01
1.00
2.01
1.00
1.01
0.95
1.00
0.95
1.04
3.00
1.06
1.04
10.0 9.5 9.0 8.5 8.0 7.5 7.0 6.5 6.0 5.5 5.0 4.5 4.0 3.5 3.0 2.5 2.0 1.5 1.0 0.5 0.0
f1 (ppm)

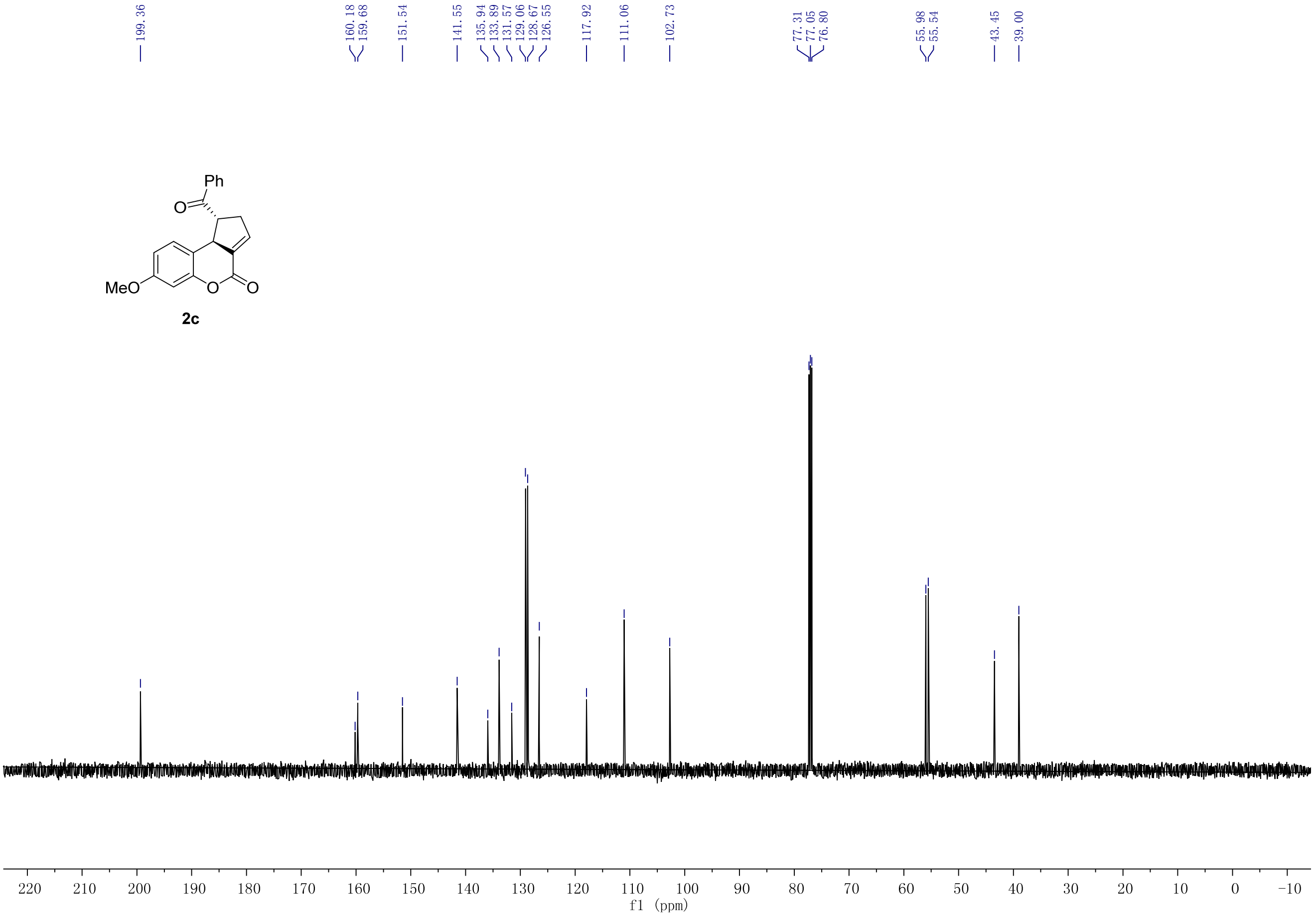
199.36
160.18
159.68
151.54
141.55
135.94
133.89
131.57
129.06
128.67
126.55
117.92
111.06
102.73
77.31
77.05
76.80
55.98
55.54
43.45
39.00
Ph
O
MeO
O
O
2c
220 210 200 190 180 170 160 150 140 130 120 110 100 90 80 70 60 50 40 30 20 10 0 -10
f1 (ppm)

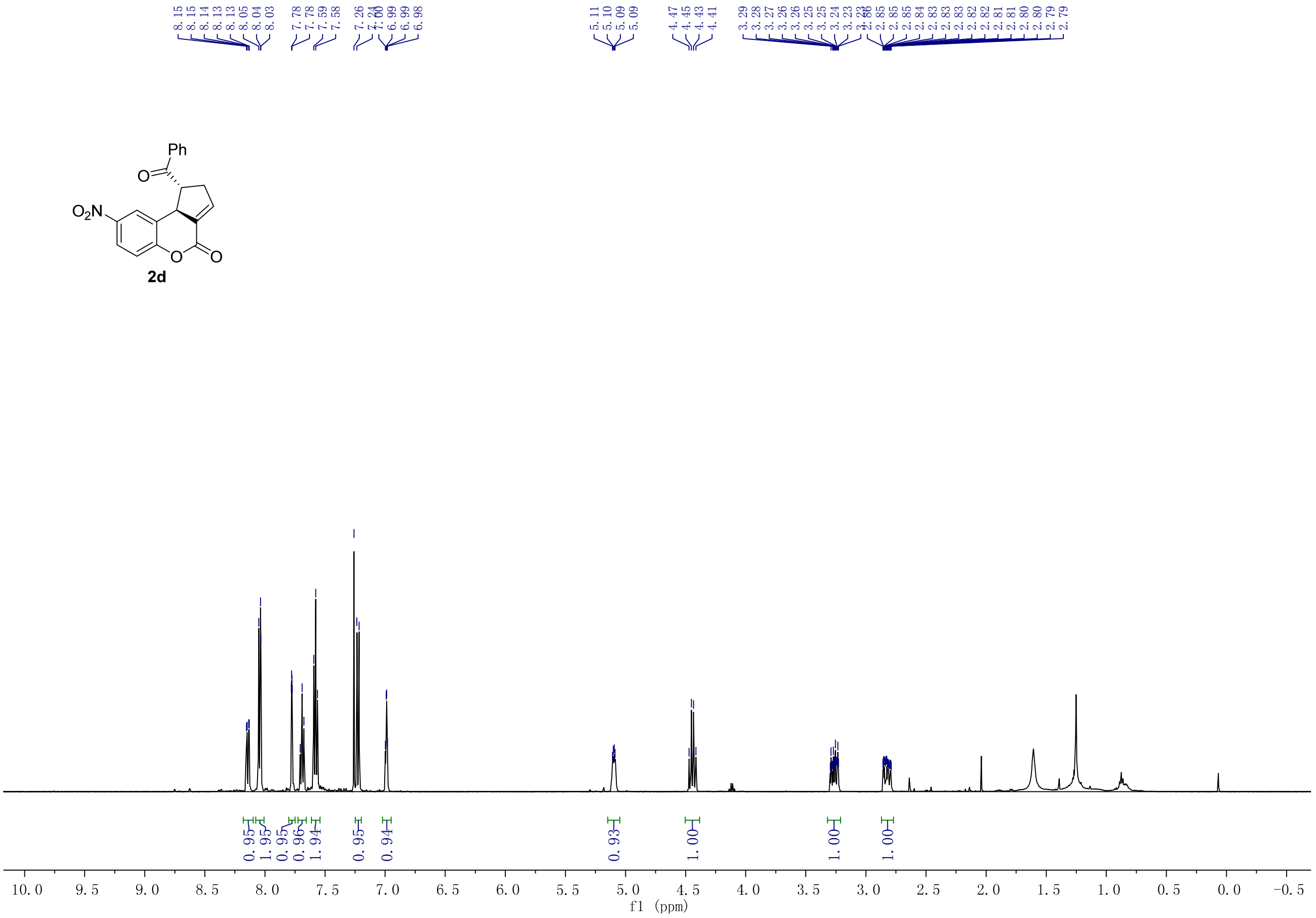

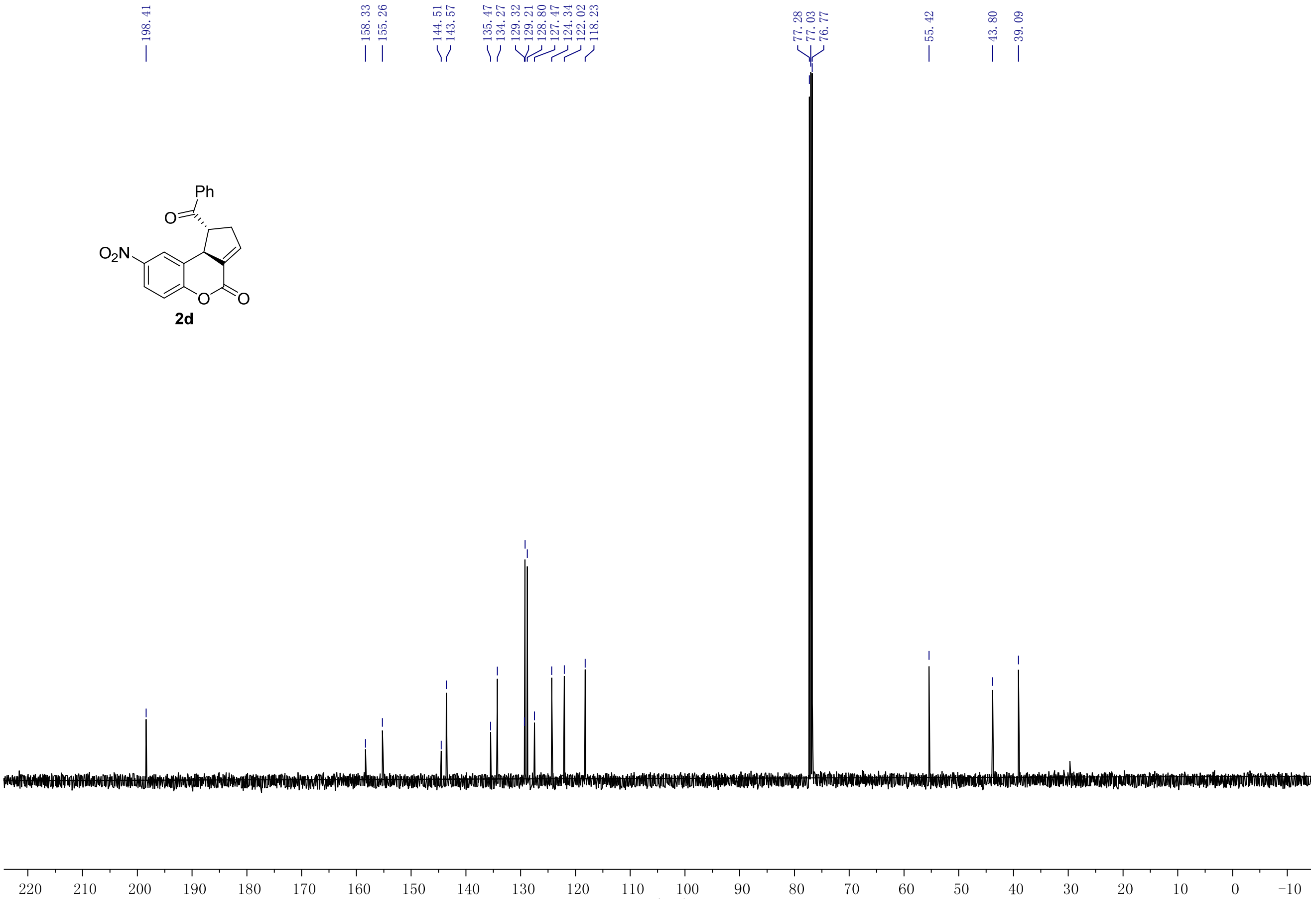

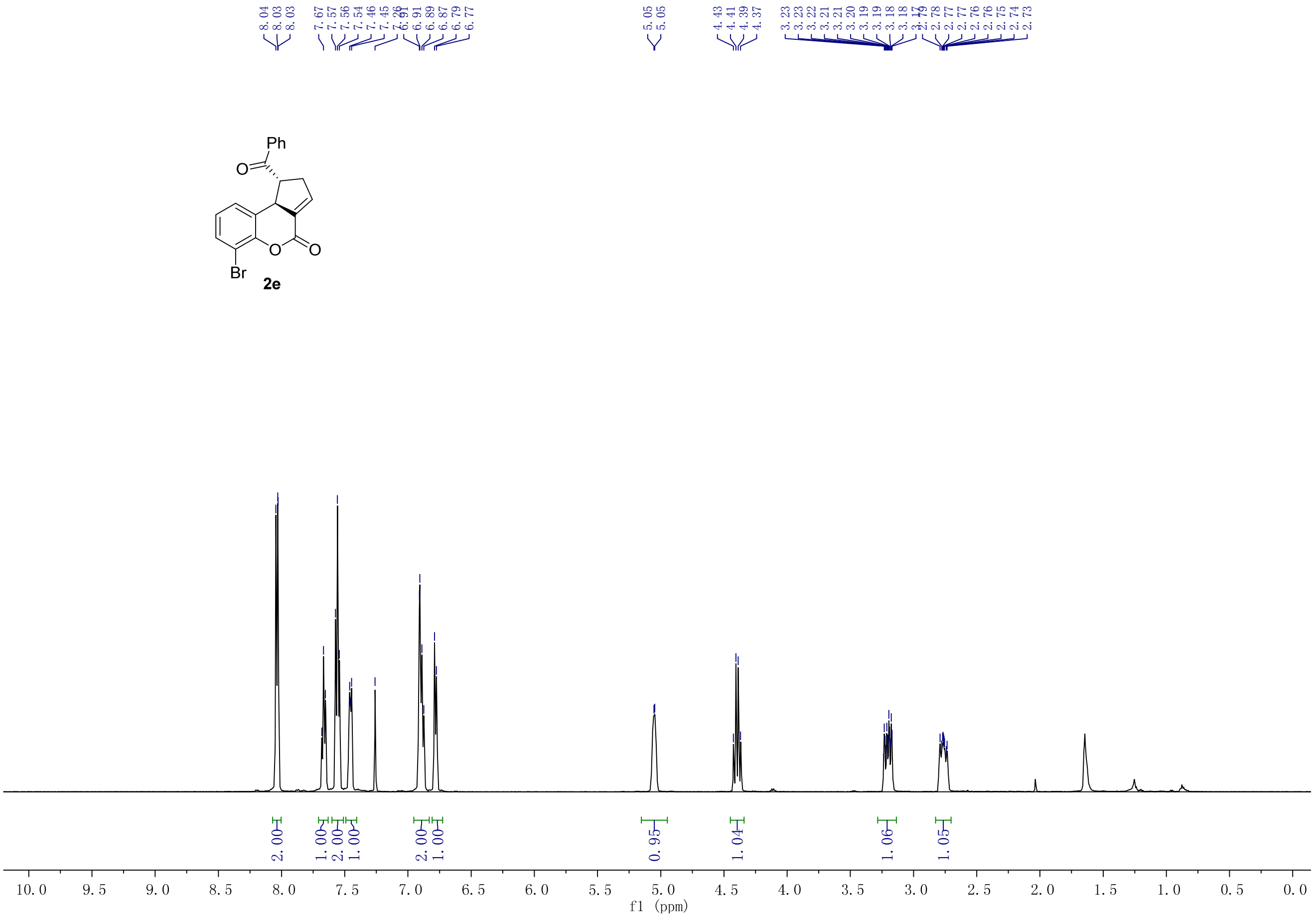

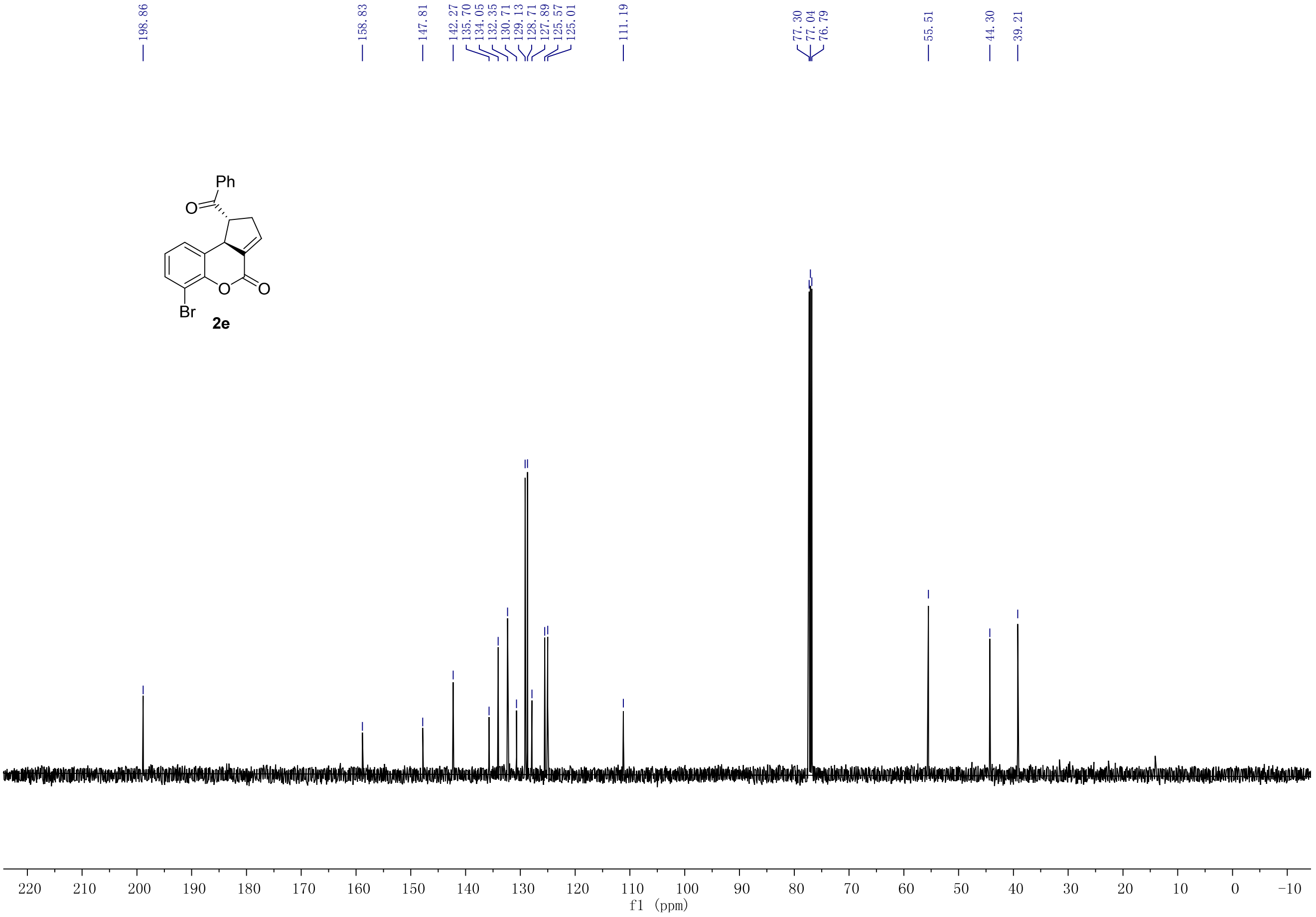

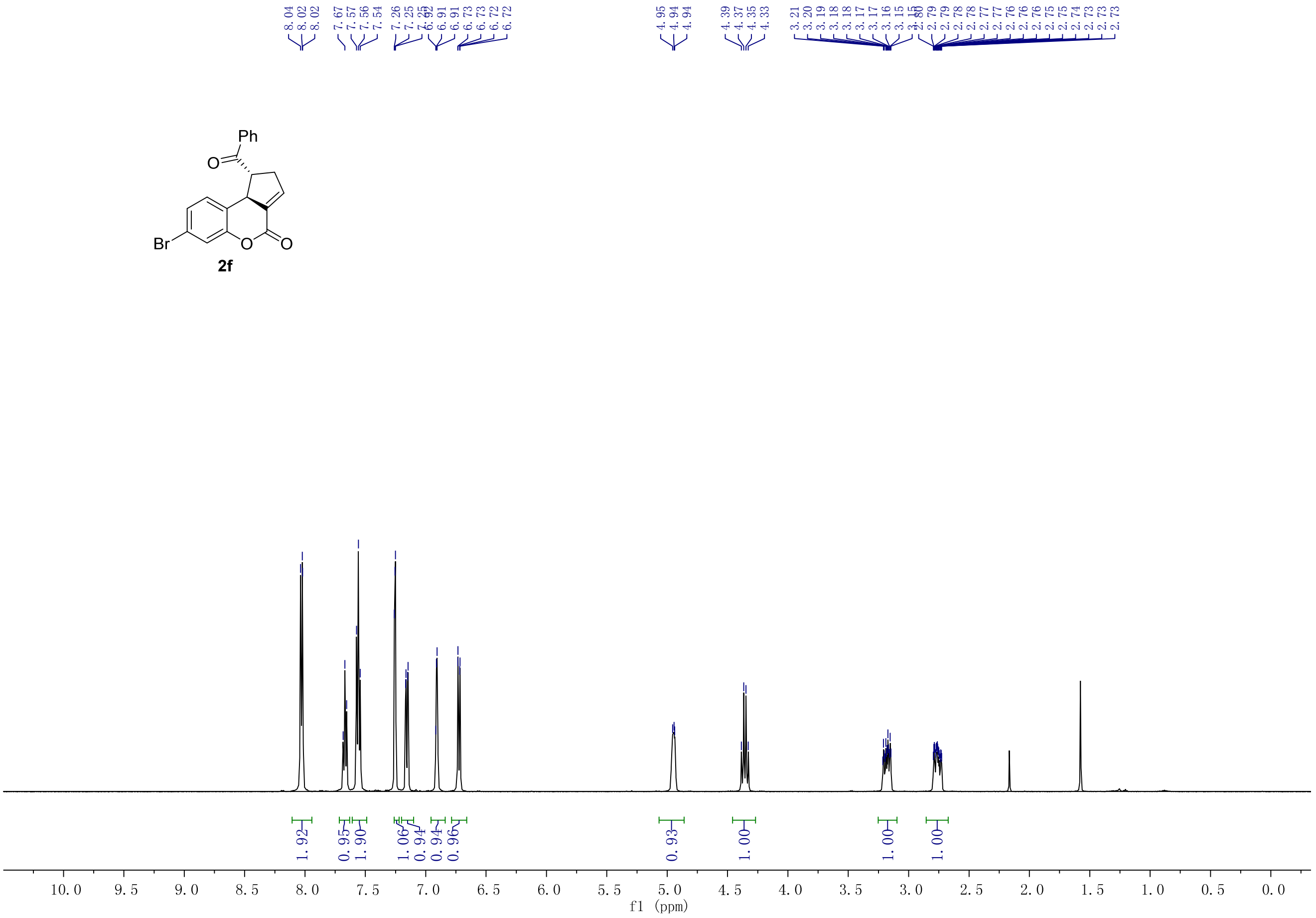
8.04
8.02
8.02
7.67
7.57
7.56
7.54
7.26
7.25
6.92
6.91
6.91
6.73
6.73
6.72
6.72
4.95
4.94
4.94
4.39
4.37
4.35
4.33
3.21
3.20
3.19
3.18
3.18
3.17
3.17
3.16
3.15
2.79
2.79
2.78
2.78
2.77
2.77
2.76
2.76
2.76
2.75
2.75
2.74
2.73
2.73
2.73
Ph
O
Br
O
O
2f
1.92
0.95
1.90
1.06
0.94
0.94
0.96
0.93
1.00
1.00
1.00
f1 (ppm)

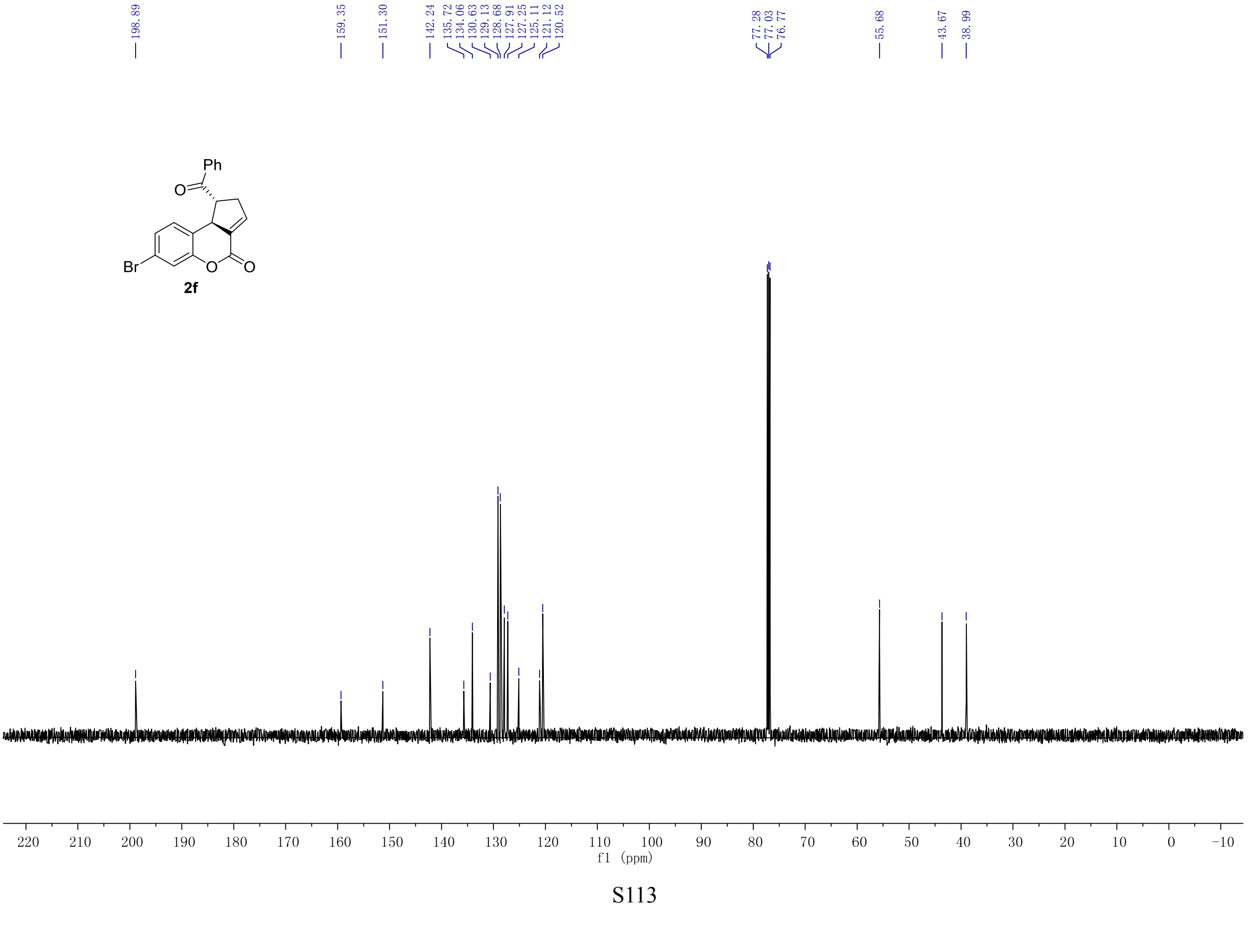
198.89
159.35
151.30
142.24
135.72
134.06
130.63
129.13
128.68
127.91
127.25
125.11
121.12
120.52
77.28
77.03
76.77
55.68
43.67
38.99
Ph
O
Br
O
O
2f
f1 (ppm)

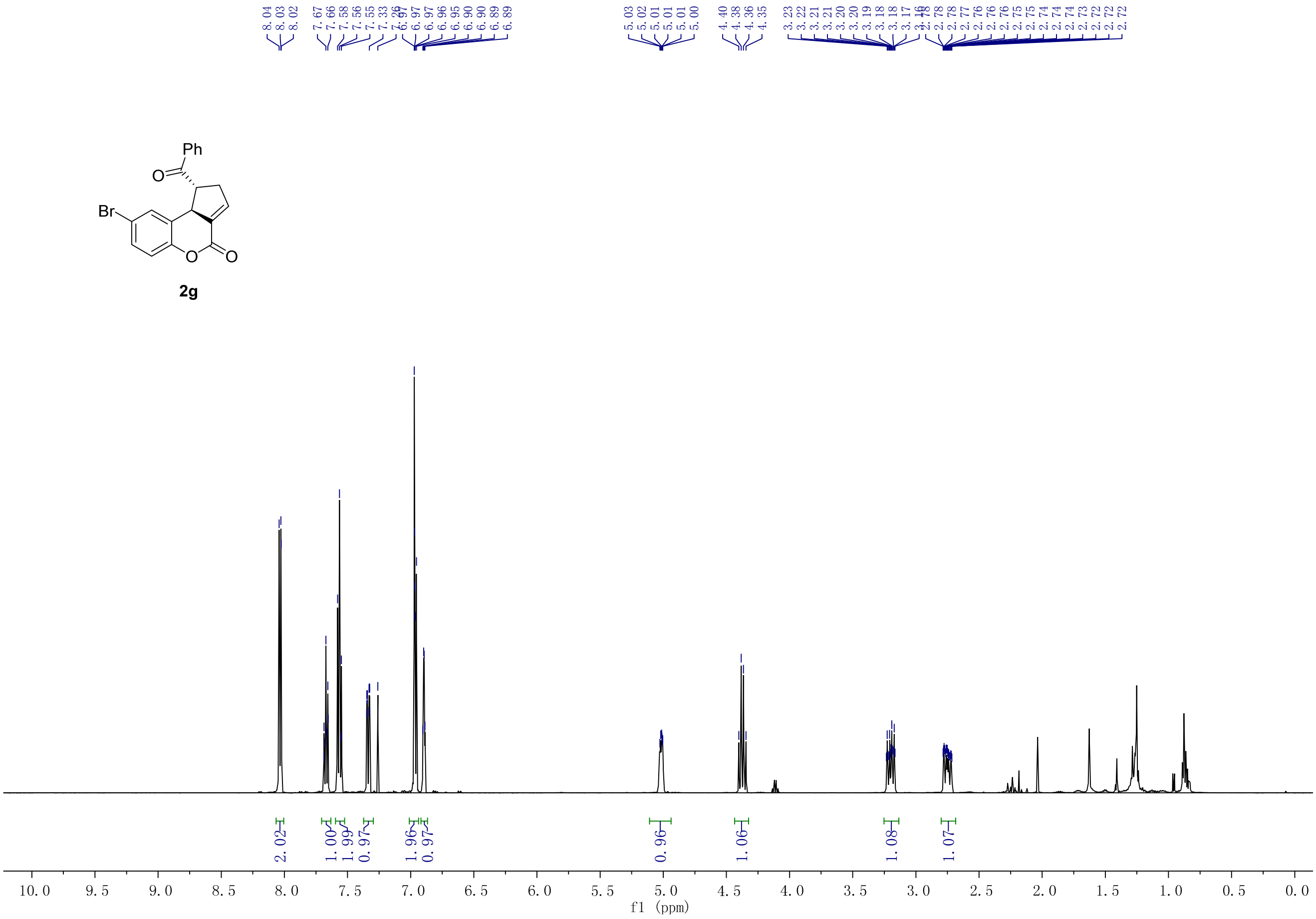
8.04 8.03 8.02 7.67 7.66 7.58 7.56 7.55 7.33 7.26 6.97 6.97 6.97 6.96 6.95 6.90 6.90 6.89 6.89
5.03 5.02 5.01 5.01 5.01 5.00 4.40 4.38 4.36 4.35
3.23 3.22 3.21 3.21 3.20 3.20 3.19 3.18 3.18 3.17 3.16 2.78 2.78 2.77 2.76 2.76 2.76 2.75 2.75 2.74 2.74 2.74 2.73 2.73 2.72 2.72 2.72
Ph
O
Br
O
O
2g
2.02 1.00 1.99 0.97 1.96 0.97 0.96 1.06 1.08 1.07
f1 (ppm)

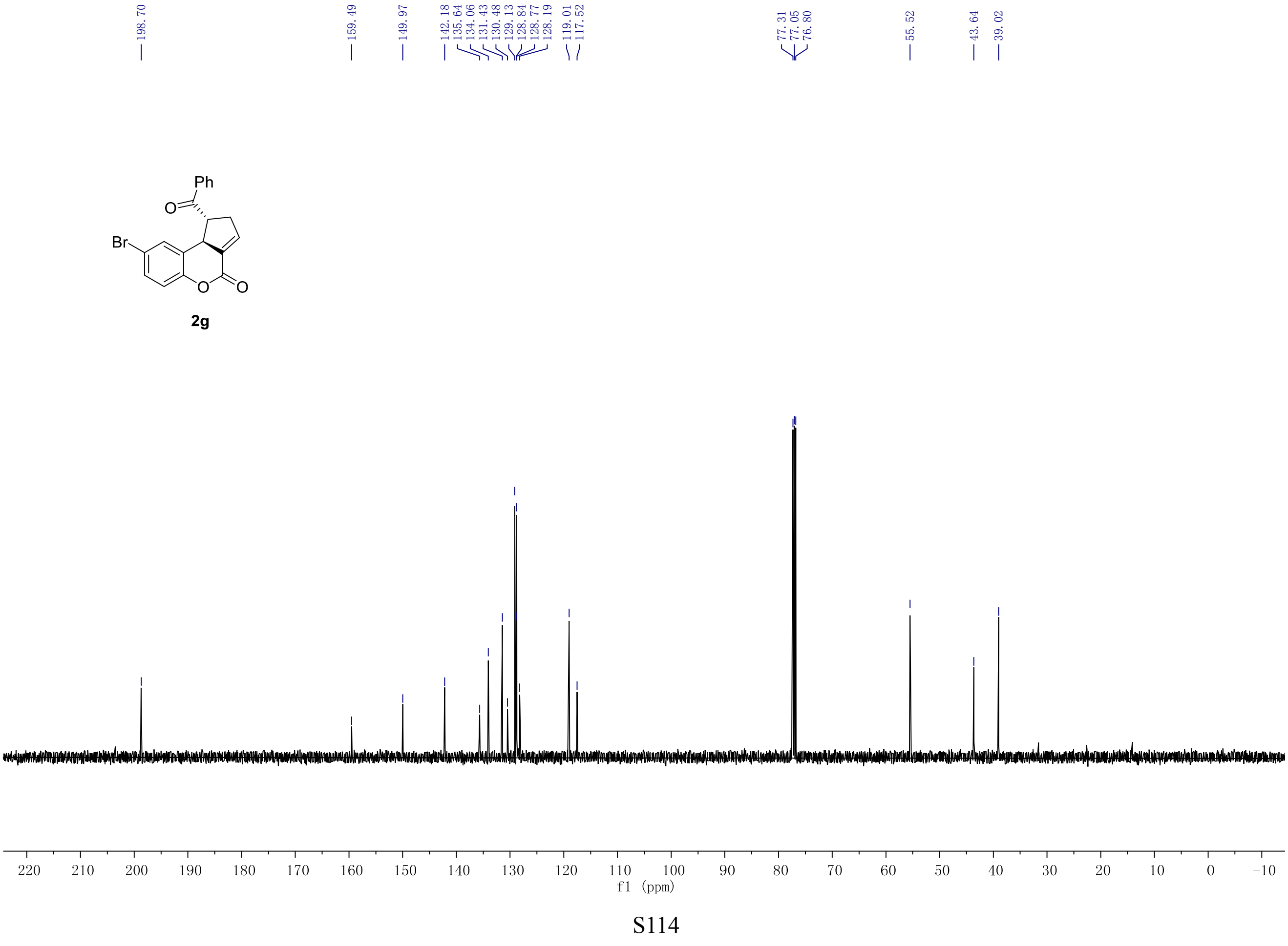
198.70 159.49 149.97 142.18 135.64 134.06 131.43 130.48 129.13 128.84 128.77 128.19 119.01 117.52
77.31 77.05 76.80 55.52 43.64 39.02
Ph
O
Br
O
O
2g
f1 (ppm)

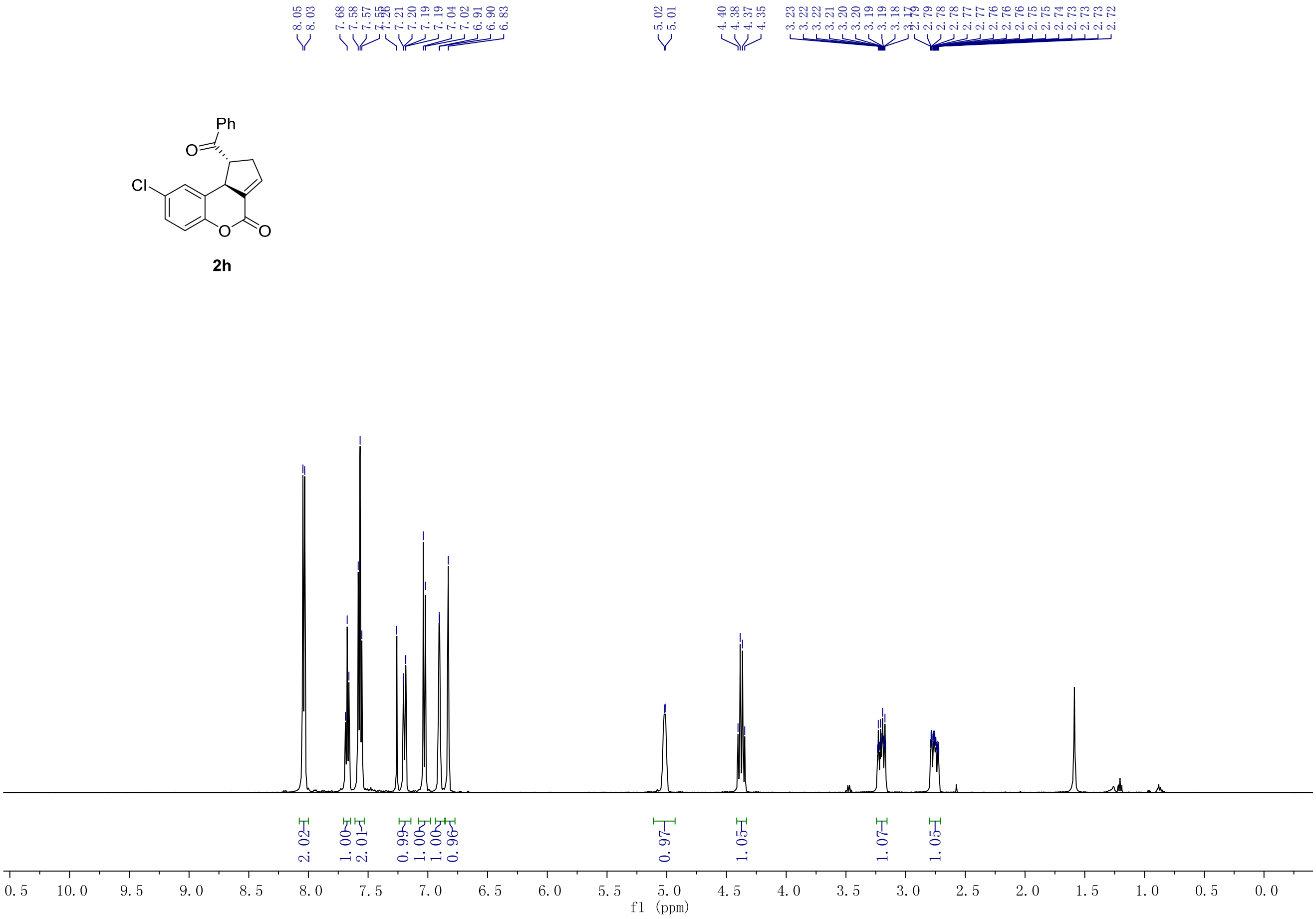

Ph
O
Cl
O
O
2h
8.05
8.03
7.68
7.58
7.57
7.21
7.20
7.19
7.19
7.04
7.02
6.91
6.90
6.83
5.02
5.01
4.40
4.38
4.37
4.35
2.02
1.00
2.01
0.99
1.00
1.00
0.96
0.97
1.05
1.07
1.05
f1 (ppm)

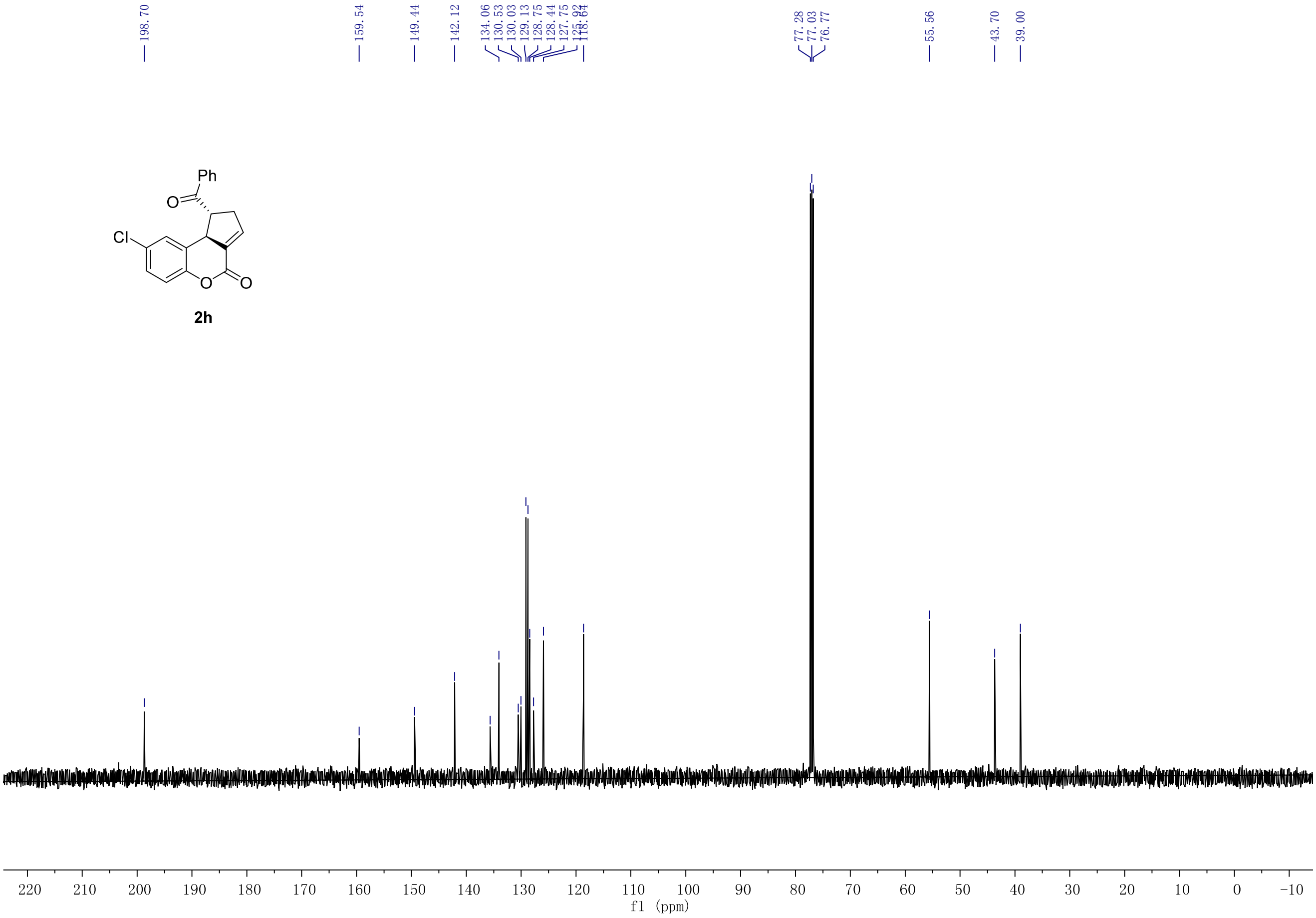

Ph
O
Cl
O
O
2h
198.70
159.54
149.44
142.12
134.06
130.53
130.03
129.13
128.75
128.44
127.75
77.28
77.03
76.77
55.56
43.70
39.00
f1 (ppm)

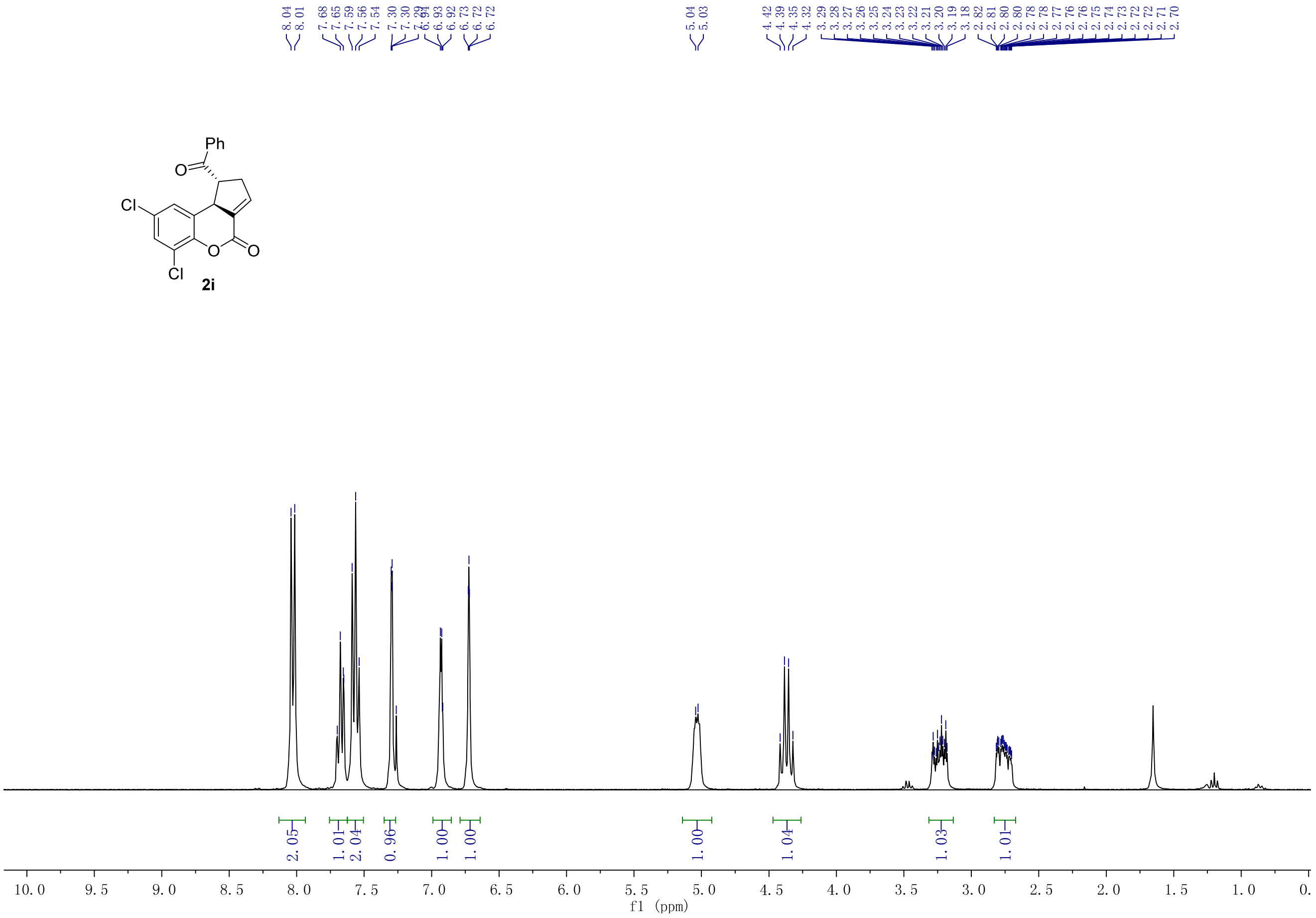

8.04
8.01
7.68
7.65
7.59
7.56
7.54
7.30
7.30
6.93
6.92
6.73
6.72
6.72
5.04
5.03
4.42
4.39
4.35
4.32
Ph
O
Cl
Cl
O
O
2i
2.05
1.01
2.04
0.96
1.00
1.00
1.00
1.04
1.03
1.01
f1 (ppm)

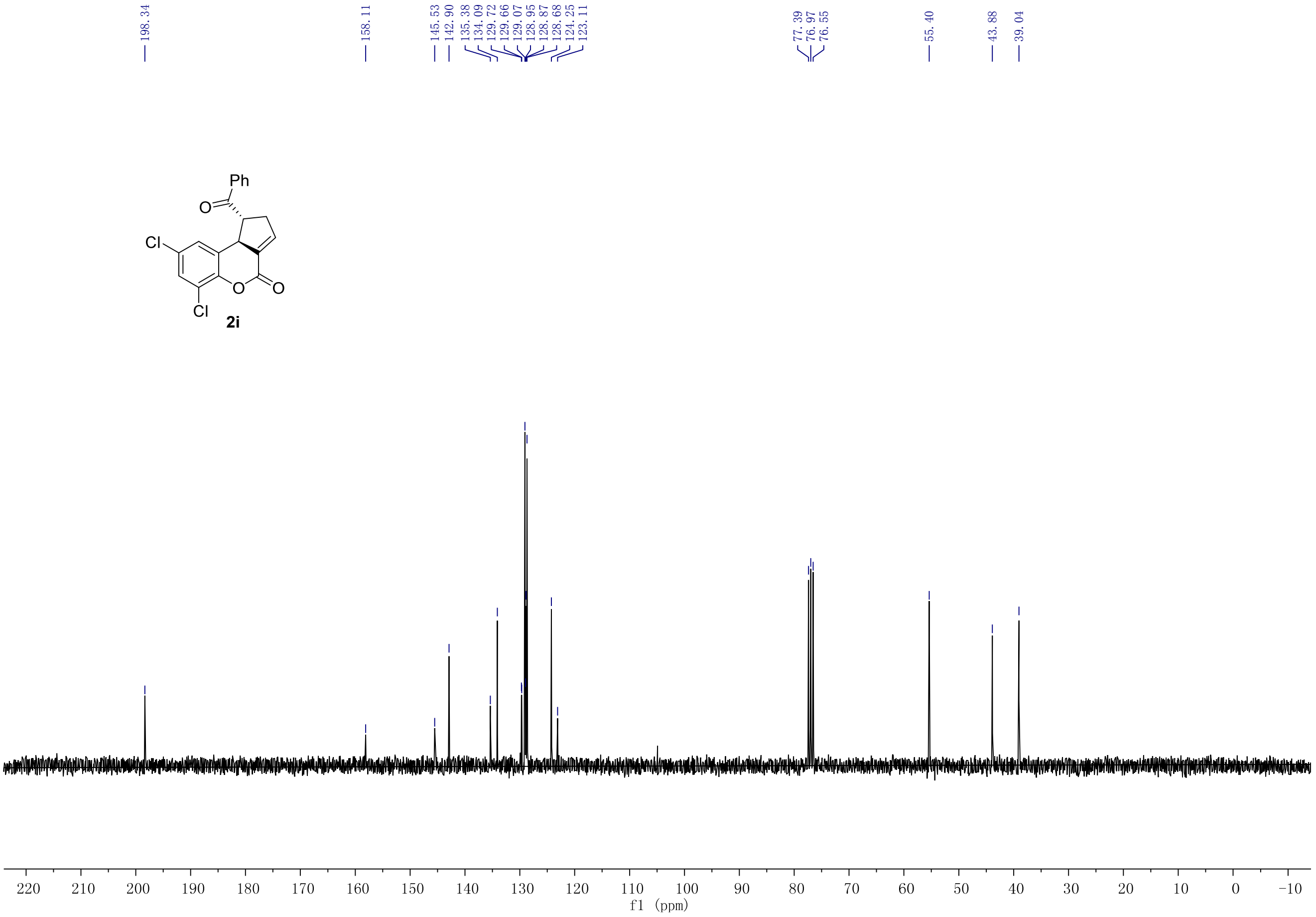

198.34
158.11
145.53
142.90
135.38
134.09
129.72
129.66
129.07
128.95
128.87
128.68
124.25
123.11
77.39
76.97
76.55
55.40
43.88
39.04
Ph
O
Cl
Cl
O
O
2i
f1 (ppm)

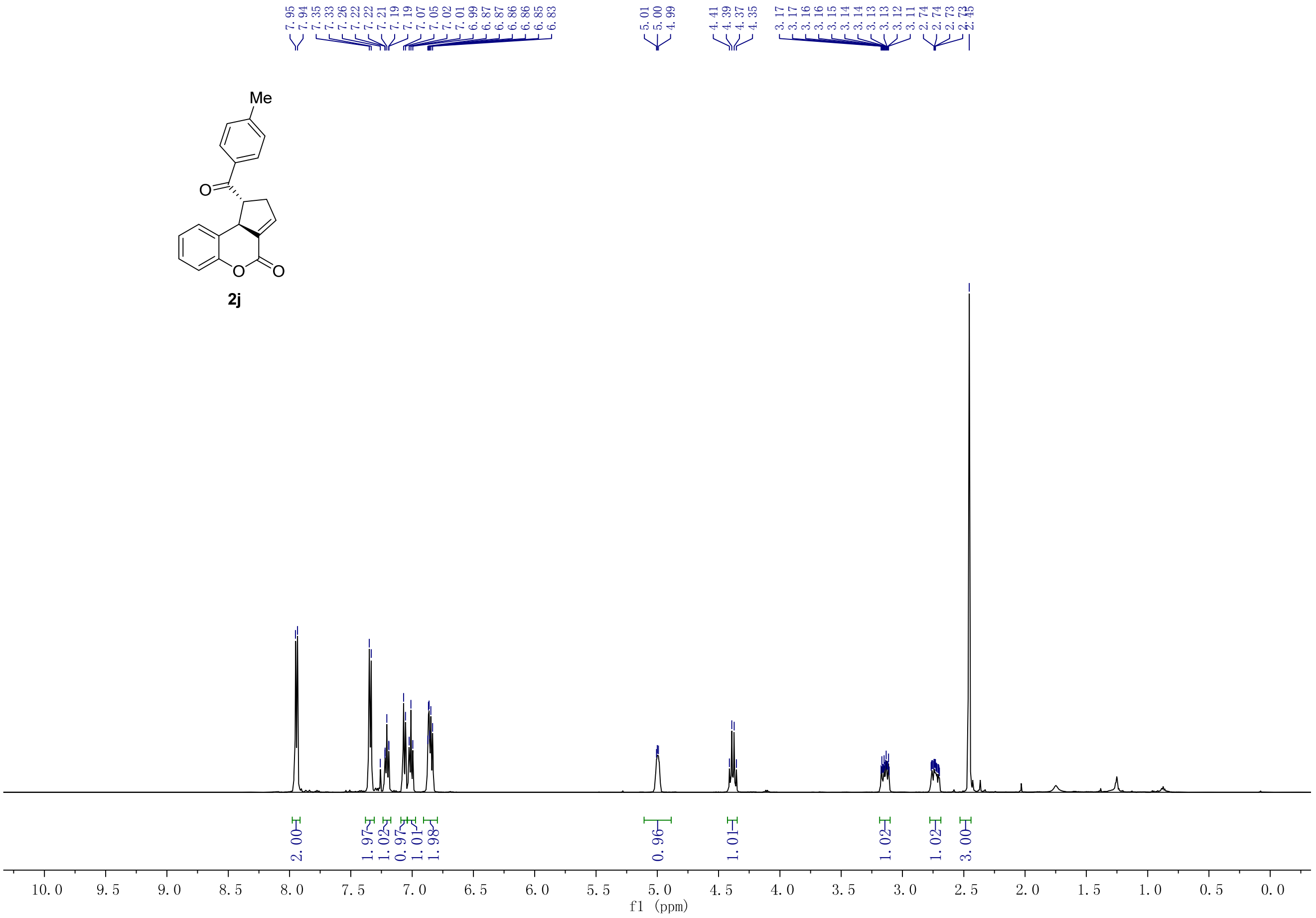
7.95 7.94 7.35 7.33 7.36 7.22 7.22 7.21 7.19 7.07 7.05 7.02 7.01 6.99 6.87 6.87 6.86 6.86 6.85 6.83
5.01 5.00 4.99
4.41 4.39 4.37 4.35
3.17 3.17 3.16 3.16 3.15 3.14 3.14 3.13 3.13 3.12 3.11 2.74 2.74 2.73 2.73 2.45
Me
O
O
O
2j
2.00 1.97 1.02 0.97 1.01 1.98 0.96 1.01 1.02 1.02 3.00
f1 (ppm)

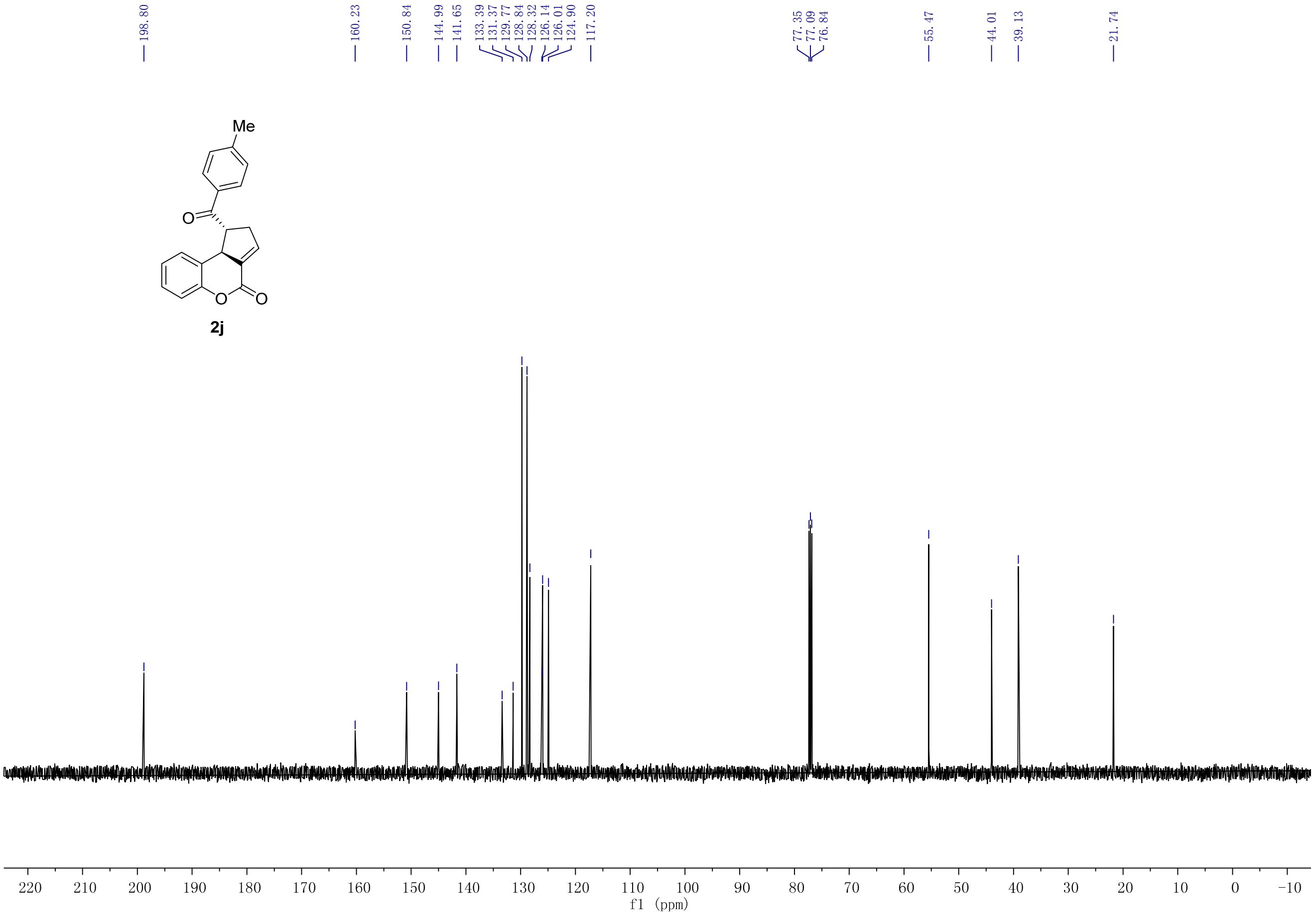
198.80
160.23
150.84
144.99
141.65
133.39 131.37 129.77 128.84 128.32 126.14 126.01 124.90
117.20
77.35 77.09 76.84
55.47
44.01
39.13
21.74
Me
O
O
O
2j
f1 (ppm)

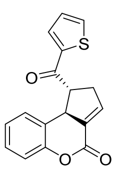

2k

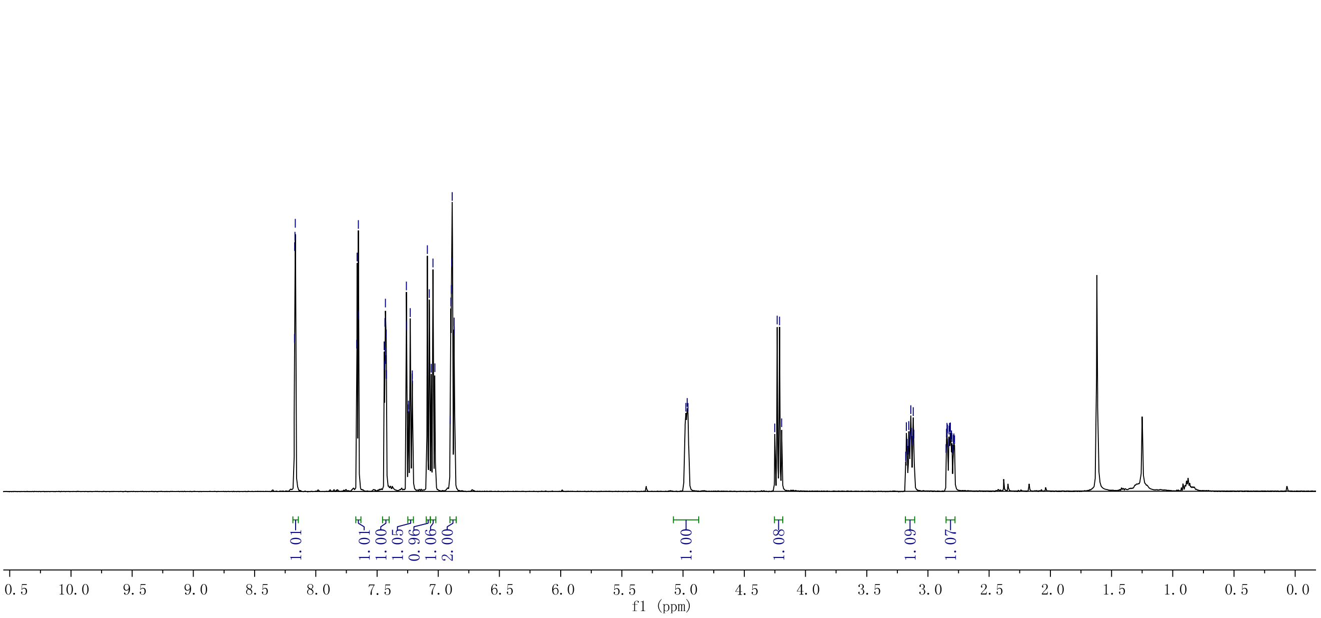

1.01
1.01
1.00
1.05
0.96
1.06
2.00
1.00
1.08
1.09
1.07
f1 (ppm)

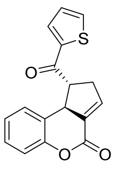

2k

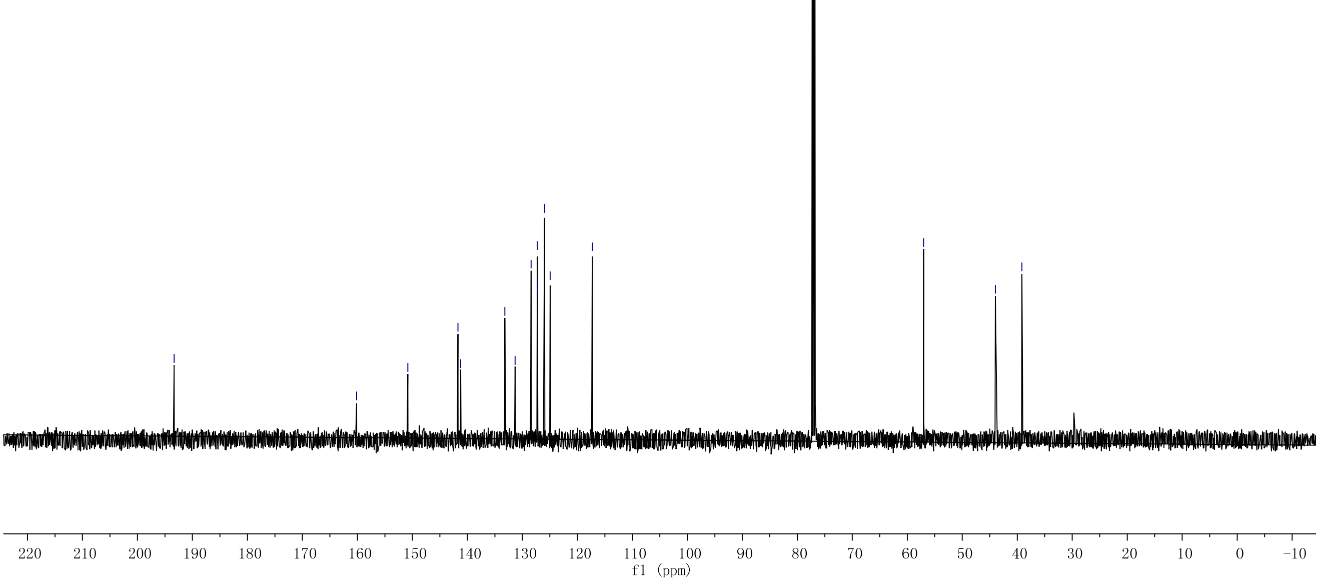

193.34
160.14
150.83
141.70
141.21
133.18
131.33
128.41
127.27
127.21
125.97
124.93
117.28
77.29
77.04
76.78
57.01
43.98
39.15
f1 (ppm)

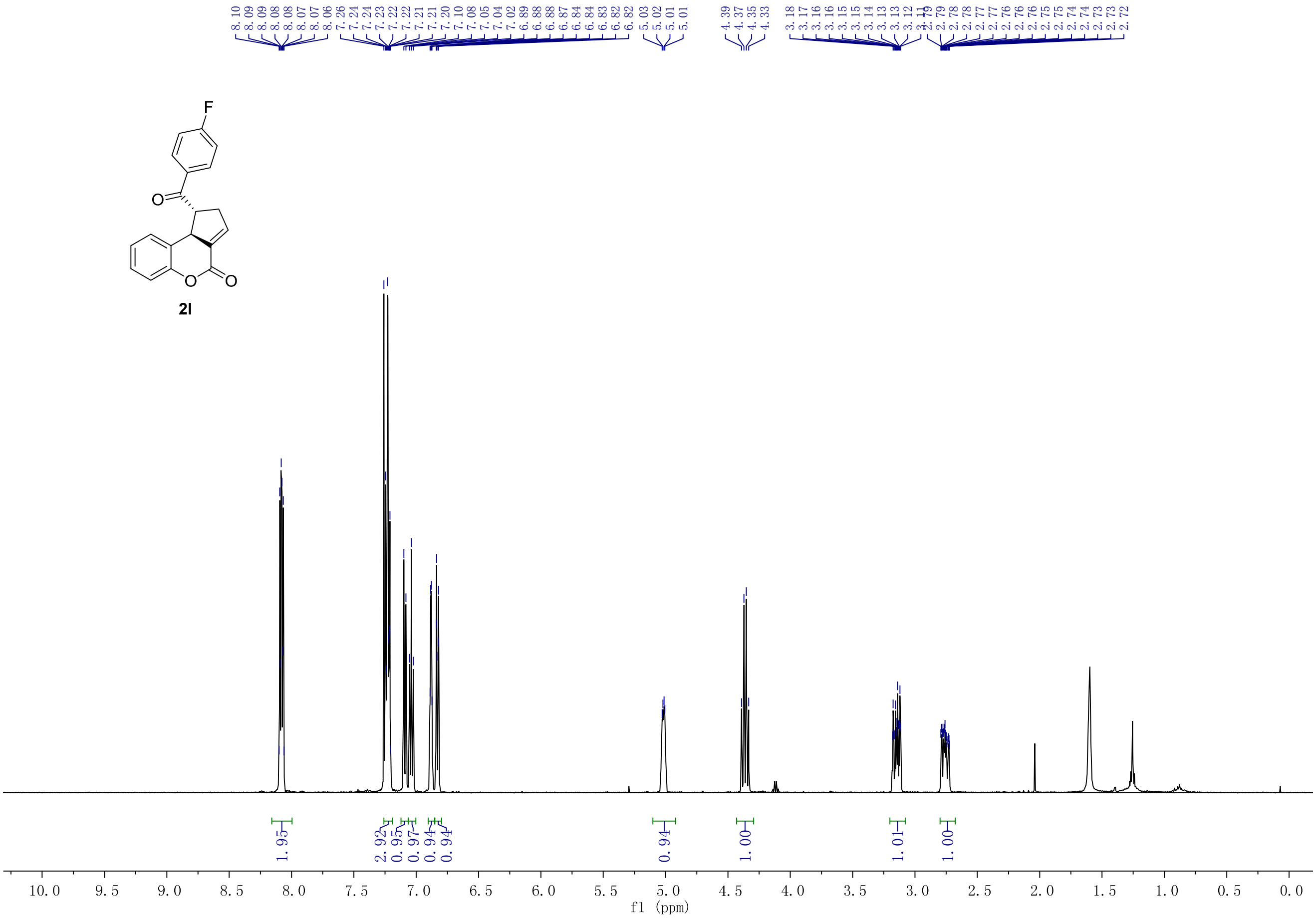
8.10 8.09 8.09 8.08 8.08 8.07 8.06 7.26 7.24 7.24 7.23 7.22 7.22 7.21 7.21 7.20 7.10 7.08 7.05 7.04 7.02 6.89 6.88 6.87 6.84 6.84 6.83 6.82 6.82 5.03 5.02 5.01 5.01 4.39 4.37 4.35 4.33 3.18 3.17 3.16 3.16 3.15 3.15 3.14 3.13 3.13 3.12 2.79 2.78 2.78 2.77 2.77 2.76 2.76 2.75 2.75 2.74 2.74 2.73 2.73 2.72
F
O
O
O
2l
1.95
2.92
0.95
0.97
0.94
0.94
0.94
1.00
1.01
1.00
10.0 9.5 9.0 8.5 8.0 7.5 7.0 6.5 6.0 5.5 5.0 4.5 4.0 3.5 3.0 2.5 2.0 1.5 1.0 0.5 0.0
f1 (ppm)

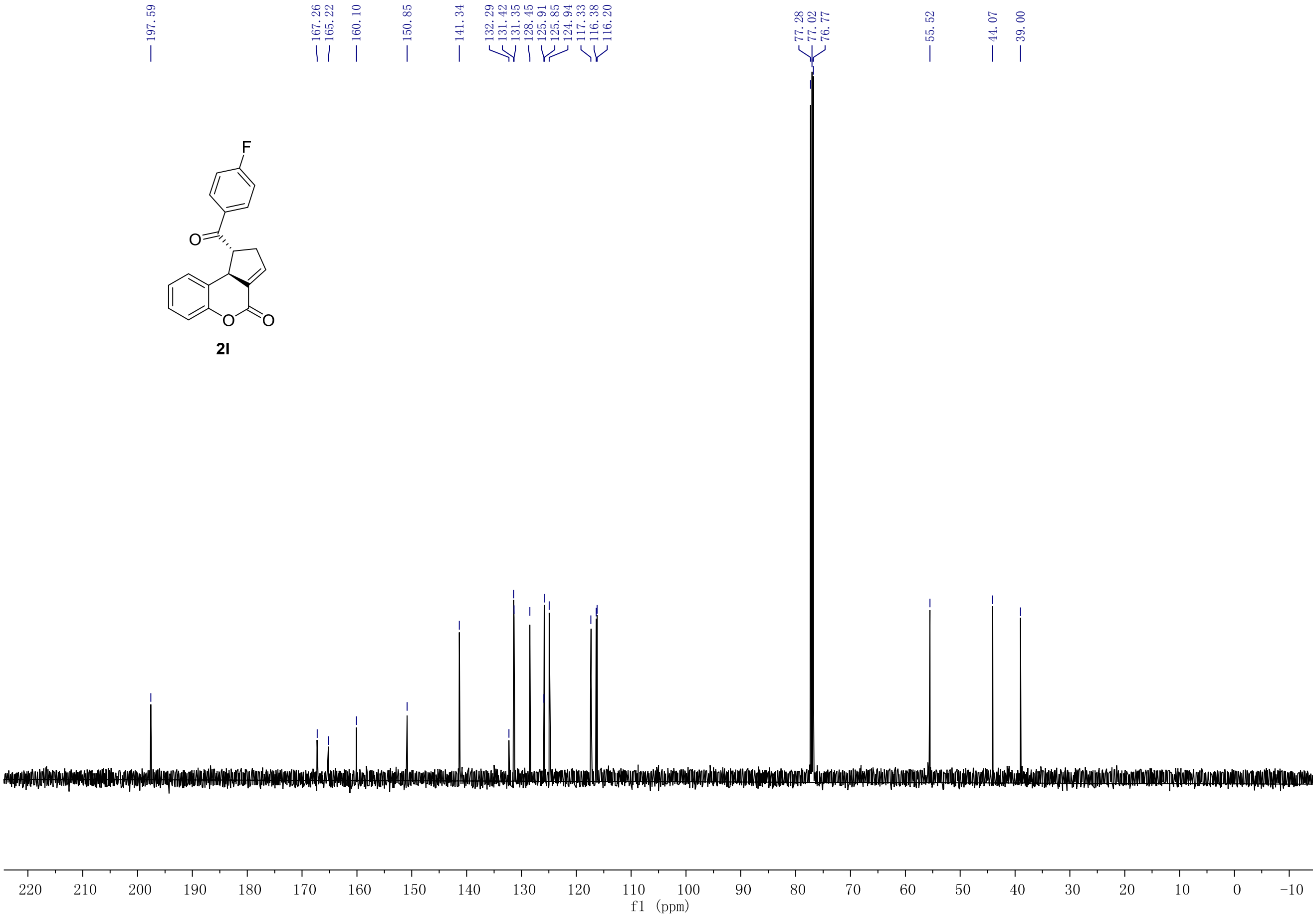
197.59
167.26 165.22
160.10
150.85
141.34
132.29 131.42 131.35 128.45 125.91 125.85 124.94 117.33 116.38 116.20
77.28 77.02 76.77
55.52
44.07
39.00
F
O
O
O
2l
220 210 200 190 180 170 160 150 140 130 120 110 100 90 80 70 60 50 40 30 20 10 0 -10
f1 (ppm)

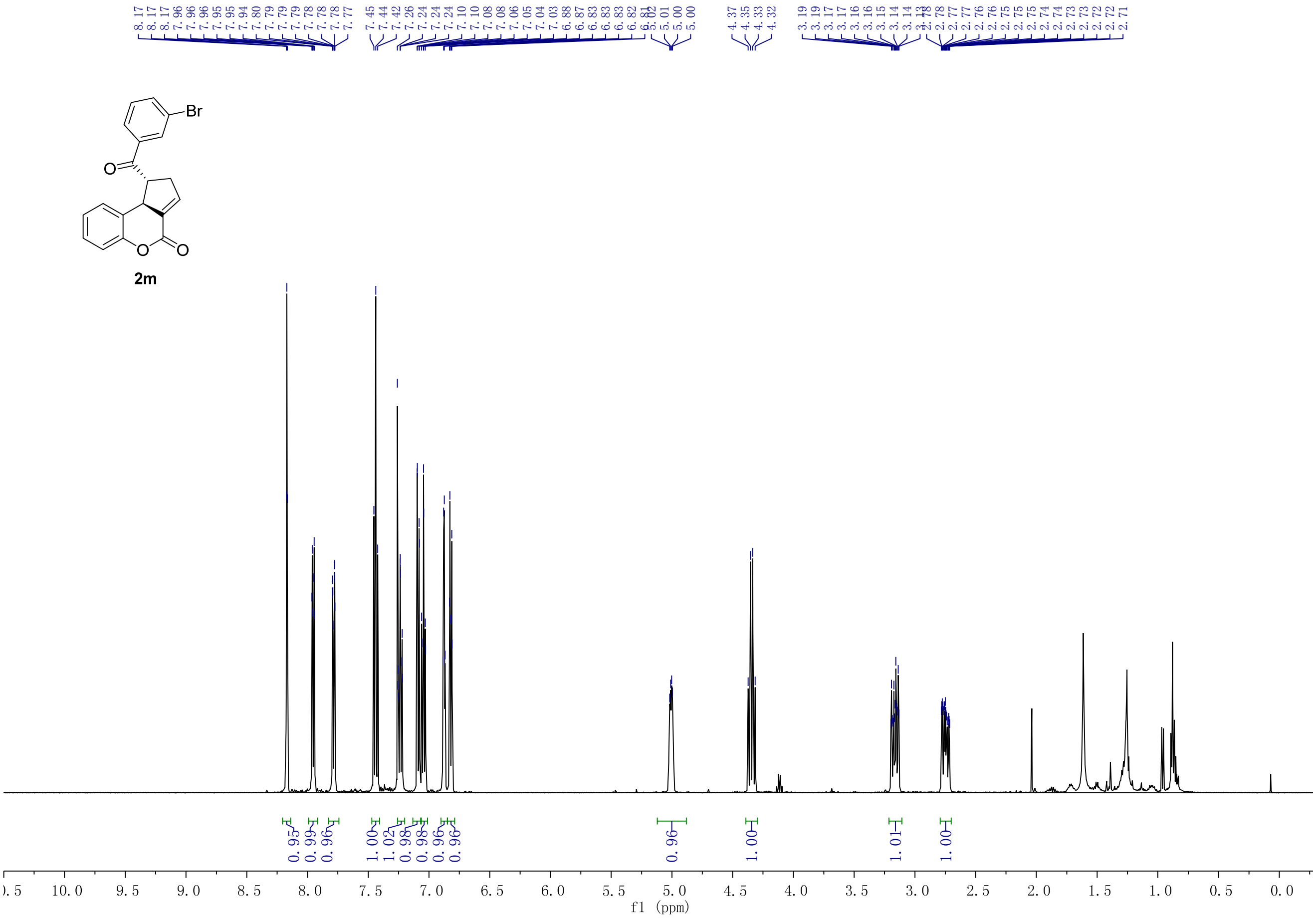

2m

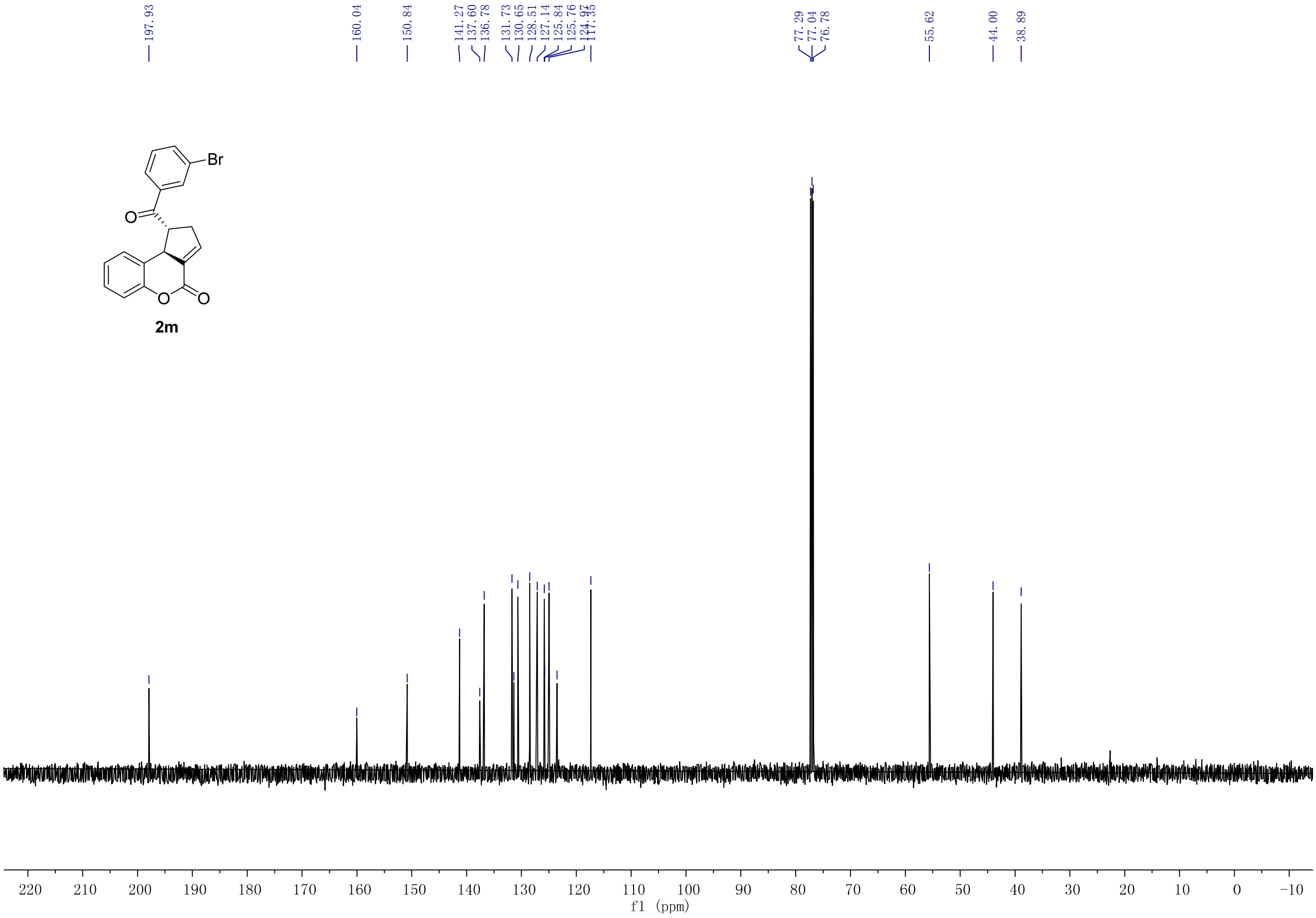

2m

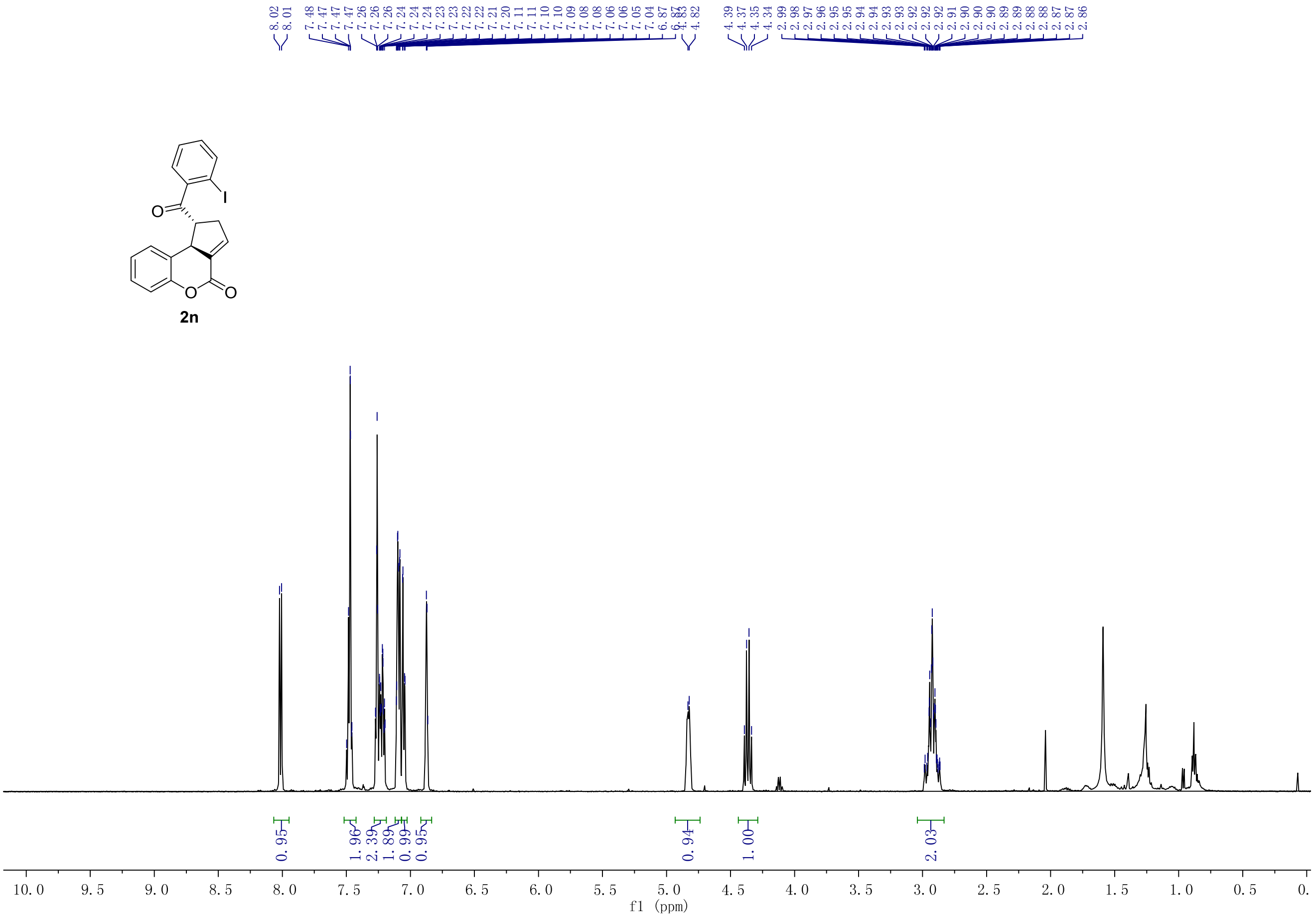
8.02 8.01 7.48 7.47 7.47 7.47 7.26 7.26 7.26 7.24 7.24 7.24 7.24 7.23 7.23 7.22 7.22 7.21 7.20 7.11 7.11 7.10 7.09 7.08 7.08 7.06 7.06 7.05 7.04 6.87 4.83 4.82 4.39 4.37 4.35 4.34 2.99 2.98 2.97 2.96 2.95 2.95 2.94 2.94 2.93 2.93 2.92 2.92 2.92 2.92 2.91 2.90 2.90 2.89 2.89 2.88 2.88 2.87 2.87 2.86
I
O
O
O
2n
0.95 1.96 2.39 1.89 0.99 0.95 0.94 1.00 2.03
10.0 9.5 9.0 8.5 8.0 7.5 7.0 6.5 6.0 5.5 5.0 4.5 4.0 3.5 3.0 2.5 2.0 1.5 1.0 0.5 0.
f1 (ppm)

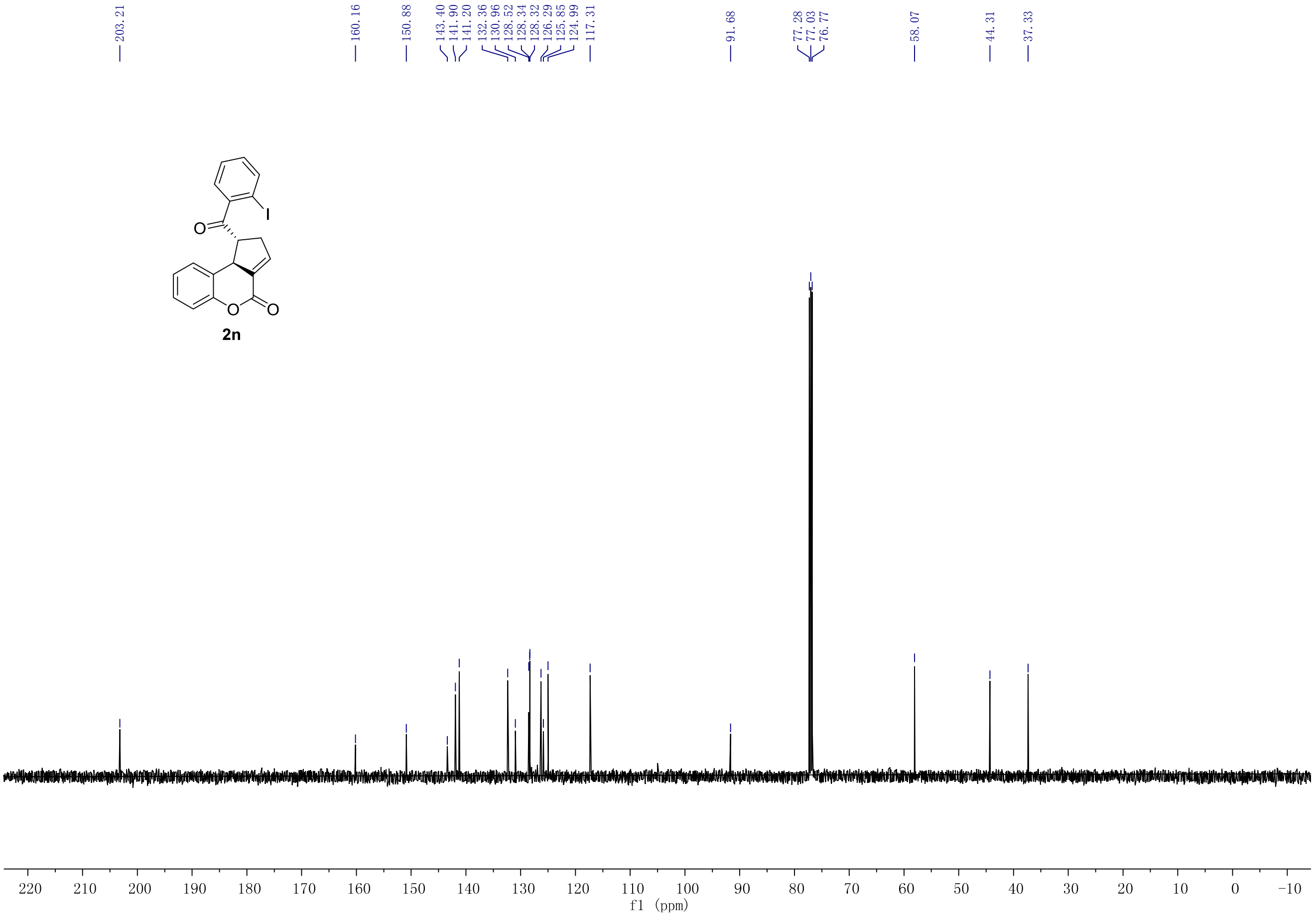
203.21 160.16 150.88 143.40 141.90 141.20 132.36 130.96 128.52 128.34 128.32 126.29 125.85 124.99 117.31 91.68 77.28 77.03 76.77 58.07 44.31 37.33
I
O
O
O
2n
220 210 200 190 180 170 160 150 140 130 120 110 100 90 80 70 60 50 40 30 20 10 0 −10
f1 (ppm)

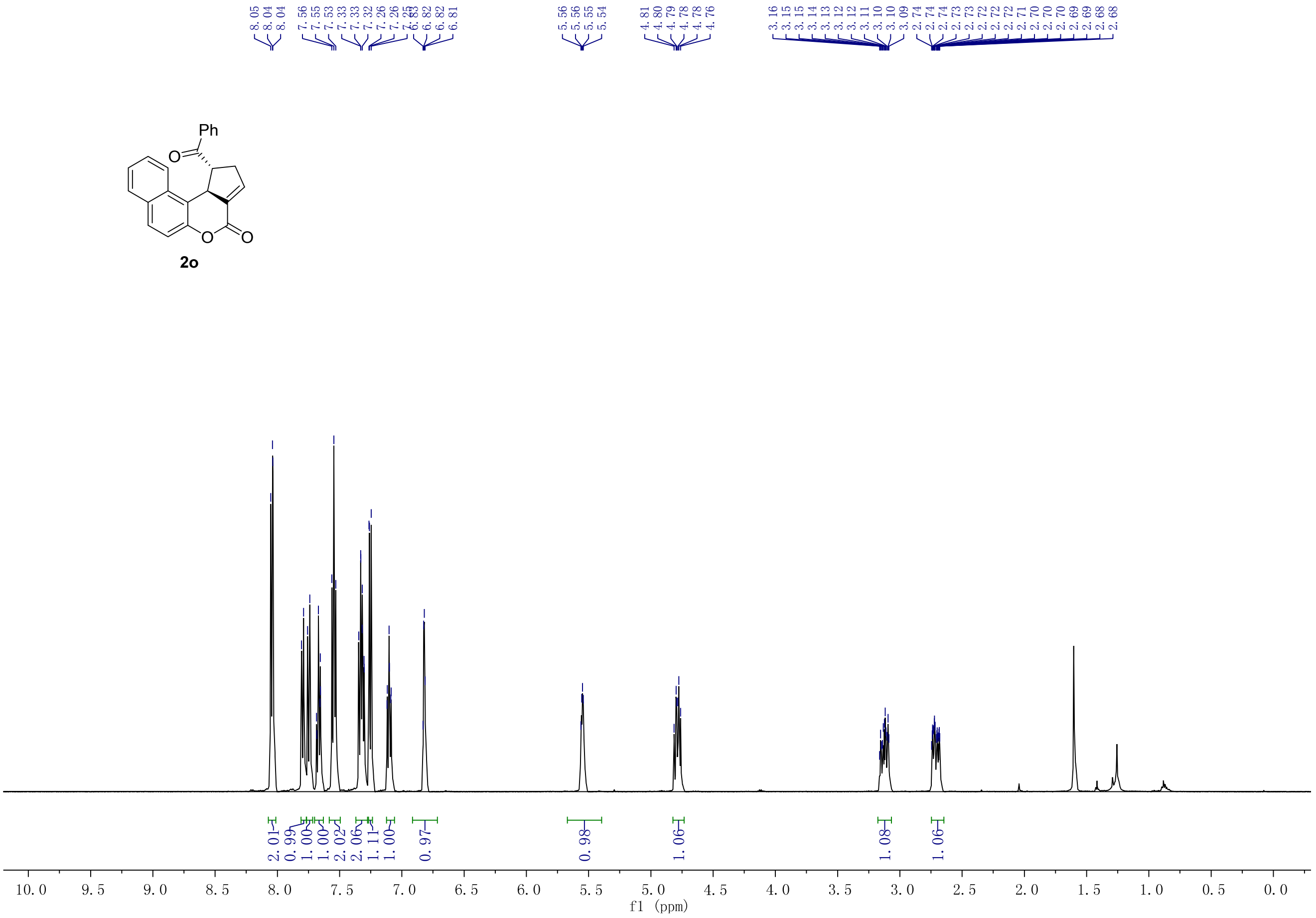
8.05
8.04
8.04
7.56
7.55
7.53
7.33
7.33
7.32
7.26
7.26
7.25
6.83
6.82
6.82
6.81
5.56
5.56
5.55
5.54
4.81
4.80
4.79
4.78
4.78
4.76
3.16
3.15
3.15
3.14
3.13
3.12
3.12
3.11
3.10
3.10
3.09
2.74
2.74
2.74
2.73
2.73
2.72
2.72
2.72
2.71
2.70
2.70
2.69
2.69
2.68
2.68
Ph
O
O
O
2o
2.01
0.99
1.00
1.00
2.02
2.06
1.11
1.00
0.97
0.98
1.06
1.08
1.06
10.0
9.5
9.0
8.5
8.0
7.5
7.0
6.5
6.0
5.5
5.0
4.5
4.0
3.5
3.0
2.5
2.0
1.5
1.0
0.5
0.0
f1 (ppm)

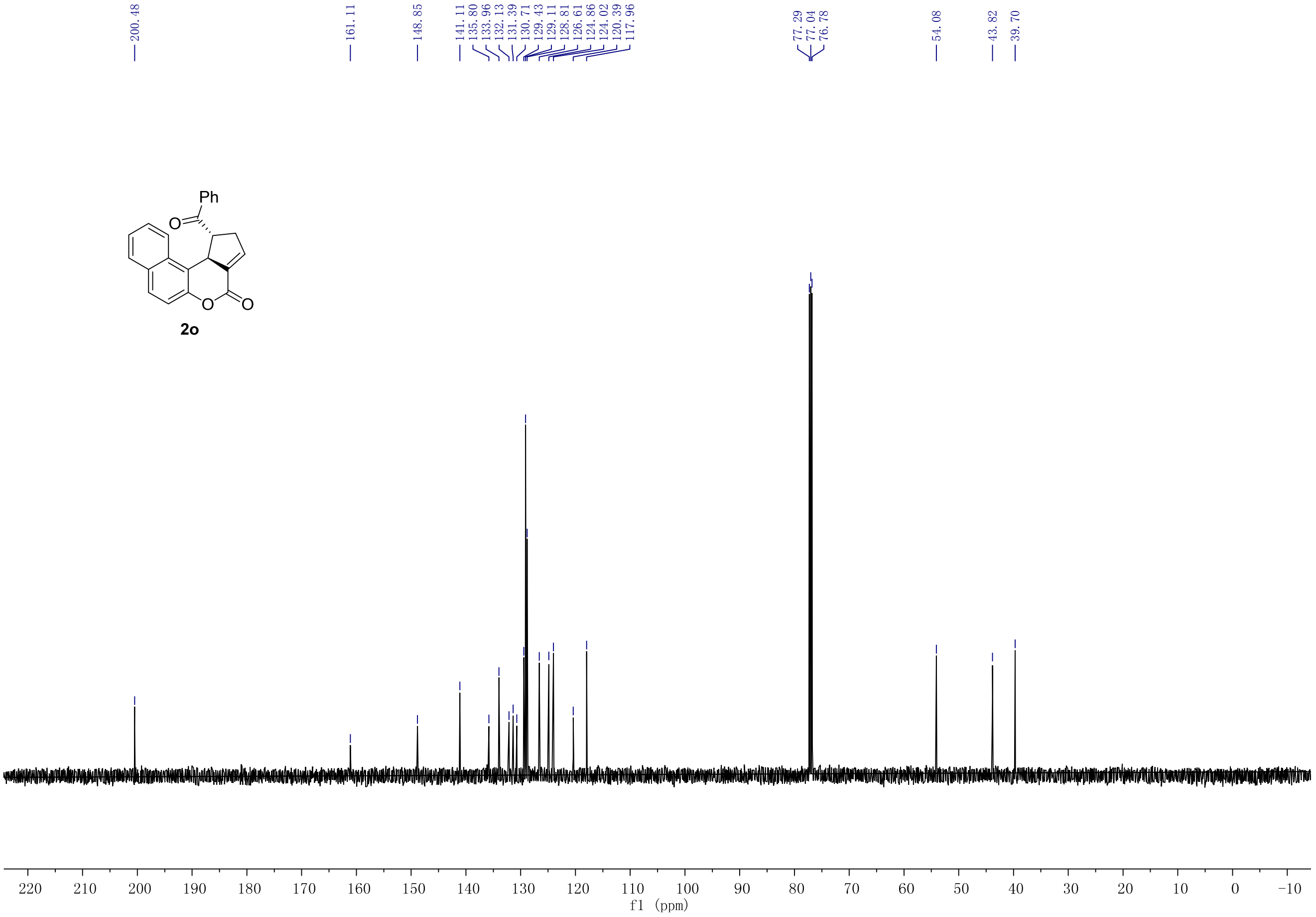
200.48
161.11
148.85
141.11
135.80
133.96
132.13
131.39
130.71
129.43
129.11
128.81
126.61
124.86
124.02
120.39
117.96
77.29
77.04
76.78
54.08
43.82
39.70
Ph
O
O
O
2o
220
210
200
190
180
170
160
150
140
130
120
110
100
90
80
70
60
50
40
30
20
10
0
-10
f1 (ppm)

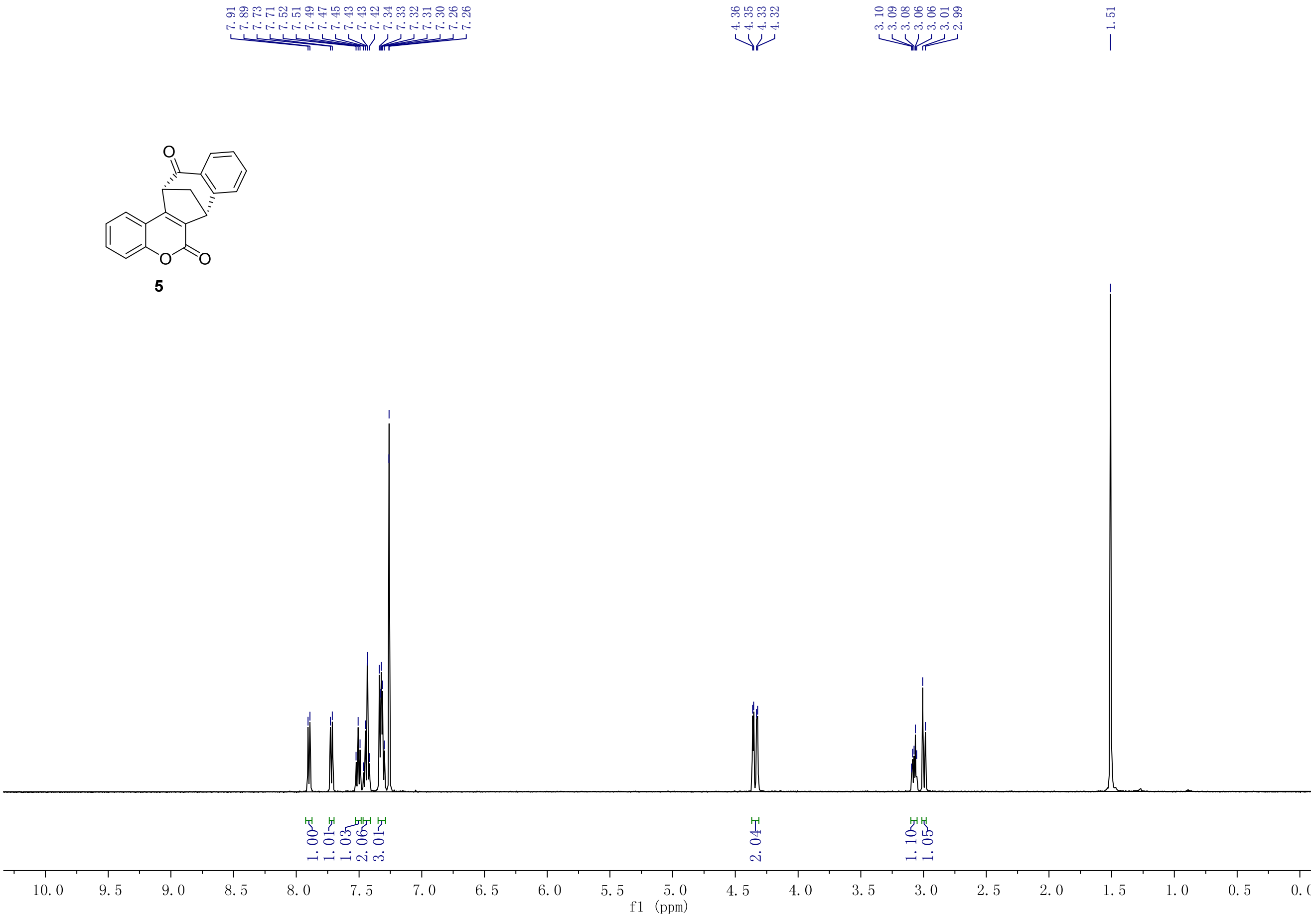
7.91
7.89
7.73
7.71
7.52
7.51
7.49
7.47
7.45
7.43
7.43
7.43
7.42
7.34
7.33
7.32
7.31
7.30
7.26
7.26
4.36
4.35
4.33
4.32
3.10
3.09
3.08
3.06
3.06
3.01
2.99
1.51
5
1.00
1.01
1.03
2.06
3.01
2.04
1.10
1.05
f1 (ppm)

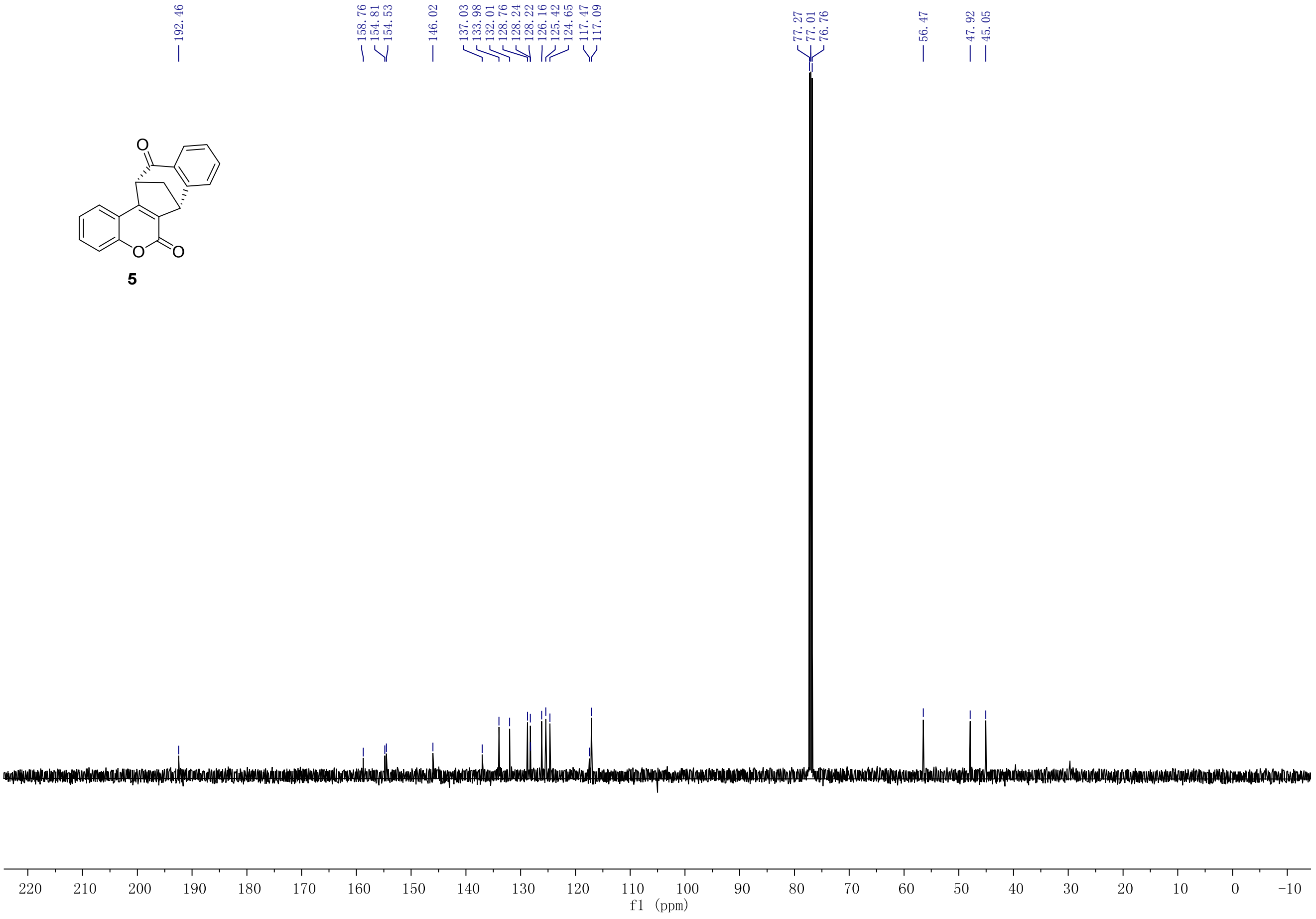
192.46
158.76
154.81
154.53
146.02
137.03
133.98
132.01
128.76
128.24
128.22
126.16
125.42
124.65
117.47
117.09
77.27
77.01
76.76
56.47
47.92
45.05
5
f1 (ppm)
